# Supplementary material for: Skeletal metalation of lactams through a carbonyl-to-nickel-exchange logic
Source: Nat Commun. 2023 Aug 29;14:5273. doi: 10.1038/s41467-023-40979-3 (PMC10465567; doi:10.1038/s41467-023-40979-3)
Supplement: Supplementary file 1 — Supplementary Information [file 41467_2023_40979_MOESM1_ESM.pdf]

Supplementary Information

Skeletal Metalation of Lactams through a Carbonyl-to-Nickel-Exchange Logic

Hongyu Zhong,[1] Dominic T. Egger,[1] Valentina C. M. Gasser,[1†] Patrick Finkelstein,[1†] Loris Keim,[1] Merlin Z. Seidel,[1] Nils Trapp[1] and Bill Morandi[1]*

[1]Laboratorium für Organische Chemie, ETH Zürich, 8093 Zürich, Switzerland

*Corresponding author. Email: bill.morandi@org.chem.ethz.ch

†These authors contributed equally to this work

Table of Contents

1. General Information ... 2

2. Synthesis and characterization of organometallic compounds ... 4

3. Synthesis and characterization of organic compounds ... 23

3.1 Synthesis and characterization of organic substrates ... 23

3.2 Reactivity of organonickel complexes and characterization of organic products ... 32

4. Computational studies ... 38

4.1 General information ... 38

4.2 DFT-computed relative ground state energies of (IPr)Ni(0) precursors ... 43

4.3 Correctional term for the barrier of C–N bond oxidative addition ... 45

4.4 Participation of the Boc group in the C–N bond oxidative addition transition states ... 46

4.5 Investigations into the CO dissociation step ... 47

5. Organometallic studies ... 50

5.1 Evaluation of nitrogen substituents and nickel precursors ... 50

5.2. NMR monitoring of C–N bond oxidative addition and CO deinsertion reaction progress ... 52

5.3. *β*-hydride elimination of metallacycles ... 62

5.4 Reactions between metallacycles and $CO_{(g)}$ ... 67

6. X-ray crystallographic data ... 75

6.1 Comparative analysis of X-ray structure parameters of 5- to 8-membered nickellacycles ... 75

6.2 Crystal structure tables ... 79

6.3 ORTEP plots of X-ray structures and solid-state parameters ... 85

7. NMR spectra of organometallic compounds ... 107

8. NMR spectra of organic compounds ... 127

9. IR spectra of organometallic compounds ... 135

References ... 143

1. General Information

All air- and moisture-sensitive experiments were carried out in an MBraun glovebox under an argon atmosphere or using standard Schlenk line techniques under nitrogen atmosphere. All glassware were dried and stored in an oven prior to use. Solvents for air-sensitive manipulations were dried using the appropriate drying agents, degassed and stored over molecular sieves. Bis(1,5-cyclooctadiene)nickel(0) was purchased from Strem and used as received. 1,3-Bis(2,6-diisopropylphenyl)imidazol-2-ylidene was purchased from TCI and used as received. Compounds *N*–Boc-2-pyrrolidinone (**1**), *N*–Boc-2-piperidinone (**2**) and *N*–Boc-2-azepanone (**16**) were purchased from commercial suppliers and dried under high vacuum prior to use. Unless otherwise noted, chemicals used to synthesize organic substrates were purchased from commercial suppliers and used without purification.

Nuclear magnetic resonance spectra were acquired on a Bruker Avance III 400 MHz, a Bruker Neo 400 MHz or a Bruker Avance III 500 MHz instrument, all equipped with a BBFO probe at the NMR facility of ETH Zürich. The proton signal of the residual nondeuterated solvent was used as an internal reference for ^{1}H NMR spectra. The carbon signal of the deuterated solvent was used as an internal reference for $^{13}C\{^{1}H\}$ NMR spectra. Coupling constants are reported in Hz. Multiplicities are indicated by s (singlet), d (doublet), t (triplet), q (quartet), p (pentet), hept (heptet), m (multiplet), br (broad), app (apparent) and combinations thereof. High-resolution mass spectra were provided by the mass spectrometry service facility in the Laboratories of Organic Chemistry at ETH Zürich. Infrared (ATR-IR) spectra were recorded in an argon-filled glovebox on a Bruker ALPHA II FT-IR spectrometer. The absorption bands are reported in cm^{-1} and are described as follows: very strong (vs), strong (s), middle (m), weak (w).

Single crystal structure data were collected on a Rigaku Oxford Diffraction XtaLAB Synergy-S dual-wavelength kappa diffractometer equipped with a Dectris Pilatus 300 HPAD detector and using microfocus sealed tube Cu-Kα or Mo-Kα radiation (mirror optics). Crystals were coated with polybutene oil in a glovebox under argon atmosphere and sealed in a screw-cap jar for transfer. Specimen were mounted on Kapton sample holders (MiTeGen) for measurement. If solvent loss or other sample degradation were observed, a μCHILL device[1] was employed for low-temperature sample preparation under N_2 atmosphere. All measurements were carried out at 100K using an Oxford Cryosystems Cryostream 800 N_2-atmosphere sample cryostat. Data were integrated using the CrysAlisPro software suite and corrected for absorption effects using a combination of empirical (ABSPACK) and numerical corrections.[2] The structures were solved using SHELXS[3] or SHELXT[4] and refined by full-matrix least squares analysis (SHELXL),[3,5] using the program package OLEX2.[6] All non-hydrogen atoms were refined anisotropically. Hydrogen atoms were constrained to ideal geometries and refined with fixed isotropic displacement parameters (in terms of a riding model). CCDC 2227410-2227427 contain the supplementary crystallographic data for this paper, including structure factors and refinement instructions. These data can be obtained free of charge from The Cambridge Crystallographic Data

Centre, 12 Union Road, Cambridge CB2 1EZ, UK (fax: +44(1223)-336-033; e-mail: deposit@ccdc.cam.ac.uk), or via https://www.ccdc.cam.ac.uk/structures.

Analytical thin-layer chromatography was performed on pre-coated silica gel plates (Merck, 0.25 mm silica gel Si 60, F254). Visualization of the developed chromatogram was either performed by UV absorbance at a wavelength of λ = 254 nm or $KMnO_4$ stain. For the purification of organic substances, column chromatography using silica gel 60 (particle size 40 – 63 µm, Silicycle) was performed using technical grade solvents.

Safety note: although formation of the low-boiling, poisonous $Ni(CO)_4$ species from the solid $(IPr)Ni(CO)_3$ or alternative (IPr)Ni-carbonyl species is highly unlikely under the reaction conditions and at the reaction scale in this report, cautions must still been taken for reaction workup outside the glovebox and operations must be conducted in well-ventilated fume hoods.

2. Synthesis and characterization of organometallic compounds

2.1 Synthesis and characterization of (IPr)Ni(η^6-PhMe)

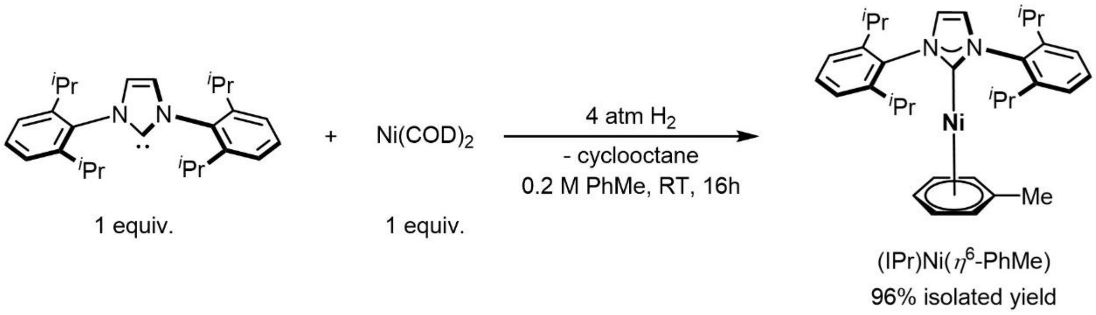

The title compound was prepared according to an adapted procedure first reported by Ogoshi.[7] In an argon-filled glovebox, a 250 mL J-Young flask was charged with IPr (2 mmol, 777.2 mg), Ni(COD)$_2$ (2 mmol, 550.1 mg), 10 mL toluene and a stir bar. The slurry was stirred at room temperature for an hour to afford a homogeneous solution. The solution and the whole J-Young flask glass body were frozen in liquid nitrogen and the headspace was evacuated. Four atm of H_2 was added, and the solution was allowed to warm up to room temperature. The reaction was stirred at room temperature for 16 hours and degassed on the vacuum line. The J-Young flask was brought back into the glovebox. The solution was then filtered, and the volatiles were removed under vacuum to afford a dark-red oil. The product was dissolved in *n*-hexane and evacuated to remove residual toluene. The resulting red oil was scraped using a metal spatula and grinded against the wall of the glass vial, followed by pulling vacuum. The operation was repeated multiple times until the oil solidified that afforded the product as a dark red powder (1.92 mmol, 1035.5 mg, 96%,). The title compound was found to dimerize in *n*-hexane solution by coordination to the IPr arene backbone and loss of toluene. Crystals of the dimeric (IPrNi)$_2$[8] suitable for X-ray diffraction were grown from a concentrated *n*-hexane solution at –35 °C.

^{1}H NMR (500 MHz, benzene-d_6, 23 °C): δ 7.33 – 7.27 (m, 2H, 2×*para*-CH_{Dipp}), 7.20 – 7.17 (m, 4H, 4×*meta*-CH_{Dipp}), 6.37 (s, 2H, N(CH)$_2$N), 2.91 (hept, J = 6.9 Hz, 4H, 4×CH(CH$_3$)$_2$), 1.40 (d, J = 6.9 Hz, 12H, 2×CH(CH_3)$_2$), 1.12 (d, J = 6.9 Hz, 12H, 2×CH(CH_3)$_2$) (complete arene exchange with benzene-d_6).

^{1}H NMR (500 MHz, toluene-d_8, 23 °C): δ 7.29 – 7.23 (m, 2H, 2×*para*-CH_{Dipp}), 7.18 – 7.13 (m, 4H, 4×*meta*-CH_{Dipp}), 6.41 (s, 2H, N(CH)$_2$N), 2.93 (hept, J = 6.9 Hz, 4H, 4×CH(CH$_3$)$_2$), 1.38 (d, J = 6.9 Hz, 12H, 2×CH(CH_3)$_2$), 1.10 (d, J = 6.9 Hz, 12H, 2×CH(CH_3)$_2$) (complete arene exchange with toluene-d_8).

^{13}C{^{1}H} NMR (126 MHz, toluene-d_8, 23 °C): δ 195.6 (Ni-C_{carbene}), 146.6 (*ortho*-C_{Dipp}), 137.5 (*ipso*-C_{Dipp}), 128.6 (*para*-C_{Dipp}), 123.4 (N(CH)$_2$N), 119.4 (*meta*-C_{Dipp}), 28.7 (CH(CH$_3$)$_2$), 24.5 (CH(CH$_3$)$_2$), 23.5 (CH(CH$_3$)$_2$) (complete arene exchange with toluene-d_8).

2.2 Synthesis and characterization of **Ni-1**

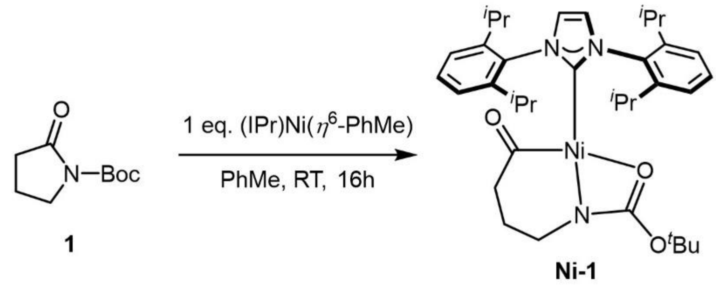

In an argon-filled glovebox, a 150 mL Schlenk flask was charged with (IPr)Ni(η^6-PhMe) (0.2 mmol, 107.9 mg), **1** (0.2 mmol, 37.0 mg), 5 mL toluene and a stir bar. The solution was stirred under room temperature for 16 hours. The volatiles were then removed under vacuum. To the residue was added 1 mL n-hexane which afforded a slurry. The content was filtered, and the precipitate was washed with 0.5 mL *n*-hexane twice. The precipitate and the filtrate were both collected. The precipitate was dried under vacuum to give nickel product **Ni-1** (52.6 mg, 0.087 mmol, 43.5%) as a yellow powder. The filtrate was concentrated and recrystallized by slow evaporation of *n*-hexane at room temperature over 24 hours to give a second crop of **Ni-1** (35.6 mg, 0.059 mmol, 29.7%; combined yield: 88.2 mg, 0.146 mmol, 73.2%). Slow evaporation of a concentrated *n*-hexane solution of **Ni-1** at room temperature over 24 hours afforded crystals suitable for X-ray diffraction.

^{1}H NMR (500 MHz, benzene-d_6, 23 °C): δ 7.32-7.26 (m, 2H, 2×*para*-CH_{Dipp}), 7.26-7.22 (m, 4H, 4×*meta*-CH_{Dipp}), 6.57 (s, 2H, N(CH)$_2$N), 3.28 (hept, ^3J = 6.7 Hz, 4H, 4×CH(CH$_3$)$_2$), 2.82 (t, ^3J = 6.0 Hz, 2H, NCH_2), 1.64 (d, ^3J = 6.7 Hz, 12H, 2×CH(CH_3)$_2$), 1.49-1.41 (m, 2H, C(O)CH_2), 1.29 (s, 9H, C(CH_3)$_3$), 1.26-1.22 (m, 2H, CH$_2$(CH_2)CH$_2$), 1.11 (d, ^3J = 6.7 Hz, 12H, 2×CH(CH_3)$_2$).

^{13}C{^{1}H} NMR (126 MHz, benzene-d_6, 23 °C) δ 237.4 (Ni-*C*(O)), 188.0 (Ni-C_{carbene}), 162.4 (*C*(O)$_{\text{Boc}}$), 146.9 (*ortho*-C_{Dipp}), 136.5 (*ipso*-C_{Dipp}), 129.8 (*para*-C_{Dipp}), 124.2 (N(*C*H)$_2$N), 123.1(*meta*-C_{Dipp}), 77.6 (O*C*(CH$_3$)$_3$), 42.3 (C(O)*C*H$_2$), 39.8 (N*C*H$_2$), 28.9 (*C*H(CH$_3$)$_2$), 28.8 (C(*C*H$_3$)$_3$), 27.1(CH$_2$(*C*H$_2$)CH$_2$), 26.1 (CH(*C*H$_3$)$_2$), 23.6 (CH(*C*H$_3$)$_2$).

IR (ATR) 2960 (m), 2926 (w), 2866 (w), 2833 (w), 1638 (s, ν(CO), nickel acyl), 1549 (s, ν(CO), Boc carbonyl), 1465 (s), 1430 (vs), 1403 (m), 1384 (w), 1362 (m), 1333 (m), 1277 (w), 1211 (w), 1183 (w), 1150 (vs), 1042 (w), 977 (w), 941 (w), 828 (w), 801 (s), 758 (vs), 728 (s), 704 (s), 610 (m), 571 (m), 441 (m) cm^{-1}.

2.3 Synthesis and characterization of **Ni-2**

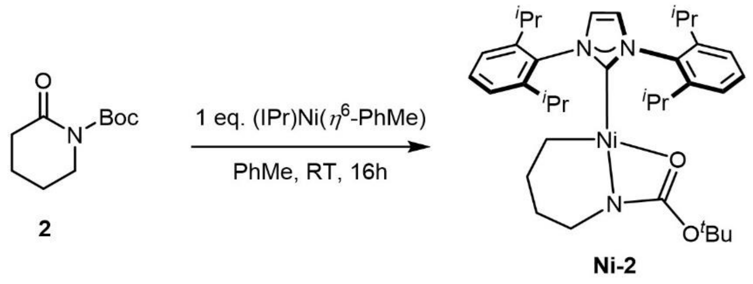

In an argon-filled glovebox, a 150 mL Schlenk flask was charged with (IPr)Ni(η^6-PhMe) (0.2 mmol, 107.9 mg), **2** (0.2 mmol, 39.9 mg), 3 mL toluene and a stir bar. The solution was stirred under room temperature for 16 hours. The volatiles were then removed under vacuum. To the residue was added 1 mL *n*-hexane which afforded a slurry. The content was filtered, and the precipitate and the filtrate were both collected. The precipitate was dried under vacuum to give nickel product **Ni-2** (77.6 mg, 0.125 mmol, 62.7%) as a yellow powder. The filtrate was concentrated and recrystallized by slow evaporation of *n*-hexane at room temperature over 3 days to give a second crop of **Ni-2** (14.8 mg, 0.024 mmol, 12.0%; combined yield: 92.4 mg, 0.149 mmol, 74.7%). Cooling a concentrated *n*-hexane solution of **Ni-2** at -35 °C overnight afforded crystals suitable for X-ray diffraction.

^{1}H NMR (500 MHz, benzene-d_6, 23 °C): δ 7.34-7.29 (m, 2H, 2×*para*-CH_{Dipp}), 7.27-7.22 (m, 4H, 4×*meta*-CH_{Dipp}), 6.50 (s, 2H, N(CH)$_2$N), 3.18 (hept, ^3J = 6.8 Hz, 4H, 4×CH(CH$_3$)$_2$), 2.74 (t, ^3J = 6.0 Hz, 2H, NCH_2), 1.65 (d, ^3J = 6.8 Hz, 12H, 2×CH(CH_3)$_2$), 1.28 (s, 9H, overlapping, C(CH_3)$_3$), 1.26 (m, 2H, NCH$_2$CH_2, overlapping), 1.11 (d, ^3J = 6.9 Hz, 12H, 2×CH(CH_3)$_2$), 0.75-0.65 (m, 2H, NiCH$_2$CH_2), 0.48-0.41 (m, 2H, NiCH_2).

^{13}C{^{1}H} NMR (126 MHz, benzene-d_6, 23 °C): δ 189.9 (Ni-C_{carbene}), 161.1 (C(O)$_{\text{Boc}}$), 146.4 (*ortho*-C_{Dipp}), 136.8 (*ipso*-C_{Dipp}), 129.7 (*para*-C_{Dipp}), 124.2 (*meta*-C_{Dipp}), 123.6 (N(CH)$_2$N), 76.9 (OC(CH$_3$)$_3$), 38.9 (NCH$_2$), 30.9 (NCH$_2$$CH_2$), 29.1($C$H(CH$_3$)$_2$), 28.9 (C($CH_3$)$_3$), 26.4 (NiCH$_2$$CH_2$), 25.8 (CH($CH_3$)$_2$), 23.6 (CH($CH_3$)$_2$), 1.6 (Ni$CH_2$).

IR (ATR): 2960 (m), 2926 (w), 2912 (w), 2869 (w), 2821 (w), 1551 (s, ν(CO), Boc carbonyl), 1466 (m), 1427 (vs), 1395 (s), 1345 (w), 1326 (m), 1263 (w), 1206 (w), 1157 (vs), 1138 (s), 1106 (w), 1059 (w), 1038 (w), 1015 (m), 938 (m), 802 (s), 758 (s), 747 (s), 710 (m), 612 (s), 451 (m), 427 (m) cm^{-1}.

2.4 Synthesis and characterization of **Ni-3a**

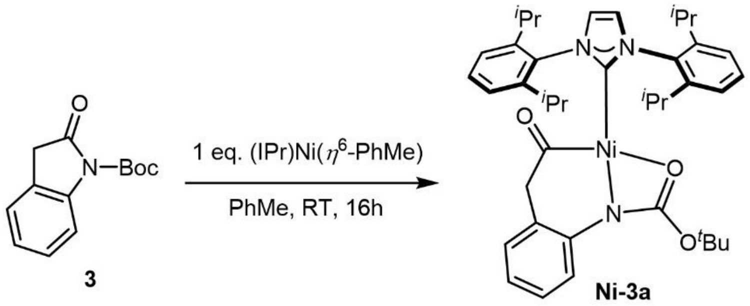

In an argon-filled glovebox, a 150 mL Schlenk flask was charged with (IPr)Ni(η^6-PhMe) (0.2 mmol, 107.9 mg), **3** (0.2 mmol, 46.7 mg), 5 mL toluene and a stir bar. The solution was stirred under room temperature for 16 hours. The volatiles were then removed under vacuum. To the residue was added 2 mL *n*-hexane which afforded a slurry. The content was filtered, and the precipitate was collected, washed with 0.5 mL *n*-hexane twice and dried under vacuum to give nickel product **Ni-3a** (91.2 mg, 0.134 mmol, 67.0%) as an orange powder. Slow evaporation of a concentrated toluene solution of **Ni-3a** at room temperature afforded crystals suitable for X-ray diffraction.

^{1}H NMR (500 MHz, benzene-d_6, 23 °C): δ 7.69 (dd, ^3J = 8.2 Hz, ^4J = 1.2 Hz, 1H, Ar-*H*), 7.31 – 7.24 (m, 2H, 2×*para*-CH_{Dipp}), 7.23 – 7.18 (m, 4H, 4×*meta*-CH_{Dipp}), 7.14 – 7.07 (m, 1H, Ar-*H*), 6.74 – 6.69 (m, 1H, Ar-*H*), 6.63 (td, ^3J = 7.3 Hz, ^4J = 1.2 Hz, 1H, Ar-*H*), 6.58 (s, 2H, N(C*H*)$_2$N), 3.22 (hept, ^3J = 6.9 Hz, 4H, 4×C*H*(CH$_3$)$_2$), 3.02 (s, 2H, NiC(O)CH_2), 1.59 (d, ^3J = 6.7 Hz, 12H, 2×CH(CH_3)$_2$), 1.21 (s, 9H, C(CH_3)$_3$), 1.10 (d, ^3J = 7.0 Hz, 12H, 2×CH(CH_3)$_2$).

^{13}C{^{1}H} NMR (126 MHz, benzene-d_6, 23 °C): δ 236.7 (Ni-*C*(O)), 187.1 (Ni-$C_{carbene}$), 160.9 (*C*(O)$_{Boc}$), 146.8 (*ortho*-C_{Dipp}), 139.9 (Ar), 136.2 (*ipso*-C_{Dipp}), 130.0 (*para*-C_{Dipp}), 128.8 (Ar), 127.2 (Ar), 127.0 (Ar), 124.4 (*meta*-C_{Dipp}), 123.3 (N(*C*H)$_2$N), 119.7 (Ar), 118.7 (Ar), 80.3 (O*C*(CH$_3$)$_3$), 50.4 (NiC(O)*C*H$_2$), 29.0 (*C*H(CH$_3$)$_2$), 28.4 (C(*C*H$_3$)$_3$), 26.1 (CH(*C*H$_3$)$_2$), 23.5 (CH(*C*H$_3$)$_2$).

IR (ATR): 2961 (m), 2925 (w), 2864(w), 1670 (vs, ν(CO), nickel acyl), 1528 (vs, ν(CO), Boc carbonyl), 1495 (vs), 1456 (s), 1428 (vs), 1405 (m), 1383 (w), 1363 (m), 1337 (m), 1310 (s), 1266 (s), 1162 (vs), 1116 (s), 1045 (s), 1027 (s), 980 (m), 967 (m), 942 (w), 821 (w), 799 (m), 756 (s), 748 (vs), 731 (s), 708 (m), 694 (m), 662 (m), 597 (m), 547 (w), 513 (w), 458 (m), 443 (w), 423 (w) cm^{-1}.

2.5 Formation of **Ni-3b**

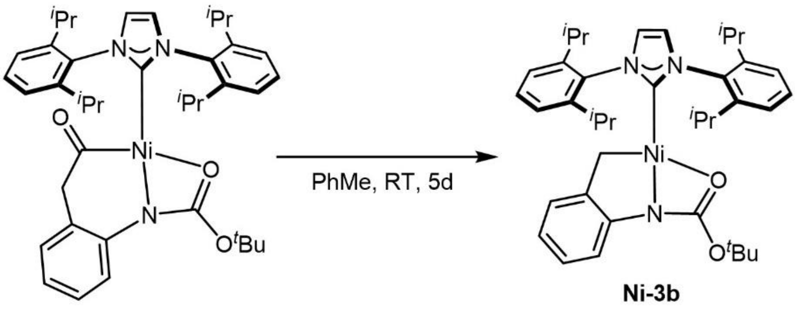

In an argon-filled glovebox, an 8 mL scintillation vial was charged with **Ni-3a** (0.05 mmol, 34.0 mg), 2 mL toluene, a stir bar and sealed. The solution was stirred at room temperature for 5 days, and the volatiles were removed under vacuum. The product was sampled for ^{1}H NMR analysis, and a mixture of **Ni-3a** : **Ni-3b** = **1.8 :1** along with lactam **3a** and IPrNi(CO)$_3$ were formed. Attempts to facilitate **Ni-3b** formation at 50 °C led to decomposition and formation of paramagnetic nickel species. Starting from **Ni-3a** and 2 equivalents of (IPr)Ni(η^6-PhMe) at RT or 50 °C also did not afford full conversion to **Ni-3b**. Separation of **Ni-3b** from residual **Ni-3a** by recrystallization was unsuccessful.

^{1}H NMR (500 MHz, benzene-d_6, 23 °C): δ 7.66 – 7.63 (m, 1H, Ar-*H*), 7.31 – 7.24 (m, 2H, 2×*para*-C*H*$_{\text{Dipp}}$), 7.23 – 7.18 (m, 4H, 4×*meta*-C*H*$_{\text{Dipp}}$), 7.10 – 7.07 (m, 1H, Ar-*H*), 6.74 – 6.69 (m, 1H, Ar-*H*), 6.75 (td, *J* = 7.3, 1.1 Hz, 1H, Ar-*H*), 6.43 (s, 2H, N(C*H*)$_2$N), 3.13 (hept, ^{3}J = 6.9 Hz, 4H, 4×C*H*(CH$_3$)$_2$), 1.53 (s, 2H, NiC*H*$_2$), 1.51 (d, ^{3}J = 6.9 Hz, 12H, 2×CH(C*H*$_3$)$_2$), 1.37 (s, 9H, C(C*H*$_3$)$_3$), 1.02 (d, ^{3}J = 7.0 Hz, 12H, 2×CH(C*H*$_3$)$_2$).

2.6 Synthesis and characterization of **Ni-4**

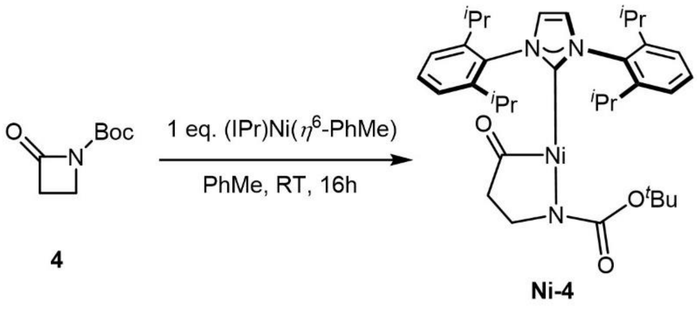

In an argon-filled glovebox, a 150 mL Schlenk flask was charged with (IPr)Ni(η^6-PhMe) (0.2 mmol, 107.9 mg), **4** (0.2 mmol, 34.2 mg), 5 mL toluene and a stir bar. The solution was stirred under room temperature for 16 hours. The volatiles were then removed under vacuum. To the residue was added 2 mL *n*-hexane which afforded a solution. The content was filtered, concentrated under vacuum and kept at -35 °C for 2 days for recrystallization. The nickel product **Ni-4** (64.9 mg, 0.105 mmol, 52.5%) was obtained as a black solid. Cooling a concentrated *n*-hexane solution of **Ni-4** at -35 °C afforded crystals suitable for X-ray diffraction.

^{1}H NMR (500 MHz, benzene-d_6, 23 °C): δ 7.29 – 7.25 (m, 2H, 2×*para*-CH_{Dipp}), 7.25 – 7.21 (m, 4H, 4×*meta*-CH_{Dipp}), 6.54 (s, 2H, N(CH)$_2$N), 3.01 (hept, ^3J = 6.9 Hz, 4H, 4×CH(CH$_3$)$_2$), 2.52 (t, ^3J = 6.7 Hz, 2H, NCH$_2$CH_2), 1.92 (t, ^3J = 6.7 Hz, 2H, NCH_2), 1.56 (d, ^3J = 6.9 Hz, 12H, 2×CH(CH_3)$_2$), 1.43 (s, 9H, C(CH_3)$_3$), 1.08 (d, ^3J = 6.9 Hz, 12H, 2×CH(CH_3)$_2$).

^{13}C{^{1}H} NMR (126 MHz, benzene-d_6, 23 °C): δ 188.4 (Ni-C_{carbene}), 146.8 (*ortho*-C_{Dipp}), 136.2 (*ipso*-C_{Dipp}), 130.3 (*para*-C_{Dipp}), 124.4 (*meta*-C_{Dipp}), 123.6 (N(CH)$_2$N), 76.9 (OC(CH$_3$)$_3$), 53.7 (NCH$_2$), 40.4 (NCH$_2$$CH_2$), 29.0 ($C$H(CH$_3$)$_2$), 28.9 (C($CH_3$)$_3$), 25.9 (CH($CH_3$)$_2$), 23.7 (CH($CH_3$)$_2$). The nickel acyl carbon signal and the Boc carbonyl carbon signal were not located potentially caused by dynamic solution behavior of **Ni-4**.

IR (ATR): 3109 (w), 3073(w), 2965(m), 2924(w), 2867(w), 1716 (s, ν(CO), nickel acyl), 1628 (vs, ν(CO), uncoordinated Boc carbonyl), 1459 (s), 1445 (s), 1405 (s), 1381 (s), 1362 (s), 1335 (vs), 1270 (w), 1163 (s), 1116 (w), 1063 (s), 945 (m), 868 (w), 801 (s), 755 (s), 708 (s), 571(m) cm^{-1}.

2.7 Synthesis and characterization of **Ni-5**

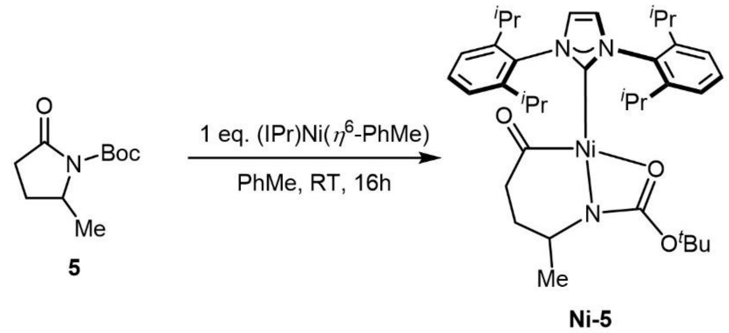

In an argon-filled glovebox, a 150 mL Schlenk flask was charged with (IPr)Ni(η^6-PhMe) (0.2 mmol, 107.9 mg), **5** (0.2 mmol, 39.9 mg), 5 mL toluene and a stir bar. The solution was stirred under room temperature for 16 hours. The volatiles were then removed under vacuum. To the residue was added 2 mL *n*-hexane which afforded a slurry. The content was filtered, and the precipitate was collected, washed with 0.5 mL *n*-hexane twice and dried under vacuum to give nickel product **Ni-5** (101.9 mg, 0.158 mmol, 78.8%) as a yellow powder. Slow evaporation of a concentrated *n*-hexane solution of **Ni-5** at room temperature afforded crystals suitable for X-ray diffraction.

^{1}H NMR (500 MHz, benzene-d_6, 23 °C): δ 7.30 – 7.26 (m, 2H, 2×*para*-CH_{Dipp}), 7.26 – 7.21 (m, 4H, 4×*meta*-CH_{Dipp}), 6.56 (s, 2H, N(C*H*)$_2$N), 3.40 (hept, ^3J = 6.8 Hz, 2H, 2×C*H*(CH$_3$)$_2$), 3.28 – 3.19 (m, 1H, N(C*H*)), 3.16 (hept, ^3J = 6.8 Hz, 2H, 2×C*H*(CH$_3$)$_2$), 1.69 (d, ^3J = 6.8 Hz, 6H, CH(CH_3)$_2$), 1.61 (d, ^3J = 6.8 Hz, 6H, CH(CH_3)$_2$), 1.56 – 1.50 (m, 2H, C(O)CH$_2$CH_2), 1.42 – 1.36 (m, 2H, C(O)CH_2), 1.26 (s, 9H, C(CH_3)$_3$), 1.11 (d, ^3J = 6.8 Hz, 6H, CH(CH_3)$_2$), 1.09 (d, ^3J = 6.8 Hz, 6H, CH(CH_3)$_2$), 1.02 (d, ^3J = 6.1 Hz, 3H, NCHCH_3).

^{13}C{^{1}H} NMR (126 MHz, benzene-d_6, 23 °C): δ 239.1 (Ni-*C*(O)), 188.0 (Ni-C_{carbene}), 162.4 (*C*(O)$_{\mathrm{Boc}}$), 146.9 (*ortho*-C_{Dipp}), 146.8 (*ortho*-C_{Dipp}), 136.5 (*ipso*-C_{Dipp}), 129.8 (*para*-C_{Dipp}), 124.2 (*meta*-C_{Dipp}), 124.1 (*meta*-C_{Dipp}), 123.0 (N(*C*H)$_2$N), 77.7 (O*C*(CH$_3$)$_3$), 43.7 (N(*C*H)), 40.0 (NCH*C*H$_2$), 34.4 (Ni(CO)*C*H$_2$), 28.9 (*C*H(CH$_3$)$_2$), 28.9 (*C*H(CH$_3$)$_2$), 28.8 (C(*C*H$_3$)$_3$), 26.1 (CH(*C*H$_3$)$_2$), 26.0 (CH(*C*H$_3$)$_2$), 23.7 (CH(*C*H$_3$)$_2$), 23.5 (CH(*C*H$_3$)$_2$), 21.2 (NCH*C*H$_3$).

IR (ATR): 2961 (m), 2930 (w), 2869 (w), 2833 (w), 1672 (w), 1652 (s, ν(CO), nickel acyl), 1526 (vs, ν(CO), Boc carbonyl), 1453 (s), 1411 (vs), 1363 (s), 1327 (s), 1145 (vs), 1090 (s), 1048 (m), 1016 (m), 983 (s), 938 (m), 802 (s), 755 (vs), 578 (m), 458 (m) cm^{-1}.

2.8 Synthesis and characterization of **Ni-6**

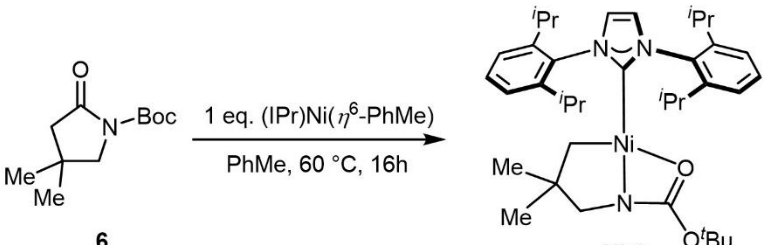

In an argon-filled glovebox, a 150 mL Schlenk flask was charged with (IPr)Ni(η^6-PhMe) (0.2 mmol, 107.9 mg), **6** (0.2 mmol, 42.7 mg), 5 mL toluene and a stir bar. The solution was stirred at 60 °C for 16 hours. The volatiles were then removed under vacuum. To the residue was added 2 mL *n*-hexane which afforded a solution. The content was filtered and concentrated under vacuum. Recrystallization by slow evaporation of *n*-hexane at room temperature from multiple crops afforded the nickel product **Ni-6** (83.0 mg, 0.131 mmol, 65.6%) as a black-red solid. Slow evaporation of a concentrated *n*-hexane solution of **Ni-6** at room temperature afforded crystals suitable for X-ray diffraction.

^{1}H NMR (500 MHz, benzene-d_6, 23 °C): δ 7.31 – 7.25 (m, 2H, 2×*para*-CH_{Dipp}), 7.23 – 7.18 (m, 4H, 4×*meta*-CH_{Dipp}), 6.44 (s, 2H, N(CH)$_2$N), 3.05 (hept, ^3J = 6.8 Hz, 4H, 4×CH(CH$_3$)$_2$), 2.74 (br s, 2H, NCH_2), 1.64 (d, ^3J = 6.8 Hz, 12H, 2×CH(CH_3)$_2$), 1.37 (s, 9H, C(CH_3)$_3$), 1.08 (d, ^3J = 6.8 Hz, 12H, 2×CH(CH_3)$_2$), 0.94 (s, 6H, (CH_3)$_2$), 0.84 (br s, 2H, NiCH_2).

^{13}C{^{1}H} NMR (126 MHz, benzene-d_6, 23 °C): δ 186.8 (Ni-$C_{carbene}$), 146.3 (*ortho*-C_{Dipp}), 136.7 (*ipso*-C_{Dipp}), 130.0 (*para*-C_{Dipp}), 124.3 (*meta*-C_{Dipp}), 123.7 (N(CH)$_2$N), 76.7 (OC(CH$_3$)$_3$), 32.0 (NCH$_2$), 29.1 (C(CH$_3$)$_3$), 27.9 (CH(CH$_3$)$_2$), 25.7 (CH(CH$_3$)$_2$), 23.5 (CH(CH$_3$)$_2$), 23.1 (NCH$_2$$C(Me)_2$), 14.4 (Ni$CH_2$). The C(O) signal of Boc group was not located.

IR (ATR): 2960 (m), 2928 (w), 2868 (w), 1945 (w), 1584 (s, ν(CO), Boc carbonyl), 1454 (s), 1396 (s), 1359 (s), 1323 (s), 1146 (s), 1049 (m), 1009 (m), 938 (m), 801 (m), 756 (s), 703 (m), 652 (m), 456 (m) cm^{-1}.

2.9 Synthesis and characterization of **Ni-7a**

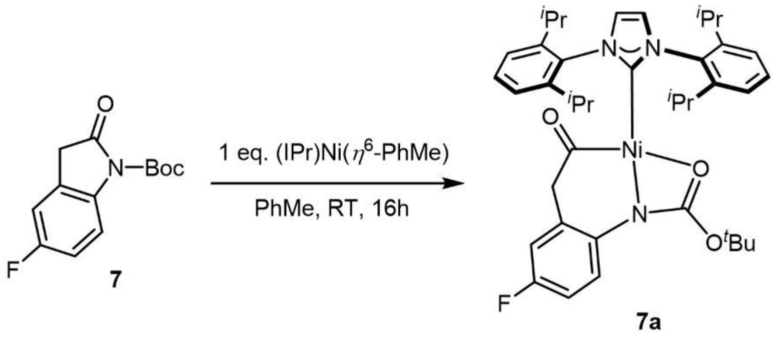

In an argon-filled glovebox, a 150 mL Schlenk flask was charged with (IPr)Ni(η^6-PhMe) (0.2 mmol, 107.9 mg), **7** (0.2 mmol, 50.3 mg), 5 mL toluene and a stir bar. The solution was stirred under room temperature for 16 hours. The volatiles were then removed under vacuum. To the residue was added 2 mL *n*-hexane which afforded a slurry. The content was filtered, and the precipitate was collected, washed with 0.5 mL *n*-hexane twice and dried under vacuum to give nickel product **Ni-7a** (99.5 mg, 0.142 mmol, 71.2%) as a brownish orange powder. Slow evaporation of a concentrated toluene solution of **Ni-7a** at room temperature afforded crystals suitable for X-ray diffraction.

^{1}H NMR (500 MHz, benzene-d_6, 23 °C): δ 7.47 (dd, $^3J_{H\text{-}F}$ = 8.9 Hz, ^3J = 5.3 Hz, 1H, Ar-*H*), 7.30 – 7.25 (m, 2H, 2×*para*-C*H*$_{Dipp}$), 7.24 – 7.18 (m, 4H, 4×*meta*-C*H*$_{Dipp}$), 6.76 (td, $^4J_{H\text{-}F}$ = 8.7 Hz, ^3J = 3.0 Hz, 1H, Ar-*H*), 6.57 (s, 2H, N(C*H*)$_2$N), 6.37 (dd, $^3J_{H\text{-}F}$ = 9.1 Hz, ^3J = 3.0 Hz, 1H, Ar-*H*), 3.18 (hept, ^3J = 6.8 Hz, 4H, 4×C*H*(CH$_3$)$_2$), 2.86 (s, 2H, C(O)C*H*$_2$), 1.57 (d, ^3J = 6.8 Hz, 12H, 2×CH(C*H*$_3$)$_2$), 1.19 (s, 9H, C(C*H*$_3$)$_3$), 1.09 (d, ^3J = 6.8 Hz, 12H, 2×CH(C*H*$_3$)$_2$).

^{13}C{^{1}H} NMR (126 MHz, benzene-d_6, 23 °C): δ 235.6 (Ni-*C*(O)), 186.6 (Ni-*C*$_{carbene}$), 160.6 (*C*(O)$_{Boc}$), 156.7 (d, $^1J_{C\text{-}F}$ = 237.3 Hz, Ar), 146.8 (*ortho*-*C*$_{Dipp}$), 136.1 (*ipso*-*C*$_{Dipp}$), 135.9 (d, $^4J_{C\text{-}F}$ = 2.5 Hz, Ar), 130.0 (*para*-*C*$_{Dipp}$), 128.5 (d, $^4J_{C\text{-}F}$ = 2.9 Hz, Ar), 124.4 (*meta*-*C*$_{Dipp}$), 123.3 (N(*C*H)$_2$N), 119.1 (d, $^3J_{C\text{-}F}$ = 7.2 Hz, Ar), 115.2 (d, $^2J_{C\text{-}F}$ = 22.5 Hz, Ar), 113.2 (d, $^2J_{C\text{-}F}$ = 21.3 Hz, Ar), 80.4 (O*C*(CH$_3$)$_3$), 50.1 (NiC(O)*C*H$_2$), 29.0 (*C*H(CH$_3$)$_2$), 28.3 (C(*C*H$_3$)$_3$), 26.0 (CH(*C*H$_3$)$_2$), 23.4 (CH(*C*H$_3$)$_2$).

^{19}F{^{1}H} NMR (376 MHz, benzene-d_6, 23 °C): δ -124.7.

IR (ATR): 2963 (m), 2927 (w), 2868 (w), 1672 (vs, ν(CO), nickel acyl), 1576 (m), 1536 (s, ν(CO), Boc carbonyl), 1501 (s), 1473 (s), 1442 (vs), 1393 (s), 1363 (s), 1306 (s), 1269 (s), 1156 (vs), 1102 (vs), 1046 (m), 1027 (m), 981 (m), 831 (m), 802 (m), 756 (s), 730 (m), 696 (m), 588 (s), 461 (w) cm^{-1}.

2.10 Formation of **Ni-7b**

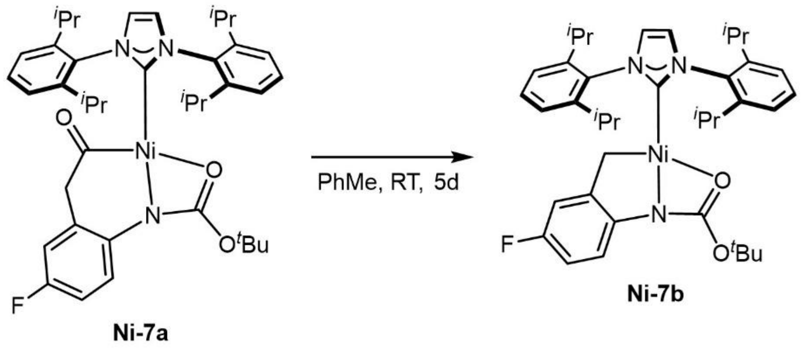

In an argon-filled glovebox, an 8 mL scintillation vial was charged with **Ni-7a** (0.05 mmol, 34.0 mg), 2 mL toluene, a stir bar and sealed. The solution was stirred at room temperature for 5 days, and the volatiles were removed under vacuum. The product was sampled for ^{1}H NMR and ^{19}F NMR analysis, and a mixture of **Ni-7a** : **Ni-7b** = **0.7 :1** were formed. Attempts to facilitate **Ni-7b** formation at 50 °C led to decomposition and formation of paramagnetic nickel species. Separation of **Ni-7b** from residual **Ni-7a** by recrystallization was unsuccessful.

^{1}H NMR (500 MHz, benzene-d_6, 23 °C): δ 7.45 (dd, $^3J_{H\text{-}F}$ = 11.5 Hz, ^3J = 6.7 Hz, 1H, Ar-*H*), 7.29 – 7.26 (m, 2H, 2×*para*-C*H*$_{Dipp}$), 7.25 – 7.19 (m, 4H, 4×*meta*-C*H*$_{Dipp}$), 7.14 – 7.10 (m, 1H, Ar-*H*), 7.09 – 7.05 (m, 1H, Ar-*H*), 6.83 – 6.77 (m, 1H, Ar-*H*), 6.40 (s, 2H, N(C*H*)$_2$N), 3.01 (hept, ^3J = 6.8 Hz, 4H, 4×C*H*(CH$_3$)$_2$), 1.43 (s, 2H, NiC*H*$_2$), 1.47 (d, ^3J = 6.8 Hz, 12H, 2×CH(C*H*$_3$)$_2$), 1.34 (s, 9H, C(C*H*$_3$)$_3$), 1.00 (d, ^3J = 6.8 Hz, 12H, 2×CH(C*H*$_3$)$_2$).

^{19}F{^{1}H} NMR (376 MHz, benzene-d_6, 23 °C): δ -127.04

2.11 Synthesis and characterization of **Ni-8**

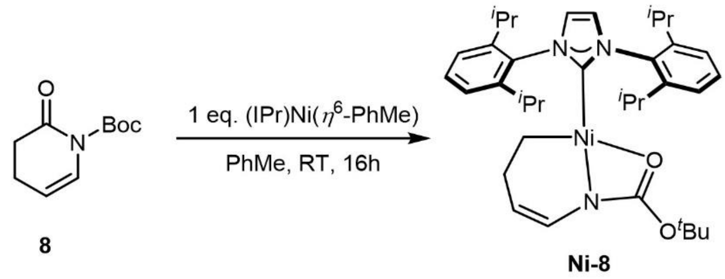

In an argon-filled glovebox, a 150 mL Schlenk flask was charged with (IPr)Ni(η^6-PhMe) (0.2 mmol, 107.9 mg), **8** (0.2 mmol, 39.4 mg), 5 mL toluene and a stir bar. The solution was stirred under room temperature for 16 hours. The volatiles were then removed under vacuum. To the residue was added 1 mL *n*-hexane which afforded a slurry. The content was filtered, and the precipitate was collected and dried under vacuum to give nickel product **Ni-8** (83.1 mg, 0.135 mmol, 67.4%) as a yellow powder. Slow evaporation of a concentrated *n*-hexane solution of **Ni-8** at room temperature afforded crystals suitable for X-ray diffraction.

^{1}H NMR (500 MHz, benzene-d_6, 23 °C): δ 7.32 – 7.28 (m, 2H, 2×*para*-CH_{Dipp}), 7.24 – 7.21 (m, 4H, 4×*meta*-CH_{Dipp}), 6.51 (s, 2H, N(C*H*)$_2$N), 6.44 (dt, $^3J_1$ = 7.3 Hz, $^4J_2$ = 1.9 Hz, 1H, N(C*H*)), 4.73 (dt, $^3J_1$ = 7.3 Hz, $^3J_2$ = 4.7 Hz, 1H, N(CH)(C*H*)), 3.13 (hept, ^3J = 6.9 Hz, 4H, 4×C*H*(CH$_3$)$_2$), 1.57 (d, ^3J = 6.9 Hz, 12H, 2×CH(CH_3)$_2$), 1.26 (dtd, $^3J_1$ = 5.9 Hz, $^3J_2$ = 4.7 Hz, $^4J_3$ = 1.9 Hz, 2H, NiCH$_2$CH_2), 1.23 (s, 9H, C(CH_3)$_3$), 1.08 (d, ^3J = 6.9 Hz, 12H, 2×CH(CH_3)$_2$), 0.91 (t, ^3J = 5.9 Hz, 2H, NiCH_2).

^{13}C{^{1}H} NMR (126 MHz, benzene-d_6, 23 °C): δ 189.3 (Ni-$C_{carbene}$), 158.8 (*C*(O)$_{Boc}$), 146.3 (*ortho*-C_{Dipp}), 136.5 (*ipso*-C_{Dipp}), 129.9 (*para*-C_{Dipp}), 124.9 (N(*C*H)), 124.2 (*meta*-C_{Dipp}), 123.7 (N(*C*H)$_2$N), 108.5 (N(CH)(*C*H)), 78.1 (O*C*(CH$_3$)$_3$), 29.1 (C(*C*H$_3$)$_3$), 28.6 (*C*H(CH$_3$)$_2$), 25.9 (CH(*C*H$_3$)$_2$), 24.7 (Ni(CH$_2$)(*C*H$_2$)), 23.4 (CH(*C*H$_3$)$_2$), 3.7 (Ni*C*H$_2$).

IR (ATR): 2962 (m), 2933 (w), 2870 (w), 2801 (w), 1964 (w), 1630 (w, ν(C=C), alkene), 1548 (s, ν(CO), Boc carbonyl), 1439 (s), 1381 (s), 1328 (s), 1253 (m), 1153 (s), 1114 (s), 1040 (s), 805 (s), 1003 (s), 939 (m), 856 (m), 806 (m), 762 (s), 734 (s), 714 (s), 615 (s), 459 (s) cm^{-1}.

2.12 Synthesis and characterization of **Ni-9**

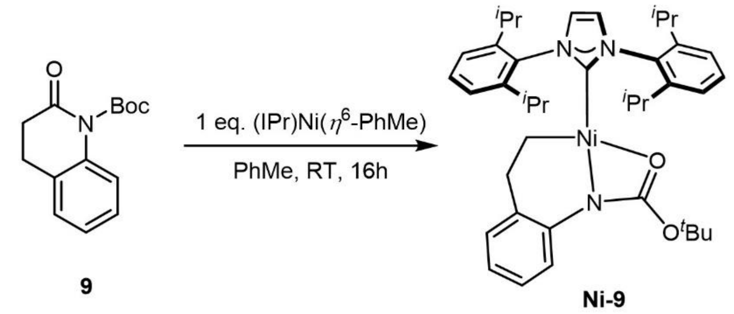

In an argon-filled glovebox, a 150 mL Schlenk flask was charged with (IPr)Ni(η^6-PhMe) (0.2 mmol, 107.9 mg), **9** (0.2 mmol, 49.5 mg), 5 mL toluene and a stir bar. The solution was stirred under room temperature for 16 hours. The volatiles were then removed under vacuum. To the residue was added 1 mL *n*-hexane which afforded a slurry. The content was filtered, and the precipitate was collected, washed with 0.5 mL *n*-hexane twice and dried under vacuum to give nickel product **Ni-9** (94.9 mg, 0.142 mmol, 71.2%) as an orange powder. Slow evaporation of a concentrated toluene solution of **Ni-9** at room temperature afforded crystals suitable for X-ray diffraction.

^{1}H NMR (500 MHz, benzene-d_6, 23 °C): δ 7.72 (dd, ^3J = 8.1, 1.3 Hz, 1H, Ar-H), 7.31 – 7.25 (m, 2H, 2×*para*-CH_{Dipp}), 7.22 – 7.18 (m, 4H, 4×*meta*-CH_{Dipp}), 7.16 – 7.13 (m, overlapping, 1H, Ar-H), 7.01 (dd, $^3J_1$ = 7.4 Hz, $^4J_2$ = 1.6 Hz, 1H, Ar-H), 6.74 (td, $^3J_1$ = 7.4 Hz, $^4J_2$ = 1.3 Hz, 1H, Ar-H), 6.53 (s, 2H, N(CH)$_2$N), 3.18 (hept, ^3J = 6.8 Hz, 4H, 4×CH(CH$_3$)$_2$), 1.93 (app t, ^3J = 5.8 Hz, 2H, NiCH$_2$CH_2), 1.53 (d, ^3J = 6.8 Hz, 12H, 2×CH(CH_3)$_2$), 1.25 (s, 9H, C(CH_3)$_3$), 1.08 (d, ^3J = 6.8 Hz, 12H, 2×CH(CH_3)$_2$), 0.72 (app t, ^3J = 5.8 Hz, 2H, NiCH_2).

^{13}C{^{1}H} NMR (126 MHz, benzene-d_6, 23 °C): δ 188.9 (Ni-$C_{carbene}$), 160.2 (C(O)$_{Boc}$), 146.2 (*ortho*-C_{Dipp}), 139.4 (Ar), 136.5 (*ipso*-C_{Dipp}), 134.9 (Ar), 129.9 (*para*-C_{Dipp}), 129.2 (Ar), 126.7 (Ar), 124.3 (*meta*-C_{Dipp}), 123.7 (N(CH)$_2$N), 119.5 (Ar), 119.2 (Ar), 79.5 (OC(CH$_3$)$_3$), 32.5 (NiCH$_2$$CH_2$), 29.1 ($C$H(CH$_3$)$_2$), 28.6 (C($CH_3$)$_3$), 25.9 (CH($CH_3$)$_2$), 23.4 (CH($CH_3$)$_2$), 5.0 (Ni$CH_2$).

IR (ATR): 2958 (m), 2867 (m), 1526 (vs, ν(CO), Boc carbonyl), 1496 (s), 1454 (s), 1434 (s), 1424 (s), 1363 (m), 1331 (m), 1303 (s), 1254 (s), 1167 (s), 1042 (s), 1024 (s), 940 (w), 800 (m), 744 (s), 730 (s), 703 (m), 658 (m), 448 (m) cm^{-1}.

2.13 Synthesis and characterization of **Ni-10**

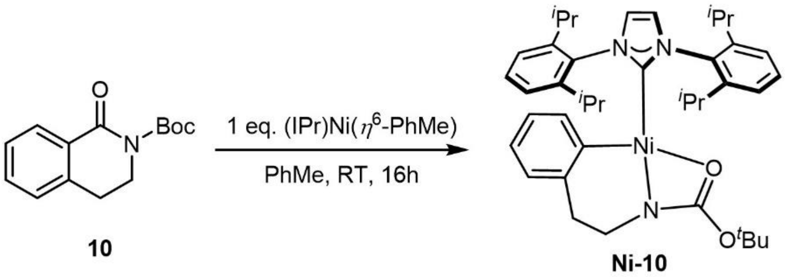

In an argon-filled glovebox, a 150 mL Schlenk flask was charged with (IPr)Ni(η^6-PhMe) (0.2 mmol, 107.9 mg), **10** (0.2 mmol, 49.5 mg), 5 mL toluene and a stir bar. The solution was stirred under room temperature for 16 hours. The volatiles were then removed under vacuum. To the residue was added 2 mL *n*-hexane which afforded a slurry. The content was filtered, and the precipitate was collected, washed with 1 mL *n*-hexane twice and dried under vacuum to give nickel product **Ni-10** (84.0 mg, 0.126 mmol, 63.0%) as an orange powder. Slow evaporation of a concentrated toluene solution of **Ni-10** at room temperature afforded crystals suitable for X-ray diffraction.

^{1}H NMR (500 MHz, benzene-d_6, 23 °C): δ 7.30 – 7.24 (m, 2H, 2×*para*-CH_{Dipp}), 7.23 – 7.18 (m, 4H, 4×*meta*-CH_{Dipp}), 6.92 – 6.83 (m, 2H, Ar-H), 6.82 – 6.74 (m, 1H, Ar-H), 6.64 (s, 2H, N(CH)$_2$N), 6.63 – 6.57 (m, 1H, Ar-H), 3.23 (hept, ^3J = 6.8 Hz, 4H, 4×CH(CH$_3$)$_2$), 2.59 (t, ^3J = 5.6 Hz, 2H, NCH$_2$CH_2), 2.36 (t, ^3J = 5.6 Hz, 2H, NCH_2CH$_2$), 1.40 (d, ^3J = 6.8 Hz, 12H, 2×CH(CH_3)$_2$), 1.30 (s, 9H, C(CH_3)$_3$), 1.05 (d, ^3J = 6.8 Hz, 12H, 2×CH(CH_3)$_2$).

^{13}C{^{1}H} NMR (126 MHz, benzene-d_6, 23 °C): δ 188.2 (Ni-C_{carbene}), 161.4 (C(O)$_{\mathrm{Boc}}$), 146.5 (*ortho*-C_{Dipp}), 144.7 (Ar), 139.9 (Ar), 138.7 (Ar), 136.8 (*ipso*-C_{Dipp}), 129.9 (*para*-C_{Dipp}), 125.8 (Ar), 124.3 (*meta*-C_{Dipp}), 124.2 (N(CH)$_2$N), 124.0 (Ar), 122.9 (Ar), 78.0 (OC(CH$_3$)$_3$), 42.7 (NiCH_2CH$_2$), 37.9 (NiCH$_2$CH_2), 29.2 (CH(CH$_3$)$_2$), 28.9 (C(CH$_3$)$_3$), 26.5 (CH(CH$_3$)$_2$), 23.5 (CH(CH$_3$)$_2$).

IR (ATR): 2964 (m), 2915 (m), 1938 (m), 1558 (vs, ν(CO), Boc carbonyl), 1455 (s), 1424 (vs), 1398 (m), 1363 (m), 1324 (m), 1255 (w), 1202 (w), 1168 (m), 1144 (vs), 1053 (w), 998 (m), 939 (m), 801 (m), 757 (m), 741 (s), 724 (s), 703 (m), 622 (s), 464 (m), 446 (m), 428 (m) cm^{-1}.

2.14 Synthesis and characterization of **Ni-11**

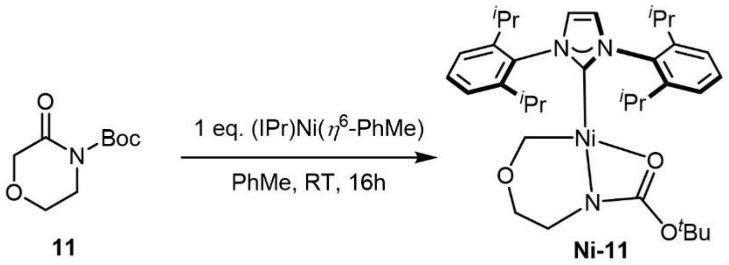

In an argon-filled glovebox, a 150 mL Schlenk flask was charged with (IPr)Ni(η^6-PhMe) (0.2 mmol, 107.9 mg), **11** (0.2 mmol, 40.2 mg), 5 mL toluene and a stir bar. The solution was stirred under room temperature for 16 hours. The volatiles were then removed under vacuum. To the residue was added 2 mL *n*-hexane which afforded a slurry. The content was filtered, and the precipitate was collected and dried under vacuum to give nickel product **Ni-11** (91.2 mg, 0.147 mmol, 73.5%) as a yellow powder. Slow evaporation of a concentrated *n*-hexane solution of **Ni-11** at room temperature afforded crystals suitable for X-ray diffraction.

^{1}H NMR (500 MHz, benzene-d_6, 23 °C): δ 7.33 – 7.27 (m, 2H, 2×*para*-C*H*$_{Dipp}$), 7.24 – 7.21 (m, 4H, 4×*meta*-C*H*$_{Dipp}$), 6.47 (s, 2H, N(C*H*)$_2$N), 3.87 (s, 2H, NiC*H*$_2$), 3.14 – 3.08 (m, 2H, overlapping, NCH$_2$C*H*$_2$), 3.08 (hept, 4H, ^3J = 6.8 Hz, overlapping, 4×C*H*(CH$_3$)$_2$), 2.51 – 2.46 (m, 2H, NC*H*$_2$), 1.70 (d, ^3J = 6.8 Hz, 12H, 2×CH(C*H*$_3$)$_2$), 1.28 (s, 9H, C(C*H*$_3$)$_3$), 1.10 (d, ^3J = 6.8 Hz, 12H, 2×CH(C*H*$_3$)$_2$).

^{13}C{^{1}H} NMR (126 MHz, benzene-d_6, 23 °C): δ 189.2 (Ni-*C*$_{carbene}$), 161.2 (*C*(O)$_{Boc}$), 146.4 (*ortho*-*C*$_{Dipp}$), 136.3 (*ipso*-*C*$_{Dipp}$), 129.9 (*para*-*C*$_{Dipp}$), 124.3 (*meta*-*C*$_{Dipp}$), 123.6 (N(*C*H)$_2$N), 77.2 (O*C*(CH$_3$)$_3$), 71.0 (*N*CH$_2$*C*H$_2$), 61.1 (Ni*C*H$_2$), 35.8 (N*C*H$_2$), 29.2 (*C*H(CH$_3$)$_2$), 28.8 (C(*C*H$_3$)$_3$), 25.8 (CH(*C*H$_3$)$_2$), 23.7 (CH(*C*H$_3$)$_2$).

IR (ATR): 2961 (s), 2931 (w), 2867 (w), 2826 (w), 2782 (w), 1548 (s, ν(CO), Boc carbonyl), 1429 (vs), 1395 (s), 1362 (s), 1327 (vs), 1243 (w), 1152 (vs), 1103 (s), 1067 (s), 1038 (s), 1019 (m), 940 (s), 847 (s), 802 (s), 758 (s), 710 (m), 615 (s), 516 (m), 452 (m), 428 (m) cm^{-1}.

2.15 Synthesis and characterization of **Ni-12**

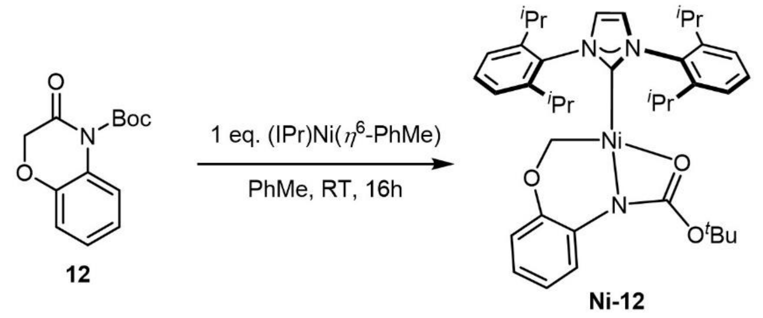

In an argon-filled glovebox, a 150 mL Schlenk flask was charged with (IPr)Ni(η^6-PhMe) (0.2 mmol, 107.9 mg), **12** (0.2 mmol, 49.9 mg), 5 mL toluene and a stir bar. The solution was stirred under room temperature for 16 hours. The volatiles were then removed under vacuum. The orange solid was collected as analytically pure **Ni-12** (131.3 mg, 0.196 mmol, 98.2%). Slow evaporation of a concentrated *n*-hexane solution of **Ni-12** at room temperature afforded crystals suitable for X-ray diffraction.

^{1}H NMR (500 MHz, benzene-d_6, 23 °C): δ 7.67 (dd, $^3J_1$ = 8.0, $^4J_2$ = 1.6 Hz, 1H, Ar-H), 7.28 – 7.22 (m, 2H, 2×*para*-CH_{Dipp}), 7.18 – 7.15 (m, 4H, 4×*meta*-CH_{Dipp}), 6.97 (dd, $^3J_1$ = 7.9 Hz, $^4J_2$ = 1.6 Hz, 1H, Ar-H), 6.83 (ddd, $^3J_1$ = 7.9 Hz, $^3J_2$ = 7.2 Hz, $^4J_3$ = 1.6 Hz, 1H, Ar-H), 6.62 (ddd, $^3J_1$ = 7.9 Hz, $^3J_2$ = 7.2 Hz, $^4J_3$ = 1.6 Hz, 1H, Ar-H), 6.49 (s, 2H, N(C*H*)$_2$N), 4.17 (s, 2H, NiCH_2), 3.08 (hept, ^3J = 6.8 Hz, 4H, 4×C*H*(CH$_3$)$_2$), 1.61 (d, ^3J = 6.8 Hz, 12H, 2×CH(CH_3)$_2$), 1.22 (s, 9H, C(CH_3)$_3$), 1.08 (d, ^3J = 6.8 Hz, 12H, 2×CH(CH_3)$_2$).

^{13}C{^{1}H} NMR (126 MHz, benzene-d_6, 23 °C): δ 187.5 (Ni-C_{carbene}), 160.0 (*C*(O)$_{\text{Boc}}$), 151.1 (Ar), 146.2 (*ortho*-C_{Dipp}), 135.8 (*ipso*-C_{Dipp}), 130.1 (*para*-C_{Dipp}), 129.1 (Ar), 124.4 (*meta*-C_{Dipp}), 123.8 (N(*C*H)$_2$N), 121.2 (Ar), 120.1 (Ar), 119.6 (Ar), 118.3 (Ar), 79.9 (O*C*(CH$_3$)$_3$), 61.5 (Ni*C*H$_2$), 29.2 (*C*H(CH$_3$)$_2$), 28.5 (C(*C*H$_3$)$_3$), 25.8 (CH(*C*H$_3$)$_2$), 23.6 (CH(*C*H$_3$)$_2$).

IR (ATR): 2961 (m), 2925 (w), 2866 (w), 1531 (vs, ν(CO), Boc carbonyl), 1455 (s), 1429 (s), 1404 (m), 1363 (s), 1331 (s), 1312 (s), 1266 (m), 1246 (s), 1163 (m), 1147 (s), 1041 (s), 1020 (s), 953 (s), 803 (s), 735 (s), 705 (s), 651 (s), 546 (w), 527 (w), 454 (m) cm^{-1}.

2.16 Synthesis and characterization of **Ni-13**

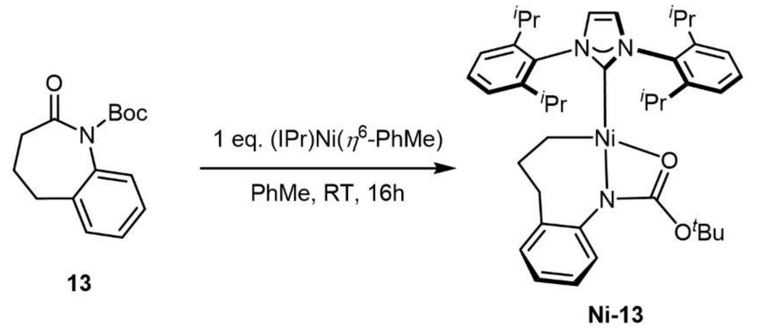

In an argon-filled glovebox, a 150 mL Schlenk flask was charged with (IPr)Ni(η^6-PhMe) (0.2 mmol, 107.9 mg), **13** (0.2 mmol, 52.3 mg), 5 mL toluene and a stir bar. The solution was stirred under room temperature for 16 hours. The volatiles were then removed under vacuum. To the residue was added 2 mL *n*-hexane which afforded a slurry. The content was filtered, and the precipitate was collected, washed with 0.5 mL *n*-hexane twice and dried under vacuum to give nickel product **Ni-13** (105.6 mg, 0.155 mmol, 77.6%) as an orange powder. Slow evaporation of a concentrated toluene solution of **Ni-13** at room temperature afforded crystals suitable for X-ray diffraction.

^{1}H NMR (500 MHz, benzene-d_6, 23 °C): δ 7.44 (dd, $^3J_1$ = 8.0 Hz, $^4J_2$ = 1.3 Hz, 1H, Ar-H), 7.34 – 7.27 (m, 2H, 2×*para*-C*H*$_{Dipp}$), 7.26 – 7.21 (m, 4H, 4×*meta*-C*H*$_{Dipp}$), 7.16 – 7.11 (m, 1H, Ar-H), 6.97 (dd, $^3J_1$ = 7.4 Hz, $^4J_2$ = 1.6 Hz, 1H, Ar-H), 6.85 (td, $^3J_1$ = 7.3 Hz, $^4J_2$ = 1.3 Hz, 1H, Ar-H), 6.52 (s, 2H, N(C*H*)$_2$N), 3.29 (hept, ^3J = 6.8 Hz, 4H, 4×C*H*(CH$_3$)$_2$), 2.86 (t, ^3J = 6.2 Hz, 2H, Ni(CH$_2$)$_2$C*H*$_2$), 1.61 (d, ^3J = 6.8 Hz, 12H, 2×CH(C*H*$_3$)$_2$), 1.18 (s, 9H, C(C*H*$_3$)$_3$), 1.10 (d, ^3J = 6.8 Hz, 12H, 2×CH(C*H*$_3$)$_2$), 0.46 (t, ^3J = 6.6 Hz, 2H, NiC*H*$_2$), 0.18 (app p, ^3J = 6.5 Hz, 2H, NiCH$_2$C*H*$_2$).

^{13}C{^{1}H} NMR (126 MHz, benzene-d_6, 23 °C): δ 187.7 (Ni-*C*$_{carbene}$), 160.0 (*C*(O)$_{Boc}$), 146.2 (*ortho*-*C*$_{Dipp}$), 142.3 (Ar), 136.6 (*ipso*-*C*$_{Dipp}$), 135.3 (Ar), 129.7 (*para*-*C*$_{Dipp}$), 129.3 (Ar), 126.3 (Ar), 124.4 (*meta*-*C*$_{Dipp}$), 123.5 (N(*C*H)$_2$N), 122.9 (Ar), 121.4 (Ar), 78.6 (O*C*(CH$_3$)$_3$), 34.8 (Ni(CH$_2$)$_2$*C*H$_2$), 29.3 (NiCH$_2$*C*H$_2$), 29.1 (*C*H(CH$_3$)$_2$), 28.5 (C(*C*H$_3$)$_3$), 25.7 (CH(*C*H$_3$)$_2$), 23.5 (CH(*C*H$_3$)$_2$), 2.7 (Ni*C*H$_2$).

IR (ATR): 2962 (m), 2916 (m), 2866 (w), 2812 (w), 1525 (vs, ν(CO), Boc carbonyl), 1494 (m), 1450 (m), 1431 (s), 1395 (m), 1363 (m), 1330 (m), 1297 (m), 1264 (m), 1170 (s), 1042 (m), 1021 (m), 939 (w), 836 (w), 800 (m), 754 (s), 737 (m), 705 (m), 659 (m), 599 (w), 490 (w), 451 (w), 418 (w) cm^{-1}.

2.17 Synthesis and characterization of **Ni-14**

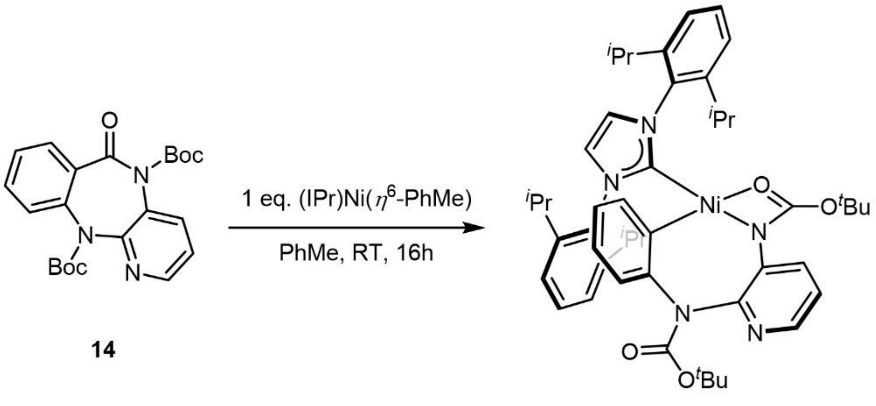

14 **Ni-14**

In an argon-filled glovebox, a 150 mL Schlenk flask was charged with (IPr)Ni(η^6-PhMe) (0.2 mmol, 107.9 mg), **14** (0.2 mmol, 82.3 mg), 5 mL toluene and a stir bar. The solution was stirred under room temperature for 16 hours. The volatiles were then removed under vacuum. To the residue was added 3 mL *n*-hexane which afforded a slurry. The content was filtered, and the precipitate was collected, washed with 1 mL *n*-hexane twice and dried under vacuum to give nickel product **Ni-14** (137.5 mg, 0.166 mmol, 82.8%) as an orange powder. Slow evaporation of a concentrated toluene solution of **Ni-14** at room temperature afforded crystals suitable for X-ray diffraction.

^{1}H NMR (500 MHz, benzene-d_6, 23 °C): δ 7.98 (dd, $^3J_1$ = 4.6 Hz, $^4J_2$ = 1.8 Hz, 1H, Ar-H), 7.45 (dd, $^3J_1$ = 8.1 Hz, $^4J_2$ = 1.8 Hz, 1H, Ar-H), 7.42 (br s, 4H, 4×*meta*-CH_{Dipp}), 7.36 (dd, $^3J_1$ = 7.6 Hz, $^4J_2$ = 1.6 Hz, 1H, Ar-H), 7.12 (dd, $^3J_1$ = 6.8 Hz, $^4J_2$ = 2.5 Hz, 2H, 2×*para*-CH_{Dipp}), 6.87 (dd, $^3J_1$ = 7.6 Hz, $^4J_2$ = 1.6 Hz, 1H, Ar-H), 6.82 (td, $^3J_1$ = 7.4 Hz, $^4J_2$ = 1.6 Hz, 1H, Ar-H), 6.73 (td, $^3J_1$ = 7.4 Hz, $^4J_2$ = 1.6 Hz, 1H, Ar-H), 6.60 – 6.56 (dd, $^3J_1$ = 8.1 Hz, $^3J_2$ = 4.6 Hz, 1H, Ar-H), 6.55 (s, 2H, N(CH)$_2$N), 4.27 (br s, 2H, 2×CH(CH$_3$)$_2$), 2.23 (br s, 2H, 2×CH(CH$_3$)$_2$), 2.11 (br s, 6H, CH(CH_3)$_2$), 1.55 (s, 9H, C(CH_3)$_3$), 1.23 (d, ^3J = 7.2 Hz, 6H, CH(CH_3)$_2$), 1.03 (d, ^3J = 6.8 Hz, 6H, CH(CH_3)$_2$), 0.99 (s, 9H, overlapping, C(CH_3)$_3$), 0.95 (br s, 6H, overlapping, CH(CH_3)$_2$).

^{13}C{^{1}H} NMR (126 MHz, benzene-d_6, 23 °C): δ 181.0 (Ni-C_{carbene}), 161.7 (C(O)$_{\mathrm{Boc}}$), 154.8 (C(O)$_{\mathrm{Boc}}$), 148.7 (Ar), 147.5 (Ar), 146.0 (Ar), 141.2 (Ar), 140.1 (Ar), 138.3 (Ar), 136.1 (Ar), 134.1 (Ar), 130.5 (Ar), 130.2 (Ar), 129.5 (Ar), 125.2 (Ar), 125.1 (Ar), 124.9 (Ar), 124.8 (Ar), 124.7 (Ar), 121.8 (Ar), 80.5 (OC(CH$_3$)$_3$), 78.2 (OC(CH$_3$)$_3$), 29.6 (CH(CH$_3$)$_2$), 29.0 (C(CH$_3$)$_3$), 28.8 (CH(CH$_3$)$_2$), 28.2 (C(CH$_3$)$_3$), 26.6 (CH(CH$_3$)$_2$), 25.9 (CH(CH$_3$)$_2$), 25.0 (CH(CH$_3$)$_2$), 23.8 (CH(CH$_3$)$_2$).

IR (ATR): 2960 (m), 2928 (w), 2869 (w), 1706 (vs, ν(CO), uncoordinated Boc carbonyl), 1566 (w), 1507 (vs, ν(CO), coordinated Boc carbonyl), 1479 (m), 1446 (m), 1414 (s), 1393 (w), 1345 (s), 1307 (s), 1244 (m), 1165 (s), 1077 (m), 1043 (s), 1022 (s), 972 (w), 934 (m), 798 (s), 754 (s), 725 (s), 706 (m), 687 (w), 641 (w), 552 (w), 453 (w)cm^{-1}.

2.18 Synthesis and characterization of **Ni-15**

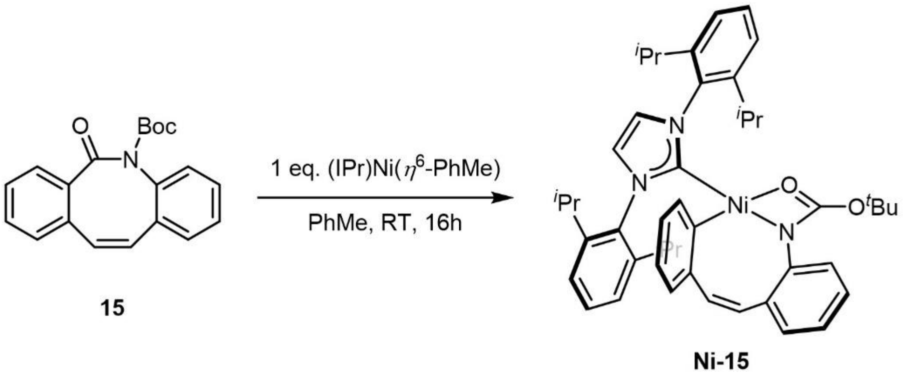

In an argon-filled glovebox, a 150 mL Schlenk flask was charged with (IPr)Ni(η^6-PhMe) (0.2 mmol, 107.9 mg), **15** (0.2 mmol, 64.3 mg), 5 mL toluene and a stir bar. The solution was stirred under room temperature for 16 hours. The volatiles were then removed under vacuum. To the residue was added 2 mL *n*-hexane which afforded a slurry. The content was filtered, and the precipitate was collected, washed with 0.5 mL *n*-hexane twice and dried under vacuum to give nickel product **Ni-15** (124.0 mg, 0.167 mmol, 83.7%) as an orange powder. Slow evaporation of a concentrated *n*-hexane solution of **Ni-15** at room temperature afforded crystals suitable for X-ray diffraction.

^{1}H NMR (500 MHz, benzene-d_6, 23 °C): δ 7.42 (dd, $^3J_1$ = 7.6 Hz, $^4J_2$ = 1.2 Hz, overlapping, 1H, Ar-H), 7.41 (dd, $^3J_1$ = 7.8 Hz, $^4J_2$ = 1.5 Hz, overlapping, 2H, 2×*meta*-CH_{Dipp}), 7.32 (app t, J = 7.8 Hz, 2H, 2×*meta*-CH_{Dipp}), 7.06 (dd, $^3J_1$ = 7.8 Hz, $^4J_2$ = 1.5 Hz, 2H, *para*-CH_{Dipp}), 6.94 – 6.90 (m, 1H, Ar-H), 6.82 – 6.78 (m, 1H, Ar-H), 6.75 – 6.71 (m, overlapping, 1H, Ar-H), 6.71 – 6.70 (m, overlapping 1H, Ar-H), 6.70 – 6.67 (m, overlapping, 1H, Ar-H), 6.56 – 6.52 (m, overlapping, 1H, Ar-H), 6.53 (d, overlapping, ^3J = 11.0 Hz, 1H, olefinic C–H), 6.50 (s, 2H, N(CH)$_2$N), 6.47 – 6.43 (m, 1H, Ar-H), 6.10 (d, ^3J = 11.0 Hz, 1H, olefinic C–H), 4.17 (hept, ^3J = 6.8 Hz, 2H, 2×CH(CH$_3$)$_2$), 2.20 (d, ^3J = 6.8 Hz, 6H, CH(CH_3)$_2$), 2.15 (hept, ^3J = 6.8 Hz, 2H, 2×CH(CH$_3$)$_2$), 1.19 (d, ^3J = 6.8 Hz, 6H, CH(CH_3)$_2$), 1.07 (s, 9H, CH(CH_3)$_2$), 0.96 (d, ^3J = 6.8 Hz, 6H, CH(CH_3)$_2$), 0.89 (d, ^3J = 6.8 Hz, 6H, CH(CH_3)$_2$).

^{13}C{^{1}H} NMR (126 MHz, benzene-d_6, 23 °C): δ 182.0 (Ni-C_{carbene}), 160.6 (C(O)$_{\text{Boc}}$), 148.9 (Ar), 146.8 (Ar), 146.3 (Ar), 145.8 (Ar), 141.1 (Ar), 140.1 (olefinic), 137.3 (Ar), 137.0 (Ar), 134.7 (Ar), 129.8 (Ar), 127.7 (olefinic), 127.3 (Ar), 126.2 (Ar), 125.2 (Ar), 124.7 (Ar), 124.6 (Ar), 124.3 (Ar), 123.4 (Ar), 122.3 (Ar), 78.5 (OC(CH$_3$)$_3$), 29.6 (CH(CH$_3$)$_2$), 28.8 (CH(CH$_3$)$_2$), 28.6 (OC(CH$_3$)$_3$), 26.6 (CH(CH$_3$)$_2$), 26.1 (CH(CH$_3$)$_2$), 24.1 (CH(CH$_3$)$_2$), 23.1 (CH(CH$_3$)$_2$).

IR (ATR): 2959 (m), 2926 (m), 2868 (m), 1568 (w), 1524 (vs, ν(CO), Boc carbonyl), 1452 (s), 1427 (vs), 1394 (m), 1366 (s), 1325 (s), 1287 (s), 1247 (s), 1171 (s), 1107 (s), 1044 (s), 1021 (s), 938 (s), 833 (w), 804 (w), 757 (s), 731 (vs), 702 (s), 651 (m), 551 (m), 490 (s), 454 (m), 438 (m), 424 (m) cm^{-1}.

2.19 Synthesis and characterization of (IPr)Ni$(CO)_3$

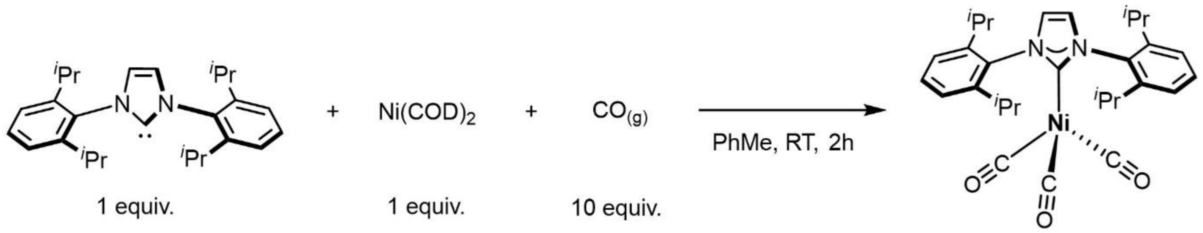

In an argon-filled glovebox, a COware was charged with IPr (0.074 mmol, 28.6 mg), $NiCOD_2$ (0.073 mmol, 20.1 mg), 0.5 mL toluene, stir bars and sealed. The vessel was brought out and attached to N_2 flow via a needle. The other chamber was charged with methanesulfonyl chloride (0.73 mmol, 56.5 uL), formic acid (0.73 mmol, 27.5 uL) and 2 mL anhydrous toluene, followed by addition of NEt_3 (1.46 mmol, 0.2 mL). The $CO_{(g)}$ was released[9] evidenced by vigorous bubbling and decolorization of the nickel solution. The nickel solution was allowed to stir under RT for 2 hours and transferred to a 10 mL Schlenk flask via syringe. The volatiles were removed under vacuum affording the title compound as an off-white solid (0.059 mmol, 31.4 mg, 81%). The ^{1}H NMR resonances slightly deviate from those reported.[10]

^{1}H NMR (500 MHz, benzene-d_6, 23 °C): δ 7.28 (ddt, J = 8.3, 7.2, 0.5 Hz, 2H), 7.16 – 7.14 (m, 4H), 6.62 (s, 2H), 2.78 (hept, J = 6.9 Hz, 4H), 1.36 (d, J = 6.9 Hz, 12H), 1.05 (d, J = 6.9 Hz, 12H).

^{13}C{^{1}H} NMR (126 MHz, benzene-d_6, 23 °C): δ 197.8, 197.4, 146.1, 137.8, 130.2, 124.3, 123.4, 28.8, 25.5, 22.8.

^{1}H NMR (500 MHz, toluene-d_8, 23 °C): δ 7.25 (ddt, J = 8.3, 7.3, 0.5 Hz, 2H), 7.12 (d, J = 7.8 Hz, 4H), 6.64 (s, 2H), 2.75 (hept, J = 6.9 Hz, 4H), 1.34 (d, J = 6.9 Hz, 13H), 1.05 (d, J = 6.9 Hz, 12H).

^{13}C{^{1}H} NMR (126 MHz, toluene-d_8, 23 °C): δ 197.7, 197.6, 146.0, 137.5, 130.2, 124.2, 123.4, 28.7, 25.4, 22.7.

2.20 Synthesis and characterization of (IPr)Ni(η^2-norbornene$)_2$

The title complex was synthesized according to a literature reported procedure and the ^{1}H NMR data are in accordance with those reported.[11]

2.21 Synthesis and characterization of $(IPr)_2$Ni

The title complex was synthesized according to a literature reported procedure and the ^{1}H NMR data are in accordance with those reported.[12]

3. Synthesis and characterization of organic compounds

3.1 Synthesis and characterization of organic substrates

General procedure for the preparation of *N*–Boc lactam substrates

To a 250 mL oven-dried Schlenk flask was added the free lactam (1 eq.), 4-dimethylaminopyridine (DMAP) (0.1 eq.), a stir bar and sealed. The flask was evacuated on the Schlenk line and refilled with N_2, followed by another two cycles. Anhydrous CH_2Cl_2 (0.25 M) and Et_3N (1 eq.) were added by a syringe and all solids were dissolved upon stirring. The solution was cooled to 0 °C with an ice bath. To the flask a solution of Boc_2O (1.2 eq.) in anhydrous CH_2Cl_2 (1 M) was added by a syringe. The reaction mixture was allowed to warm up and stirred at room temperature under N_2 for 16 hours. The reaction was quenched by adding saturated NH_4Cl(aq) solution (10 mL). The organic layer was separated. The aqueous layer was extracted with CH_2Cl_2 (3 × 5 mL). The combined organic layer was dried with Na_2SO_4(s). The product was purified by silica gel chromatography and dried under high vacuum prior to use.

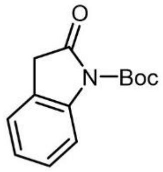

***N*–Boc 2-oxindole (3):** Prepared from 2-oxindole (0.50 g, 3.76 mmol, 1 eq.), DMAP (0.12 g, 0.934 mmol, 0.25 eq.), Et_3N (1.3 mL, 9.4 mmol, 2.5 eq.), Boc_2O (0.86 g, 3.94 mmol, 1.05 eq.) according to the general procedure. Purification by silica gel column chromatography (hexanes : ethyl acetate = 7 : 3) afforded the analytically pure product (0.84 g, 3.61 mmol, 96%) as a white solid. The NMR data are in accordance with those reported.[13]

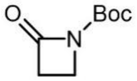

***N*–Boc 2-azetidinone** (**4**): Prepared from azetidin-2-one (0.21 g, 3.0 mmol, 1.0 eq.), DMAP (37 mg, 0.3 mmol, 0.1 eq.), Et_3N (0.42 mL, 3 mmol, 1.0 eq.), Boc_2O (0.79 g, 3.6 mmol, 1.2 eq.) according to the general procedure. Purification by silica gel column chromatography (hexanes : ethyl acetate = 9 : 1) afforded the analytically pure product (0.44 g, 2.58 mmol, 86%) as a colorless oil. The NMR data are in accordance with those reported.[14]

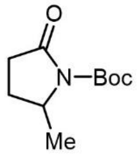

***N*–Boc 5-methyl-2-pyrrolidinone** (**5**): Prepared from 5-methylpyrrolidin-2-one (0.21 g, 3.0 mmol, 1.0 eq.), DMAP (37 mg, 0.3 mmol, 0.1 eq.), Et_3N (0.42 mL, 3 mmol, 1.0 eq.), Boc_2O (0.79 g, 3.6 mmol, 1.2 eq.) according to the general procedure. Purification by silica gel column chromatography (hexanes : ethyl acetate = 9 : 1) afforded the analytically pure product (0.59 g, 2.94 mmol, 98%) as a colorless oil. The NMR data are in accordance with those reported.[15]

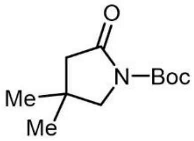

***N*–Boc 4,4-dimethyl-2-pyrrolidinone** (**6**): Prepared from 4,4-dimethylpyrrolidin-2-one (0.34 g, 3.0 mmol, 1.0 eq.), DMAP (37 mg, 0.3 mmol, 0.1 eq.), Et_3N (0.42 mL, 3 mmol, 1.0 eq.), Boc_2O (0.79 g, 3.6 mmol, 1.2 eq.) according to the general procedure. Purification by silica gel column chromatography (hexanes : ethyl acetate = 9 : 1) afforded the analytically pure product (0.58 g, 2.73 mmol, 91%) as a white solid.

^{1}H NMR (500 MHz, chloroform-*d*, 23 °C) δ 3.44 (s, 2H), 2.32 (s, 2H), 1.52 (s, 9H), 1.16 (s, 6H).

^{13}C{^{1}H} NMR (126 MHz, chloroform-*d*, 23 °C) δ 173.8, 150.4, 82.9, 59.2, 47.9, 31.6, 28.2, 27.3.

HRMS (ESI): [M+H] calcd.: 214.1438; found: 214.1441.

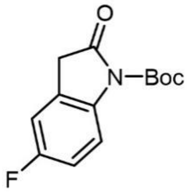

***N*–Boc-5-fluoro-2-oxindole** (**7**): Prepared from 5-fluoroindolin-2-one (0.57 g, 3.76 mmol, 1 eq.), DMAP (0.12 g, 0.934 mmol, 0.25 eq.), Et_3N (1.3 mL, 9.4 mmol, 2.5 eq.), Boc_2O (0.86 g, 3.94 mmol, 1.05 eq.) according to the general procedure. Purification by silica gel column chromatography (hexanes : ethyl acetate = 5 : 5) afforded the analytically pure product (0.83 g, 3.31 mmol, 88%) as a white solid. The NMR data are in accordance with those reported.[16]

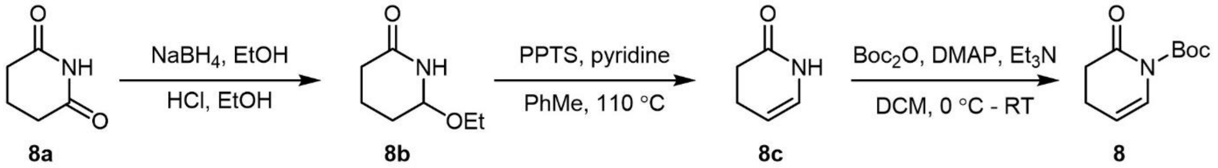

***N*–Boc-3,4-dihydropyridin-2(1*H*)-one** (**8**): The free lactam 3,4-dihydropyridin-2(1*H*)-one (**8c**) was prepared according to a literature reported method.[17] The title compound was prepared from 3,4-dihydropyridin-2(1*H*)-one (0.29 g, 3.0 mmol, 1 eq), DMAP (37 mg, 0.3 mmol, 0.1 eq.), Et_3N (0.42 mL, 3 mmol, 1.0 eq.), Boc_2O (0.79 g, 3.6 mmol, 1.2 eq.) according to the general procedure. Purification by silica gel column chromatography (hexanes : ethyl acetate = 7 : 3) afforded the analytically pure product (0.56 g, 2.85 mmol, 95%) as a colorless oil. The NMR data are in accordance with those reported.[17]

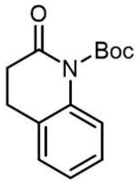

***N*–Boc-3,4-dihydroquinolin-2(1*H*)-one** (**9**): Prepared from 3,4-dihydroquinolin-2(1*H*)-one (0.44 g, 3.0 mmol, 1.0 eq.), DMAP (37 mg, 0.3 mmol, 0.1 eq.), Et_3N (0.42 mL, 3 mmol, 1.0 eq.), Boc_2O (0.79 g, 3.6 mmol, 1.2 eq.) according to the general procedure. Purification by silica gel column chromatography (hexanes : ethyl acetate = 7 : 3) afforded the analytically pure product (0.72 g, 2.91 mmol, 97%) as a white solid. The NMR data are in accordance with those reported.[18]

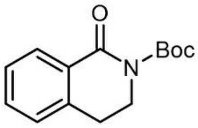

***N*–Boc-3,4-dihydroisoquinolin-1(2*H*)-one** (**10**): Prepared from 3,4-dihydroisoquinolin-1(2*H*)-one (0.44 g, 3.0 mmol, 1 eq.), DMAP (37 mg, 0.3 mmol, 0.1 eq.), Et_3N (0.42 mL, 3 mmol, 1.0 eq.), Boc_2O (0.79 g, 3.6 mmol, 1.2 eq.) according to the general procedure. Purification by silica gel column chromatography (hexanes : ethyl acetate = 7 : 3) afforded the analytically pure product (0.67 g, 2.70 mmol, 90%) as a white solid. The NMR data are in accordance with those reported.[19]

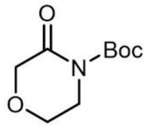

***N*–Boc-3-morpholinone** (**11**): Prepared from 3-morpholinone (0.30 g, 3.0 mmol, 1 eq.), DMAP (37 mg, 0.3 mmol, 0.1 eq.), Et_3N (0.42 mL, 3 mmol, 1.0 eq.), Boc_2O (0.79 g, 3.6 mmol, 1.2 eq.) according to the general procedure. Purification by silica gel column chromatography (hexanes : ethyl acetate = 7 : 3) afforded the analytically pure product (0.57 g, 2.85 mmol, 95%) as a white solid. The NMR data are in accordance with those reported.[20]

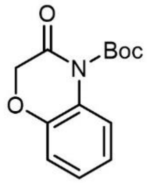

***N*–Boc-2*H*-benzo[*b*][1,4]oxazin-3(4*H*)-one** (**12**): Prepared from 2*H*-benzo[*b*][1,4]oxazin-3(4*H*)-one (0.45 g, 3.0 mmol, 1 eq.), DMAP (37 mg, 0.3 mmol, 0.1 eq.), Et_3N (0.42 mL, 3 mmol, 1.0 eq.), Boc_2O (0.79 g, 3.6 mmol, 1.2 eq.) according to the general procedure. Purification by silica gel column chromatography (hexanes : ethyl acetate = 7 : 3) afforded the analytically pure product (0.73 g, 2.91 mmol, 97%) as a colorless oil. The NMR data are in accordance with those reported.[21]

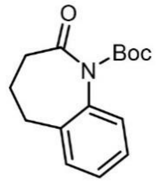

***N*–Boc-2,3,4,5-tetrahydro-1*H*-1-benzo[*b*]azepin-2-one** (**13**): Prepared from 2,3,4,5-tetrahydro-1*H*-1-benzo[*b*]azepin-2-one (0.48 g, 3.0 mmol, 1 eq.), DMAP (37 mg, 0.3 mmol, 0.1 eq.), Et_3N (0.42 mL, 3 mmol, 1.0 eq.), Boc_2O (0.79 g, 3.6 mmol, 1.2 eq.) according to the general procedure. Purification by silica gel column chromatography (hexanes : ethyl acetate = 7 : 3) afforded the analytically pure product (0.69 g, 2.64 mmol, 88%) as a colorless oil. The NMR data are in accordance with those reported.[22]

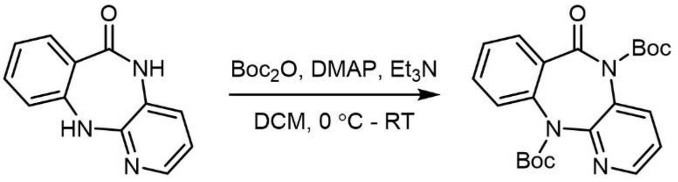

***N*,*N*-Di-Boc-5,11-dihydro-6*H*-benzo[*e*]pyrido[3,2-b][1,4]diazepin-6-one** (**14**): Prepared from 5,11-dihydro-6*H*-benzo[*e*]pyrido[3,2-b][1,4]diazepin-6-one (0.48 g, 1.5 mmol, 1 eq.), DMAP (37 mg, 0.3 mmol, 0.2 eq.), Et_3N (0.42 mL, 3 mmol, 2.0 eq.), Boc_2O (0.79 g, 3.6 mmol, 2.4 eq.) according to the general procedure. Purification by silica gel column chromatography (hexanes : ethyl acetate = 3 : 7) afforded the analytically pure product (0.52 g, 1.28 mmol, 85%) as a white solid.

^{1}H NMR (500 MHz, chloroform-*d*, 23 °C) δ 8.43 (dd, *J* = 4.7, 1.7 Hz, 1H), 8.00 (dd, *J* = 7.8, 1.6 Hz, 1H), 7.94 (dd, *J* = 8.1, 1.7 Hz, 1H), 7.65 – 7.59 (m, 1H), 7.55 (ddd, *J* = 8.1, 7.3, 1.7 Hz, 1H), 7.37 – 7.32 (m, 1H), 7.30 (dd, *J* = 8.1, 4.7 Hz, 1H), 1.51 (s, 9H), 1.44 (s, 9H).

^{13}C{^{1}H} NMR (126 MHz, chloroform-*d*, 23 °C) δ 165.1, 152.2, 151.7, 151.3, 148.0, 142.2, 137.8, 133.9, 132.7, 130.5, 128.4, 128.3, 127.7, 123.2, 84.8, 82.5, 28.3, 27.9.

HRMS (ESI): [M+H] calcd.: 412.1867; found: 412.1866.

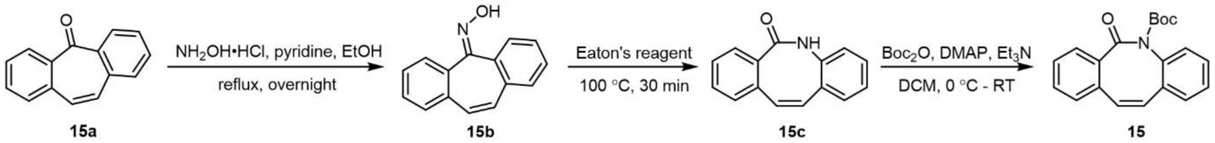

***N*–Boc-dibenzo[*b*,*f*]azocin-6(5*H*)-one** (**15**): The free lactam dibenzo[*b*,*f*]azocin-6(5*H*)-one (**15c**) was prepared according to a literature reported method.[23] The title compound was prepared from **15c** (0.32 g, 1.5 mmol, 1 eq.), DMAP (18 mg, 0.15 mmol, 0.1 eq.), Et_3N (0.21 mL, 1.5 mmol, 1.0 eq.), Boc_2O (0.39 g, 1.8 mmol, 1.2 eq.) according to the general procedure. Purification by silica gel column chromatography (hexanes : ethyl acetate = 7 : 3) afforded the analytically pure product (0.48 g, 1.37 mmol, 91%) as a white solid.

^{1}H NMR (500 MHz, chloroform-*d*, 23 °C) δ 7.60 – 7.55 (m, 1H), 7.31 – 7.23 (m, 2H), 7.25 – 7.17 (m, 3H), 7.17 – 7.11 (m, 1H), 7.05 – 7.00 (m, 1H), 6.96 (br s, 2H), 1.41 (s, 9H).

^{13}C{^{1}H} NMR (126 MHz, chloroform-*d*, 23 °C) δ 171.0, 151.4, 137.6, 136.2, 135.9, 134.2, 132.6, 130.4, 129.3, 129.2, 129.2, 128.4, 128.3, 128.1, 128.1, 127.8, 83.3, 28.0.

HRMS (ESI): [M+Na] calcd.: 344.1257; found: 344.1254.

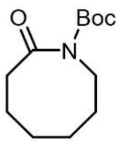

***N*–Boc-2-azocanone** (**17**): Prepared from 2-azocanone (0.38 g, 3.0 mmol, 1 eq.), DMAP (37 mg, 0.3 mmol, 0.1 eq.), Et_3N (0.42 mL, 3 mmol, 1.0 eq.), Boc_2O (0.79 g, 3.6 mmol, 1.2 eq.) according to the general procedure. Purification by silica gel column chromatography (hexanes : ethyl acetate = 9 : 1) afforded the analytically pure product (0.65 g, 2.88 mmol, 96%) as a colorless oil. The NMR data are in accordance with those reported.[24]

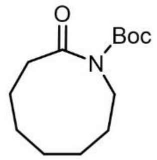

***N*–Boc-2-azonanone** (**18**): Prepared from 2-azonanone (0.42 g, 3.0 mmol, 1 eq.), DMAP (37 mg, 0.3 mmol, 0.1 eq.), Et_3N (0.42 mL, 3 mmol, 1.0 eq.), Boc_2O (0.79 g, 3.6 mmol, 1.2 eq.) according to the general procedure. Purification by silica gel column chromatography (hexanes : ethyl acetate = 9 : 1) afforded the analytically pure product (0.64 g, 2.67 mmol, 89%) as a colorless oil. The NMR data are in accordance with those reported.[25]

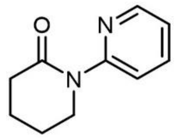

***N*–2-pyridyl-piperidin-2-one (2-py)**: To an oven-dried 100 mL two-neck round bottom flaks was added CuI (59 mg, 0.31 mmol, 0.02 eq.), K_2CO_3 (4.16 g, 30.1 mmol, 2 eq.) and a stir bar. The reaction vessel was sealed, evacuated and refilled with N_2 (x3), then piperidin-2-one (1.54 g, 15.5 mmol, 1 eq.), *N*,*N*'-dimethylethylenediamine (180 mg, 1.55 mmol, 0.1 eq.), 2-bromopyridine (2.45 g, 15.5 mmol, 1 eq.) and toluene (20 mL) were added via syringe. The reaction mixture was refluxed for 24 h, cooled and filtered, and the volatiles were removed in vacuo. The solid residue was purified by silica gel column chromatography (dichloromethane : methanol = 95:5) to yield the analytically pure product (2.43 g, 13.8 mmol, 89%) as a colorless oil. The NMR data are in accordance with those reported.[26]

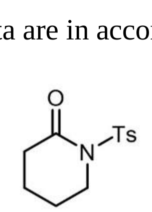

***N*–Ts-piperidin-2-one (2-Ts)**: A 100 mL three-necked flask was charged with a solution of piperidin-2-one (0.475 g, 4.8 mmol, 1 eq.) in anhydrous THF (6 mL) and a stir bar under N_2. The mixture was cooled to -78 °C. A solution of *n*-BuLi in *n*-hexane (1.6 M, 1.05 eq., 5.04 mmol, 3.15 mL) was dropwise added at -78°C. The mixture was stirred for 1 h. A solution of *p*-toluenesulfonyl chloride (5.04 mmol,

1.05 eq. 0.960 g) in anhydrous THF (5.2 mL) was added dropwise at -78 °C. The mixture was allowed to warm up to room temperature and stirred for 90 min. The mixture was quenched with sat. aq. NH_4Cl (20 mL) and the aqueous phase was extracted with EtOAc (3 x 10 ml). The organic layers were combined and washed with brine, dried over Mg_2SO4, filtered and concentrated. The crude mixture was purified by flash column chromatography (hexanes : ethyl acetate = 9:1 - 7:3) to yield the product (0.248 g, 0.98 mmol, 20%) as a white solid. The NMR data are in accordance with those reported.[27]

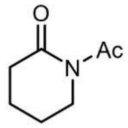

***N*–Ac-piperidin-2-one (2-Ac)**: An oven dried 100 mL three-necked flask was charged with piperidin-2-one (0.500 g, 5.04 mmol, 1.1 eq.), toluene (14.4 mL) and a stir bar. A solution of acetyl chloride (0.360 g, 4.58 mmol, 0.33 mL, 1 eq.) in toluene (7.2 mL) was added dropwise at room temperature. The mixture was stirred under reflux for 17 hours. The mixture was cooled to room temperature. EtOAc (21 mL) was added to the mixture and the organic layer was washed with aq. sat. NH_4Cl (2 x 36 mL) and followed by water (2 x 50 mL). The organic layer was dried over Na_2SO_4, filtered and concentrated. The crude product was purified by flash column chromatography (hexanes : ethyl acetate = 5:5) to yield the product as a colorless oil (0.171 g, 1.21 mmol, 26%). The NMR data are in accordance with those reported.[28]

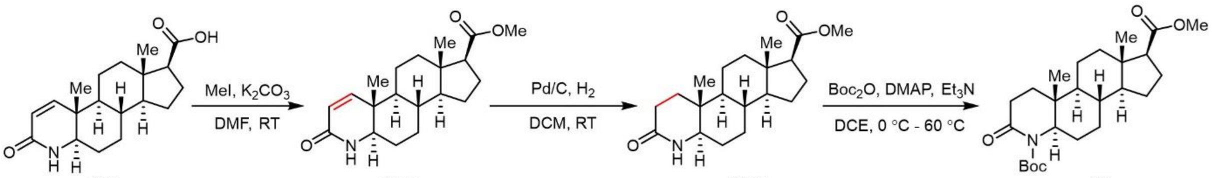

***N*–Boc-3-oxo-4-aza-5α-androstane-17β-methyl carboxylate** (**19**): prepared from 3-Oxo-4-aza-5α-androst-1-ene-17β-carboxylic acid (**19-I**) as shown above.

Methylation: into a round bottom flask was added **19-I** (0.95 g, 3 mmol, 1 eq), anhydrous K_2CO_3 (0.83 g, 6 mmol, 2 eq.) and a stir bar. The flask was attached to the Schlenk line and evacuated, followed by refilling with N_2. Anhydrous DMF (10 mL) and MeI (0.28 mL, 4.5 mmol, 1.5 eq.) were added via syringe. The reaction was stirred under N_2 at RT for 16 hours and was quenched by adding 10% NaOH (aq) (2 mL). The reaction was filtered, and the DMF solvent and water were removed under vacuum. The solid residue was purified by silica gel column chromatography (dichloromethane : methanol = 9 : 1) to afford analytically pure **19-II** (0.96 g, 2.9 mmol, 96%).

Hydrogenation: A J-Young flask was charged with **19-II** (497 mg, 1.5 mmol, 1 eq.), Pd/C (10% Pd, 50 mg), anhydrous DCM (10 mL), a stir bar and sealed. The flask was attached to the Schlenk line, the solution was frozen in liquid nitrogen and the headspace was evacuated. One atmosphere of H_2 was introduced. The reaction was allowed to warm to room temperature and stir for 16 hours. The residual

H_2 was released, and the reaction was filtered through Celite. The filtrate was dried to afford analytically pure **19-III** (495 mg, 1.49 mmol, 99%).

Boc protection: To a 250 mL oven-dried Schlenk flask was added **19-III** (333 mg, 1 mmol, 1 eq.), DMAP (122 mg, 1 mmol, 1 eq.), a stir bar and sealed. The flask was evacuated on the Schlenk line and refilled with N_2, followed by another two cycles. Anhydrous dichloroethane (5 mL) and Et_3N (3.5 mL, 25 mmol, 25 eq.) were added via syringe. The solution was cooled to 0 °C with an ice bath. To the flask a solution of Boc_2O (2.2 g, 10 mmol, 10 eq.) in anhydrous dichloroethane (5 mL) was added via syringe. The reaction mixture was allowed to warm up and stirred at 60 °C with a reflux condenser under N_2 for 16 hours. The reaction was quenched by adding saturated NH_4Cl(aq) solution (10 mL). The organic layer was separated. The aqueous layer was extracted with CH_2Cl_2 (3 × 5 mL). The combined organic layer was dried with Na_2SO_4(s). The product was purified by silica gel chromatography (dichloromethane : methanol = 95 : 5) to afford analytically pure **19** (395 mg, 0.91 mmol, 91%).

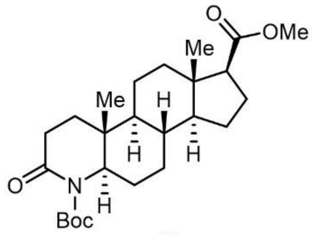

19

^{1}H NMR (500 MHz, chloroform-*d*, 23 °C) δ 3.67 (s, 3H), 3.34 (dd, *J* = 12.1, 3.3 Hz, 1H), 2.53 – 2.40 (m, 2H), 2.35 (t, *J* = 9.4 Hz, 1H), 2.13 (dddd, *J* = 14.1, 11.7, 9.2, 2.9 Hz, 1H), 2.01 (dt, *J* = 12.4, 2.9 Hz, 1H), 1.88 (ddd, *J* = 13.3, 6.6, 2.8 Hz, 1H), 1.84 – 1.64 (m, 4H), 1.62 – 1.57 (m, 1H), 1.52 (s, 9H), 1.50 – 1.21 (m, 6H), 1.11 (ddd, *J* = 12.6, 10.8, 7.1 Hz, 1H), 1.05 – 0.94 (m, 1H, overlapping), 0.98 (s, 3H, overlapping) 0.91 – 0.78 (m, 1H), 0.67 (s, 3H).

^{13}C{^{1}H} NMR (126 MHz, chloroform-*d*, 23 °C) δ 174.5, 170.5, 154.2, 84.0, 64.8, 55.4, 55.3, 51.6, 51.4, 44.3, 38.3, 35.8, 35.0, 33.1, 29.7, 29.5, 27.8, 25.0, 24.4, 23.8, 21.2, 13.7, 12.5.

HRMS (ESI): [M+Na] calcd.: 456.2720; found: 456.2711.

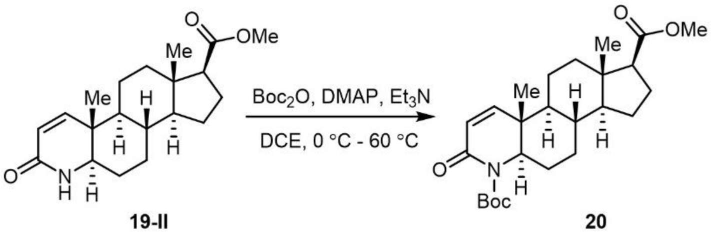

***N*–Boc-3-Oxo-4-aza-5α-androst-1-ene-17β-methyl carboxylate** (**20**): prepared from the free amide **19-II** according to a modified procedure. To a 250 mL oven-dried Schlenk flask was added **19-II** (265 mg, 0.8 mmol, 1 eq.), DMAP (98 mg, 0.8 mmol, 1 eq.), a stir bar and sealed. The flask was evacuated on the Schlenk line and refilled with N_2, followed by another two cycles. Anhydrous dichloroethane (5 mL) and Et_3N (2.8 mL, 20 mmol, 25 eq.) were added via syringe. The solution was cooled to 0 °C with an ice bath. To the flask a solution of Boc_2O (1.8 g, 8 mmol, 10 eq.) in anhydrous dichloroethane (5 mL) was added via syringe. The reaction mixture was allowed to warm up and stirred at 60 °C with a reflux condenser under N_2 for 16 hours. The reaction was quenched by adding saturated NH_4Cl(aq) solution (10 mL). The organic layer was separated. The aqueous layer was extracted with CH_2Cl_2 (3 × 5 mL). The combined organic layer was dried with Na_2SO_4(s). The product was purified by silica gel chromatography (dichloromethane : methanol = 95 : 5) to afford analytically pure **20** (285 mg, 0.66 mmol, 83%).

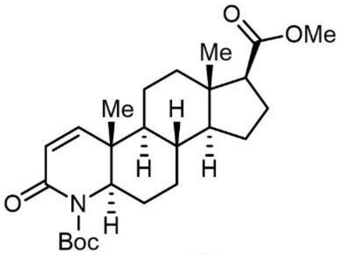

20

^{1}H NMR (500 MHz, chloroform-*d*, 23 °C) δ 6.84 (d, *J* = 9.5 Hz, 1H), 5.81 (d, *J* = 10.0 Hz, 1H), 3.67 (s, 3H), 3.56 (dd, *J* = 12.5, 3.5 Hz, 1H), 2.36 (t, *J* = 9.4 Hz, 1H), 2.14 (dddd, *J* = 14.2, 11.4, 9.2, 2.9 Hz, 1H), 2.05 (dt, *J* = 12.7, 3.2 Hz, 1H), 1.99 (dq, *J* = 12.8, 3.6 Hz, 1H), 1.87 – 1.64 (m, 4H), 1.57 (dd, *J* = 12.8, 3.7 Hz, 1H), 1.53 (s, 9H), 1.49 – 1.35 (m, 2H), 1.34 – 1.22 (m, 2H), 1.14 (ddd, *J* = 12.6, 10.6, 7.0 Hz, 1H), 1.04 (s, 3H, overlapping), 1.07 – 0.97 (m, 2H, overlapping), 0.68 (s, 3H).

^{13}C{^{1}H} NMR (126 MHz, chloroform-*d*, 23 °C) δ 174.4, 164.4, 153.3, 151.5, 122.6, 83.8, 63.7, 55.4, 55.2, 51.4, 47.8, 44.3, 39.7, 38.2, 35.2, 29.7, 27.9, 24.4, 23.8, 23.7, 21.3, 13.8, 13.0.

HRMS (ESI): [M+Na] calcd.: 454.2564; found: 454.2558.

3.2 Reactivity of organonickel complexes and characterization of organic products

3.21 Isolation of ring contraction product **6a**

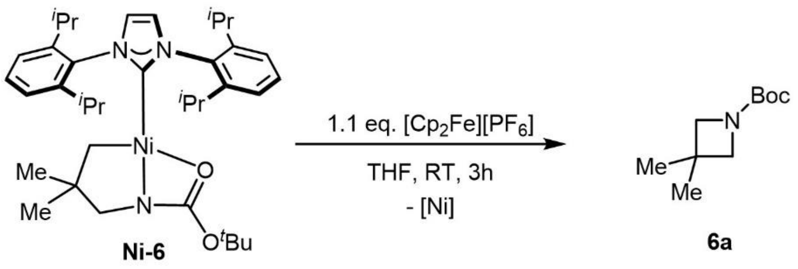

In an argon-filled glovebox, an 8 mL scintillation vial was added the nickel complex **Ni-6** (31.6 mg, 0.05 mmol, 1 eq.), 2 mL THF and a stir bar. To the solution was added ferrocenium hexafluorophosphate (18.2 mg, 0.055 mmol, 1.1 eq.). The solution was allowed to stir at RT for 3 hours. The content was filtered, and the volatiles were removed under vacuum. The residue was sampled for analysis in benzene-d_6 with TMS_2O as internal standard. The formation of the ring-contracted azetidine product **6a** (73% NMR yield) was confirmed by ^{1}H NMR spectroscopy. The sample was then purified by silica gel column chromatography (hexanes : ethyl acetate = 7 : 3) to afford **6a** (6.3 mg, 0.034 mmol, 68%).

^{1}H NMR (500 MHz, benzene-d_6, 23 °C) δ 3.48 (s, 4H), 1.50 (s, 9H), 0.77 (s, 6H).

^{1}H NMR (500 MHz, chloroform-*d*, 23 °C) δ 3.60 (s, 4H), 1.44 (s, 9H), 1.24 (s, 6H).

^{13}C{^{1}H} NMR (126 MHz, chloroform-*d*, 23 °C) δ 157.0, 79.2, 61.6, 30.8, 28.6, 27.1.

The NMR data are in accordance with those reported.[29]

3.22 Isolation of hydrolysis product **6b**

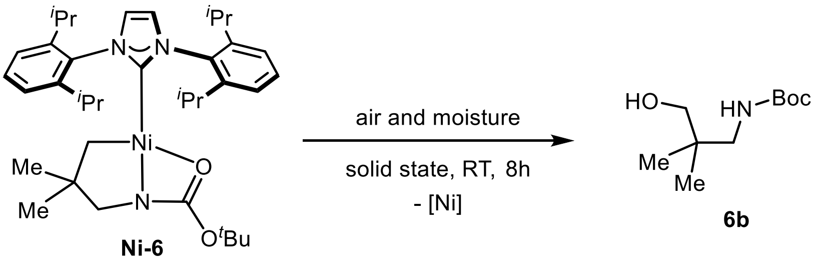

The solid nickel complex **Ni-6** (0.05 mmol, 31.6 mg) was exposed to air in an open Schlenk flask at room temperature for 8 hours. A color change from black-red to grayish brown was observed. The flask was then sealed, evacuated and brought into an argon-filled glovebox. The content was sampled for NMR analysis in benzene-d_6 with TMS_2O as internal standard. The formation of the ring-opened hydrolysis product **6b** (95% NMR yield) was confirmed by ^{1}H NMR spectroscopy. The sample was then purified by silica gel column chromatography (dichloromethane : methanol = 95 : 5) to afford **6b** (7.5 mg, 0.037 mmol, 74%).

^{1}**H NMR** (500 MHz, benzene-d_6, 23 °C) δ 4.24 (br s, 1H), 3.83 (br s, 1H), 3.20 (br s, 2H), 2.72 (d, *J* = 6.8 Hz, 2H), 1.37 (s, 9H), 0.61 (s, 6H).

^{1}**H NMR** (400 MHz, chloroform-*d*, 23 °C) δ 4.85 (br s, 1H), 3.71 (app t, *J* = 7.1 Hz, 1H), 3.23 (d, *J* = 7.4 Hz, 2H), 2.99 (d, *J* = 7.0 Hz, 2H), 1.47 (s, 9H), 0.87 (s, 6H).

The NMR data are in accordance with those reported.[30]

3.23 Isolation of ring contraction product **10a**

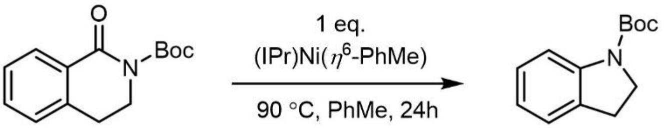

10 **10a**

In an argon-filled glovebox, a 150 mL Schlenk flask was charged with **10** (24.7 mg, 0.1 mmol, 1 eq.), IPrNi(η^6-PhMe) (54.0 mg, 0.1 mmol, 1 eq.), 3 mL toluene, a stir bar and sealed. The reaction was stirred at 90 °C for 24 hours. The volatiles were then removed under vacuum. The solid residue was sampled for ^{1}H NMR analysis in toluene-d_8 with TMS_2O as internal standard. The formation of **10a** (86 % NMR yield) was confirmed by comparing to the spectrum of an authentic sample. The sample was then purified by silica gel column chromatography (hexanes : ethyl acetate = 7 : 3) to afford **10a** (18.0 mg, 0.082 mmol, 82%). Attempts to render this reaction catalytic at higher temperature (up to 150 °C) that would facilitate CO dissociation from $(L)_nNi(CO)_x$ species and substrate coordination was unsuccessful due to the thermal instability of the Boc group.

^{1}**H NMR** (500 MHz, toluene-d_8, 23 °C) δ 7.89 – 7.54 (br s, 1H), 7.12 – 6.96 (m, 1H), 6.87 – 6.83 (m, 1H), 6.81 – 6.75 (m, 1H), 3.84 – 3.29 (br m, 2H), 2.46 (t, *J* = 8.6 Hz, 2H), 1.48 (s, 9H).

^{1}**H NMR** (500 MHz, chloroform-*d*, 23 °C) δ 7.86 – 7.37 (br s, 1H), 7.21 – 7.14 (m, 2H), 6.94 (td, *J* = 7.4, 1.1 Hz, 1H), 4.00 (t, *J* = 8.6 Hz, 2H), 3.11 (t, *J* = 9.0 Hz, 2H), 1.59 (s, 9H).

The NMR data are in accordance with those reported.[31]

3.24 Synthesis of **Ni-19** from **19**

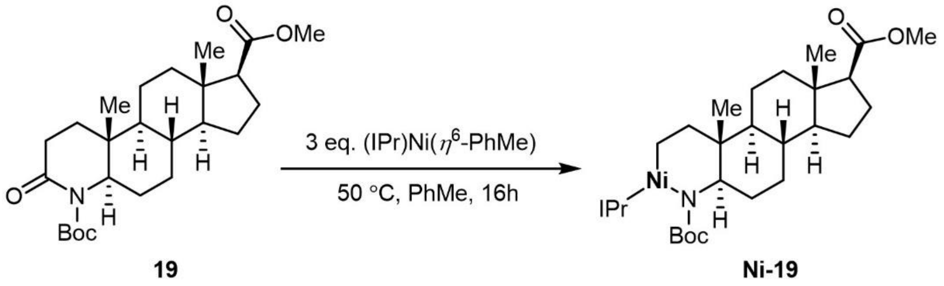

In an argon-filled glovebox, a 150 mL Schlenk flask was charged with **19** (43.4 mg, 0.1 mmol, 1 eq.), (IPr)Ni(η^6-PhMe) (161.8 mg, 0.3 mmol, 3 eq.), 5 mL toluene, a stir bar and sealed. The reaction was stirred at 50 °C for 16 hours. The solution was then filtered, and the volatiles were removed under vacuum. The solid residue (175.8 mg) was sampled for ^{1}H NMR analysis in benzene-d_6 which confirmed the formation of **Ni-19** (>98% conv.). Purification of **Ni-19** by recrystallization proved difficult due to the high solubility of all product components in *n*-hexane.

3.25. Synthesis of **19a** from **Ni-19**

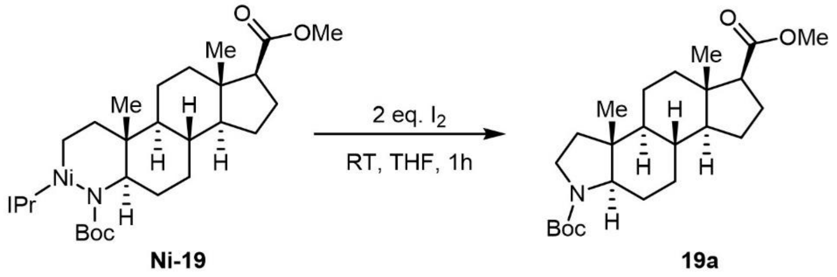

In an argon-filled glovebox, an 8 mL scintillation vial was charged with **Ni-19** (175.8 mg product mixture from synthesis with 0.1 mmol **19**), 4 mL THF, a stir bar and sealed with a septum cap. The reaction was brought out, and a 1 mL THF solution of I_2 (50.8 mg, 0.2 mmol, 2 eq.) was added via syringe. The reaction was stirred at RT for 1h and quenched by adding a 5 mL methanol solution of sodium thiosulfate $Na_2S_2O_3 \cdot 5H_2O$ (50 mg). The volatiles were removed under vacuum, and the solid residue was purified by silica gel column chromatography (*n*-hexane : ethyl acetate = 9 : 1) to afford **19a** (30.4 mg, 0.075 mmol, 75% over two steps). Slow evaporation of a concentrated solution of **19a** in 20:1 *n*-hexane : ethyl acetate at room temperature afforded crystals suitable for X-ray diffraction.

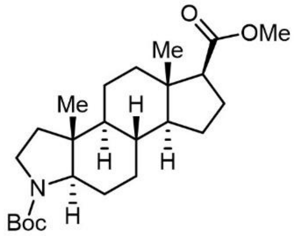

19a

^{1}H NMR (500 MHz, chloroform-*d*, 23 °C) δ 3.67 (s, 3H), 3.47 – 3.27 (br m, 2H), 2.90 – 2.75 (br m, 1H), 2.75 – 2.40 (br m, 2H), 2.35 (t, *J* = 9.5 Hz, 1H), 2.12 (dddd, *J* = 14.0, 11.6, 9.1, 2.9 Hz, 1H), 1.97 (dt, *J* = 12.7, 3.3 Hz, 1H), 1.84 – 1.76 (m, 1H), 1.76 – 1.70 (m, 1H), 1.68 (dq, *J* = 9.4, 2.6 Hz, 1H), 1.66 – 1.59 (m, 1H), 1.45 (s, 9H), 1.44 – 1.41 (m, 2H), 1.41 – 1.39 (m, 1H), 1.38 – 1.29 (m, 1H), 1.29 – 1.20 (m, 2H), 1.10 (ddd, *J* = 12.7, 10.8, 7.1 Hz, 1H), 0.95 (td, *J* = 13.1, 4.6 Hz, 1H), 0.91 – 0.84 (m, 1H), 0.83 (s, 3H), 0.66 (s, 3H).

^{13}C{^{1}H} NMR (126 MHz, chloroform-*d*, 23 °C) δ 174.7, 156.3, 79.1, 67.9, 55.4, 55.3, 52.3, 51.4, 46.3, 44.8, 44.5, 38.3, 36.2, 35.5, 30.3, 28.8, 25.4, 24.7, 23.8, 23.0, 14.2, 13.9.

HRMS (ESI): [M+Na] calcd.: 428.2771; found: 428.2768.

3.26. Synthesis of **^{13}C-19** from **Ni-19**

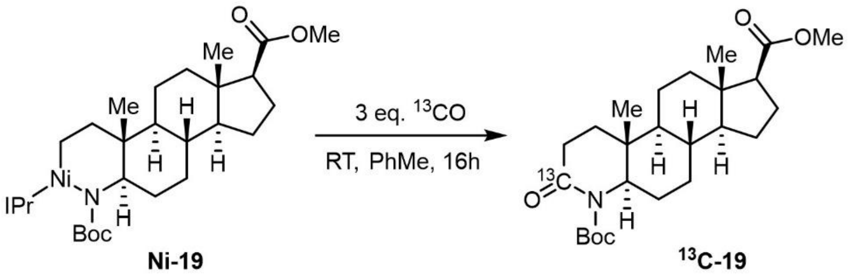

Generation of ^{13}CO(g) from ^{13}COgen in COware:

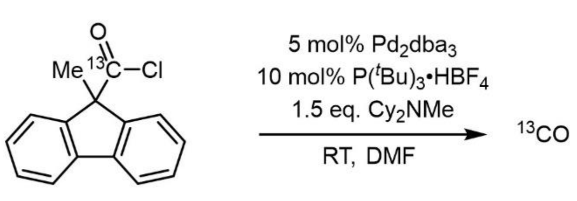

In an argon-filled glovebox, a COware was charged with **Ni-19** (169.2 mg product mixture from synthesis with 0.1 mmol **19**), 2 mL toluene, a stir bar in one chamber and ^{13}COgen (73.1 mg, 0.3 mmol), Pd_2dba_3 (13.7 mg, 0.015 mmol), P(^{t}Bu)$_3$ · HBF_4 (8.7 mg, 0.03 mmol), a stir bar in the other chamber. The COware was sealed and brought out of the glovebox. To the ^{13}COgen chamber was added Cy_2NMe (96 uL, 0.45 mmol) and 2 mL anhydrous DMF via syringe under N_2. The reactions were allowed to stir at room temperature for 16 hours. The solution was quenched by exposure to air and the volatiles were

removed by vacuum. The solid residue was purified by silica gel column chromatography (*n*-hexane : ethyl acetate = 7 : 3) to afford **^{13}C-19** (24.3 mg, 0.056 mmol, 56% over two steps). The %^{13}C-incorporation (80±1%) was determined by mass spectrometry analysis.

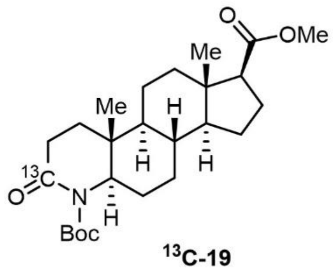

^{13}C-19

^{1}H NMR (500 MHz, chloroform-*d*, 23 °C) δ 3.67 (s, 3H), 3.34 (dd, *J* = 12.1, 3.3 Hz, 1H), 2.54 – 2.40 (m, 2H), 2.35 (t, *J* = 9.4 Hz, 1H), 2.13 (dddd, *J* = 14.1, 11.7, 9.2, 3.0 Hz, 1H), 2.04 – 1.99 (m, 1H), 1.92 – 1.84 (m, 1H), 1.84 – 1.64 (m, 4H), 1.63 – 1.57 (m, 1H), 1.52 (s, 9H), 1.50 – 1.23 (m, 6H), 1.11 (ddd, *J* = 12.6, 10.6, 7.0 Hz, 1H), 1.05 – 0.94 (m, 1H, overlapping), 0.98 (s, 3H, overlapping), 0.87 – 0.80 (m, 1H), 0.67 (s, 3H).

^{13}C{^{1}H} NMR (126 MHz, chloroform-*d*, 23 °C) δ 174.5, 170.6 (^{13}C-labeled), 154.2, 84.0, 64.8, 55.4, 55.3, 51.6, 51.4, 44.3, 38.3, 35.8, 35.0, 33.1, 29.7, 29.5 (d, $^{1}J_{C\text{-}C}$ = 49.2 Hz), 27.8, 25.0, 24.4, 23.8, 21.2, 13.8, 12.5.

HRMS (ESI): [M+Na] calcd.: 457.2754; found: 457.2742.

3.27. Synthesis of **Ni-20** from **20**

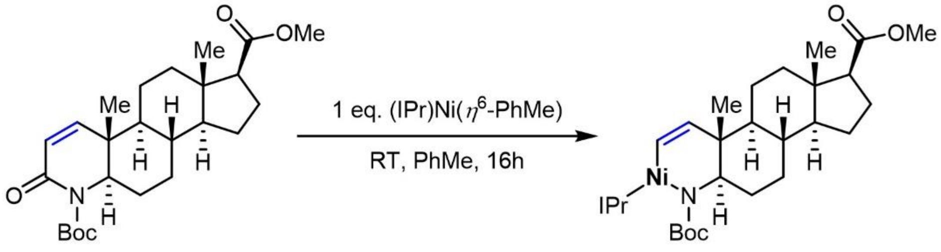

20 **Ni-20**

In an argon-filled glovebox, a 150 mL Schlenk flask was charged with **20** (43.2 mg, 0.1 mmol, 1 eq.), (IPr)Ni(η^6-PhMe) (72.4 mg, 0.1 mmol, 1.34 eq.), 5 mL toluene, a stir bar and sealed. The reaction was stirred at RT for 16 hours. The solution was then filtered, and the volatiles were removed under vacuum. The solid residue (102.6 mg) was sampled for ^{1}H NMR analysis which confirmed the full consumption of **20** (>98% conv.). Purification of **Ni-20** by recrystallization proved difficult due to the high solubility of all product components in *n*-hexane.

3.28. Synthesis of **^{13}C-20** from **Ni-20**

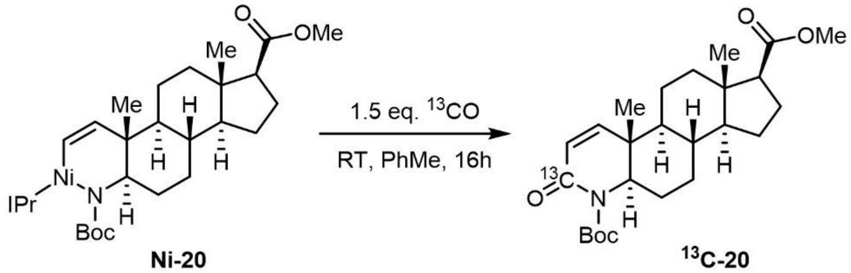

In an argon-filled glovebox, a COware was charged with **Ni-20** (102.6 mg product mixture from synthesis with 0.1 mmol **20**), 2 mL toluene, a stir bar in one chamber and ^{13}COgen (36.6 mg, 0.15 mmol), Pd_2dba_3 (6.9 mg, 0.0075 mmol), $P(tBu)_3$-HBF_4 (4.4 mg, 0.015 mmol), a stir bar in the other chamber. The COware was sealed and brought out of the glovebox. To the ^{13}COgen chamber was added Cy_2NMe (48 uL, 0.225 mmol) and 2 mL anhydrous DMF via syringe under N_2. The reactions were allowed to stir at room temperature for 16 hours. The solution was quenched by exposure to air and the volatiles were removed by vacuum. The solid residue was purified by silica gel column chromatography (*n*-hexane : ethyl acetate = 9 : 1) to afford **^{13}C-20** (29.8 mg, 0.069 mmol, 69% over two steps). The %^{13}C-incorporation (17±1%) was determined by mass spectrometry analysis.

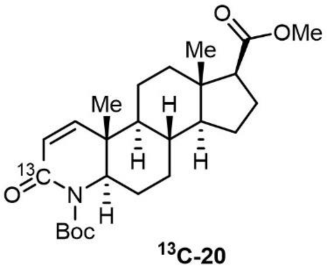

^{13}C-20

^{1}H NMR (500 MHz, chloroform-*d*, 23 °C) δ 6.87 – 6.81 (m, 1H), 5.84 – 5.80 (m, 1H), 3.68 (s, 3H), 3.56 (dd, *J* = 12.5, 3.6 Hz, 1H), 2.36 (t, *J* = 9.4 Hz, 1H), 2.14 (dddd, *J* = 14.1, 11.7, 9.2, 2.9 Hz, 1H), 2.06 (dt, *J* = 12.7, 3.2 Hz, 1H), 2.00 (dq, *J* = 12.8, 3.6 Hz, 1H), 1.88 – 1.64 (m, 4H), 1.64 – 1.57 (m, 1H), 1.53 (s, 9H), 1.49 – 1.35 (m, 2H), 1.33 – 1.22 (m, 2H), 1.14 (ddd, *J* = 12.6, 10.7, 7.0 Hz, 1H), 1.04 (s, 3H, overlapping), 1.07 – 0.97 (m, 2H, overlapping), 0.68 (s, 3H).

^{13}C{^{1}H} NMR (126 MHz, chloroform-*d*, 23 °C) δ 174.4, 164.4 (^{13}C-enriched), 153.3, 151.5, 122.6, 83.9, 63.7, 55.4, 55.2, 51.5, 47.8, 44.3, 39.7, 38.2, 35.2, 29.6, 27.9, 24.4, 23.8, 23.7, 21.3, 13.8, 13.0.

HRMS (ESI): [M+Na] calcd.: 455.2597; found: 455.2596.

4. Computational studies

4.1 General information

All calculations were run on the Euler cluster from the ETH Zurich. Unless stated otherwise conformer searches were performed for all proposed intermediates shown in the main text **Fig. 4A** with xTB version 6.4.1,[32,33] CREST version 2.11,[34,35] and CENSO version 1.2.0.[36] For CREST, we used the gbsa solvent model (toluene) and chose the xtb optimized structure as the starting structure. For CENSO, we used parts 0-2:

- In part 0 (cheap prescreening of the electronic energy) we used xtb 6.4.1 gfn2 with the b97-d3 functional and def2-SV(P) basis set. The threshold for the next part was set to 4 kcal/mol relative to the lowest energy.
- In part 1 (prescreening of the free energy) we used xtb 6.4.1 gfn2 with the b97-d3 functional and def2-SV(P) basis set. The solvation correction was calculated with xtb, using cpcm (toluene) and the thermochemical corrections were computed at 298.15 K. The threshold for the next part was set to 3.5 kcal/mol relative to the lowest energy.
- In part 2 (optimization of the remaining conformers) we used ORCA 5.0.2 with the b97-d3 functional and def2-SV(P) basis set. The solvation correction was calculated using cpcm (toluene) and the thermochemical corrections were computed at 298.15 K.

The described settings for CENSO calculations are also summarized in the .censorc file at the end of this section.

The geometry of the lowest lying conformer from CENSO was further optimized by DFT using ORCA 5.0.2[37] with the BP86 functional,[38,39] the def2-tzvp basis set for Ni and the def2-svp basis set for all other atoms.[40] Grimme's atom-pairwise dispersion correction with the Becke-Johnson damping scheme was used,[41,42] the def2/J auxiliary basis set was chosen,[43] and the cpcm solvation model was used for toluene.[44]

Input example for geometry optimizations and frequency calculations:

```
! BP86 def2-SVP D3BJ RI def2/J DefGrid2 tightopt freq cpcm(toluene)
%basis
newgto Ni "def2-tzvp" end
end
*xyzfile 0 1 <coordinates>.xyz
```

Transition states were located mainly from relaxed potential energy surface scan using ORCA at the same level of theory as the ground state geometry optimizations. These transition state guesses were further optimized and were confirmed by the number of imaginary frequencies (N_{if}=1) and IRC calculations or manual displacement.

The electronic energy of all structures was further calculated with the PBE0 functional,[45] def2-qzvp basis set for Ni and def2-tzvp basis set for all other atoms. Grimme's atom-pairwise dispersion correction with the Becke-Johnson damping scheme was used, the def2/J auxiliary basis set was chosen, and the cpcm solvation model was used for toluene.

Input example for the single-point calculations:

```
! PBE0 def2-TZVP D3BJ RIJCOSX def2/j cpcm(toluene)
%basis
newgto Ni "def2-qzvp" end
end
*xyzfile 0 1 <coordinates>.xyz
```

The Gibbs free energy of the reaction was calculated by adding the electronic energy from the single-point calculation to the thermochemical correction from the frequency calculation at 25 °C, assuming standard conditions for all reactants.

For structures which are shown in the SI chapter 4.2, no additional conformer search was performed, after pre-optimization with xTB version 6.4.1 the geometry optimization / frequency calculation as well as electronic energy calculations were performed with the same settings using ORCA 5.0.2 as specified above.

All structures were visualized with CYLView20[46] and the cartesian coordinates of all optimized structures are given in a separate .xyz file.

.censorc file:

```
$CENSO global configuration file: .censorc
$VERSION:1.2.0

ORCA: /path/excluding/binary/
ORCA version: 5.0.2
GFN-xTB: /path/including/binary/xtb-binary
CREST: /path/including/binary/crest-binary
mpshift: /path/including/binary/mpshift-binary
escf: /path/including/binary/escf-binary

#COSMO-RS
ctd = BP_TZVP_C30_1601.ctd cdir = "/software/cluster/COSMOthermX16/COSMOtherm/CTDATA-FILES"
ldir = "/software/cluster/COSMOthermX16/COSMOtherm/CTDATA-FILES"
```

```
$ENDPROGRAMS

$CRE SORTING SETTINGS:
$GENERAL SETTINGS:
nconf: all                      # ['all', 'number e.g. 10 up to all conformers']
charge: 0                       # ['number e.g. 0']
unpaired: 0                     # ['number e.g. 0']
solvent: toluene                # ['gas', 'acetone', 'acetonitrile', 'aniline', 'benzaldehyde', 'benzene', 'ccl4', '...']
prog_rrho: xtb                  # ['xtb']
temperature: 298.15             # ['temperature in K e.g. 298.15']
trange: [273.15, 378.15, 5]     # ['temperature range [start, end, step]']
multitemp: on                   # ['on', 'off']
evaluate_rrho: on               # ['on', 'off']
consider_sym: on                # ['on', 'off']
bhess: on                       # ['on', 'off']
imagthr: automatic              # ['automatic or e.g., -100    # in cm-1']
sthr: automatic                 # ['automatic or e.g., 50     # in cm-1']
scale: automatic                # ['automatic or e.g., 1.0 ']
rmsdbias: off                   # ['on', 'off']
sm_rrho: alpb                   # ['alpb', 'gbsa']
progress: off                   # ['on', 'off']
check: on                       # ['on', 'off']
prog: orca                      # ['tm', 'orca']
func: b97-d3                    # ['b3-lyp', 'b3lyp', 'b3lyp-3c', 'b3lyp-d3', 'b3lyp-d3(0)', 'b3lyp-d4', 'b3lyp-nl', '...']
basis: def2-SV(P)               # ['automatic', 'def2-TZVP', 'def2-mSVP', 'def2-mSVP', 'def2-mSVP', 'def2-mSVP',
'...']
maxthreads: 1                   # ['number of threads e.g. 2']
omp: 8                          # ['number cores per thread e.g. 4']
balance: off                    # ['on', 'off']
cosmorsparam: automatic         # ['automatic', '12-fine', '12-normal', '13-fine', '13-normal', '14-fine', '...']

$PART0 - CHEAP-PRESCREENING - SETTINGS:
part0: on                       # ['on', 'off']
func0: b97-d3                   # ['b3-lyp', 'b3lyp', 'b3lyp-3c', 'b3lyp-d3', 'b3lyp-d3(0)', 'b3lyp-d4', '...']
basis0: def2-SV(P)              # ['automatic', 'def2-SV(P)', 'def2-TZVP', 'def2-mSVP', 'def2-mSVP', 'def2-mSVP',
'...']
part0_gfnv: gfn2                # ['gfn1', 'gfn2', 'gfnff']
part0_threshold: 4.0            # ['number e.g. 4.0']

$PART1 - PRESCREENING - SETTINGS:
# func and basis is set under GENERAL SETTINGS
```

```
part1: on                        # ['on', 'off']
smgsolv1: cpcm                   # ['alpb_gsolv', 'cosmo', 'cosmors', 'cosmors-fine', 'cpcm', 'dcosmors', '...']
part1_gfnv: gfn2                 # ['gfn1', 'gfn2', 'gfnff']
part1_threshold: 3.5             # ['number e.g. 5.0']

$PART2 - OPTIMIZATION - SETTINGS:
# func and basis is set under GENERAL SETTINGS
part2: on                        # ['on', 'off']
prog2opt: prog                   # ['tm', 'orca', 'prog', 'automatic']
part2_threshold: 2.5             # ['number e.g. 4.0']
sm2: cpcm                        # ['cosmo', 'cpcm', 'dcosmors', 'default', 'smd']
smgsolv2: cpcm                   # ['alpb_gsolv', 'cosmo', 'cosmors', 'cosmors-fine', 'cpcm', 'dcosmors', '...']
part2_gfnv: gfn2                 # ['gfn1', 'gfn2', 'gfnff']
ancopt: on                       # ['on']
hlow: 0.01                       # ['lowest force constant in ANC generation, e.g. 0.01']
opt_spearman: on                 # ['on', 'off']
part2_P_threshold: 99            # ['Boltzmann sum threshold in %. e.g. 95 (between 1 and 100)']
optlevel2: automatic             # ['crude', 'sloppy', 'loose', 'lax', 'normal', 'tight', 'vtight', 'extreme', '...']
optcycles: 8                     # ['number e.g. 5 or 10']
spearmanthr: -4.0                # ['value between -1 and 1, if outside set automatically']
radsize: 10                      # ['number e.g. 8 or 10']
crestcheck: off                  # ['on', 'off']

$PART3 - REFINEMENT - SETTINGS:
part3: off                       # ['on', 'off']
prog3: prog                      # ['tm', 'orca', 'prog']
func3: pw6b95                    # ['b3-lyp', 'b3lyp', 'b3lyp-3c', 'b3lyp-d3', 'b3lyp-d3(0)', 'b3lyp-d4', 'b3lyp-nl', '...']
basis3: def2-TZVPD               # ['DZ', 'QZV', 'QZVP', 'QZVPP', 'SV(P)', 'SVP', 'TZVP', 'TZVPP', 'aug-cc-pV5Z',
'...']
smgsolv3: cpcm                   # ['alpb_gsolv', 'cosmo', 'cosmors', 'cosmors-fine', 'cpcm', 'dcosmors', '...']
part3_gfnv: gfn2                 # ['gfn1', 'gfn2', 'gfnff']
part3_threshold: 99              # ['Boltzmann sum threshold in %. e.g. 95 (between 1 and 100)']

$NMR PROPERTY SETTINGS:
$PART4 SETTINGS:
part4: off                       # ['on', 'off']
couplings: on                    # ['on', 'off']
progJ: prog                      # ['tm', 'orca', 'prog']
funcJ: pbe0                      # ['b3-lyp', 'b3lyp', 'b3lyp-3c', 'b3lyp-d3', 'b3lyp-d3(0)', 'b3lyp-d4', 'b3lyp-nl', '...']
basisJ: def2-TZVP                # ['DZ', 'QZV', 'QZVP', 'QZVPP', 'SV(P)', 'SVP', 'TZVP', 'TZVPP', 'aug-cc-pV5Z',
'...']
```

```
sm4J: smd                       # ['cosmo', 'cpcm', 'dcosmors', 'smd']
shieldings: on                  # ['on', 'off']
progS: prog                     # ['tm', 'orca', 'prog']
funcS: pbe0                     # ['b3-lyp', 'b3lyp', 'b3lyp-3c', 'b3lyp-d3', 'b3lyp-d3(0)', 'b3lyp-d4', 'b3lyp-nl', '...']
basisS: def2-TZVP               # ['DZ', 'QZV', 'QZVP', 'QZVPP', 'SV(P)', 'SVP', 'TZVP', 'TZVPP', 'aug-cc-pV5Z',
'...']
sm4S: smd                       # ['cosmo', 'cpcm', 'dcosmors', 'smd']
reference_1H: TMS               # ['TMS']
reference_13C: TMS              # ['TMS']
reference_19F: CFCl3            # ['CFCl3']
reference_29Si: TMS             # ['TMS']
reference_31P: TMP              # ['TMP', 'PH3']
1H_active: on                   # ['on', 'off']
13C_active: on                  # ['on', 'off']
19F_active: off                 # ['on', 'off']
29Si_active: off                # ['on', 'off']
31P_active: off                 # ['on', 'off']
resonance_frequency: 300.0      # ['MHz number of your experimental spectrometer setup']

$OPTICAL ROTATION PROPERTY SETTINGS:
$PART5 SETTINGS:
optical_rotation: off           # ['on', 'off']
funcOR: pbe                     # ['functional for opt_rot e.g. pbe']
funcOR_SCF: r2scan-3c           # ['functional for SCF in opt_rot e.g. r2scan-3c']
basisOR: def2-SVPD              # ['basis set for opt_rot e.g. def2-SVPD']
frequency_optical_rot: [589.0]  # ['list of freq in nm to evaluate opt rot at e.g. [589, 700]']
$END CENSORC
```

4.2 DFT-computed relative ground state energies of (IPr)Ni(0) precursors

The thermodynamical stability of (IPr)Ni(η^6-PhMe) was compared with alternative Ni(0) sources. We decided to choose Ni(COD)$_2$ as our reference point, since it is by far the most commonly employed source of Ni(0) in experimental protocols. In the following the computed reaction Gibbs free energies give a good indication about the thermodynamic stability of different Ni(0) compounds compared with Ni(COD)$_2$.

In comparison with the precursor, (IPr)Ni(η^6-PhMe) was computed to be roughly 10 kcal/mol less stable. This directly impacts the kinetic barriers for oxidative addition into the C–N bond of the lactams. An equilibrium mixture of (IPr)Ni(η^6-PhMe), (IPr)$_2$Ni, free IPr and Ni(COD)$_2$ was shown to be generated from mixing IPr and Ni(COD)$_2$ in toluene.[7] The comparison with Ni(IPr)$_2$, which is known to be formed in a mixture of Ni(COD)$_2$ and free IPr, shows how this compound is much more stable by roughly 7 kcal/mol. Not unexpectedly the coordination of in-situ liberated carbon monoxide to the Ni(0) center drastically lowers the energy of the compounds relative to Ni(COD)$_2$ and corroborates the formation of Ni carbonyl complexes as one of the driving forces of the reaction. Comparison of (IPr)Ni(η^6-PhMe) with alternative single-component (IPr)Ni(0) sources such as (IPr)Ni(η^2-norbornene)$_2$,[11] (IPr)Ni(η^2-styrene)$_2$,[47] and (IPr)Ni(η^2-methylacrylate)$_2$[48] shows how these ligands stabilize the Ni(0) center a lot more and make them undesirable as precursors for clean stoichiometric activations at room temperature. Lastly, we also decided to look into the thermodynamic stability of alternative Ni(0) sources than Ni(COD)$_2$. Both Ni(cyclododecatriene) and Ni(stilbene)$_3$[49] were found to have similarly low formation energy with respect to Ni(COD)$_2$. These results corroborate the high reactivity of (IPr)Ni(η^6-PhMe) and justify its use in the clean activation of lactams.

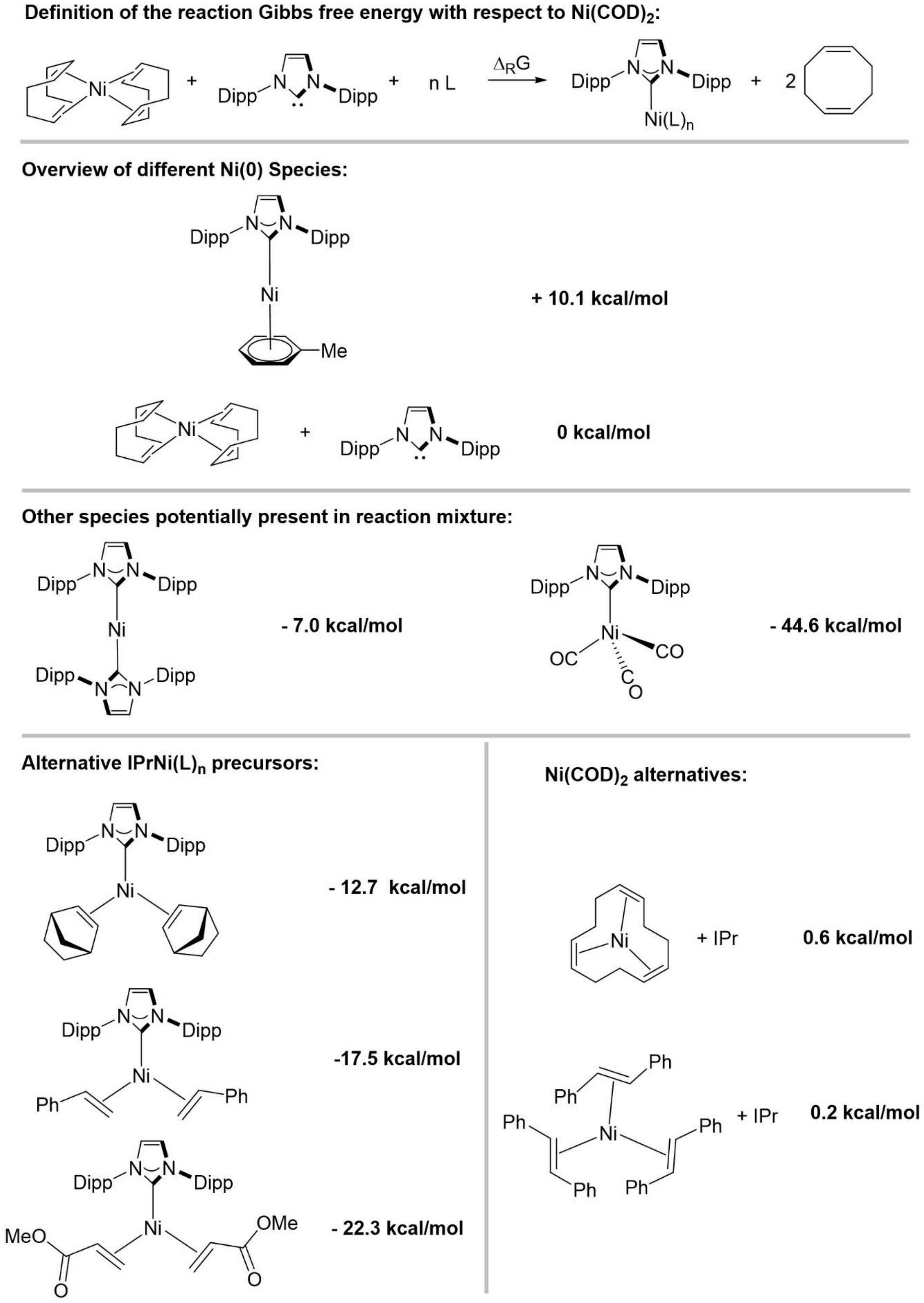

Supplementary Figure 1. Overview of the formation Gibbs free energies of selected Ni(0) compounds relative to $Ni(COD)_2$.

4.3 Correctional term for the barrier of C–N bond oxidative addition

Accounting for the equilibrium between (IPr)Ni(η^6-PhMe) and (IPr)Ni(lactam) in toluene solvent, the actual reaction Gibbs free energy ($\Delta_R G$) is computed as:[50,51]

$$\Delta_R G = \Delta_R G° + RT\ln(Q) \quad (1)$$

Where Q is given as:

$$Q = \frac{[\text{Toluene}][\text{Ni} - \text{Lactam complex}]}{[\text{IPrNi(Tol)}][\text{free Lactam}]} \quad (2)$$

While for standard conditions typically Q = 1 is assumed, we now consider the actual concentration for a typical reaction monitored in an NMR tube: 0.01 mmol lactam + 0.01 mmol (IPr)Ni(η^6-PhMe) in 0.4 mL of toluene, which corresponds to 3.77 mmol toluene.

Therefore, we find Q to be roughly 377 and $RT\ln(Q)$ = 1.987 x 10^{-3} kcal/(mol*K) * 298.15 K * 5.93 = 3.5 kcal/mol.

Accordingly, when accounting for the large excess of solvent as a competitive ligand, the actual barrier for the C–N bond oxidative addition would be roughly 3.5 kcal/mol higher when toluene needs to be released from (IPr)Ni(η^6-PhMe) into a pool of toluene solvent.

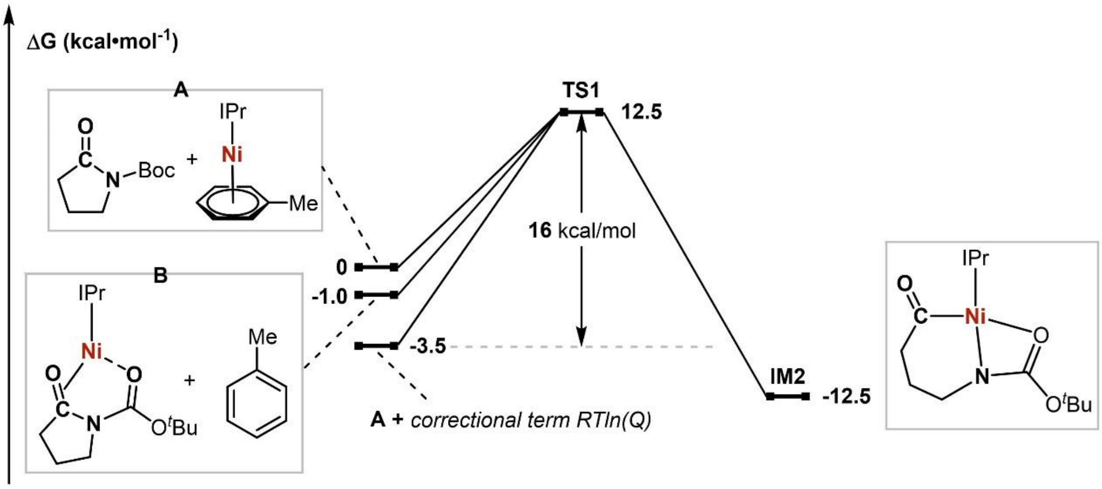

Supplementary Figure 2. Relative barriers for the C–N bond oxidative addition step of lactam **1**.

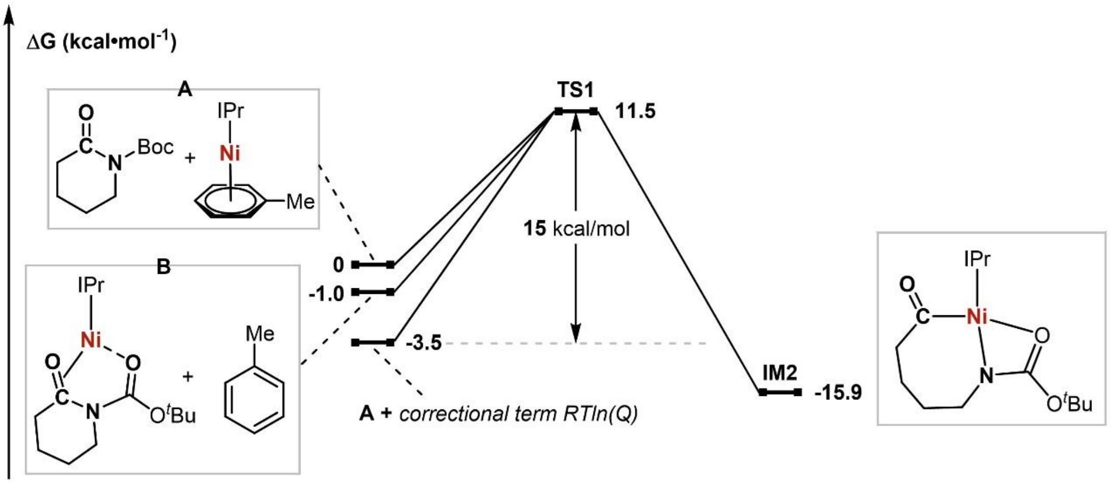

Supplementary Figure 3. Relative barriers for the C–N bond oxidative addition step of lactam **2**.

4.4 Participation of the Boc group in the C–N bond oxidative addition transition states

Based on previous computational studies[52,53] on the activation of linear amides by Ni(0) catalysts we sought to investigate if the oxidative addition into the lactam C–N bond would preferably follow a classical three-membered ring oxidative addition transition state or whether the coordinating ability of the Boc group might render a 5-membered ring transition state more favorable for cyclic amides.

For lactam **1** we managed to locate both possible activation modes of the C–N bond. Similar to most linear amides, the three-membered oxidative addition transition state is favored by roughly 5 kcal/mol over the alternative Boc-group assisted pathway. We located a C–N oxidative addition transition state with a very similar barrier for lactam **2**, albeit in this case the Boc group would be coordinated to the Ni-center. The alternative three-membered transition state for the activation of lactam **2** turned out to be roughly 2 kcal/mol higher in energy, suggesting that the preferred mode of activation might be ring-size dependent.

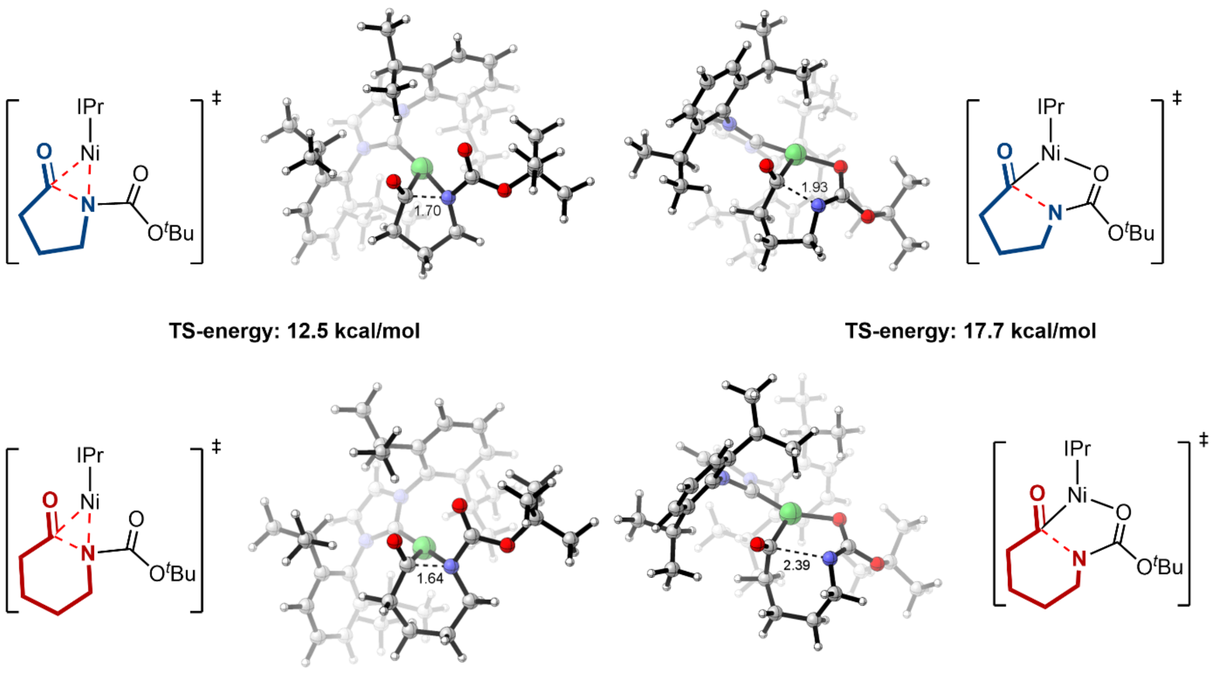

Supplementary Figure 4. Comparison of the computed transition state energies and geometries with and without the directing effect from the Boc group for 5- and 6-membered lactams. The computed energies are with respect to (IPr)Ni(PhMe) and the corresponding lactams as reference points.

4.5 Investigations into the CO dissociation step

General information

Association and dissociation transition states are notoriously difficult to model,[51] as typically the electronic energy is scanned, not the free energy, which lacks entropic contributions. As we still wished to gain some insight into the potential dissociation mode of the CO, we scanned the electronic energy as a function of the Ni–CO distance,[54] to compare different geometries of the nickel complex that could be involved in the process.

An example for the input is shown below:

```
! BP86 def2-SVP D3BJ RI def2/J DefGrid2 tightopt cpcm(toluene)
#resources
%basis
newgto Ni "def2-tzvp" end
end
%geom scan
B 0 95 = 2.00, 4.50, 101 # elongates the Ni-C bond from 2.00 to 4.50 Å in 101 steps (= 0.01 Å steps)
end
end
*xyzfile 0 1 <coordinate file>.xyz
```

γ-Lactam

For the γ-lactam system we wanted to compare the axial and in-plane CO dissociation. When attempting to scan the axial CO dissociation, however, the CO "dropped" into an in-plane position, between the amido and the nickel. This could potentially occur via an internal isomerization process.

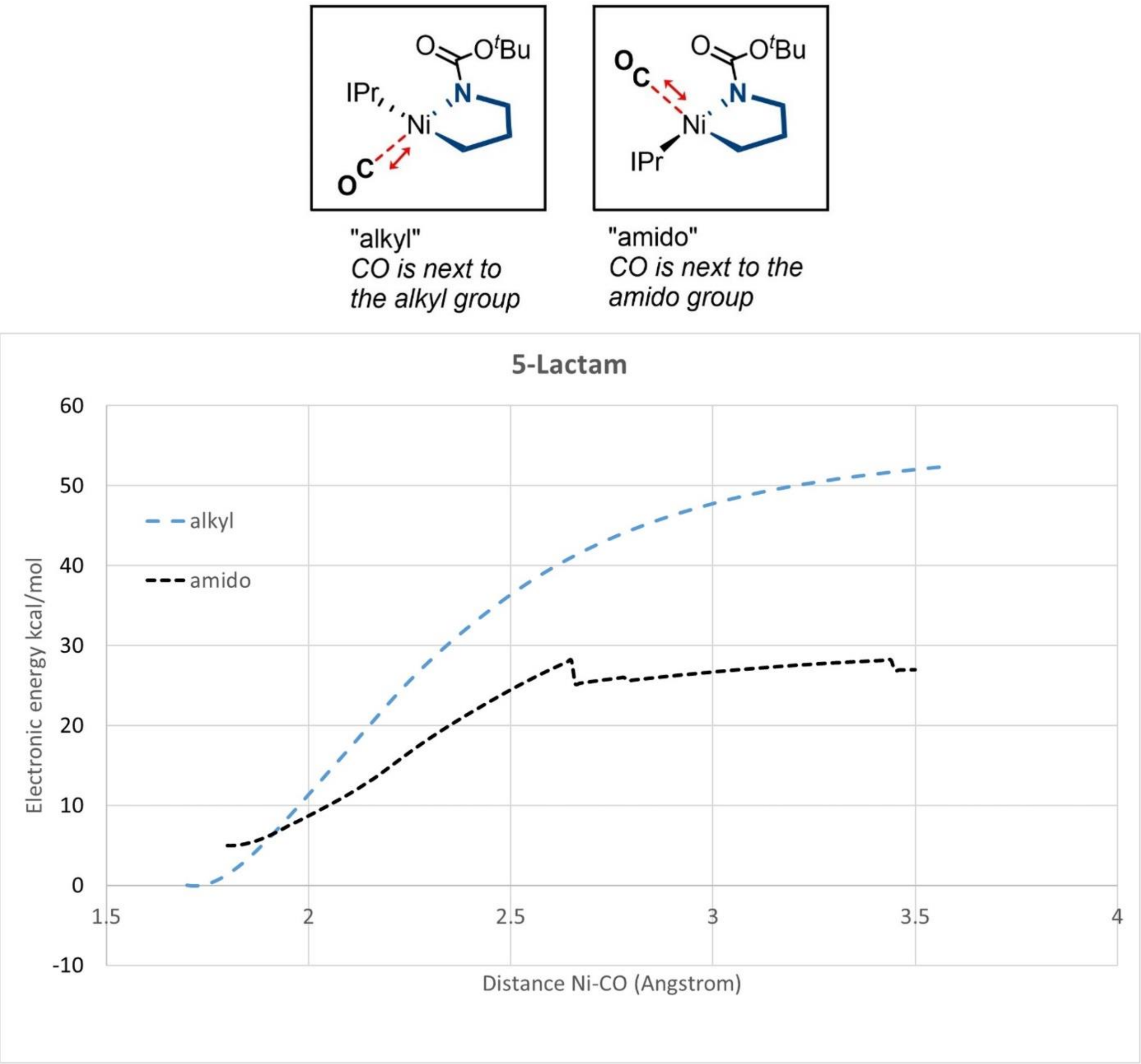

Supplementary Figure 5. Electronic energy as a function of Ni-CO distance for the *γ*-lactam **1**. While the "amido" intermediate is slightly higher in energy, the electronic energy penalty is significantly lower for a dissociating CO ligand. The difficulties in scanning an axially dissociating CO could imply an even less stable intermediate, but which could still lead to a facile dissociation.

δ-Lactam

For the δ-lactam system we decided to compare the axial and, as in the 5-lactam system, the two different in-plane CO dissociations.

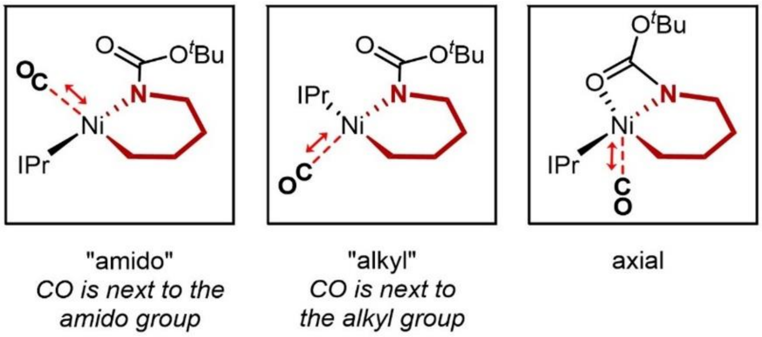

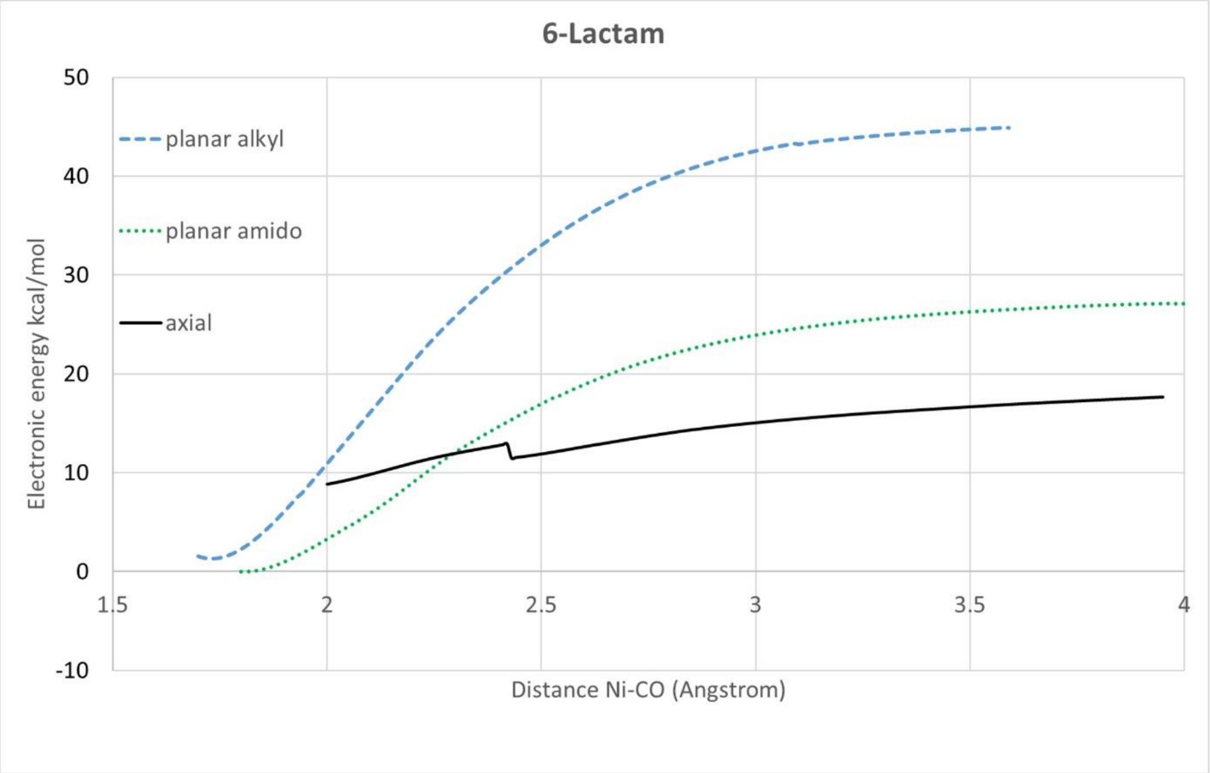

Supplementary Figure 6. Electronic energy as a function of Ni-CO distance for the δ-lactam **1**. For the 6-lactam system the "amido" intermediate has the lowest electronic energy and again has a lower electronic energy penalty for a dissociating CO ligand. However, despite a higher-energy starting point, the axial dissociation seems to be a lot more feasible with respect to the electronic energy.

5. Organometallic studies

5.1 Evaluation of nitrogen substituents and nickel precursors

In an argon-filled glovebox, lactams **2-py**, **2-Ts** and **2-Ac** (0.1 mmol) were subjected to (IPr)Ni(η^6-PhMe) (0.1 mmol) at room temperature in toluene and stirred for 20 hours. The reaction mixtures were sampled for ^{1}H NMR analysis. The product structures were further elucidated through single crystal X-ray analysis. Only the 2-pyridyl substituted δ-lactam **2-py** underwent the desired endocyclic metalation, while the Ts- and Ac-substituted δ-lactam **2-Ts** and **2-Ac** underwent the undesired exocyclic metalation affording nickel lactamate species.

Ni-2-py ^{1}H NMR (400 MHz, benzene-d_6): δ 7.27 – 7.22 (m, 2H), 7.20 – 7.14 (m, 4H), 7.03 – 7.01 (m, 1H), 7.01 – 6.99 (m, 1H), 6.93 (ddd, J = 8.9, 7.0, 1.9 Hz, 1H), 6.53 (s, 2H), 5.79 (ddd, J = 7.1, 5.3, 1.2 Hz, 1H), 5.47 (dt, J = 8.7, 1.1 Hz, 1H), 3.10 (hept, J = 6.8 Hz, 4H), 2.39 (t, J = 6.0 Hz, 2H), 1.41 (d, J = 6.8 Hz, 12H), 1.30 – 1.24 (s, 2H), 1.05 (d, J = 6.9 Hz, 12H), 0.78 (dt, J = 10.7, 5.7 Hz, 2H), 0.65 – 0.57 (m, 2H).

Ni-2-Ts ^{1}H NMR (500 MHz, benzene-d_6): δ 7.38 (dd, J = 8.4, 7.0 Hz, 2H), 7.35 – 7.26 (m, 4H), 6.95 – 6.89 (m, 2H), 6.71 – 6.65 (m, 2H), 6.53 (s, 2H), 3.20 – 3.10 (m, 4H), 2.19 (s, 3H), 2.06 (t, J = 5.8 Hz, 2H), 1.65 – 1.59 (m, 2H), 1.43 (d, J = 6.7 Hz, 12H), 1.06 (d, J = 6.9 Hz, 12H), 0.87 – 0.81 (m, 2H).

Ni-2-Ac ^{1}H NMR (500 MHz, benzene-d_6): δ 7.37 (m, 2H), 7.27 (d, J = 1.2 Hz, 4H), 6.54 (s, 2H), 3.21 – 3.08 (m, 4H), 2.43 (t, J = 5.8 Hz, 2H), 1.67 – 1.64 (m, 2H), 1.62 (d, J = 6.7 Hz, 12H), 1.10 (d, J = 6.9 Hz, 12H), 1.09 – 1.04 (m, 2H), 0.95 – 0.90 (m, 2H), -0.76 (s, 3H).

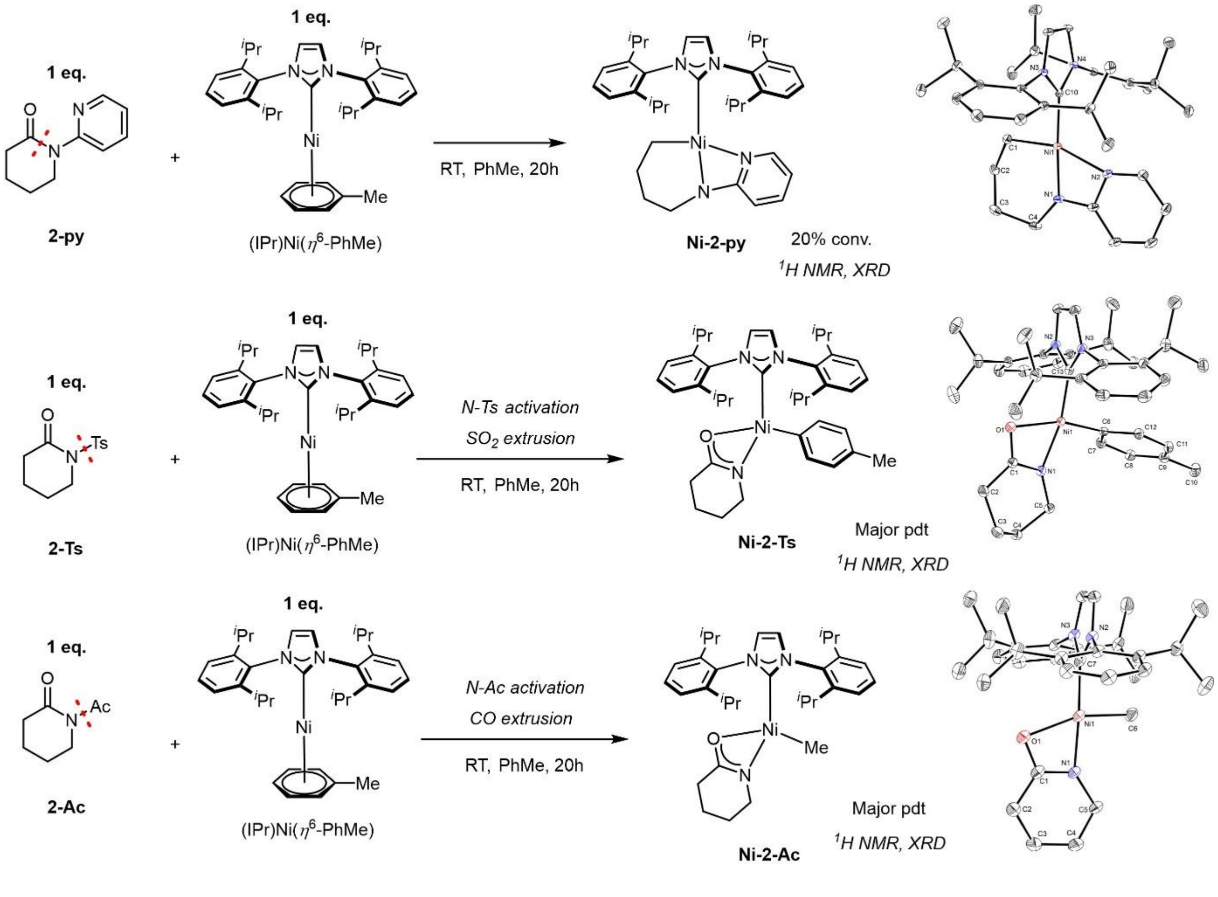

Supplementary Figure 7. Evaluation of alternative nitrogen substituents. X-ray structures at 30% thermal ellipsoids with H atoms omitted for clarity.

Lactam **2** (0.1 mmol) was treated with alternative nickel precursors (0.1 mmol): IPr + Ni(COD)$_2$; IPr$_2$Ni and (IPr)Ni(η^2-norbornene)$_2$ at room temperature in toluene for 20 hours. The conversion to the product **Ni-2** was determined by ^{1}H NMR analysis.

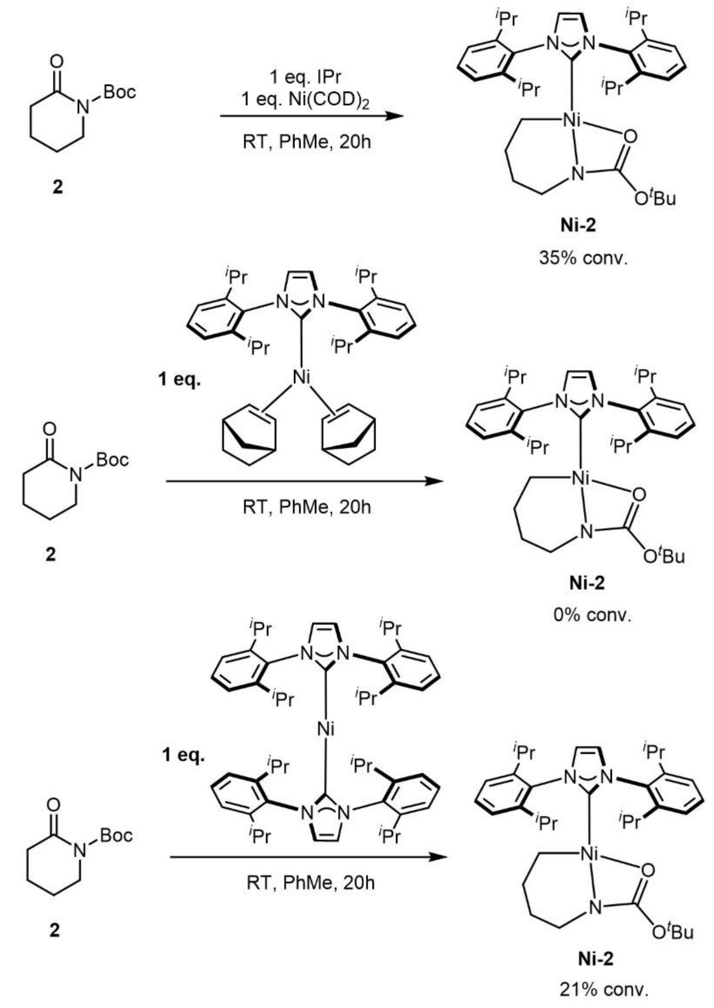

Supplementary Figure 8. Evaluation of alternative nickel precursors.

5.2. NMR monitoring of C–N bond oxidative addition and CO deinsertion reaction progress

General procedure for *in situ* NMR monitoring

In an argon-filled glovebox, *N*–Boc lactam substrate (0.01 mmol) and (IPr)Ni(η^6-PhMe) (0.01 mmol) were added into a vial, followed by addition of 0.4 mL toluene-d_8 or benzene-d_6. The solution was transferred into an oven-dried NMR tube and sealed for NMR timepoint analyses.

5.21 Reaction progress monitoring of **Ni-1**

In an argon-filled glovebox, lactam **1** (0.01 mmol, 1.9 mg) and (IPr)Ni(η^6-PhMe) (0.01 mmol, 5.4 mg) were added into a vial, followed by addition of 0.4 mL toluene-d_8. The solution was transferred into an oven-dried NMR tube and sealed for NMR timepoint analyses. Formation of the CO deinsertion product **Ni-1'** was observed after 22 hours at RT judging from the appearance of the diagnostic up-field nickel alkyl proton signals: δ 0.34 (t, J = 6.4 Hz, 2H, α-CH_2), 0.86 (m, 2H, β-CH_2). Attempts to isolate the CO deinsertion product **Ni-1'** in a pure form were unsuccessful under multiple conditions.

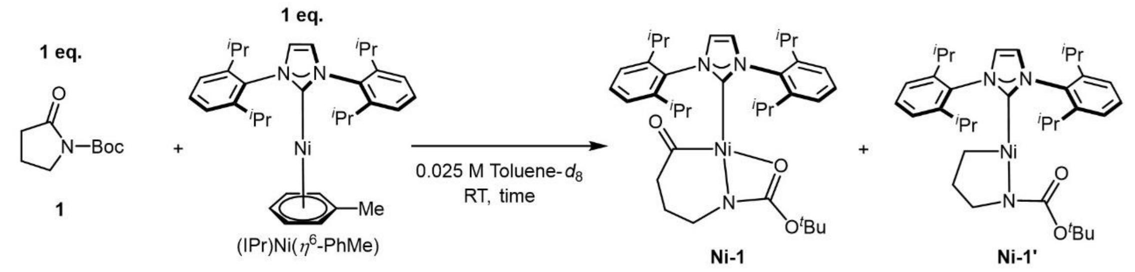

Time (h)	% remaining		% conv.	
1h	98	92	3	0
5h	78	72	24	0
22h	46	36	56	3
48h	36	17	69	8
72h	31	7	65	11
100h	28	5	63	14
192h	28	2	40	18
240h	28	1	26	25

Supplementary Figure 9. Reaction progress monitoring of **1** and (IPr)Ni(η^6-PhMe).

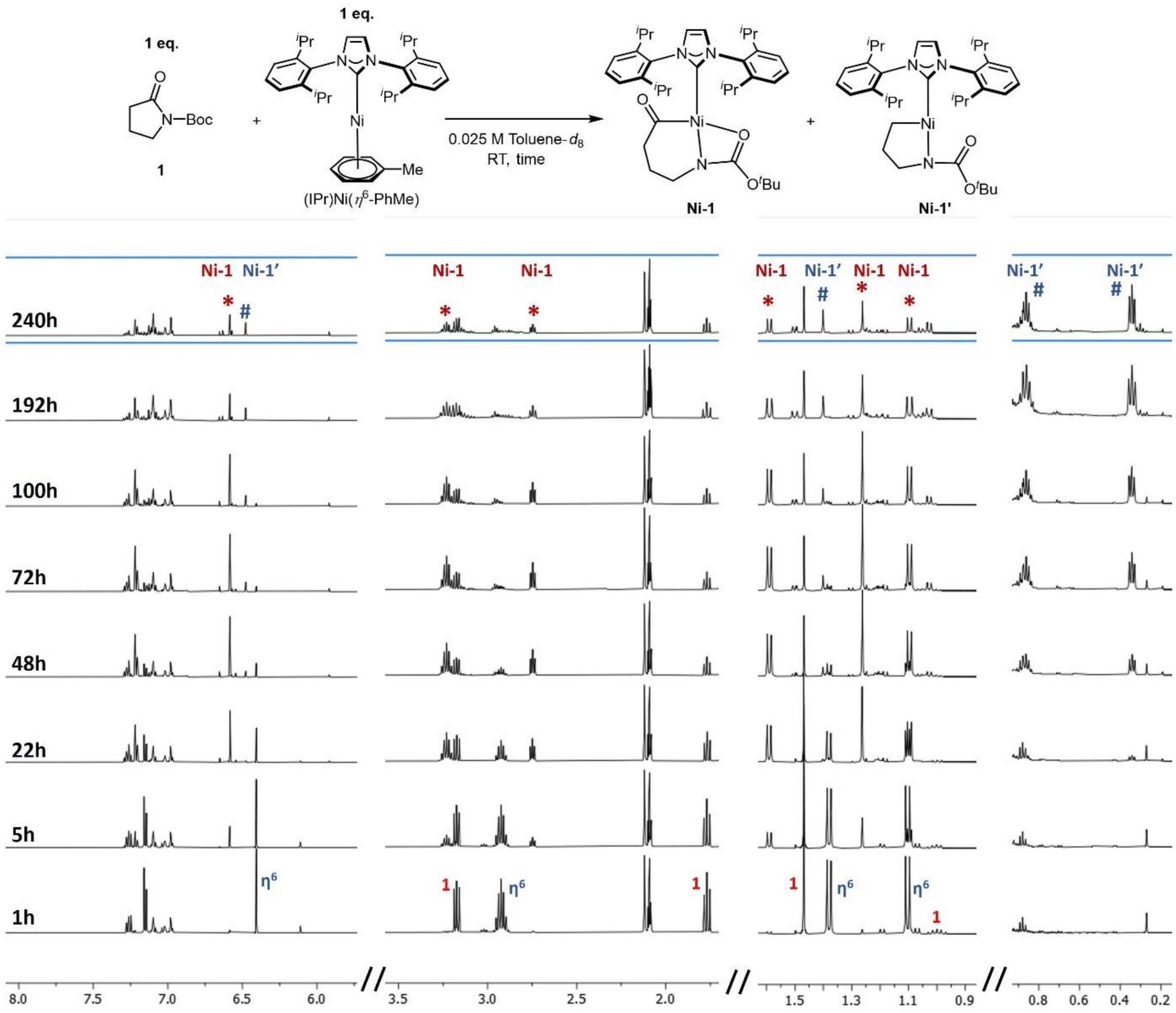

Supplementary Figure 10. Stacked ^{1}H NMR spectra of reaction progress between **1** and (IPr)Ni(η^6-PhMe).

5.22 Reaction progress monitoring of **Ni-2**

In an argon-filled glovebox, lactam **2** (0.01 mmol, 2.0 mg) and (IPr)Ni(η^6-PhMe) (0.01 mmol, 5.4 mg) were added into a vial, followed by addition of 0.4 mL toluene-d_8. The solution was transferred into an oven-dried NMR tube and sealed for NMR timepoint analyses. Formation of the proposed nickel acyl intermediate **Ni-2’** was observed after 1 hour and 4 hours at RT: δ 6.54 (s, 2H, IPr), 3.29 (hept, J = 6.8 Hz, 4H, IPr), 3.01 (m, 2H, N-CH_2), 1.69 (d, J = 6.8 Hz, 12H, IPr), 1.16 (s, 9H, *N*-Boc), 1.12 (d, J = 6.8 Hz, 12H, IPr). After 20 hours the nickel acyl intermediate **Ni-2’** was fully converted into the nickel alkyl product **Ni-2**. Slow β-H elimination of **Ni-2** was observed at room temperature after 72 hours. Conversion of lactam **2** and **Ni-2** were accelerated at 60 °C.

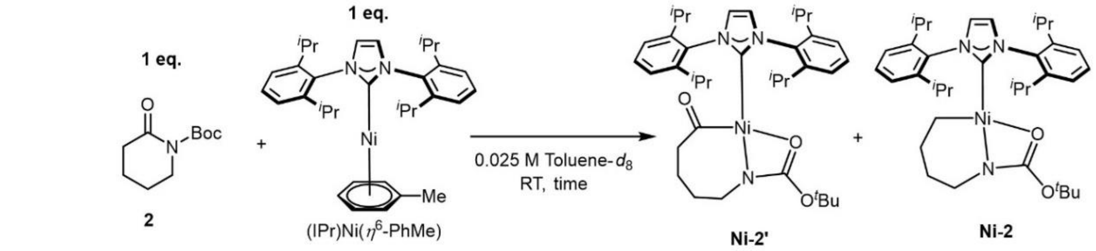

Time (h)	% remaining		% conv.	
1h	73	65	19	4
4h	48	16	19	31
20h	48	1	1	62
72h	48	0	0	56
60 °C, 18h	39	0	0	28

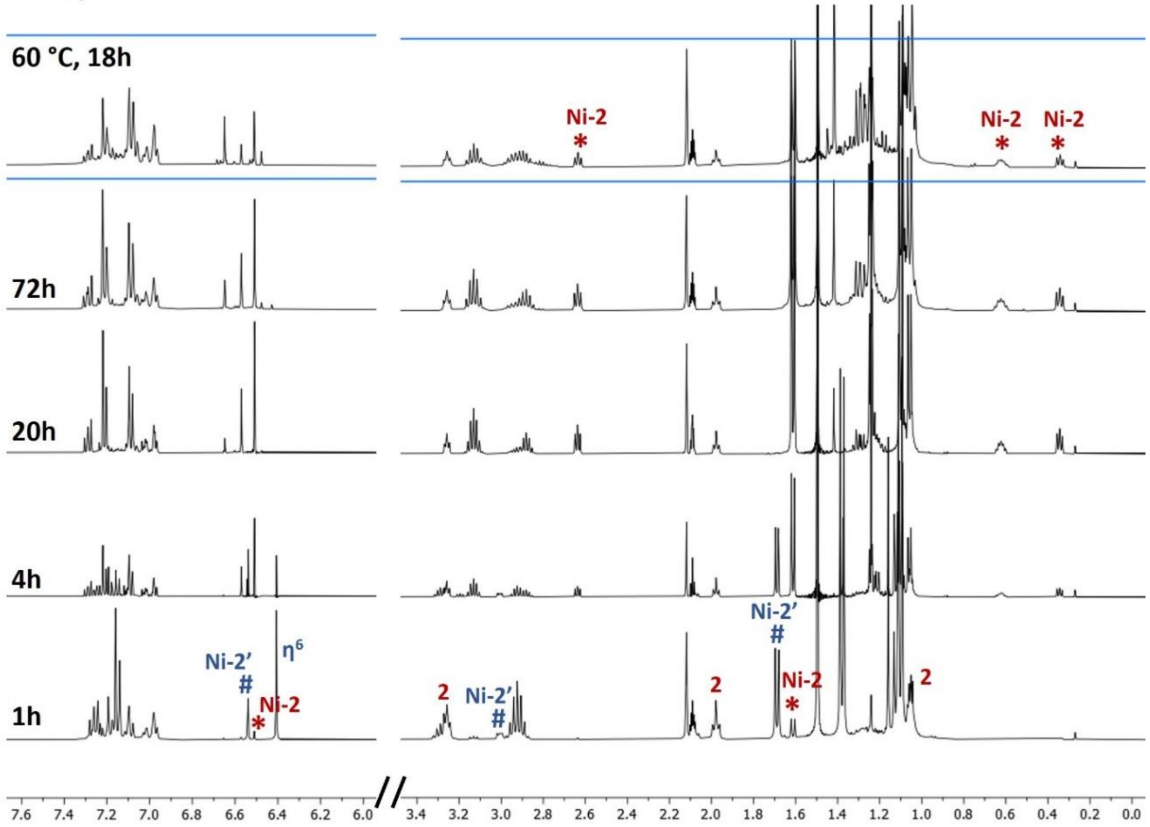

Supplementary Figure 11. Reaction progress monitoring of **2** and (IPr)Ni(η^6-PhMe).

5.23 Solution state instability of **Ni-3a**

In an argon-filled glovebox, **Ni-3a** (0.01 mmol, 6.8 mg) was added into a vial, followed by addition of 0.4 mL benzene-d_6. The solution was transferred into an oven-dried NMR tube and sealed for NMR analyses. An initial ^{1}H NMR spectrum was taken after 1 hour at RT. After 5 days at RT, a mixture of **Ni-3a** (57.6%), **Ni-3b** (31.0%), (IPr)Ni(CO)$_3$ (11.4%) and lactam **3** (12.8%) were all identified by ^{1}H NMR spectroscopy. A proposed reaction sequence accounting for the observed solution reactivity of **Ni-3a** is shown below.

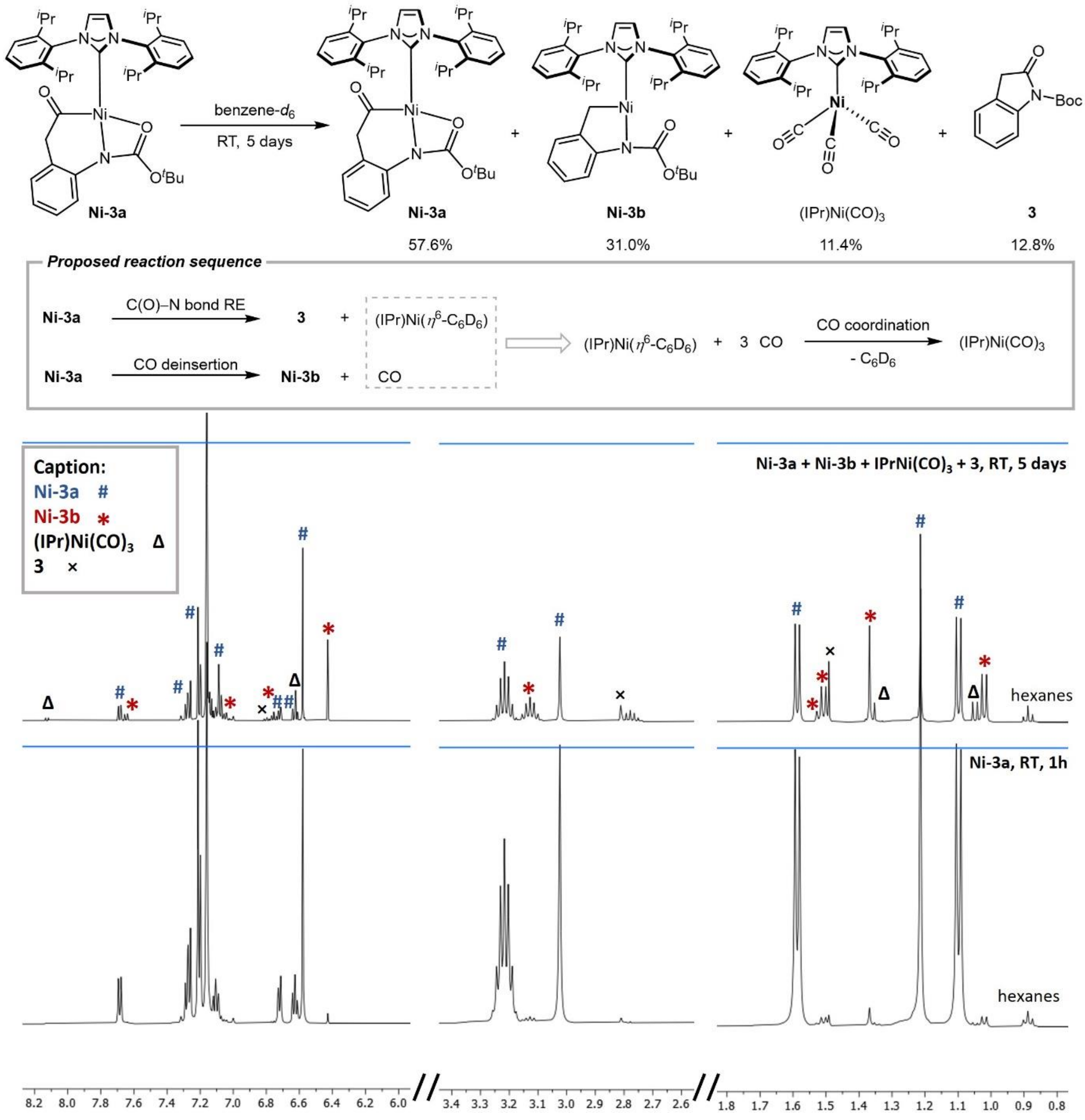

Supplementary Figure 12. Solution state instability of **Ni-3a** in benzene-d_6 monitored by ^{1}H NMR spectroscopy.

5.24 Reaction progress monitoring of **Ni-4**

In an argon-filled glovebox, lactam **4** (0.01 mmol, 1.7 mg) and (IPr)Ni(η^6-PhMe) (0.01 mmol, 5.4 mg) were added into a vial, followed by addition of 0.4 mL toluene-d_8. The solution was transferred into an oven-dried NMR tube and sealed for NMR timepoint analyses. Formation of the 14-electron nickel acyl complex **Ni-4** was observed at 1.5h after mixing (15% conv.) and did not reach full conversion after 16h (84% conv.). The formation of isobutene from Boc group decomposition was detected: δ 4.71 (2H, br), 1.60 (6H, overlapping).

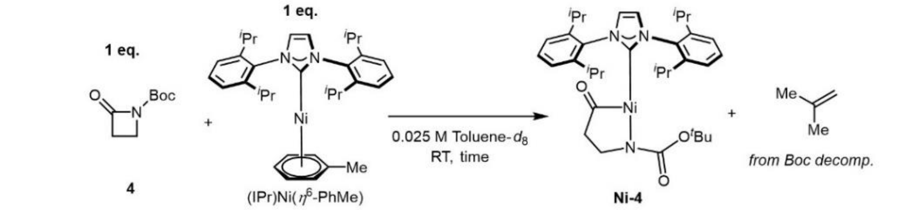

Time (h)	% remaining			% conv.
1.5h	85	87	15	0
16h	21	17	84	1.5
48h	0	0	98	2.5
312h	0	0	93	6.5

Proposed Boc decomposition pathway:

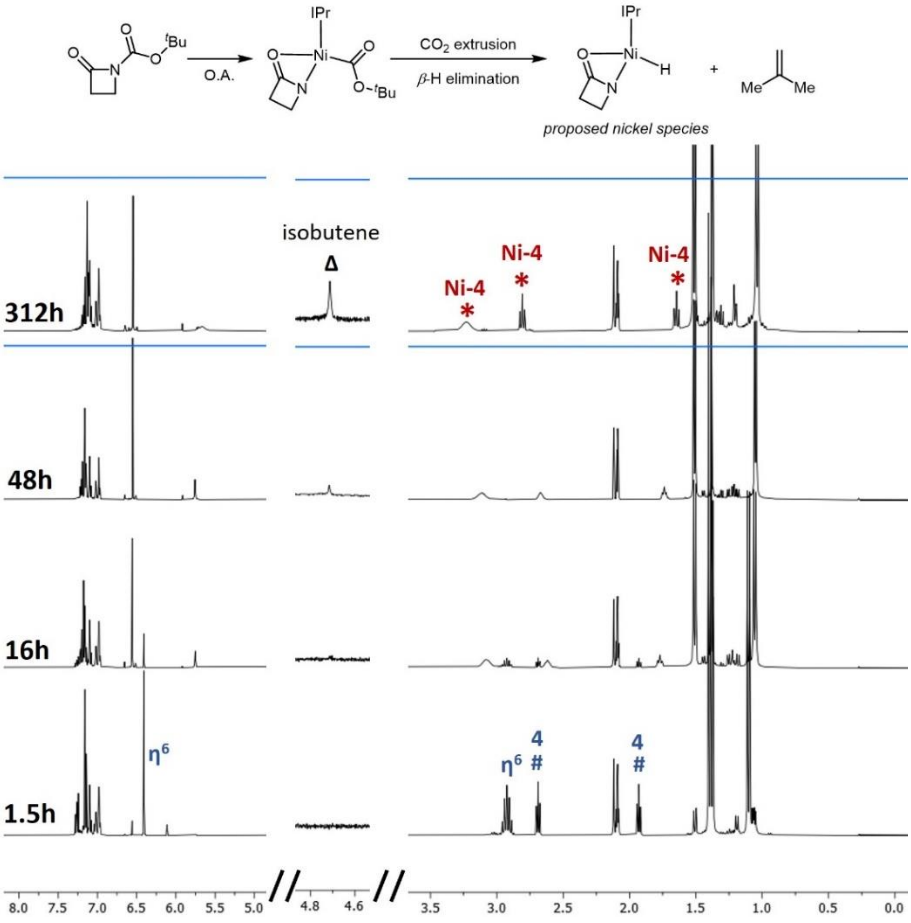

Supplementary Figure 13. Stacked ^{1}H NMR spectra of reaction progress between **4** and (IPr)Ni(η^6-PhMe).

5.25 Reaction progress monitoring of **Ni-5**

In an argon-filled glovebox, lactam **5** (0.01 mmol, 2.0 mg) and (IPr)Ni(η^6-PhMe) (0.01 mmol, 5.4 mg) were added into a vial, followed by addition of 0.4 mL toluene-d_8. The solution was transferred into an oven-dried NMR tube and sealed for NMR timepoint analyses. Formation of the CO deinsertion product **Ni-2'** was not observed judging from the absence of the nickel alkyl protons at the α-position in the 0 – 0.5 ppm region. Unidentified nickel complex(es) were formed. The low molecular symmetry of nickel complexes and diastereotopic proton signals arising from the stereocenter in **5** complicated the ^{1}H NMR analysis.

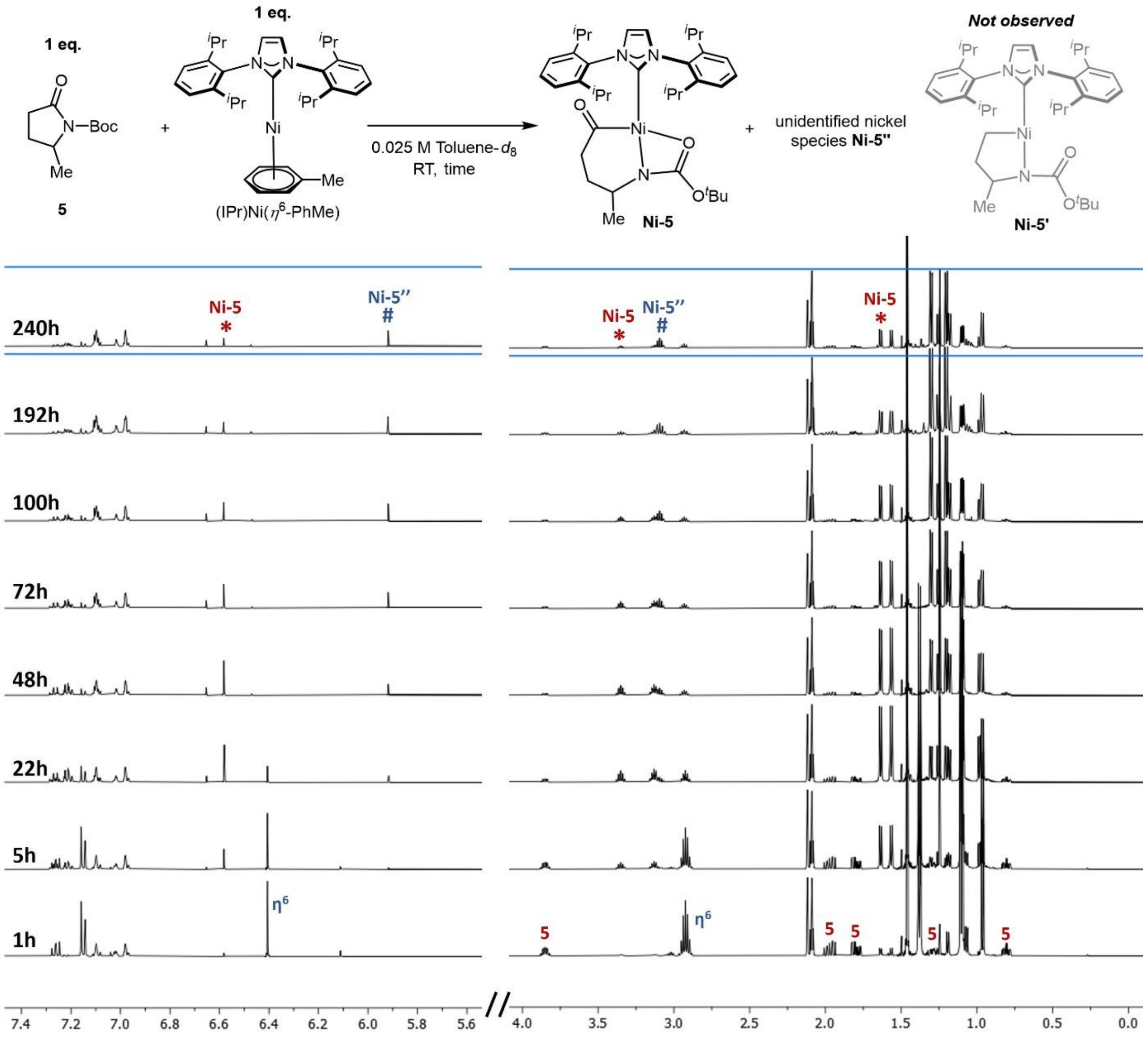

Supplementary Figure 14. Stacked ^{1}H NMR spectra of reaction progress between **5** and (IPr)Ni(η^6-PhMe).

5.26 Reaction progress monitoring of **Ni-12**

In an argon-filled glovebox, lactam **12** (0.01 mmol, 2.5 mg) and (IPr)Ni(η^6-PhMe) (0.01 mmol, 5.4 mg) were added into a vial, followed by addition of 0.4 mL toluene-d_8. The solution was transferred into an oven-dried NMR tube and sealed for NMR timepoint analyses. Formation of the CO deinserted nickel alkyl product **Ni-12** (68%) was observed after 1 hour upon mixing. The formation of (IPr)Ni(CO)$_3$ (22%) was also observed. The reaction reached completion (75% **Ni-12**) within 40 hours at RT. The contrasting NMR yield and the isolated yield of **Ni-12** (98%) on a preparative scale indicate that the reaction headspace could largely influence the product speciation from reaction of **12**.

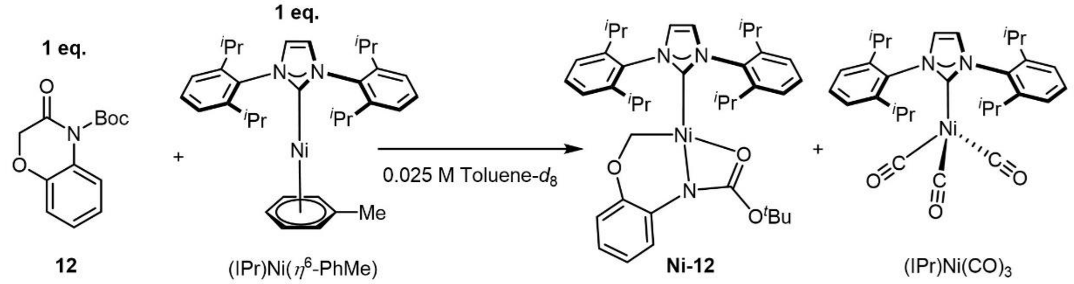

Condition	% remaining		% conv.	
**RT, 1h**	14	3	68	22
**RT, 40h**	14	0	75	23

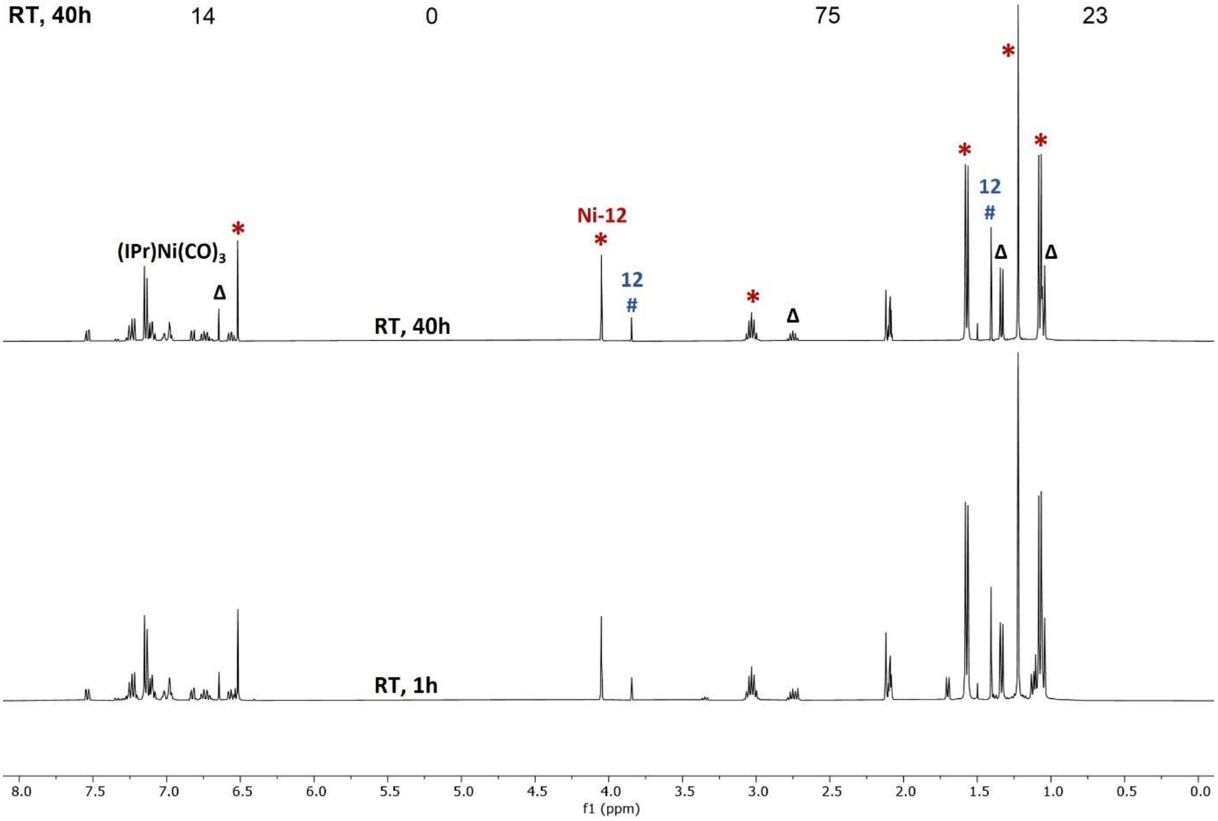

Supplementary Figure 15. Stacked ^{1}H NMR spectra of reaction progress between **12** and (IPr)Ni(η^6-PhMe).

5.27 Reaction progress monitoring of **Ni-14**

In an argon-filled glovebox, lactam **14** (0.01 mmol, 2.5 mg) and (IPr)Ni(η^6-PhMe) (0.01 mmol, 5.4 mg) were added into a vial, followed by addition of 0.4 mL toluene-d_8. The solution was transferred into an oven-dried NMR tube and sealed for NMR timepoint analyses. Formation of **Ni-14** (71%) and (IPr)Ni$(CO)_3$ (23%) was observed after 1 hour upon mixing.

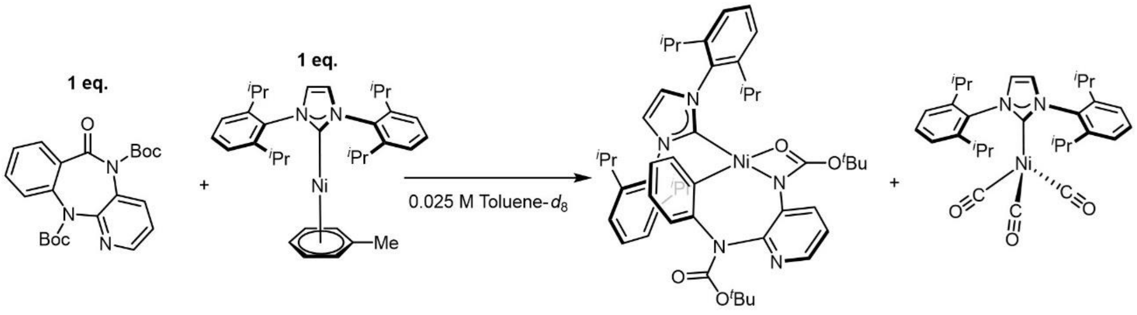

Condition	14 (% remaining)	(IPr)Ni(η^6-PhMe) (% remaining)	Ni-14 (% conv.)	(IPr)Ni$(CO)_3$ (% conv.)
RT, 1h	27	6	71	23
RT, 3.5h	24	5	72	23
RT, 17.5h	23	0	74	24

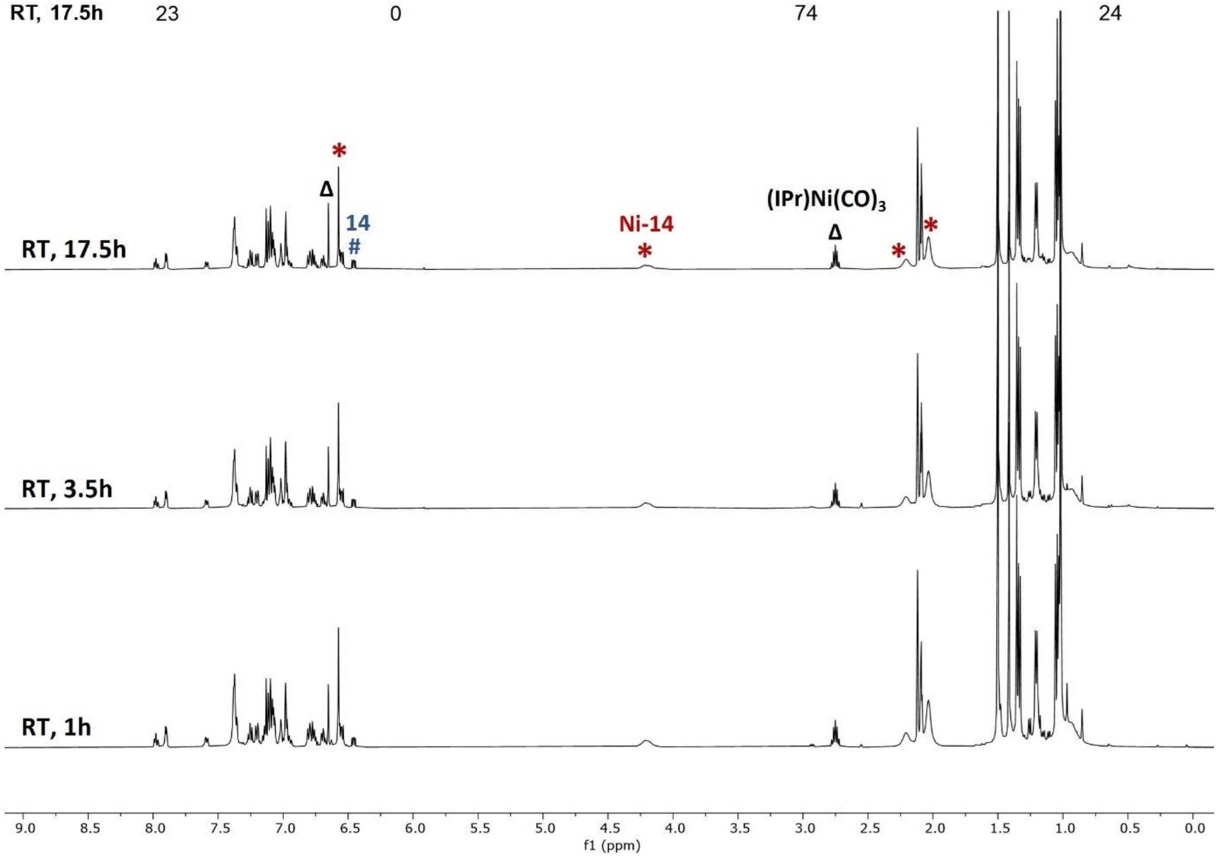

Supplementary Figure 16. Stacked ^{1}H NMR spectra of reaction progress between **14** and (IPr)Ni(η^6-PhMe).

5.28 Reaction progress monitoring of **Ni-15**

In an argon-filled glovebox, lactam **15** (0.01 mmol, 3.2 mg) and (IPr)Ni(η^6-PhMe) (0.01 mmol, 5.4 mg) were added into a vial, followed by addition of 0.4 mL toluene-d_8. The solution was transferred into an oven-dried NMR tube and sealed for NMR timepoint analyses. Formation of a nickel intermediate (61%, likely (IPr)Ni(η^2-**15**) judging from the upfield-shifted alkene ^{1}H NMR signals) and **Ni-15** (13%) was observed after 1 hour upon mixing. After 44.5h at RT, Formation of **Ni-15** (60%) and (IPr)Ni(CO)$_3$ (19%) was observed.

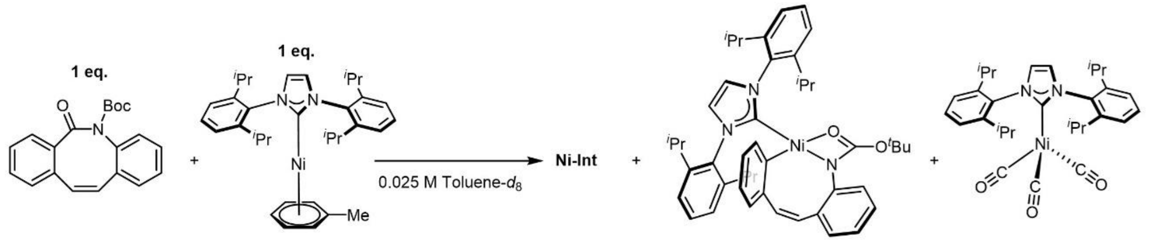

Condition	15	(IPr)Ni(η^6-PhMe)	Ni-Int	Ni-15	(IPr)Ni(CO)$_3$
	% remaining		% conv.		
RT, 1h	0	24	61	13	0
RT, 17h	0	0	0	50	14
RT, 44.5h	0	0	0	60	19

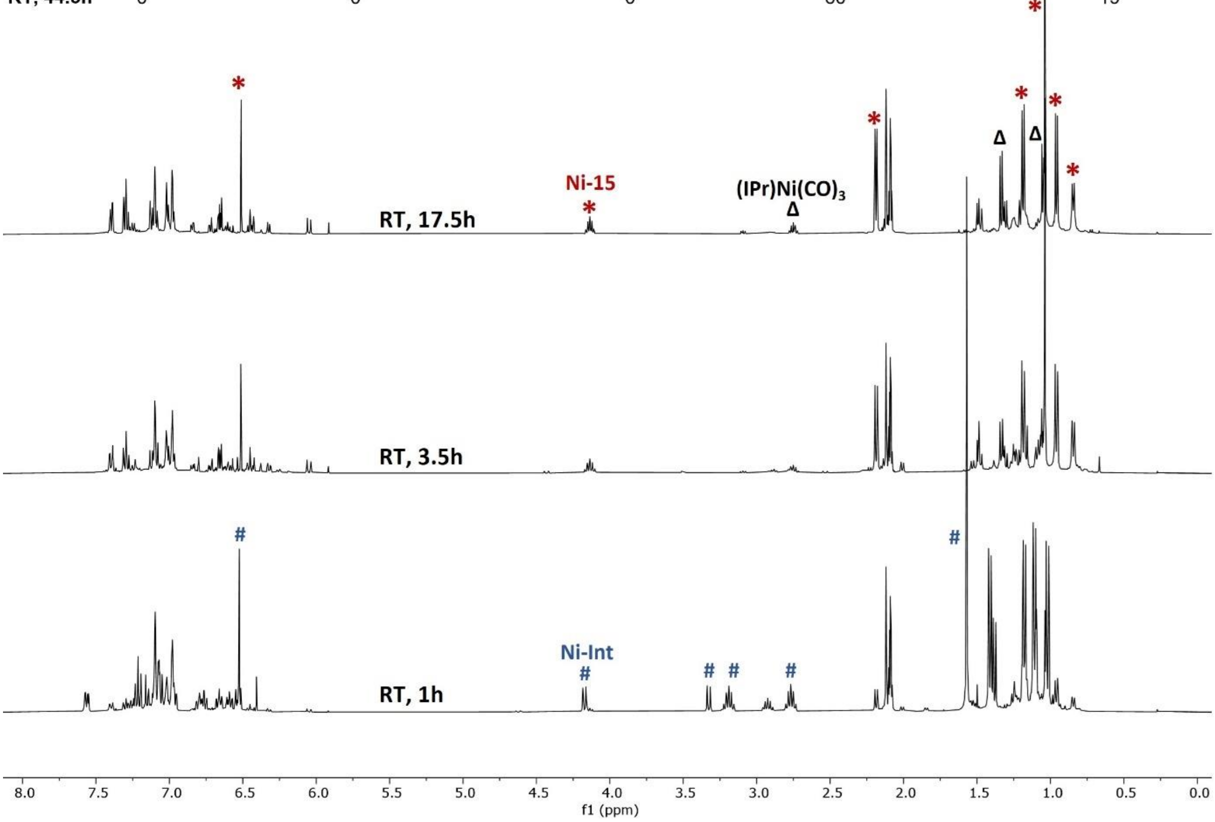

Supplementary Figure 17. Stacked ^{1}H NMR spectra of reaction progress between **15** and (IPr)Ni(η^6-PhMe).

5.29 Reaction progress monitoring of **Ni-19**

In an argon-filled glovebox, aza-steroid **19** (0.01 mmol, 4.3 mg) and (IPr)Ni(η^6-PhMe) (0.01 mmol, 5.4 mg) were added into a vial, followed by addition of 0.4 mL benzene-d_6. The solution was transferred into an oven-dried NMR tube and sealed for NMR timepoint analyses. Formation of the acyl nickellacycle **Ni-19'** (6%) was observed after 1h upon mixing. After 4.5h at RT, **Ni-19'** (24%) and the alkyl nickellacycle **Ni-19** (15%) were formed. Heating at 40 °C for 47h afforded **Ni-19** in 43% conversion.

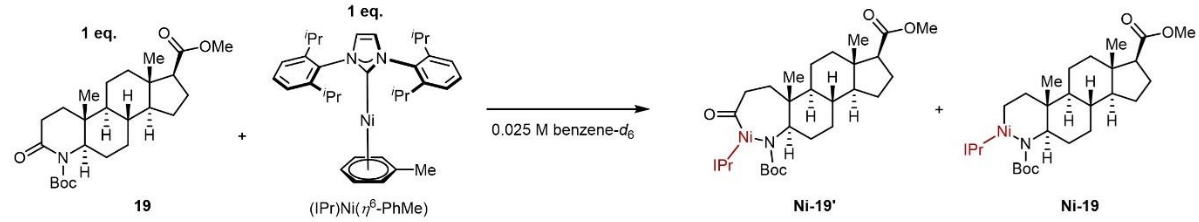

Condition	19	(IPr)Ni(η^6-PhMe)	Ni-19'	Ni-19
	% remaining		% conv.	
RT, 1h	94	70	6	0
RT, 2h	84	58	14	2
RT, 4.5h	61	16	24	15
RT, 47.5h	59	0	4	37
40°C, 22h	58	0	0	42
40°C, 47h	56	0	0	43

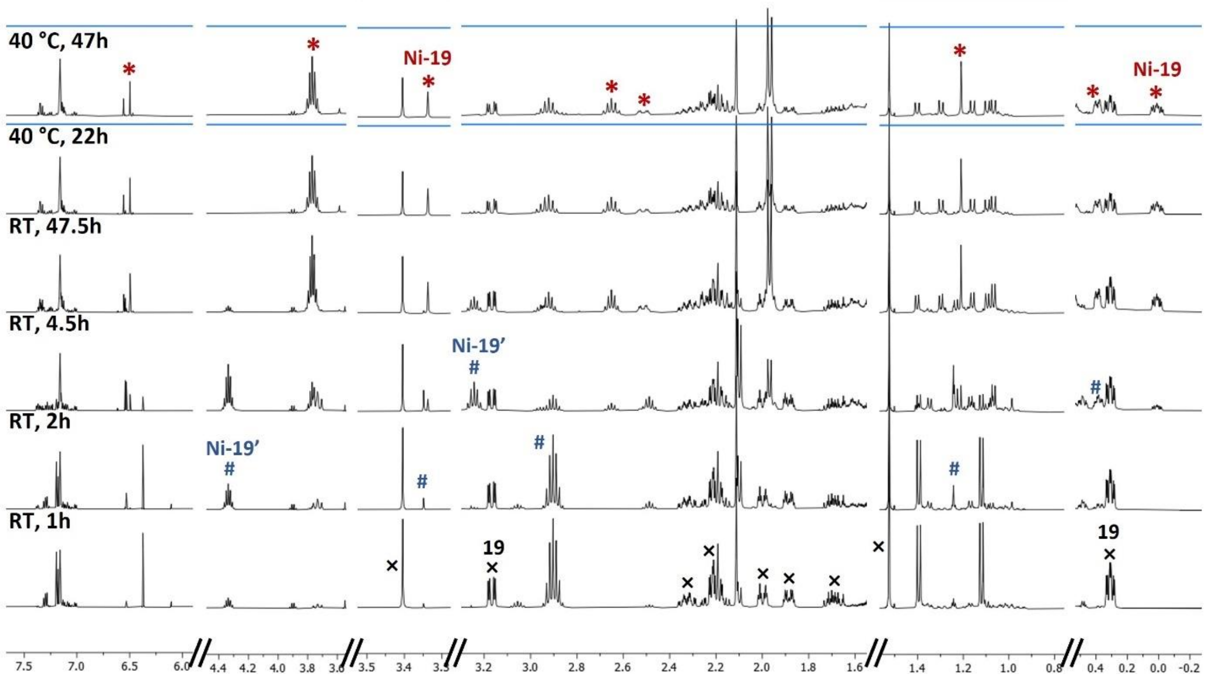

Supplementary Figure 18. Stacked ^{1}H NMR spectra of reaction progress between **19** and (IPr)Ni(η^6-PhMe).

5.3. *β*-hydride elimination of metallacycles

The *β*-hydride elimination of metallacycles **Ni-2**, **Ni-16**, **Ni-17** and **Ni-18** generated from the corresponding aliphatic lactams **2**, **16**, **17** and **18** were investigated. From metallacycle ring sizes of 6, 7, 8 to 9, the rate of *β*-hydride elimination is increasing, giving higher conversions to the ring-opened terminal alkene products which were quantitated by treatment with CO(g) that induced the N–H bond reductive elimination and product release.

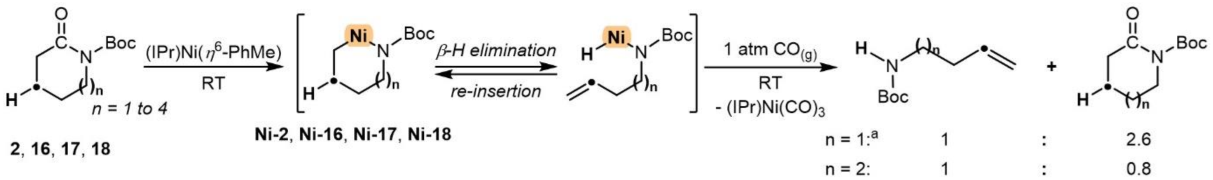

n = 1:[a]	1	:	2.6
n = 2:	1	:	0.8
n = 3:	1	:	0.6
n = 4:	1	:	0.2

Supplementary Figure 19. *β*-hydride elimination of metallacycles from 6- to 9-membered aliphatic lactams. a. 60 °C, 18h.

5.31 *β*-hydride elimination of **Ni-2**

In an argon-filled glovebox, lactam **2** (0.01 mmol, 2.0 mg) and (IPr)Ni(η^6-PhMe) (0.01 mmol, 5.4 mg) were added into a vial, followed by addition of 0.4 mL toluene-d_8. The solution was transferred into an oven-dried NMR tube and sealed. After 72 hours at RT and 18 hours at 60 °C heating, formation of **Ni-2** (28%) and a proposed nickel hydride species from *β*-H elimination of **Ni-2''** (21%) were observed by ^{1}H NMR spectroscopy. The headspace was evacuated and the reaction was treated with 1 atm of $CO_{(g)}$, affording lactam **2** (61%), terminal alkene **2'** (24%) and (IPr)Ni$(CO)_3$ (86%). The ^{1}H NMR chemical shifts of **2'** matched that of an authentic sample.

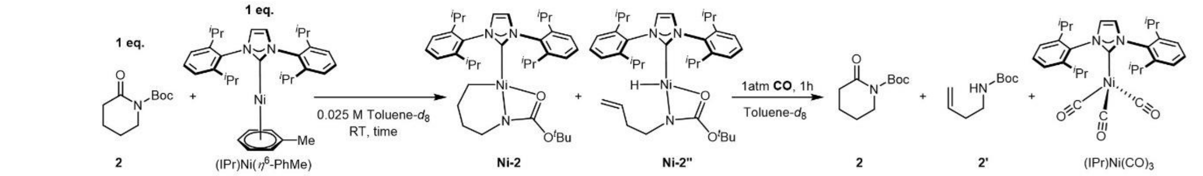

Time (h)	2	(IPr)Ni(η^6-PhMe)	Ni-2	Ni-2''	2	2'	(IPr)Ni(CO)$_3$
	% remaining		% conv.			% conv.	
RT, 72h	48	0	56	15	-	-	-
60 °C, 18h	39	0	28	21	-	-	-
1atm CO, 1h	-	-	-	-	61	24	86

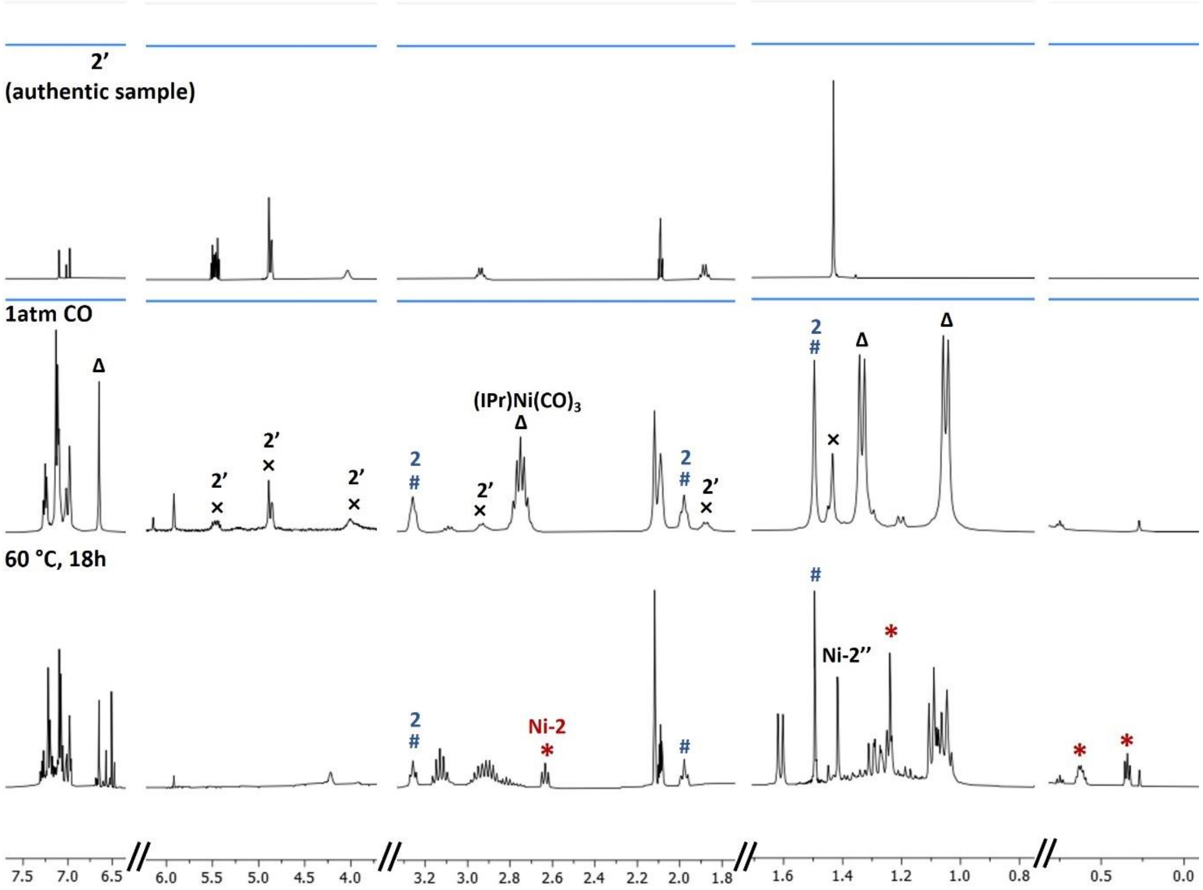

Supplementary Figure 20. Stacked ^{1}H NMR spectra of reaction between **2** and (IPr)Ni(η^6-PhMe) and treatment with $CO_{(g)}$.

5.32 β-hydride elimination of **Ni-16**

In an argon-filled glovebox, lactam **16** (0.01 mmol, 2.1 mg) and (IPr)Ni(η^6-PhMe) (0.01 mmol, 5.4 mg) were added into a vial, followed by addition of 0.4 mL toluene-d_8. The solution was transferred into an oven-dried NMR tube and sealed. After 67 hours at RT, formation of **Ni-16** (7%) and a proposed nickel hydride species from β-H elimination of **Ni-16'** (21%) were observed by ^{1}H NMR spectroscopy. The headspace was evacuated and the reaction was treated with 1atm of $CO_{(g)}$, affording lactam **16** (28%), terminal alkene **16'**(34%) and (IPr)Ni(CO)$_3$ (95%).

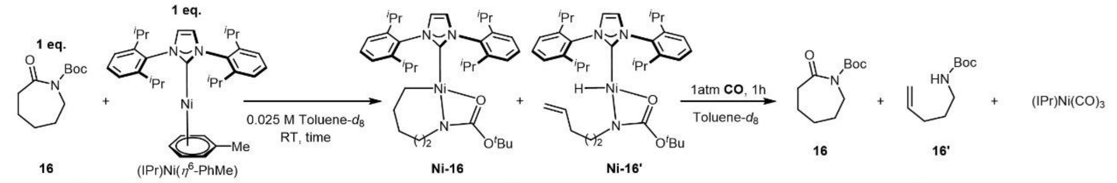

Time (h)	% remaining		% conv.		% conv.		
RT, 1h	72	72	6	0	-	-	-
RT, 2.5h	38	0	38	24	-	-	-
RT, 67h	24	0	7	44	-	-	-
1atm CO, 1h	-	-	-	-	28	34	95

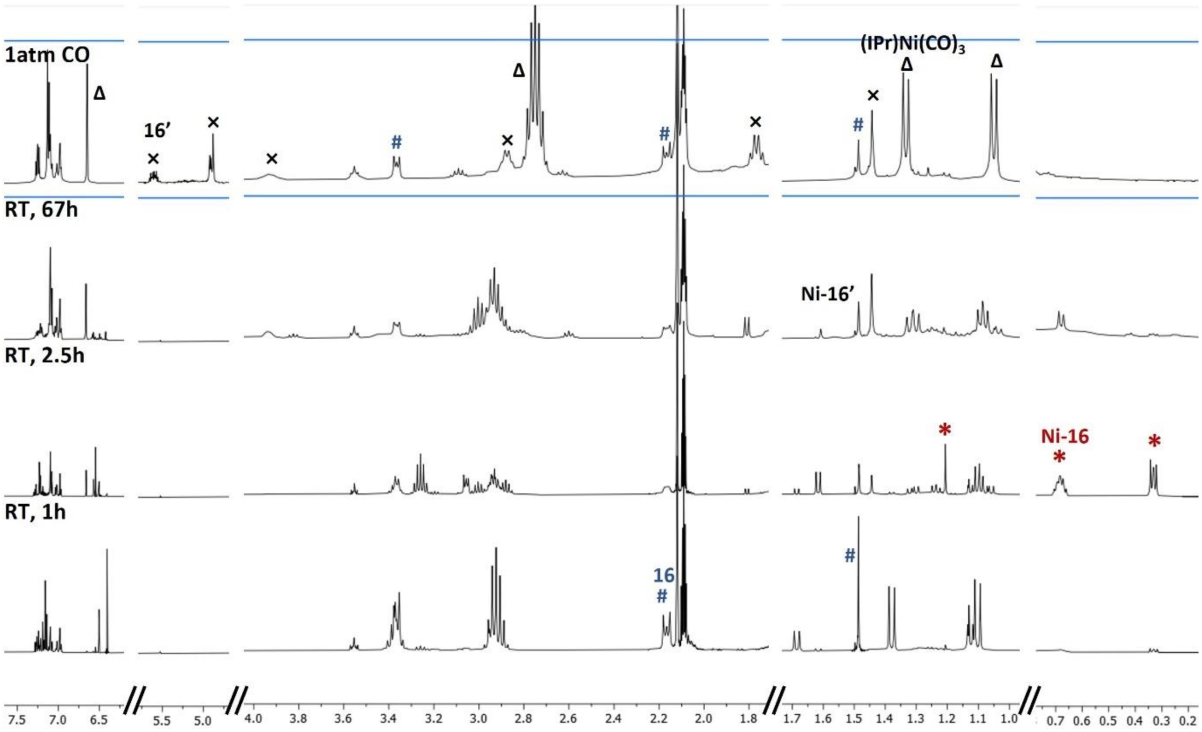

Supplementary Figure 21. Stacked ^{1}H NMR spectra of reaction between **16** and (IPr)Ni(η^6-PhMe) and treatment with $CO_{(g)}$.

5.33 *β*-hydride elimination of **Ni-17**

In an argon-filled glovebox, lactam **17** (0.01 mmol, 2.2 mg) and (IPr)Ni(η^6-PhMe) (0.01 mmol, 5.4 mg) were added into a vial, followed by addition of 0.4 mL toluene-d_8. The solution was transferred into an oven-dried NMR tube and sealed. After 67 hours at RT, formation of **Ni-17** (2%) and a proposed nickel hydride species from *β*-H elimination of **Ni-17’** (62%) were observed by ^{1}H NMR spectroscopy. The headspace was evacuated and the reaction was treated with 1atm of $CO_{(g)}$, affording lactam **17** (18%), terminal alkene **17’**(30%) and (IPr)Ni$(CO)_3$ (94%).

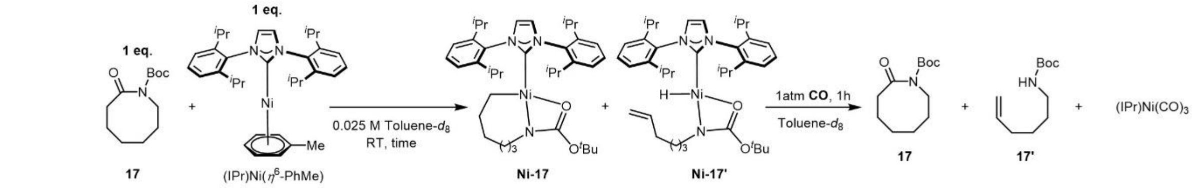

Time (h)	% remaining		% conv.				
	17	(IPr)Ni(η^6-PhMe)	Ni-17	Ni-17'	17	17'	(IPr)Ni(CO)3
RT, 1h	80	77	20	0	-	-	-
RT, 2.5h	26	16	4	58	-	-	-
RT, 67h	20	0	2	62	-	-	-
1atm CO, 1h	-	-	-	-	18	30	94

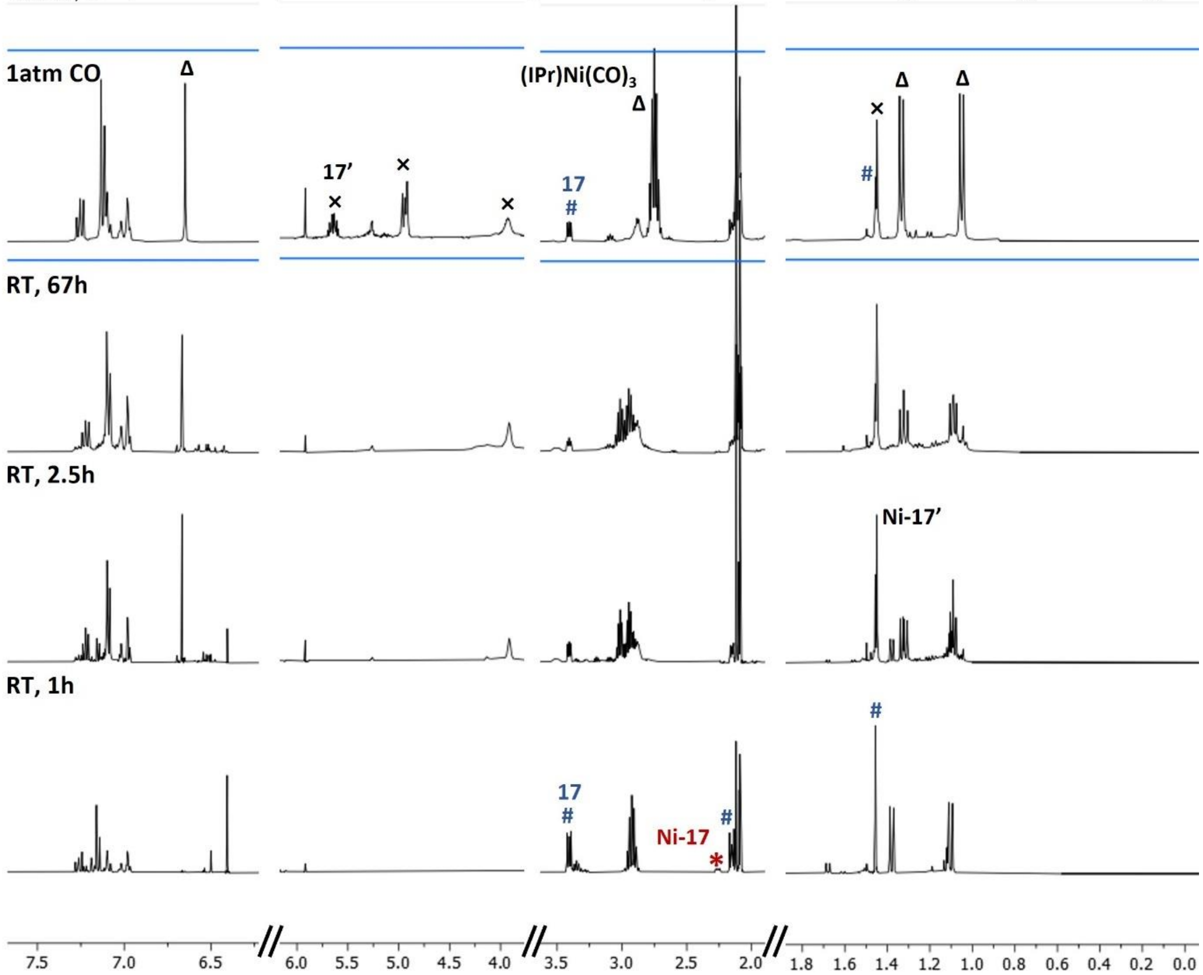

Supplementary Figure 22. Stacked ^{1}H NMR spectra of reaction between **17** and (IPr)Ni(η^6-PhMe) and treatment with $CO_{(g)}$.

5.34 β-hydride elimination of **Ni-18**

In an argon-filled glovebox, lactam **18** (0.01 mmol, 2.4 mg) and (IPr)Ni(η^6-PhMe) (0.01 mmol, 5.4 mg) were added into a vial, followed by addition of 0.4 mL toluene-d_8. The solution was transferred into an oven-dried NMR tube and sealed. After 67 hours at RT, formation of **Ni-18** (2%) and a proposed nickel hydride species from β-H elimination of **Ni-18'** (29%) were observed by ^{1}H NMR spectroscopy. The headspace was evacuated and the reaction was treated with 1atm of $CO_{(g)}$, affording lactam **18** (5%), terminal alkene **18'**(26%) and (IPr)Ni(CO)$_3$ (88%).

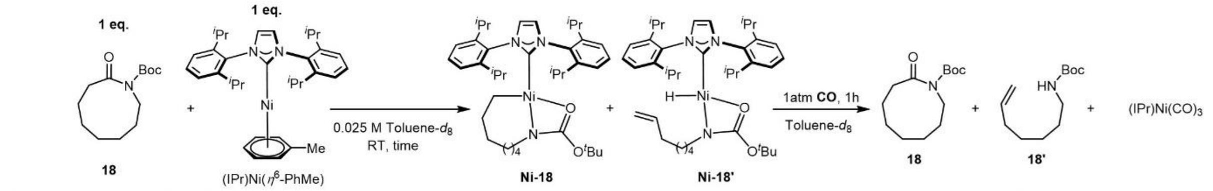

Time (h)	% remaining		% conv.			% conv.	
RT, 1h	42	35	64	0	-	-	-
RT, 2.5h	8	0	63	8	-	-	-
RT, 67h	5	0	0	29	-	-	-
1atm CO, 1h	-	-	-	-	5	26	88

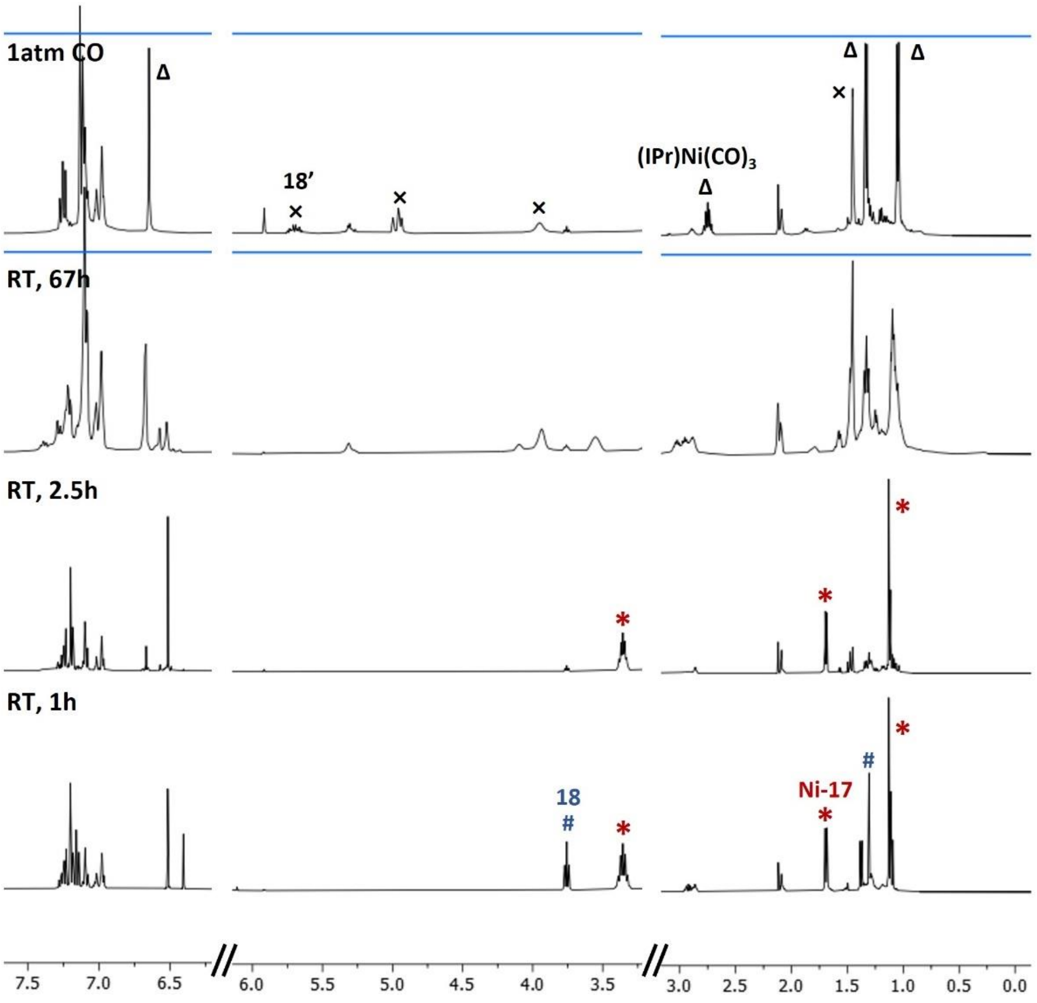

Supplementary Figure 23. Stacked ^{1}H NMR spectra of reaction between **18** and (IPr)Ni(η^6-PhMe) and treatment with $CO_{(g)}$.

5.4 Reactions between metallacycles and $CO_{(g)}$

General procedure for reactions between metallacycles and $CO_{(g)}$

In an argon-filled glovebox, *N*–Boc lactam substrate (0.01 mmol) and (IPr)Ni(η^6-PhMe) (0.01 mmol) were added into a vial, followed by addition of 0.4 mL toluene-d_8 or benzene-d_6. The solution was transferred into an oven-dried NMR tube and sealed. After confirmation of metallacycle formation by ^{1}H NMR spectroscopy, the headspace of the NMR tube was evacuated, followed by introduction of 1atm of $CO_{(g)}$ and analyzed by ^{1}H NMR spectroscopy.

5.41 Reaction progress monitoring of **Ni-6** and reaction with $CO_{(g)}$

In an argon-filled glovebox, lactam **6** (0.01 mmol, 2.1 mg) and (IPr)Ni(η^6-PhMe) (0.01 mmol, 5.4 mg) were added into a vial, followed by addition of 0.4 mL toluene-d_8. The solution was transferred into an oven-dried NMR tube and sealed. After heating at 60 °C and formation of **Ni-6** (39%), the headspace was evacuated, and 1atm of $CO_{(g)}$ was introduced. A 1:1 mixture of lactam **6** and (IPr)Ni$(CO)_3$ was obtained quantitatively.

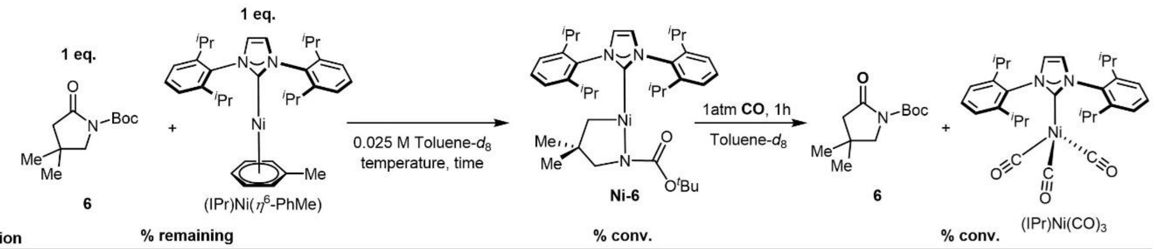

Condition	% remaining		% conv.	% conv.	
RT, 18h	95	88	0	-	-
60 °C, 18h	46	2	39	-	-
1atm CO, 1h	-	-	-	>98	>98

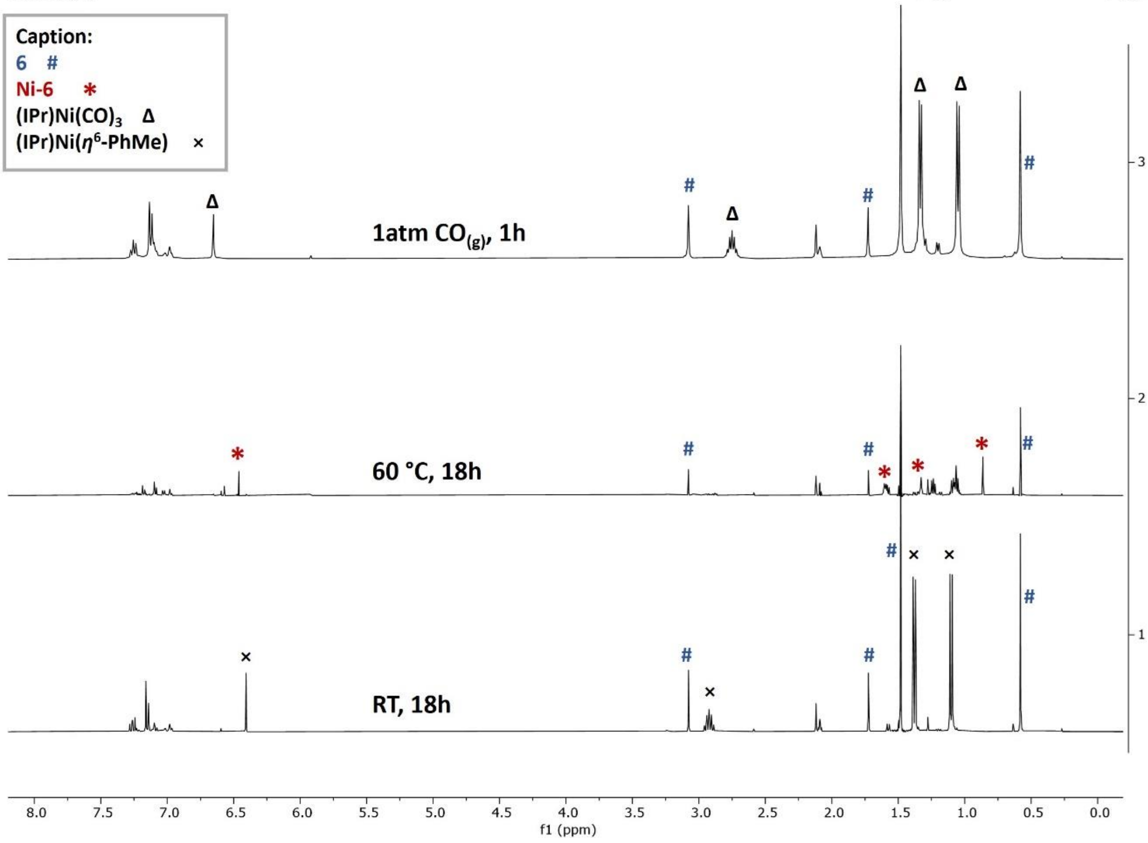

Supplementary Figure 24. Stacked ^{1}H NMR spectra of reaction progress between **6** and (IPr)Ni(η^6-PhMe) and treatment with $CO_{(g)}$.

5.42 Reaction progress monitoring of **Ni-9** and reaction with $CO_{(g)}$

In an argon-filled glovebox, lactam **9** (0.01 mmol, 2.4 mg) and (IPr)Ni(η^6-PhMe) (0.01 mmol, 5.4 mg) were added into a vial, followed by addition of 0.4 mL toluene-d_8. The solution was transferred into an oven-dried NMR tube and sealed. The reaction progress was monitored by ^{1}H NMR spectroscopy. The formation of **Ni-9** (76%) and (IPr)Ni$(CO)_3$ (31%) were confirmed before addition of $CO_{(g)}$, suggesting the CO from deinsertion were captured by (IPr)Ni(0) that formed (IPr)Ni$(CO)_3$ in this reaction. The headspace was evacuated, and 1atm of $CO_{(g)}$ was introduced. Formation of lactam **9** (58%) and (IPr)Ni$(CO)_3$ (65%) were identified.

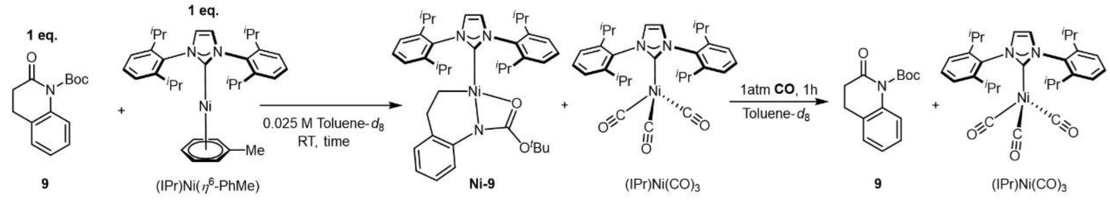

Condition	% remaining		% conv.		% conv.	
RT, 1h	86	79	14	0	-	-
RT, 4h	42	5	58	5	-	-
RT, 20h	29	0	71	22	-	-
RT, 72h	26	0	74	30	-	-
60 °C, 18h	24	0	76	31	-	-
1atm CO, 1h	-	-	-	-	58	65

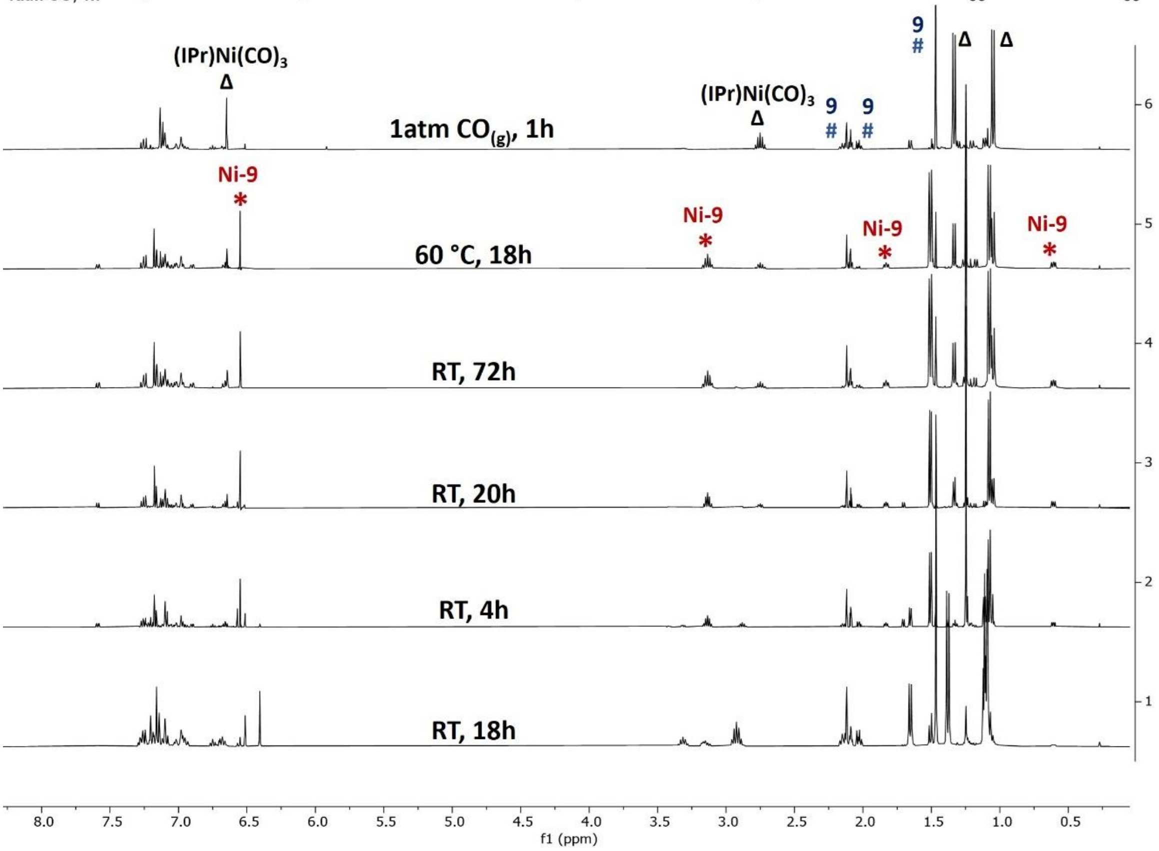

Supplementary Figure 25. Stacked ^{1}H NMR spectra of reaction progress between **9** and (IPr)Ni(η^6-PhMe) and treatment with $CO_{(g)}$.

5.43 Reaction progress monitoring of **Ni-10** and reaction with $CO_{(g)}$

In an argon-filled glovebox, lactam **10** (0.01 mmol, 2.4 mg) and (IPr)Ni(η^6-PhMe) (0.01 mmol, 5.4 mg) were added into a vial, followed by addition of 0.4 mL toluene-d_8. The solution was transferred into an oven-dried NMR tube and sealed. The reaction progress was monitored by ^{1}H NMR spectroscopy. The formation of **Ni-10** (61%) was confirmed before addition of $CO_{(g)}$. The headspace was evacuated, and 1 atm of $CO_{(g)}$ was introduced. Formation of lactam **10** (>98%) and (IPr)Ni$(CO)_3$ (>98%) were identified.

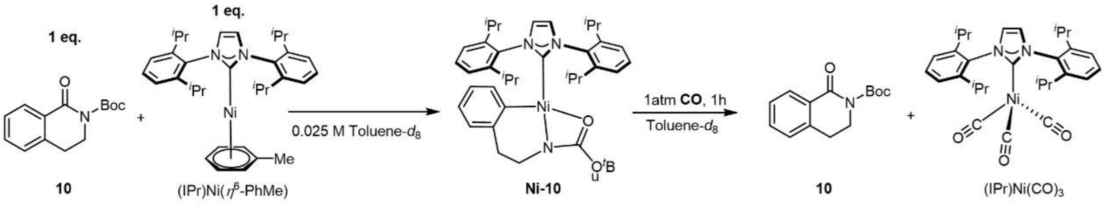

Condition	% remaining		% conv.	% conv.	
RT, 1h	58	40	9	-	-
RT, 16h	41	0	41	-	-
RT, 62h	40	0	45	-	-
60 °C, 18h	32	0	61	-	-
1atm CO, 1h	-	-	-	>98	>98

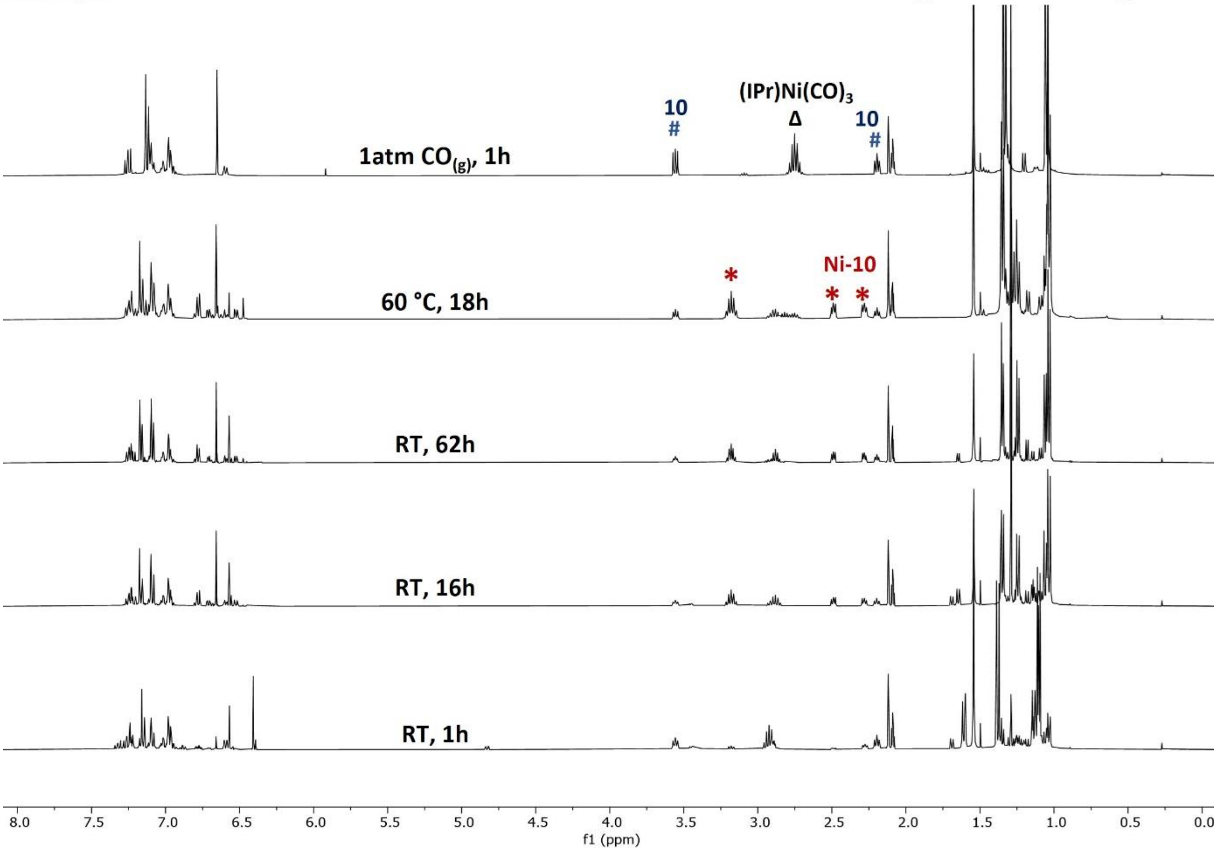

Supplementary Figure 26. Stacked ^{1}H NMR spectra of reaction progress between **10** and (IPr)Ni(η^6-PhMe) and treatment with $CO_{(g)}$.

5.44 Reaction progress monitoring of **Ni-11** and reaction with $CO_{(g)}$

In an argon-filled glovebox, lactam **11** (0.01 mmol, 2.0 mg) and (IPr)Ni(η^6-PhMe) (0.01 mmol, 5.4 mg) were added into a vial, followed by addition of 0.4 mL toluene-d_8. The solution was transferred into an oven-dried NMR tube and sealed. The reaction progress was monitored by ^{1}H NMR spectroscopy. The formation of **Ni-11** (48%) and (IPr)Ni(CO)$_3$ (14%) were confirmed before addition of $CO_{(g)}$. The headspace was evacuated, and 1atm of $CO_{(g)}$ was introduced. Formation of lactam **11** (61%) and (IPr)Ni(CO)$_3$ (62%) were identified.

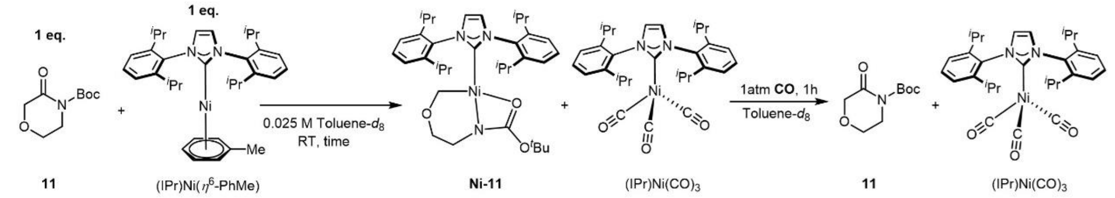

Condition	% remaining		% conv.		% conv.	
RT, 3h	86	5	57	3	-	-
RT, 17.5h	79	0	59	4	-	-
60 °C, 18h	47	0	48	14	-	-
1atm CO, 1h	-	-	-	-	61	62

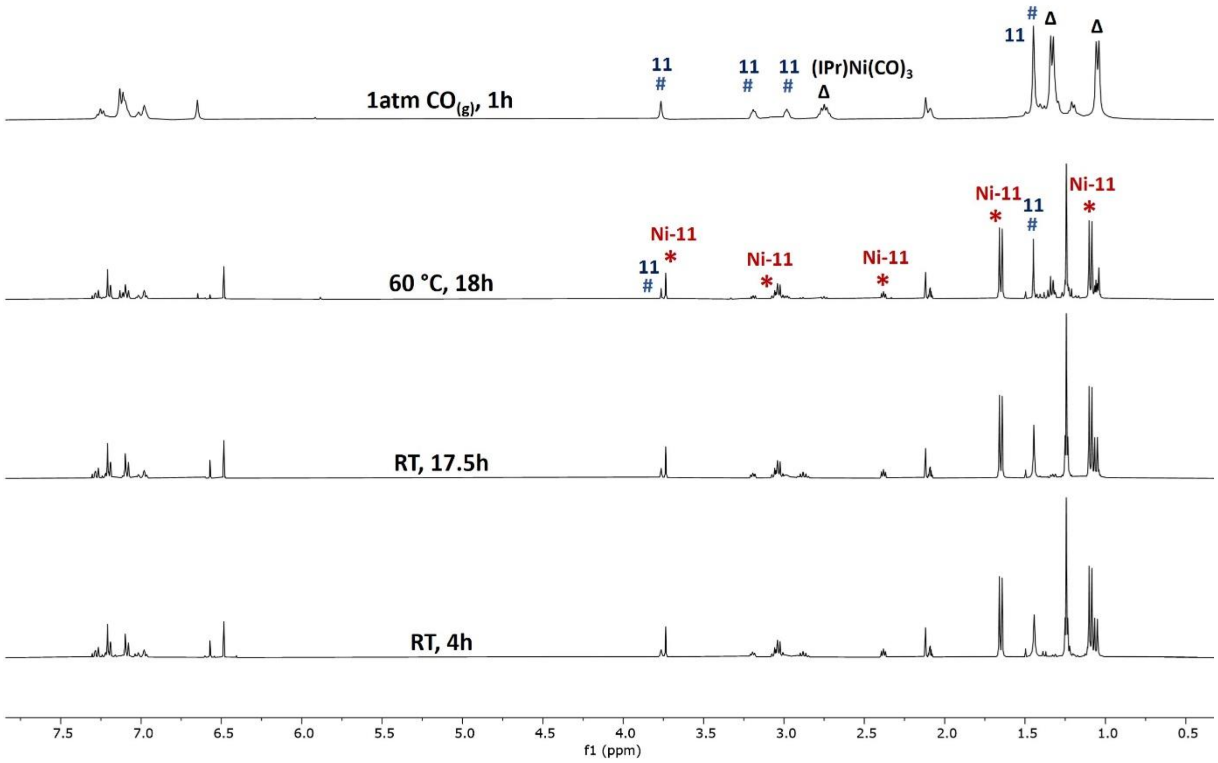

Supplementary Figure 27. Stacked ^{1}H NMR spectra of reaction progress between **11** and (IPr)Ni(η^6-PhMe) and treatment with $CO_{(g)}$.

5.45 Reaction between **Ni-13** and $CO_{(g)}$

In an argon-filled glovebox, lactam **13** (0.01 mmol, 2.6 mg) and (IPr)Ni(η^6-PhMe) (0.01 mmol, 5.4 mg) were added into a vial, followed by addition of 0.4 mL toluene-d_8. The solution was transferred into an oven-dried NMR tube and sealed. The reaction progress was monitored by ^{1}H NMR spectroscopy. The formation of **Ni-13** (63%) and (IPr)Ni(CO)$_3$ (20%) were confirmed before addition of $CO_{(g)}$. The headspace was evacuated, and 1atm of $CO_{(g)}$ was introduced. Formation of lactam **13** (>98%) and (IPr)Ni(CO)$_3$ (>98%) were identified.

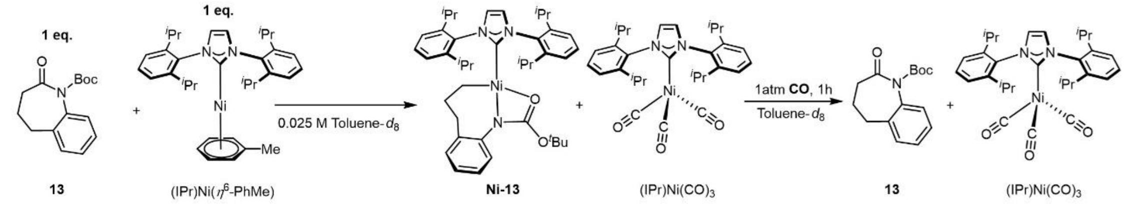

Condition	% remaining		% conv.		% conv.	
RT, 1h	58	39	41	3	-	-
RT, 40h	18	0	63	20	-	-
1atm CO, 1h	-	-	-	-	>98	>98

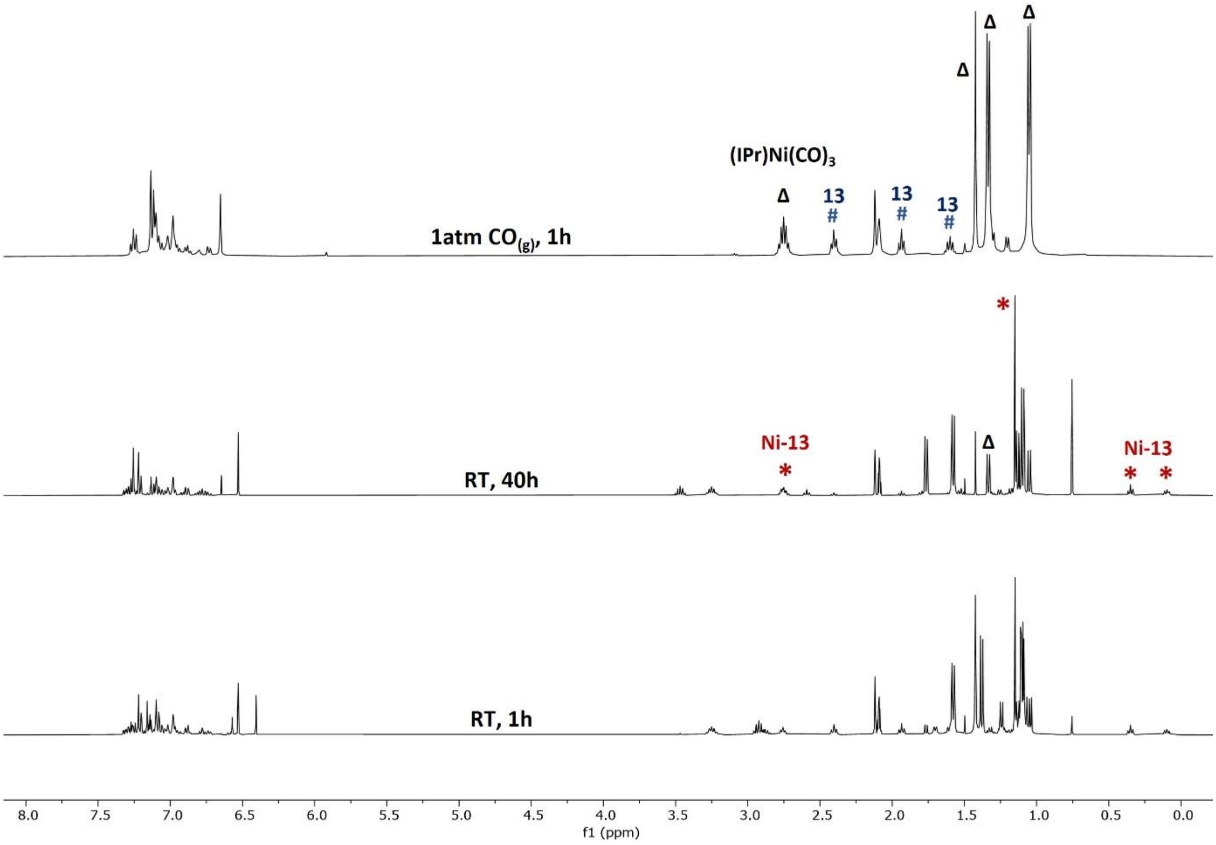

Supplementary Figure 28. Stacked ^{1}H NMR spectra of reaction progress between **13** and (IPr)Ni(η^6-PhMe) and treatment with $CO_{(g)}$.

5.46 Reaction progress monitoring of **Ni-20** and reaction with $CO_{(g)}$

In an argon-filled glovebox, aza-steroid **20** (0.01 mmol, 4.3 mg) and (IPr)Ni(η^6-PhMe) (0.01 mmol, 5.4 mg) were added into a vial, followed by addition of 0.4 mL benzene-d_6. The solution was transferred into an oven-dried NMR tube and sealed. The reaction progress was monitored by ^{1}H NMR spectroscopy. Formation of several nickel intermediates and **Ni-20** was observed. The headspace was evacuated and treatment with 1atm CO resulted in formation of **20** (64%) and (IPr)Ni(CO)$_3$ (68%).

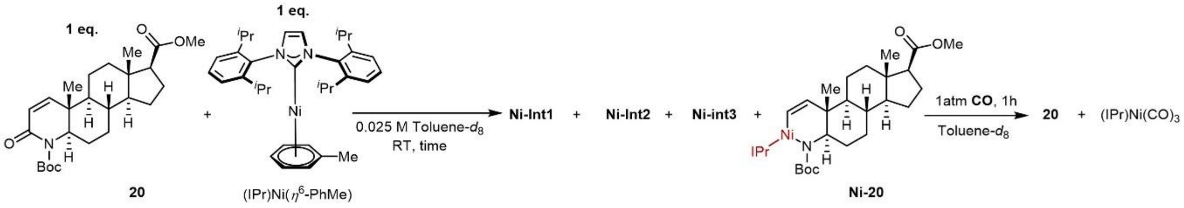

Condition	% remaining		% conv.				% conv.	
RT, 0.5h	57	0	14	26	1	0	-	-
RT, 1.5h	36	0	21	21	3	2	-	-
RT, 2.5h	29	0	15	15	6	9	-	-
RT, 6h	21	0	6	1	4	11	-	-
RT, 23.5h	16	0	0	0	5	21	-	-
RT, 74.5h	13	0	0	0	0	24	-	-
1atm CO, 1h	-	-	-	-	-	-	64	68

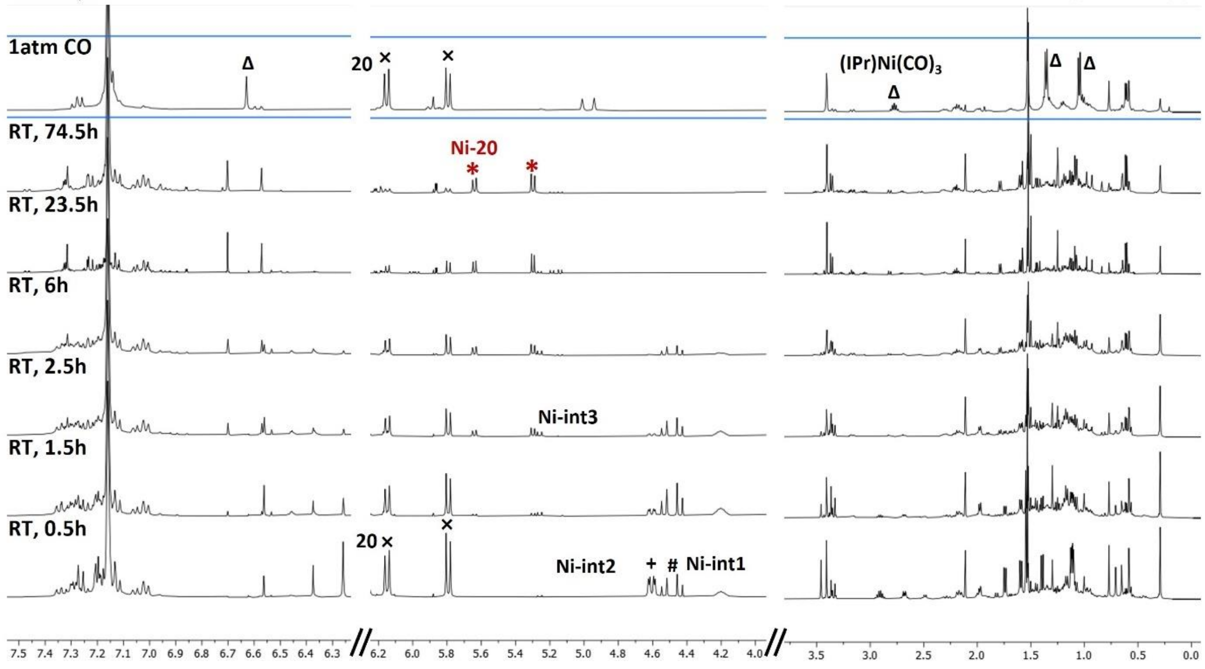

Supplementary Figure 29. Stacked ^{1}H NMR spectra of reaction progress between **20** and (IPr)Ni(η^6-PhMe) and treatment with $CO_{(g)}$.

5.47 Carbon isotope exchange of **20** with $^{13}CO_{(g)}$

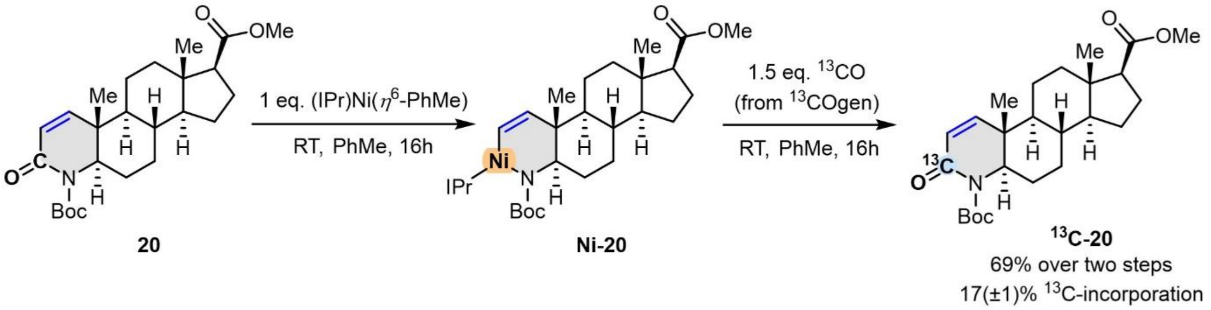

Supplementary Figure 30. Carbon isotope exchange of the lactam carbonyl of azasteroid **20** through the vinyl nickellacycle **Ni-20**. See sections 3.27 and 3.28 for experimental details. The lower reaction temperature (RT) and equivalence of nickel (1 equiv. (IPr)Ni(η^6-PhMe)) required to achieve full consumption of the starting lactam compared to the saturated azasteroid **19** (50 °C with 3 equiv. (IPr)Ni(η^6-PhMe)) indicated that activation of the enamide C–N bond and formation of a vinyl nickellacycle is more facile than its saturated counterpart **19**. The relatively lower ^{13}C-incorporation (17±1%) might be related to the multiple intermediate nickel species (observed by ^{1}H NMR monitoring in section 5.46) that interfered with the formation of **Ni-20** and subsequent reaction with $^{13}CO_{(g)}$.

6. X-ray crystallographic data

6.1 Comparative analysis of X-ray structure parameters of 5- to 8-membered nickellacycles

6.11 Comparison of Ni–N and Ni–O bond distances based on ring size.

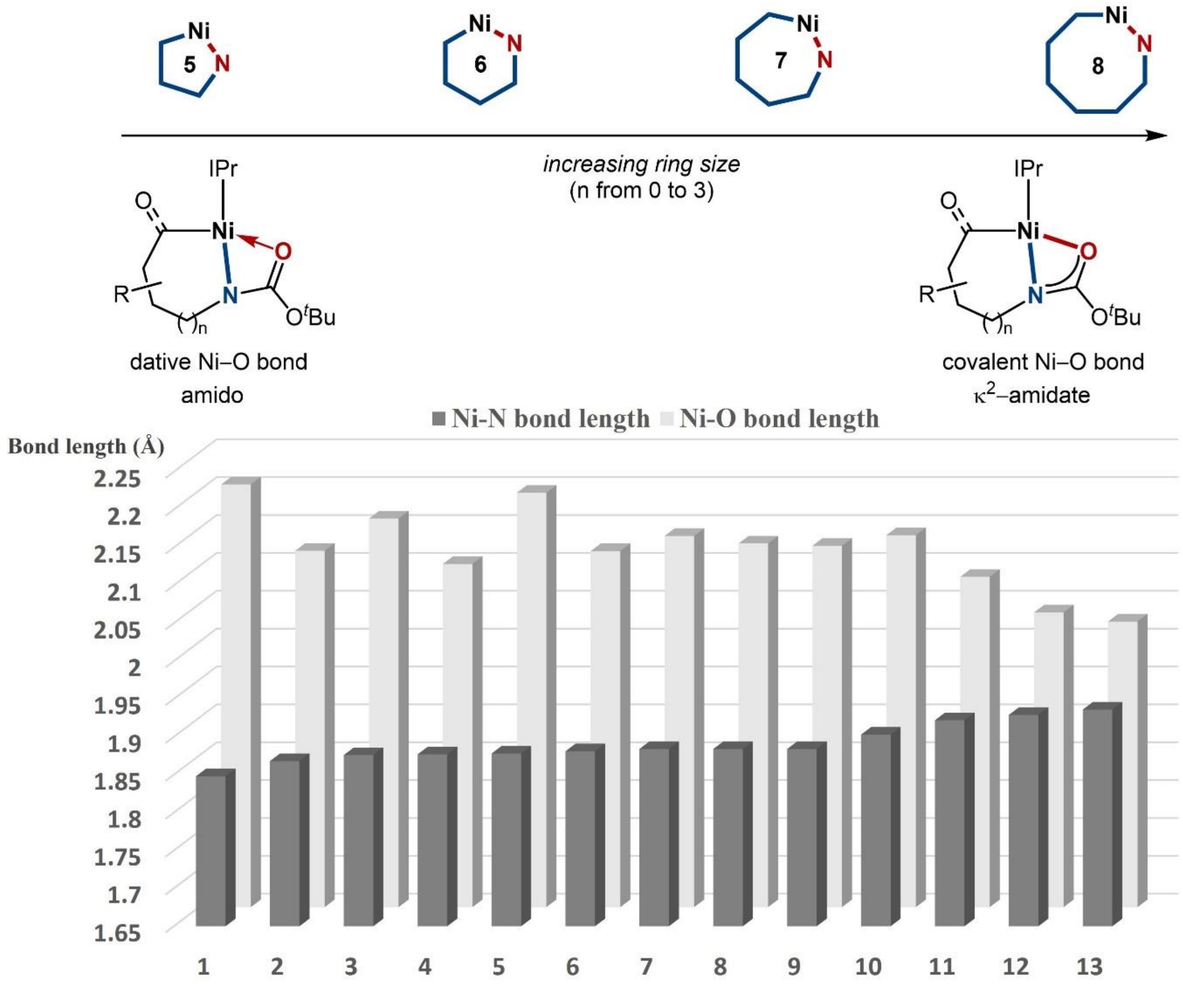

Nickellacycle	Ni-7b	Ni-12	Ni-8	Ni-10	Ni-3a	Ni-2	Ni-9	Ni-1	Ni-11	Ni-5	Ni-13	Ni-15	Ni-14
Ring size	5	6	6	6	6	6	6	6	6	6	7	8	7

Supplementary Figure 31. Comparison of Ni–N and Ni–O bond distances correlated to nickellacycle ring sizes (ranked in Ni–N bond distances). The trend suggests that in smaller nickellacycles (5-membred or 6-membered), the bonding may be better described as covalent Ni–N bonds (amido) and dative Ni–O bonds. In larger nickellacycles (7-membred or 8-membered), the Ni–N and Ni–O bond distances become comparable, and the Ni–O bond has more covalent character. The coordination may be better described as a κ^2-amidate. For reference, in a reported non-cyclic Ni(II) κ^2-amidate complex the difference between the Ni–N and Ni–O bond distances is smaller than 0.01 angstrom.[55]

6.12 Comparison of bond angles based on ring size.

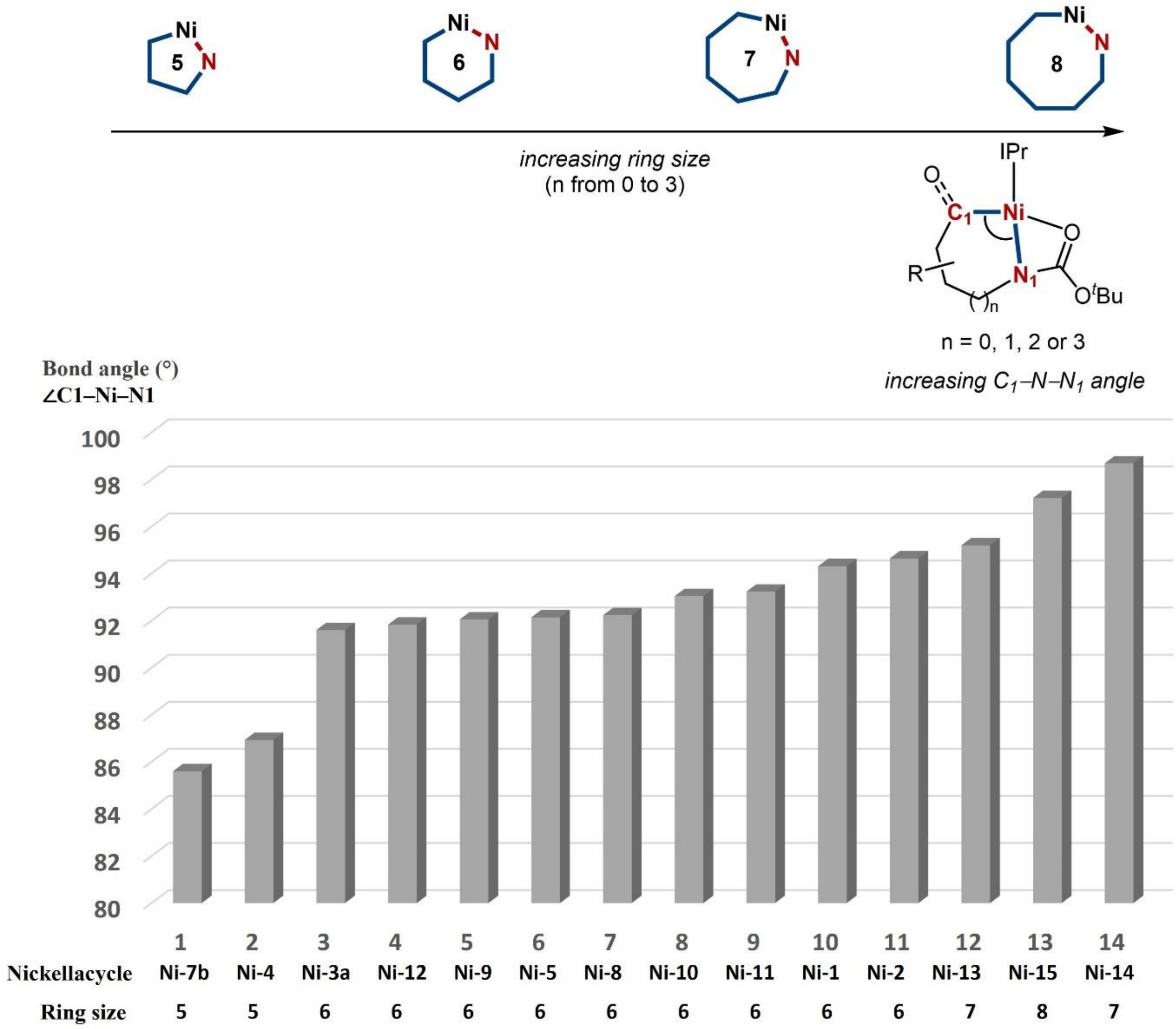

Supplementary Figure 32. Trend of ∠C1–Ni–N1 bond angles correlated to nickellacycle ring sizes.

6.13 Comparison of Ni–C bond distances based on ring size.

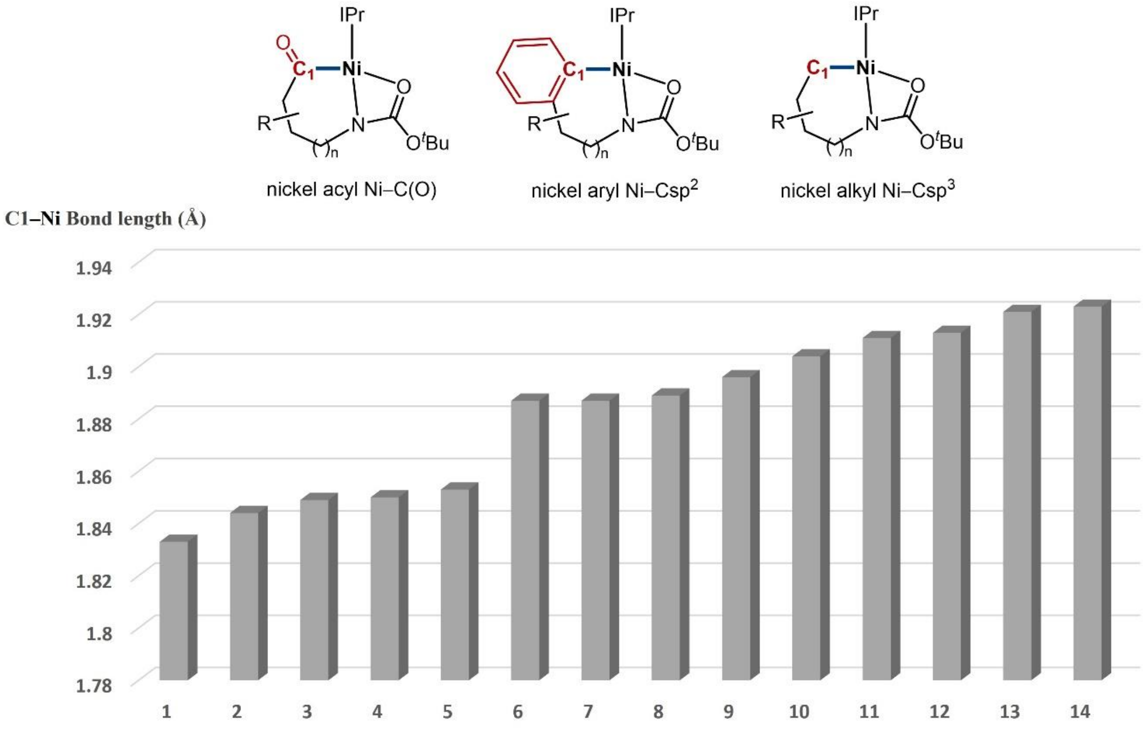

Nickellacycle	Ni-5	Ni-1	Ni-4	Ni-3a	Ni-7b	Ni-12	Ni-14	Ni-10	Ni-15	Ni-11	Ni-9	Ni-8	Ni-2	Ni-13
Ring size	6	6	5	6	5	6	7	6	8	6	6	6	6	7
Ni acyl (Ni–C(O))	√	√	√	√										
Ni aryl (Ni–Csp^2)							√	√	√					
Ni alkyl (Ni–Csp^3)					√	√				√	√	√	√	√

Supplementary Figure 33. Summary of Ni–C1 bond distances.

6.14 Comparison of torsion angles in nickel acyl complexes

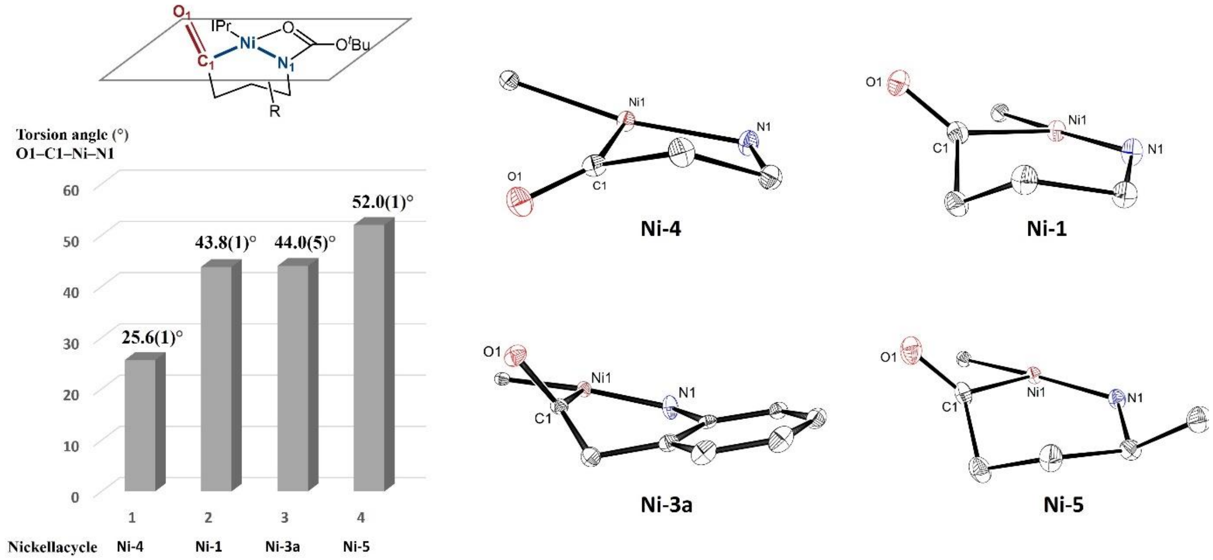

Supplementary Figure 34. Summary of torsion angles (∠O1–C1–Ni1–N1) and truncated ORTEP plots of acyl nickellacycles with H atoms omitted for clarity.

6.2 Crystal structure tables

Supplementary table 1. X-ray structure parameters of **Ni-1**, **Ni-2** and **Ni-3**.

	**Ni-1**	**Ni-2**	**Ni-3a / Ni-3b co-crystal**
CCDC #	2227410	2227411	2227412
Empirical formula	$C_{36}H_{51}N_3NiO_3$	$C_{36}H_{53}N_3NiO_2$	$C_{39.82}H_{51}N_3NiO_{2.82}$
Formula weight	632.50	618.52	675.43
Temperature/K	100.0(1)	100.0(1)	100.0(1)
Crystal system	monoclinic	monoclinic	monoclinic
Space group	$P2_1/c$ (14)	$P2_1/c$ (14)	$P2_1/n$ (14)
a/Å	13.3585(1)	11.7455(1)	11.1743(1)
b/Å	10.2840(1)	15.0020(2)	20.8403(2)
c/Å	25.3440(2)	19.9503(2)	16.1866(2)
α/°	90	90	90
β/°	98.938(1)	100.497(1)	107.085(1)
γ/°	90	90	90
Volume/Å^3	3439.45(5)	3456.53(7)	3603.12(7)
Z	4	4	4
ρ_{calc} g/cm^3	1.221	1.189	1.245
μ/mm^{-1}	1.100	1.060	1.082
F(000)	1360	1336	1446
Crystal size/mm^3	0.248×0.211×0.166	0.201×0.193×0.107	0.244×0.098×0.055
Crystal colour	clear yellow	clear yellow	clear orange
Crystal shape	block	block	block
Radiation	Cu K_α (λ=1.54184)	Cu K_α (λ=1.54184)	Cu K_α (λ=1.54184)
2Θ range/°	6.70 to 159.98	7.42 to 159.79	7.12 to 159.82
Index ranges	$-14 \leq h \leq 16$	$-14 \leq h \leq 14$	$-14 \leq h \leq 14$
	$-13 \leq k \leq 13$	$-18 \leq k \leq 19$	$-26 \leq k \leq 26$
	$-32 \leq l \leq 32$	$-25 \leq l \leq 20$	$-20 \leq l \leq 15$
Reflections collected	93080	47901	50227
Independent reflections	7402	7424	7746
	R_{int} = 0.0422	R_{int} = 0.0438	R_{int} = 0.0518
	R_{sigma} = 0.0169	R_{sigma} = 0.0258	R_{sigma} = 0.0310
Data / Restraints / Param.	7402/0/399	7424/42/409	7746/452/487
Goodness-of-fit on F^2	1.016	1.066	1.038
Final R indexes	R_1 = 0.0325	R_1 = 0.0342	R_1 = 0.0370
[$I \geq 2\sigma(I)$]	wR_2 = 0.0829	wR_2 = 0.0859	wR_2 = 0.0908
Final R indexes	R_1 = 0.0351	R_1 = 0.0388	R_1 = 0.0404
[all data]	wR_2 = 0.0846	wR_2 = 0.0883	wR_2 = 0.0928
Largest peak/hole /eÅ^3	0.40/-0.35	0.25/-0.33	0.26/-0.49
Flack x parameter	-	-	-

Supplementary table 2. X-ray structure parameters of **Ni-4**, **Ni-5** and **Ni-7a/Ni-7b**.

	**Ni-4**	**Ni-5**	**Ni-7a / Ni-7b co-crystal**
CCDC #	2227413	2227414	2227415
Empirical formula	$C_{35}H_{49}N_3NiO_3$	$C_{37}H_{53}N_3NiO_3$	$C_{39.30}H_{50}FN_3NiO_{2.30}$
Formula weight	618.48	646.53	678.86
Temperature/K	100.0(1)	100.0(1)	100.0(1)
Crystal system	monoclinic	monoclinic	monoclinic
Space group	$P2_1/c$ (14)	$P2_1/c$ (14)	$P2_1/n$ (14)
a/Å	10.2712(2)	11.1195(1)	10.6470(1)
b/Å	18.3044(4)	19.7281(2)	22.2959(3)
c/Å	18.2774(4)	16.9048(2)	15.8441(2)
α/°	90	90	90
β/°	97.707(2)	108.932(1)	101.815(1)
γ/°	90	90	90
Volume/Å^3	3405.26(13)	3507.74(7)	3681.46(8)
Z	4	4	4
ρ_{calc} g/cm^3	1.206	1.224	1.225
μ/mm^{-1}	0.606	1.089	1.091
F(000)	1328	1392	1449
Crystal size/mm^3	0.293×0.258×0.220	0.219×0.173×0.101	0.203×0.146×0.114
Crystal colour	dark brown	clear yellow	clear orange
Crystal shape	block	block	block
Radiation	Mo K_α (λ=0.71073)	Cu K_α (λ=1.54184)	Cu K_α (λ=1.54184)
2Θ range/°	4.86 to 69.75	7.12 to 159.49	6.94 to 160.11
Index ranges	$-16 \leq h \leq 16$	$-14 \leq h \leq 13$	$-13 \leq h \leq 13$
	$-29 \leq k \leq 24$	$-25 \leq k \leq 24$	$-28 \leq k \leq 28$
	$-28 \leq l \leq 28$	$-21 \leq l \leq 20$	$-18 \leq l \leq 20$
Reflections collected	68143	57499	51371
Independent reflections	13254	7562	7919
	R_{int} = 0.0372	R_{int} = 0.0463	R_{int} = 0.0432
	R_{sigma} = 0.0322	R_{sigma} = 0.0250	R_{sigma} = 0.0260
Data / Restraints / Param.	13254/0/390	7562/0/409	7919/622/520
Goodness-of-fit on F^2	1.042	1.040	1.064
Final R indexes	R_1 = 0.0376	R_1 = 0.0346	R_1 = 0.0434
[$I \geq 2\sigma(I)$]	wR_2 = 0.0962	wR_2 = 0.0838	wR_2 = 0.1040
Final R indexes	R_1 = 0.0500	R_1 = 0.0383	R_1 = 0.0492
[all data]	wR_2 = 0.1010	wR_2 = 0.0857	wR_2 = 0.1074
Largest peak/hole /eÅ^3	0.64/-0.28	0.43/-0.36	0.33/-0.48
Flack x parameter	-	-	-

Supplementary table 3. X-ray structure parameters of **Ni-8**, **Ni-9** and **Ni-10**.

	**Ni-8**	**Ni-9**	**Ni-10**
CCDC #	2227416	2227417	2227418
Empirical formula	$C_{36}H_{51}N_3NiO_2$	$C_{40}H_{53}N_3NiO_2$	$C_{40}H_{53}N_3NiO_2$
Formula weight	616.50	666.56	666.56
Temperature/K	100.0(1)	100.0(1)	100.0(1)
Crystal system	monoclinic	monoclinic	monoclinic
Space group	$P2_1/c$ (14)	$P2_1/n$ (14)	$P2_1/c$ (14)
a/Å	10.1829(1)	11.0984(2)	16.7813(2)
b/Å	18.3376(2)	20.8967(4)	10.2544(1)
c/Å	18.1778(2)	16.2402(3)	22.1529(3)
α/°	90	90	90
β/°	93.436(1)	106.909(2)	109.767(1)
γ/°	90	90	90
Volume/Å^3	3388.24(6)	3603.60(12)	3587.49(8)
Z	4	4	4
ρ_{calc} g/cm^3	1.209	1.229	1.234
μ/mm^{-1}	1.081	1.056	1.061
F(000)	1328	1432	1432
Crystal size/mm^3	0.240×0.177×0.085	0.326×0.177×0.117	0.224×0.088×0.048
Crystal colour	clear orange	clear colourless	clear yellow
Crystal shape	block	block	plank
Radiation	Cu K_α (λ=1.54184)	Cu K_α (λ=1.54184)	Cu K_α (λ=1.54184)
2Θ range/°	6.85 to 159.90	7.09 to 159.98	5.60 to 159.55
Index ranges	$-12 \leq h \leq 12$	$-10 \leq h \leq 13$	$-21 \leq h \leq 21$
	$-21 \leq k \leq 23$	$-26 \leq k \leq 25$	$-10 \leq k \leq 12$
	$-23 \leq l \leq 21$	$-20 \leq l \leq 20$	$-28 \leq l \leq 26$
Reflections collected	52038	27515	57757
Independent reflections	7279	7569	7693
	R_{int} = 0.0403	R_{int} = 0.0521	R_{int} = 0.0385
	R_{sigma} = 0.0232	R_{sigma} = 0.0453	R_{sigma} = 0.0222
Data / Restraints / Param.	7279/0/390	7569/0/426	7693/0/426
Goodness-of-fit on F^2	1.056	1.043	1.051
Final R indexes	R_1 = 0.0395	R_1 = 0.0467	R_1 = 0.0305
[$I \geq 2\sigma(I)$]	wR_2 = 0.1021	wR_2 = 0.1212	wR_2 = 0.0756
Final R indexes	R_1 = 0.0435	R_1 = 0.0554	R_1 = 0.0345
[all data]	wR_2 = 0.1048	wR_2 = 0.1264	wR_2 = 0.0775
Largest peak/hole /eÅ^3	0.99/-0.40	0.48/-0.55	0.35/-0.31
Flack x parameter	-	-	-

Supplementary table 4. X-ray structure parameters of **Ni-11**, **Ni-12** and **Ni-13**.

	**Ni-11**	**Ni-12**	**Ni-13**
CCDC #	2227419	2227420	2227421
Empirical formula	$C_{35}H_{51}N_3NiO_3$	$C_{39}H_{51}N_3NiO_3$	$C_{89}H_{118}N_6Ni_2O_4$
Formula weight	620.49	668.53	1453.31
Temperature/K	100.0(1)	100.0(1)	100.0(1)
Crystal system	monoclinic	monoclinic	triclinic
Space group	$P2_1/c$ (14)	$P2_1/n$ (14)	P-1 (2)
a/Å	11.4577(1)	10.5993(1)	10.6333(1)
b/Å	15.3356(2)	22.0788(2)	19.2822(3)
c/Å	19.9349(2)	15.9165(1)	21.9063(4)
α/°	90	90	78.638(1)
β/°	99.532(1)	101.781(1)	78.165(1)
γ/°	90	90	89.795(1)
Volume/Å^3	3454.41(7)	3646.31(5)	4306.72(11)
Z	4	4	2
ρ_{calc} g/cm^3	1.193	1.218	1.121
μ/mm^{-1}	1.085	1.067	0.922
F(000)	1336	1432	1564
Crystal size/mm^3	0.249×0.116×0.098	0.273×0.205×0.143	0.157×0.136×0.056
Crystal colour	clear yellow	clear yellow	clear yellow
Crystal shape	block	block	block
Radiation	Cu K_α (λ=1.54184)	Cu K_α (λ=1.54184)	Cu K_α (λ=1.54184)
2Θ range/°	7.31 to 159.81	6.94 to 160.41	4.68 to 160.42
Index ranges	$-14 \le h \le 14$	$-13 \le h \le 13$	$-13 \le h \le 12$
	$-19 \le k \le 16$	$-28 \le k \le 27$	$-24 \le k \le 24$
	$-22 \le l \le 25$	$-20 \le l \le 19$	$-27 \le l \le 27$
Reflections collected	55476	75225	19853
Independent reflections	7415	7890	19853
	R_{int} = 0.0443	R_{int} = 0.0461	R_{int} = 0.0520
	R_{sigma} = 0.0247	R_{sigma} = 0.0212	R_{sigma} = 0.0284
Data / Restraints / Param.	7415/0/390	7890/601/559	19853/339/999
Goodness-of-fit on F^2	1.083	1.044	1.033
Final R indexes	R_1 = 0.0315	R_1 = 0.0342	R_1 = 0.0850
[I≥2σ(I)]	wR_2 = 0.0808	wR_2 = 0.0890	wR_2 = 0.2333
Final R indexes	R_1 = 0.0355	R_1 = 0.0373	R_1 = 0.0961
[all data]	wR_2 = 0.0828	wR_2 = 0.0916	wR_2 = 0.2437
Largest peak/hole /eÅ^3	0.24/-0.37	0.26/-0.43	1.16/-0.70
Flack x parameter	-	-	-

Supplementary table 5. X-ray structure parameters of **Ni-14**, **Ni-15** and **Ni-2-py**.

	**Ni-14**	**Ni-15**	**Ni-2-py**
CCDC #	2227422	2227423	2227424
Empirical formula	$C_{60.25}H_{75}N_5NiO_4$	$C_{52}H_{69}N_3NiO_2$	$C_{43}H_{56}N_4Ni$
Formula weight	991.96	826.81	687.62
Temperature/K	100.0(1)	100.0(1)	100.0(1)
Crystal system	monoclinic	monoclinic	triclinic
Space group	$P2_1/n$ (14)	*C*2/*c* (15)	*P*-1 (2)
a/Å	23.0762(2)	26.2859(4)	10.0834(1)
b/Å	18.4087(2)	16.8933(2)	10.4578(1)
c/Å	26.6011(3)	22.1710(2)	19.9784(2)
α/°	90	90	90.705(1)
β/°	101.367(1)	92.671(1)	98.764(1)
γ/°	90	90	114.760(1)
Volume/$Å^3$	11078.6(2)	9834.5(2)	1883.90(4)
Z	8	8	2
ρ_{calc} g/cm^3	1.189	1.117	1.212
μ/mm^{-1}	0.889	0.863	0.993
F(000)	4252	3568	740
Crystal size/mm^3	0.312×0.237×0.090	0.282×0.202×0.180	0.232×0.179×0.074
Crystal colour	clear colourless	clear yellow	clear yellow
Crystal shape	block	block	block
Radiation	Cu K_α (λ=1.54184)	Cu K_α (λ=1.54184)	Cu K_α (λ=1.54184)
2Θ range/°	4.64 to 160.28	6.22 to 160.65	4.49 to 159.48
Index ranges	$-25 \leq h \leq 29$	$-33 \leq h \leq 33$	$-10 \leq h \leq 12$
	$-23 \leq k \leq 23$	$-21 \leq k \leq 21$	$-13 \leq k \leq 13$
	$-33 \leq l \leq 32$	$-28 \leq l \leq 28$	$-25 \leq l \leq 25$
Reflections collected	156805	220580	39314
Independent reflections	23876	10698	7965
	R_{int} = 0.0532	R_{int} = 0.0845	R_{int} = 0.0344
	R_{sigma} = 0.0323	R_{sigma} = 0.0204	R_{sigma} = 0.0239
Data / Restraints / Param.	23876/1156/1513	10698/372/630	7965/249/495
Goodness-of-fit on F^2	1.086	1.043	1.027
Final *R* indexes	R_1 = 0.0546	R_1 = 0.0725	R_1 = 0.0340
[$I \geq 2\sigma(I)$]	wR_2 = 0.1619	wR_2 = 0.2136	wR_2 = 0.0800
Final *R* indexes	R_1 = 0.0686	R_1 = 0.0769	R_1 = 0.0365
[all data]	wR_2 = 0.1728	wR_2 = 0.2177	wR_2 = 0.0813
Largest peak/hole /e$Å^3$	0.70/-0.51	0.87/-1.16	0.29/-0.32
Flack x parameter	-	-	-

Supplementary table 6. X-ray structure parameters of **Ni-2-Ts**, **(IPrNi)$_2$** and **19a**.

	**Ni-2-Ts**	**(IPrNi)$_2$**	**19a**
CCDC #	2227425	2227426	2227427
Empirical formula	$C_{39}H_{51}N_3NiO$	$C_{54}H_{72}N_4Ni_2$	$C_{24}H_{39}NO_4$
Formula weight	636.53	894.57	405.56
Temperature/K	100.0(1)	100.0(1)	100.0(1)
Crystal system	triclinic	monoclinic	triclinic
Space group	P-1 (2)	$P2_1/n$ (14)	P1 (1)
a/Å	9.5998(2)	12.6637(1)	6.1029(1)
b/Å	10.8720(1)	14.4699(1)	6.2303(1)
c/Å	17.4768(2)	13.1348(1)	15.3307(2)
α/°	105.188(1)	90	81.808(1)
β/°	92.671(1)	90.977(1)	82.309(2)
γ/°	92.565(1)	90	77.118(2)
Volume/Å^3	1755.34(5)	2406.50(3)	559.293(16)
Z	2	2	1
ρ_{calc} g/cm^3	1.204	1.235	1.204
μ/mm^{-1}	1.039	1.256	0.639
F(000)	684	960	222
Crystal size/mm^3	0.305×0.163×0.097	0.206×0.127×0.043	0.263×0.093×0.045
Crystal colour	clear yellow	dark brown	clear colourless
Crystal shape	block	plate	plank
Radiation	Cu K_α (λ=1.54184)	Cu K_α (λ=1.54184)	Cu K_α (λ=1.54184)
2Θ range/°	5.25 to 159.67	9.09 to 159.93	11.73 to 158.50
Index ranges	$-11 \leq h \leq 12$	$-13 \leq h \leq 15$	$-7 \leq h \leq 7$
	$-13 \leq k \leq 13$	$-18 \leq k \leq 18$	$-7 \leq k \leq 7$
	$-21 \leq l \leq 22$	$-16 \leq l \leq 16$	$-19 \leq l \leq 19$
Reflections collected	35108	64507	22762
Independent reflections	7423	5202	4496
	R_{int} = 0.0761	R_{int} = 0.0472	R_{int} = 0.0444
	R_{sigma} = 0.0493	R_{sigma} = 0.0201	R_{sigma} = 0.0312
Data / Restraints / Param.	7423/0/407	5202/0/279	4496/3/268
Goodness-of-fit on F^2	1.088	1.044	1.086
Final R indexes	R_1 = 0.0432	R_1 = 0.0317	R_1 = 0.0360
[$I \geq 2\sigma(I)$]	wR_2 = 0.1128	wR_2 = 0.0827	wR_2 = 0.0951
Final R indexes	R_1 = 0.0504	R_1 = 0.0350	R_1 = 0.0368
[all data]	wR_2 = 0.1183	wR_2 = 0.0847	wR_2 = 0.0958
Largest peak/hole /eÅ^3	0.49/-0.76	0.33/-0.28	0.23/-0.23
Flack x parameter	-	-	0.06(9)

6.3 ORTEP plots of X-ray structures and solid-state parameters

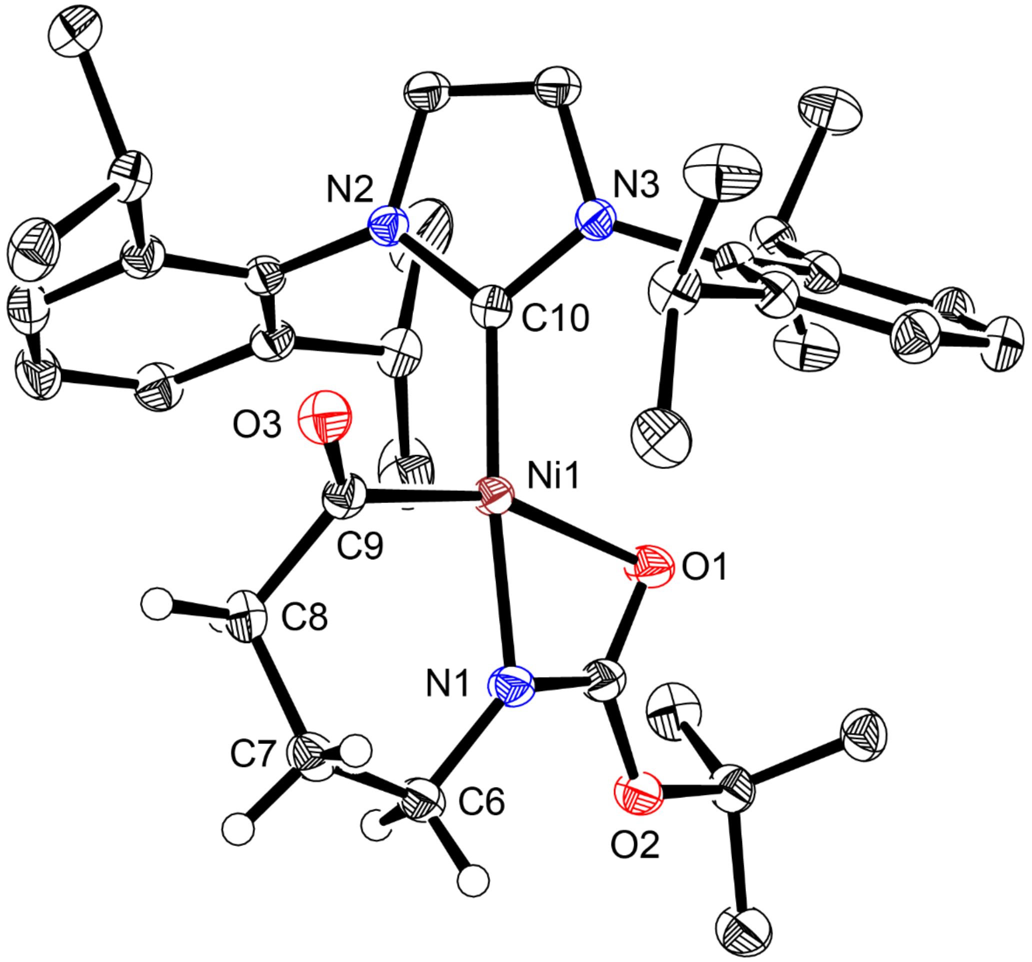

Supplementary Figure 35. ORTEP plot of **Ni-1** in 50% thermal ellipsoids, with H atoms omitted for clarity except for those in the metallacycle. Torsion angle ∠O3–C9–Ni1–N1: 43.8(1)°. Bond angles ∠C9–Ni1–N1: 94.31(5)°, ∠C9–Ni1–C10: 92.54(6)°. Bond distances (Å) Ni1–C9: 1.844(1), Ni1–N1: 1.884(1), Ni1–O1: 2.130(1), Ni1–C10: 1.871(1), C9–O3: 1.211(2).

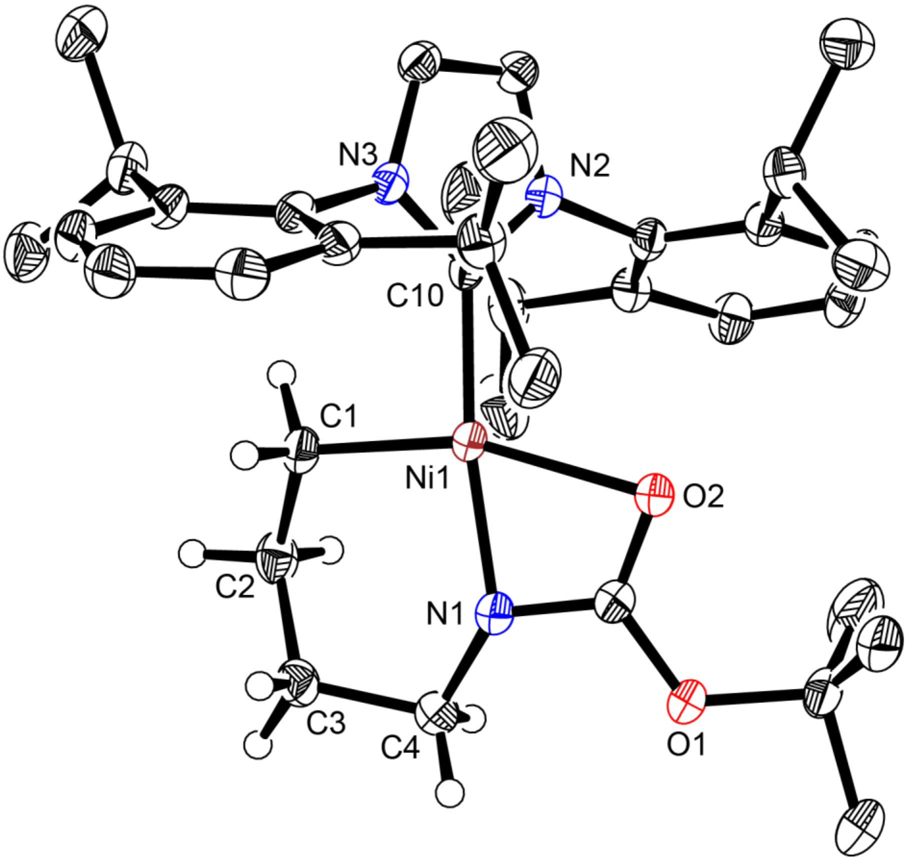

Supplementary Figure 36. ORTEP plot of **Ni-2** in 50% thermal ellipsoids, with H atoms omitted for clarity except for those in the metallacycle. C1 to C4 are disordered over two alternate positions (not shown). Bond angles ∠C1–Ni1–N1: 94.64(6)°, ∠C1–Ni1–C10: 92.58(6)°. Bond distances (Å) Ni1–C1: 1.921(1), Ni1–N1: 1.881(1), Ni1–O2: 2.120(1), Ni1–C10: 1.868(1).

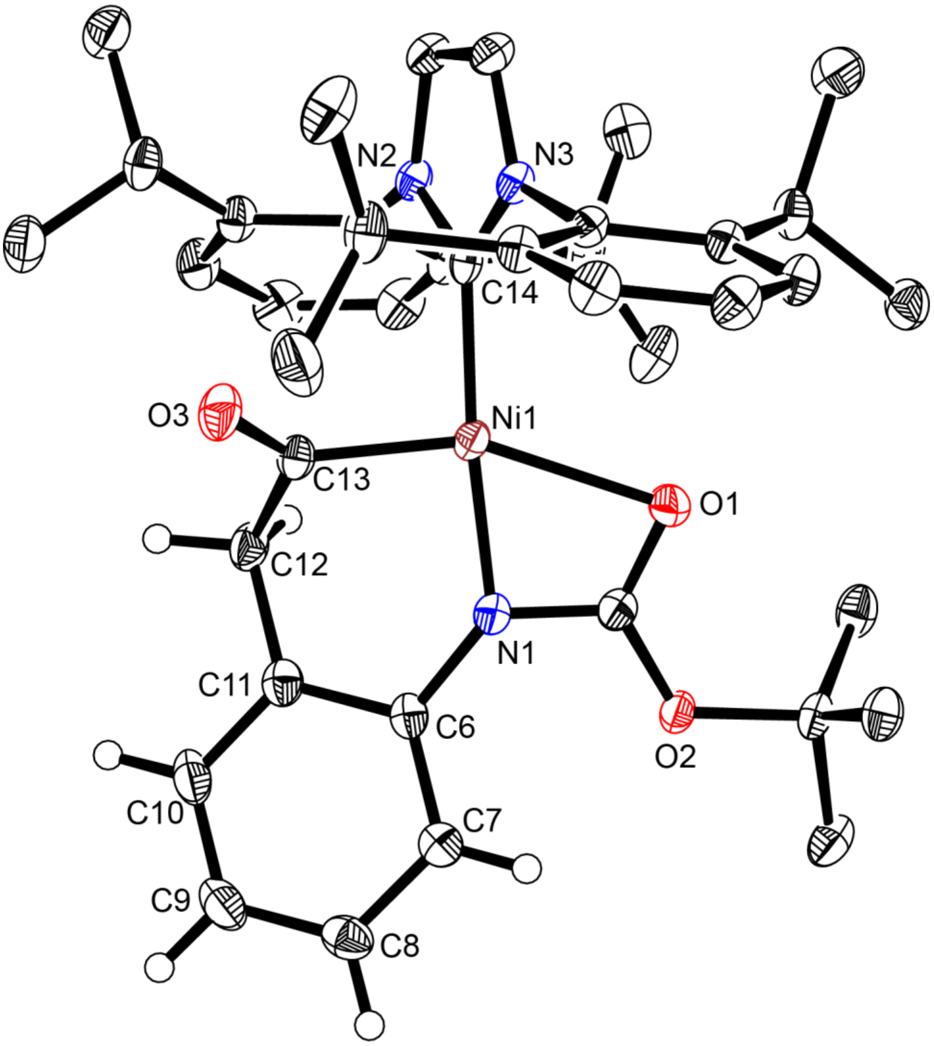

Supplementary Figure 37. ORTEP plot of **Ni-3a** in 50% thermal ellipsoids, with H atoms omitted for clarity except for those in the metallacycle. Crystallization of **Ni-3a** in an *n*-hexane solution at room temperature over the course of 48 hours led to the partial formation of **Ni-3b** (see Fig. S36) upon CO deinsertion. In the reported structure, **Ni-3a** and **Ni-3b** co-crystallized on the same position in about 4:1 occupancy ratio. Structural data of **Ni-3a** was obtained as part of a disorder model and might be biased by applied restraints. Torsion angle ∠O3–C13–Ni1–N1: 43.98(1)°. Bond angles ∠C13–Ni1–N1: 94.31(5)°, ∠C13–Ni1–C14: 92.54(6)°. Bond distances (Å) Ni1–C13: 1.850(7), Ni1–N1: 1.878(1), Ni1–O1: 2.197(1), Ni1–C14: 1.880(2), C13–O3: 1.198(8).

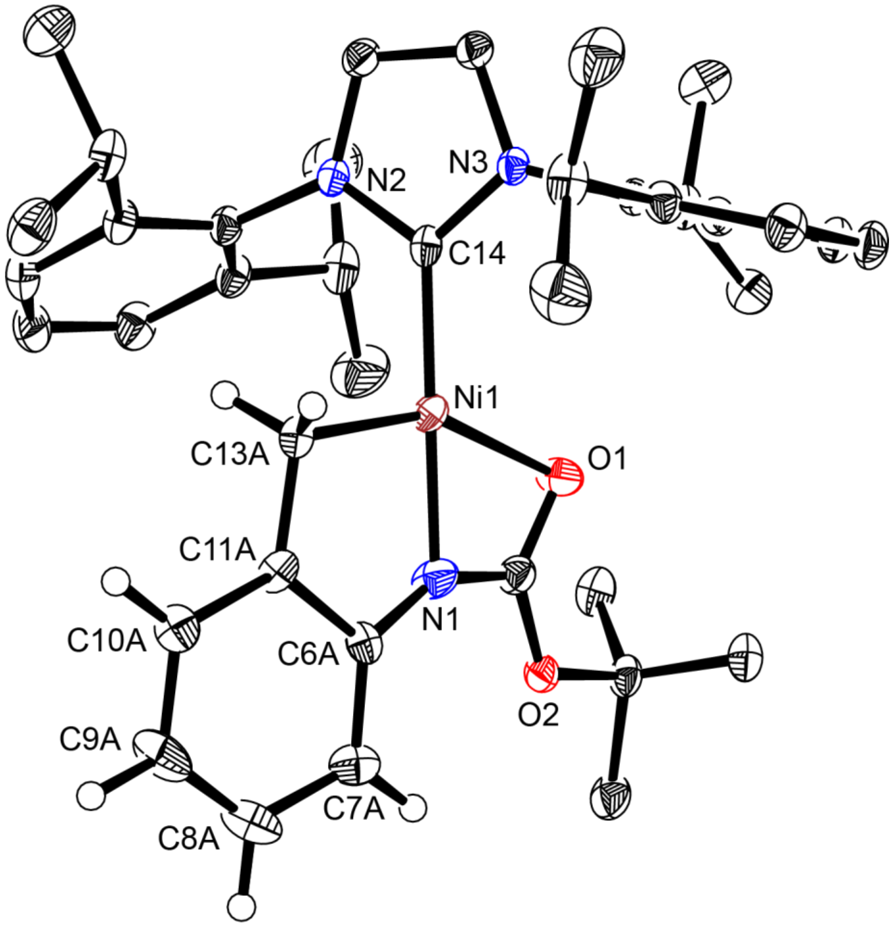

Supplementary Figure 38. ORTEP plot of **Ni-3b** in 50% thermal ellipsoids, with H atoms omitted for clarity except for those in the metallacycle. Structural data of **Ni-3b** was obtained as part of a disorder model (see Fig. S35) and might be biased by applied restraints. Bond angles ∠C13A–Ni1–N1: 87.7(8)°, ∠C13A–Ni1–C14: 96.9(8)°. Bond length (Å): Ni1–C13A: 1.89(3).

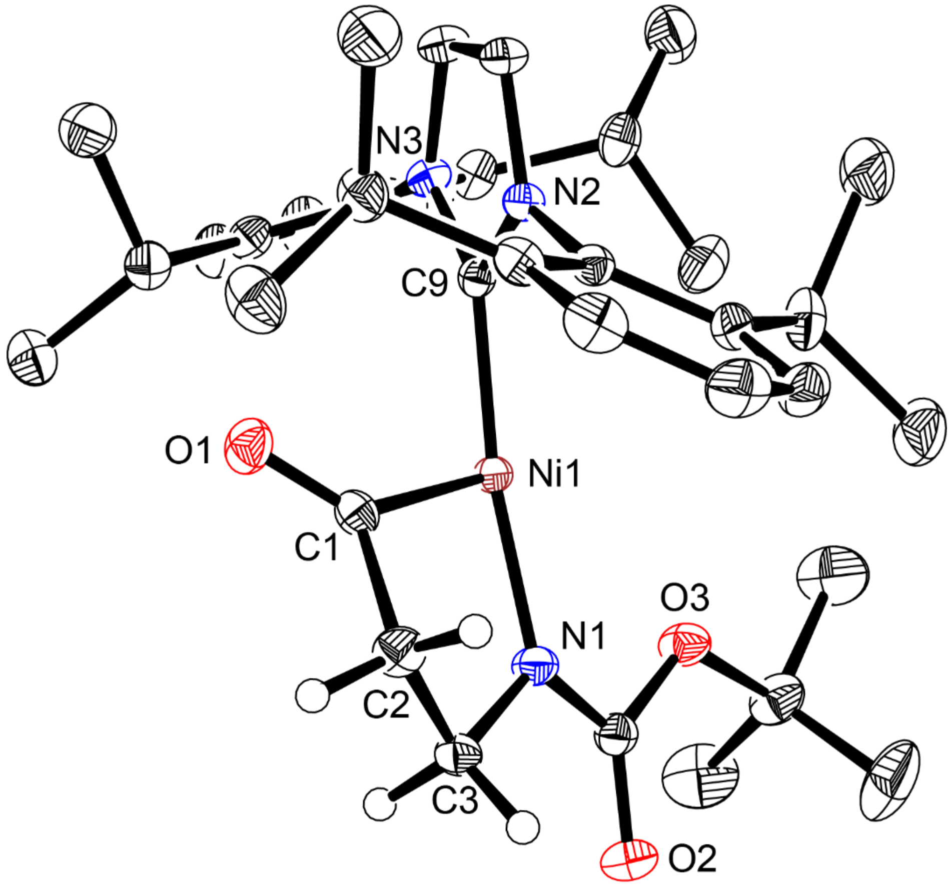

Supplementary Figure 39. ORTEP plot of **Ni-4** in 50% thermal ellipsoids, with H atoms omitted for clarity except for those in the metallacycle. Torsion angle ∠O1–C1–Ni1–N1: 25.64(1)°. Bond angles ∠C1–Ni1–N1: 86.93(5)°, ∠C1–Ni1–C9: 95.20(5)°. Bond distances (Å) Ni1–C1: 1.849(1), Ni1–N1: 1.875(1), Ni1–C9: 1.900(1), C1–O1: 1.201(1).

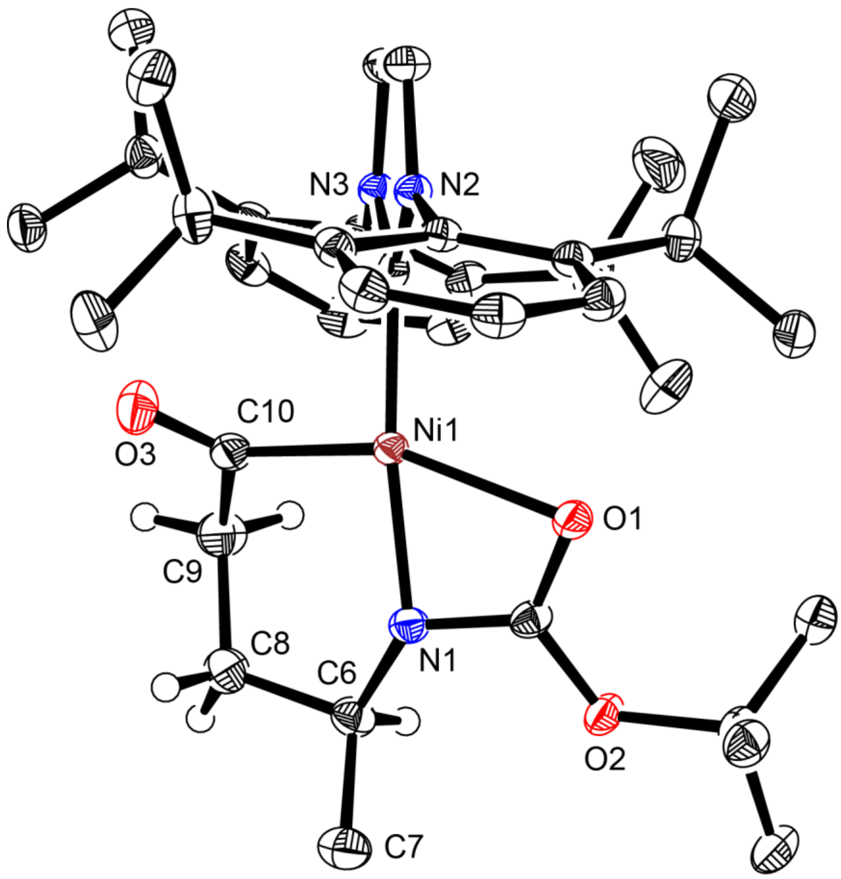

Supplementary Figure 40. ORTEP plot of **Ni-5** in 50% thermal ellipsoids, with H atoms omitted for clarity except for those in the metallacycle. $C_{(carbene)}$ = C11. Torsion angle ∠O3–C10–Ni1–N1: 52.0(1)°. Bond angles ∠C10–Ni1–N1: 92.14(6)°, ∠C10–Ni1–C11: 93.53(6)°. Bond distances (Å) Ni1–C10: 1.833(2), Ni1–N1: 1.903(1), Ni1–O1: 2.141(1), Ni1–C10: 1.833(1), C10–O3: 1.208(2).

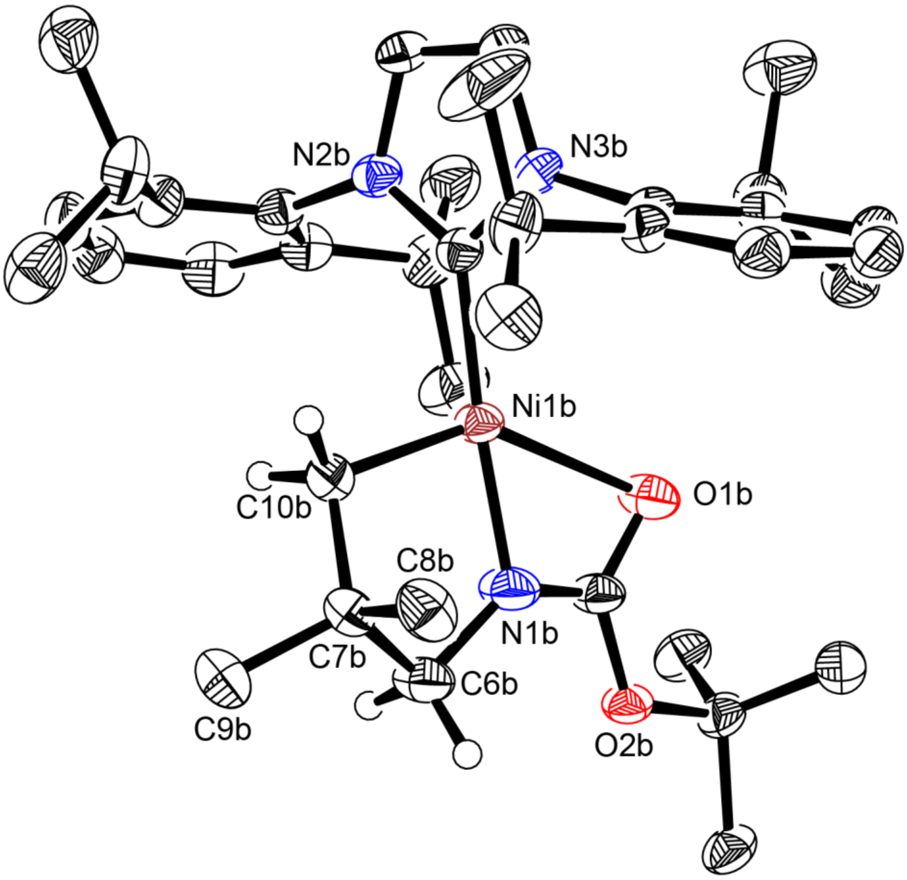

Supplementary Figure 41. ORTEP plot of preliminary structure for **Ni-6** in 50% thermal ellipsoids, with H atoms omitted for clarity except for those in the metallacycle. The crystals were systematically twinned. The asymmetric unit contains four independent nickel molecules, with slightly different disorders, which could not be fully resolved (only one moiety is shown here). Overall data quality is considered too poor for a full publication, and the structure is not discussed in detail herein, nor uploaded to the CCDC. Nevertheless, at least the connectivity of nickel complex **Ni-6** could be established.

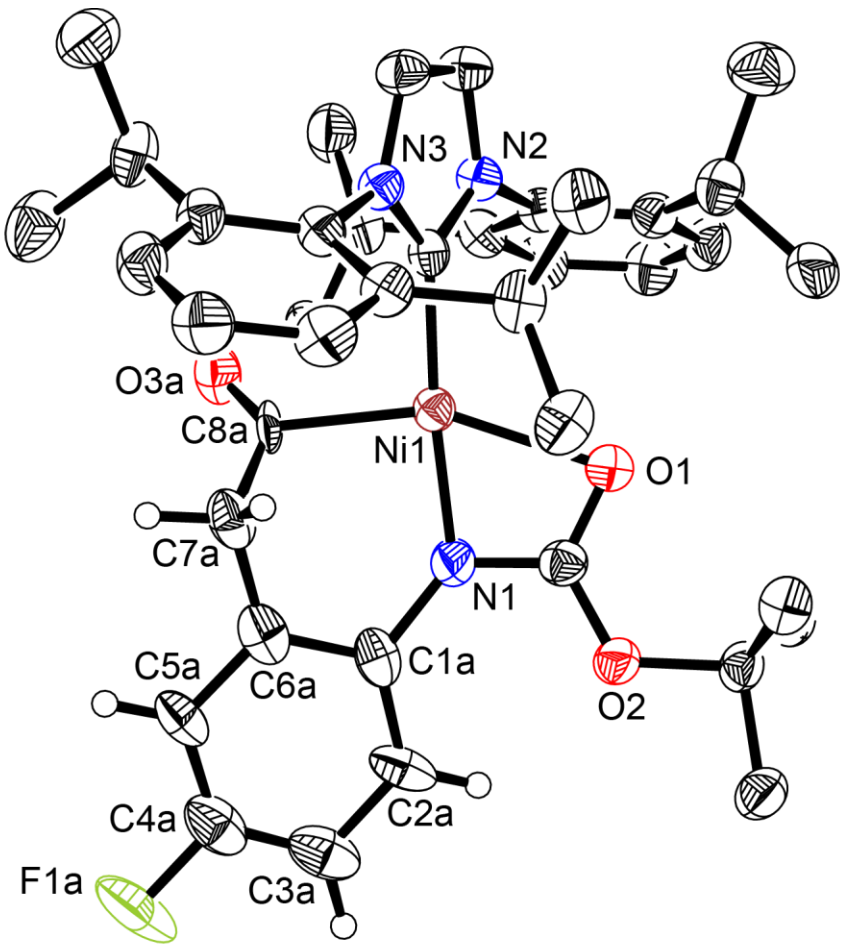

Supplementary Figure 42. ORTEP plot of **Ni-7a** in 50% thermal ellipsoids, with H atoms omitted for clarity except for those in the metallacycle. Crystallization of **Ni-7a** in an *n*-hexane solution at room temperature over the course of 48 hours led to the partial formation of **Ni-7b** (see Fig. S41) upon CO deinsertion. In the reported structure, **Ni-7a** and **Ni-7b** co-crystallized on the same position in about 3:7 occupancy ratio. Structural data of **Ni-7a** was obtained as part of a disorder model, and might be biased by applied restraints. $C_{(carbene)}$ = C13. Torsion angle ∠O3A–C8A–Ni1–N1: 52.4(1)°. Bond angles ∠C8A–Ni1–N1: 91.0(7)°, ∠C8A–Ni1–C13: 94.1(7)°. Bond distances (Å) Ni1–C13: 1.876(2), Ni1–N1: 1.848(2), Ni1–O1: 2.208(1), Ni1–C8A: 1.98 (2), C8A–O3A: 1.19(3).

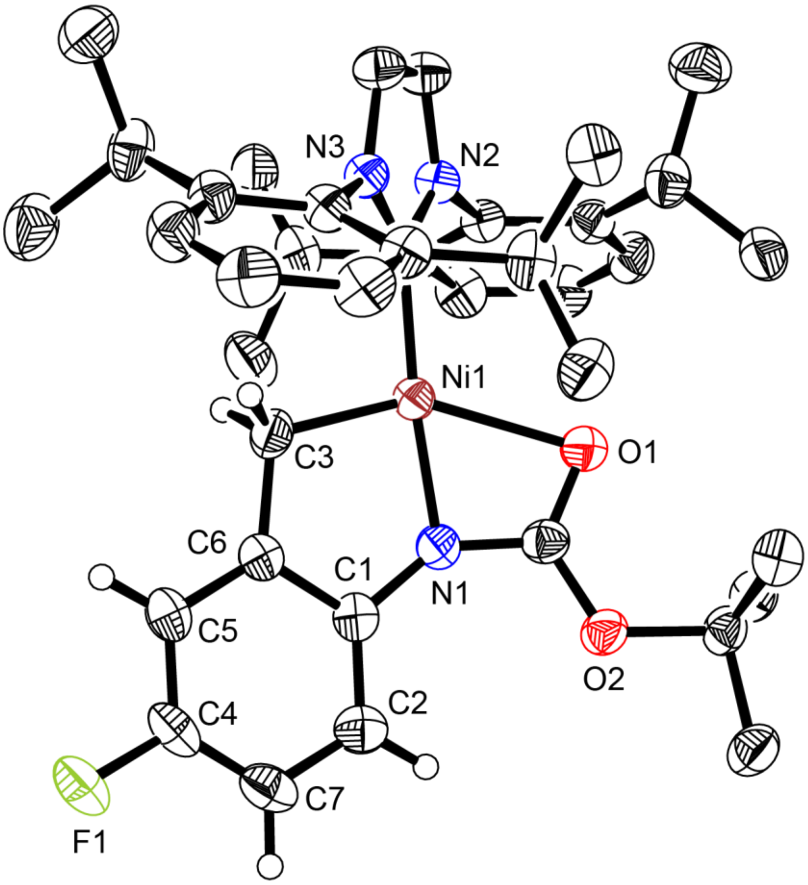

Supplementary Figure 43. ORTEP plot of **Ni-7b** in 50% thermal ellipsoids, with H atoms omitted for clarity except for those in the metallacycle. Structural data of **Ni-7b** was obtained as part of a disorder model (see Fig. S40) and might be biased by applied restraints. $C_{(carbene)}$ = C13. Bond angles ∠C3–Ni1–N1: 85.6(3)°, ∠C3–Ni1–C13: 99.4(3)°. Bond length (Å) Ni1–C3: 1.853(9).

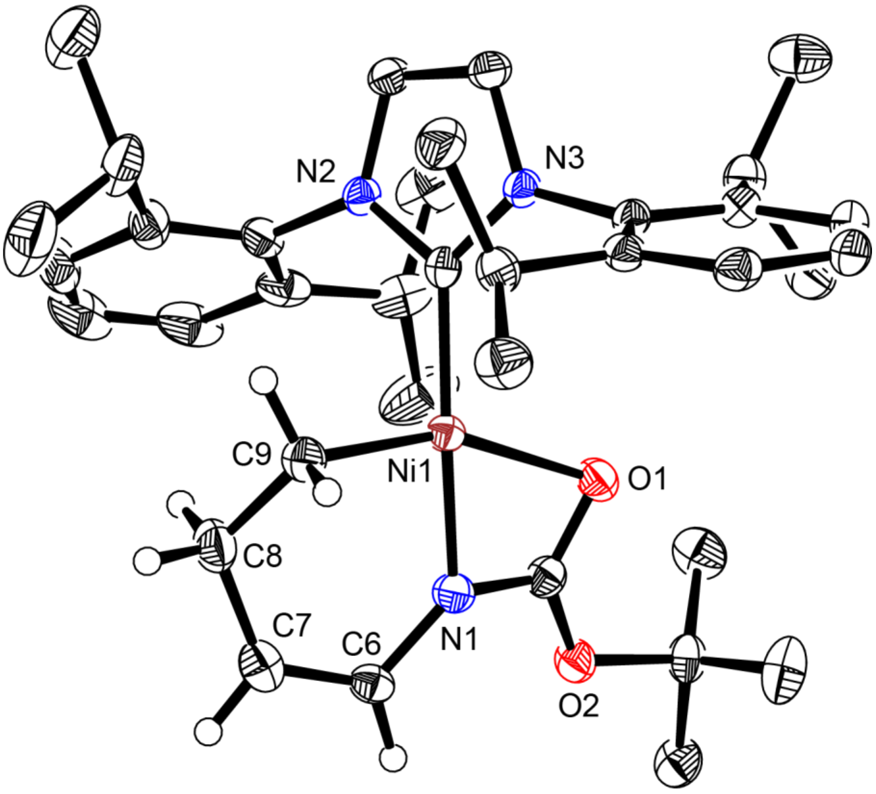

Supplementary Figure 44. ORTEP plot of **Ni-8** in 50% thermal ellipsoids, with H atoms omitted for clarity except for those in the metallacycle. $C_{(carbene)}$ = C10. Bond angles ∠C9–Ni1–N1: 92.23(6)°, ∠C9–Ni1–C10: 94.80(7)°. Bond distances (Å) Ni1–C9: 1.913(2), Ni1–N1: 1.876(1), Ni1–O1: 2.163(1), Ni1–C10: 1.867(2).

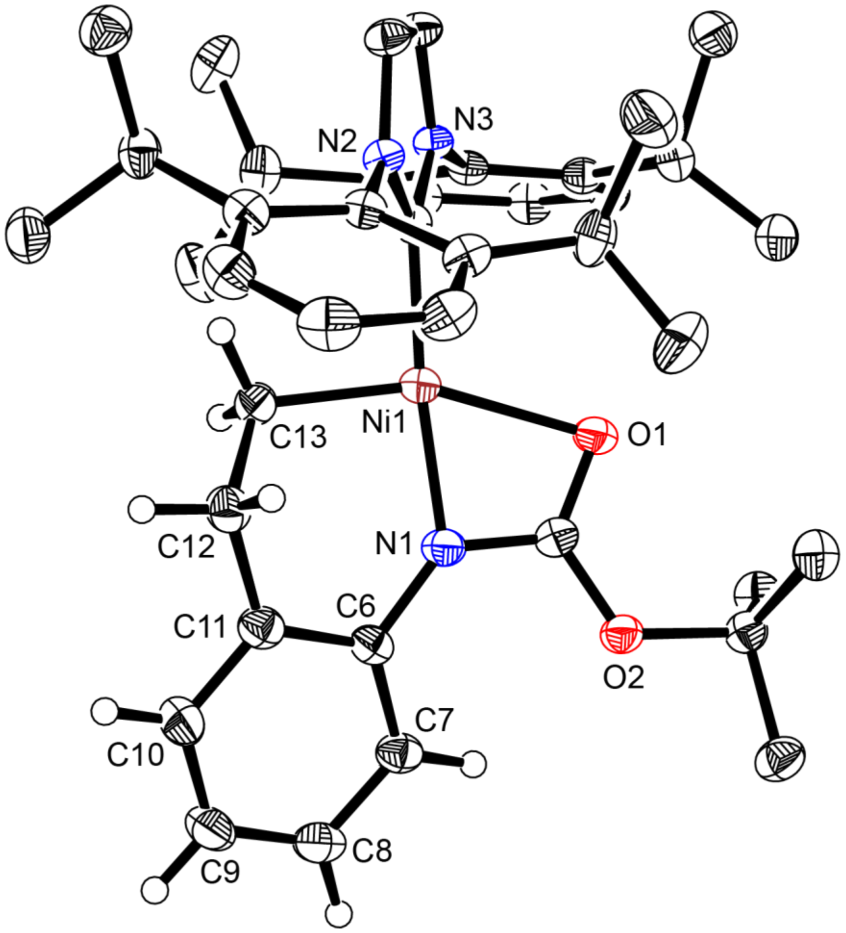

Supplementary Figure 45. ORTEP plot of **Ni-9** in 50% thermal ellipsoids, with H atoms omitted for clarity except for those in the metallacycle. $C_{(carbene)}$ = C14. Bond angles ∠C13–Ni1–N1: 92.05(8)°, ∠C13–Ni1–C14: 93.11(9)°. Bond distances (Å) Ni1–C13: 1.911(2), Ni1–N1: 1.884(2), Ni1–O1: 2.140(2), Ni1–C14: 1.864(2).

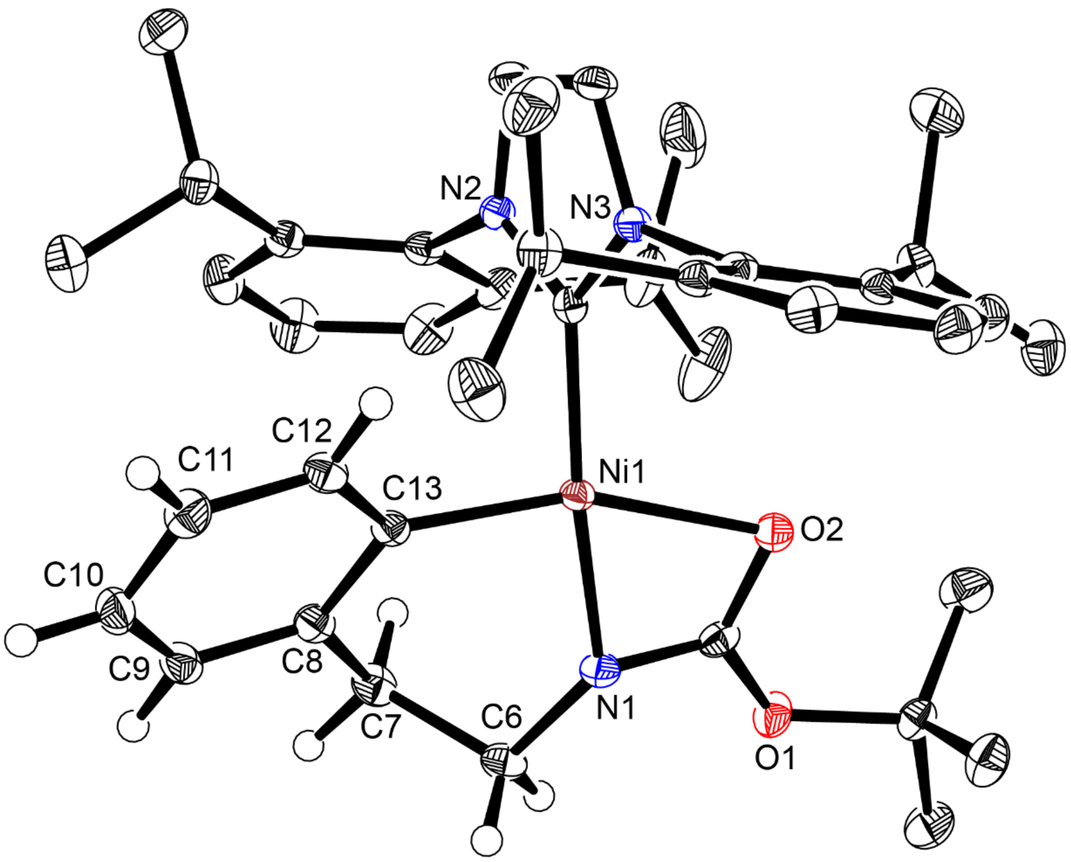

Supplementary Figure 46. ORTEP plot of **Ni-10** in 50% thermal ellipsoids, with H atoms omitted for clarity except for those in the metallacycle. $C_{(carbene)}$ = C14. Bond angles ∠C13–Ni1–N1: 93.04(5)°, ∠C13–Ni1–C14: 94.11(5)°. Bond distances (Å) Ni1–C13: 1.889(2), Ni1–N1: 1.877(1), Ni1–O2: 2.103(1), Ni1–C14: 1.883(1).

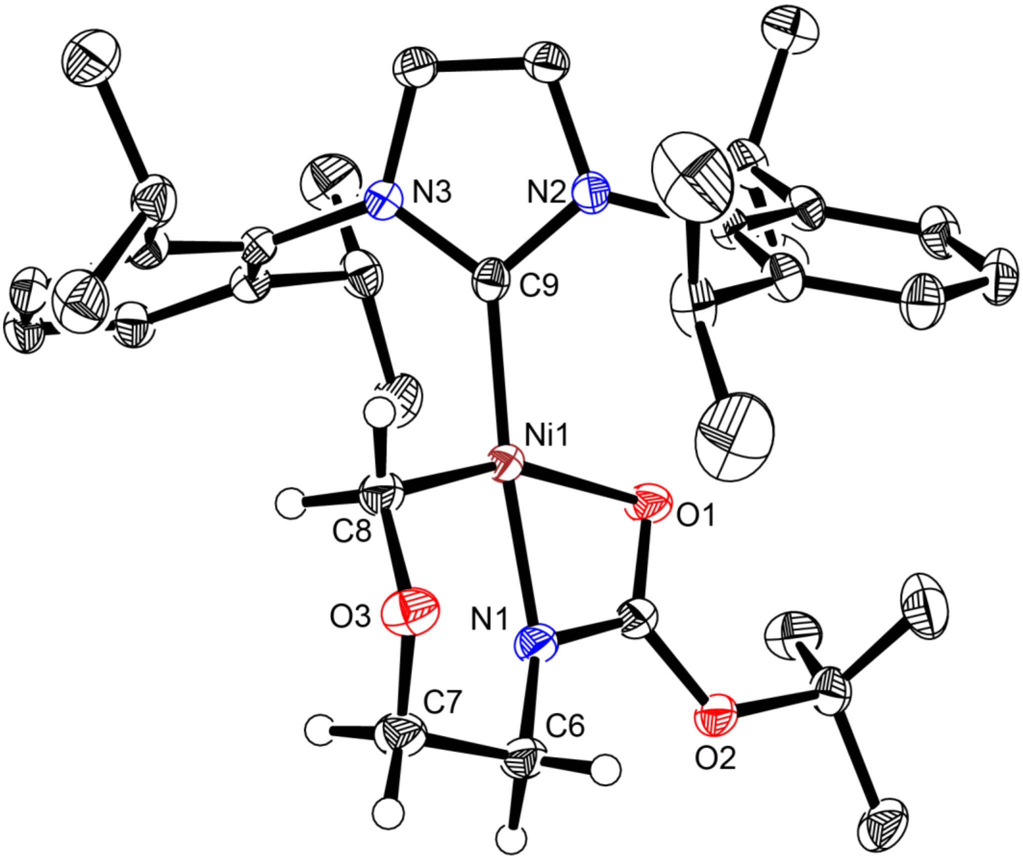

Supplementary Figure 47. ORTEP plot of **Ni-11** in 50% thermal ellipsoids, with H atoms omitted for clarity except for those in the metallacycle. Bond angles ∠C8–Ni1–N1: 93.23(5)°, ∠C8–Ni1–C9: 93.30(5)°. Bond distances (Å) Ni1–C8: 1.904(1), Ni1–N1: 1.884(1), Ni1–O1: 2.127(1), Ni1–C9: 1.866(1).

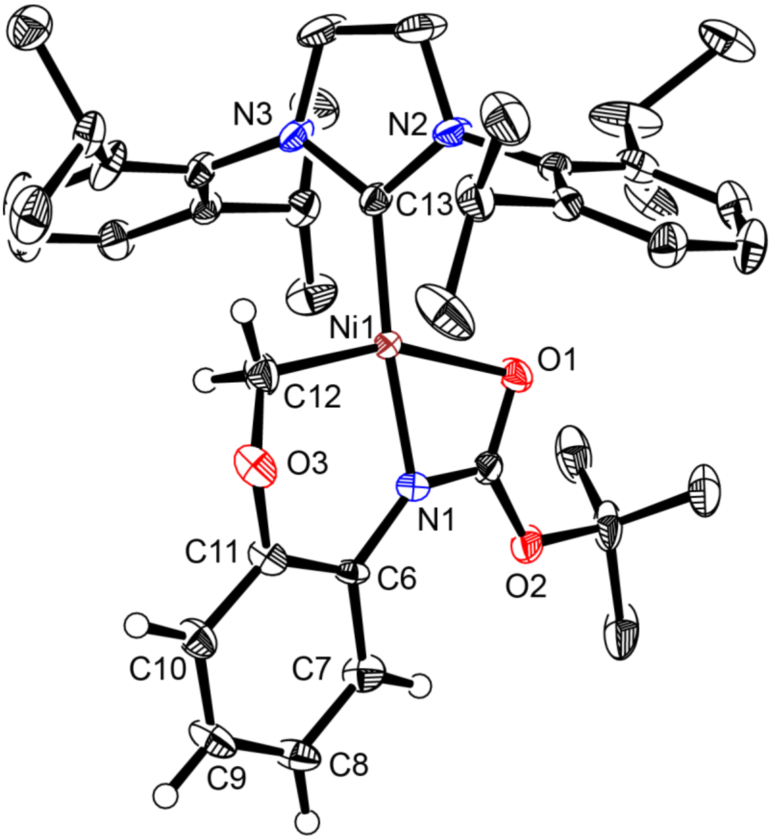

Supplementary Figure 48. ORTEP plot of **Ni-12** in 50% thermal ellipsoids, with H atoms omitted for clarity except for those in the metallacycle. Two iPr groups and the C_6H_4 unit are disordered over two alternate orientations (not shown). Bond angles ∠C12–Ni1–N1: 91.83(6)°, ∠C12–Ni1–C13: 95.15(6)°. Bond distances (Å) Ni1–C12: 1.887(1), Ni1–N1: 1.868(1), Ni1–O1: 2.120(1), Ni1–C13: 1.872(1).

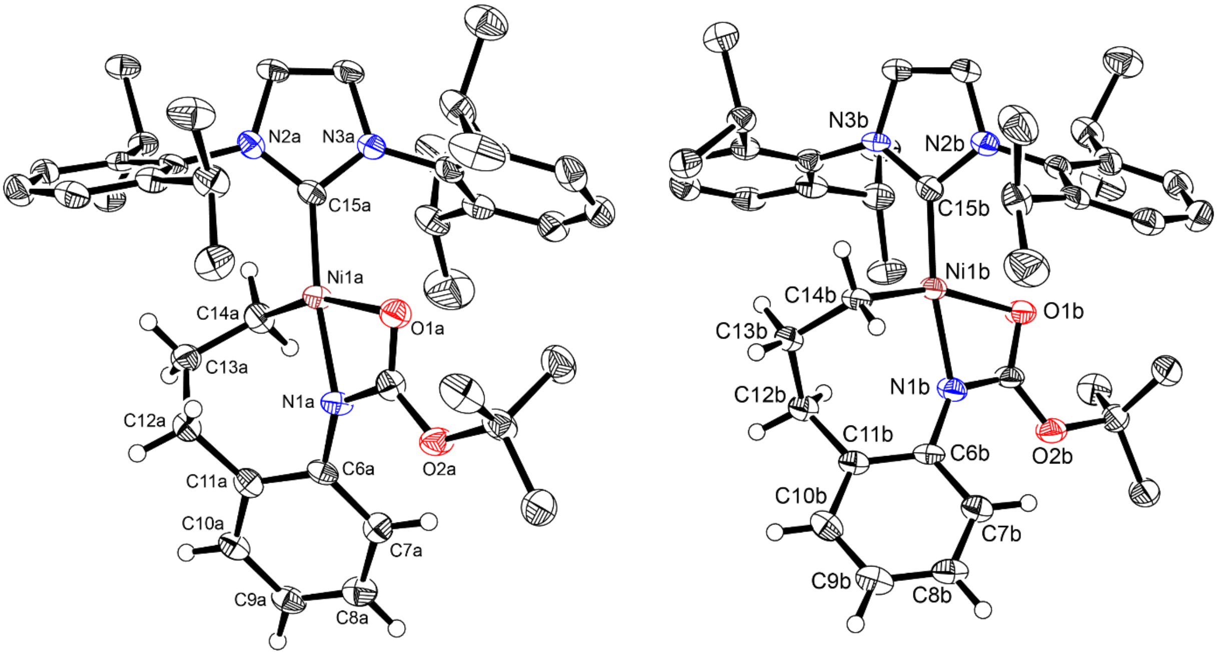

Supplementary Figure 49. ORTEP plot of both independent Ni moieties in asymmetric unit of **Ni-13**, in 50% thermal ellipsoids, with H atoms omitted for clarity except for those in the metallacycle. When one is inverted, the two moieties can almost perfectly be overlapped, but there is no crystallographic symmetry relation between them. The crystals were systematically twinned, but could be unfolded, and detwinned HKLF4 format data yielded the best refinement results (refined BASF 0.42). The asymmetric unit additionally contains a disordered toluene molecule (not shown). Further solvent, probably two more toluene molecules, was too disordered to model and thus removed using the masking procedure inbuilt in Olex2.[6] Bond angles ∠C14A–Ni1A–N1A: 95.25(2)°, ∠C14A–Ni1A–C15A: 92.94(2)°, ∠C14B–Ni1B–N1B: 95.3(2)°, ∠C14B–Ni1B–C15B: 92.7(2)°. Bond distances (Å) Ni1A–C14A: 1.923(4), Ni1A–N1A: 1.922(4), Ni1A–O1A: 2.086(3), Ni1A–C15A: 1.857(4), Ni1B–C14B: 1.926(4), Ni1B–N1B: 1.918(4), Ni1B–O1B: 2.095(3), Ni1B–C15B: 1.856(4).

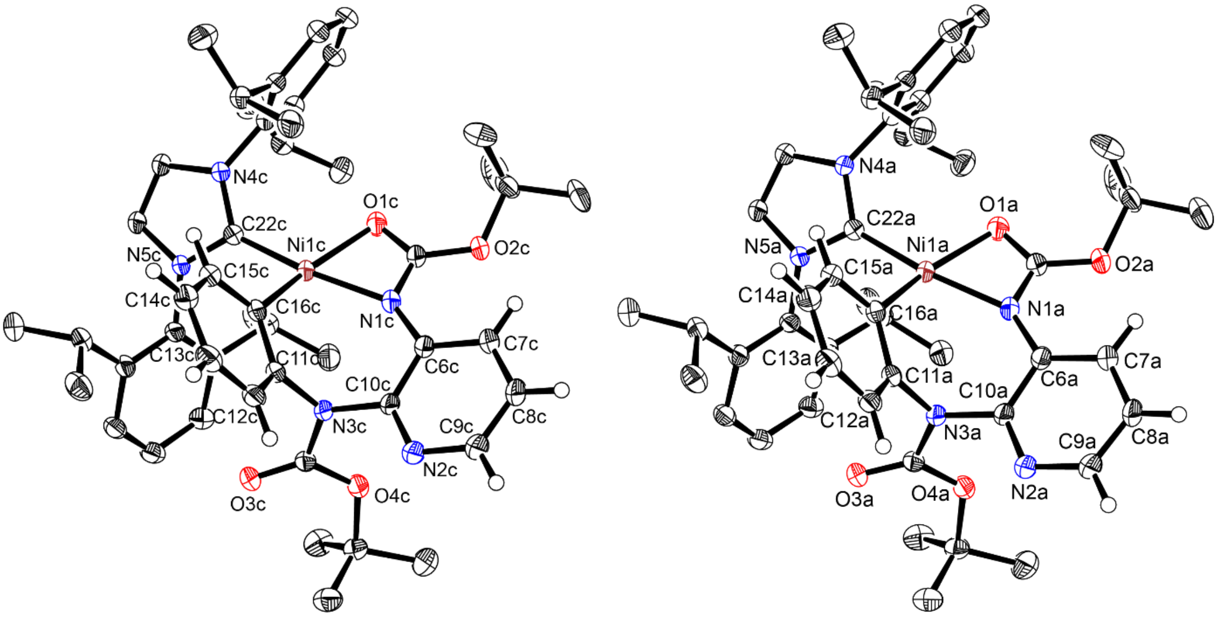

Supplementary Figure 50. ORTEP plot of both independent Ni moieties in asymmetric unit of **Ni-14**, in 50% thermal ellipsoids with H atoms omitted for clarity except for those in the metallacycle. The two moieties can almost perfectly be overlapped, and both exhibit a disorder of the ^{t}Bu group. Furthermore, the asymmetric unit contains 3.5 toluene molecules (not shown). Two toluenes exhibit regular disorder, one is not disordered, and the remainder is disordered about a crystallographic inversion center. Bond angles ∠C16A–Ni1A–N1A: 98.68(7)°, ∠C16A–Ni1A–C22A: 92.92(7)°; ∠C16C–Ni1C–N1C: 98.94(7)°, ∠C16C–Ni1C–C22C: 92.66(8)°. Bond distances (Å) Ni1A–C16A: 1.887(2), Ni1A–N1A: 1.936(2), Ni1A–O1A: 2.207(2), Ni1A–C22A: 1.873(2); Ni1C–C16C: 1.88(2), Ni1C–N1C: 1.937(2), Ni1C–O1C: 2.206(2), Ni1C–C22C: 1.870(2).

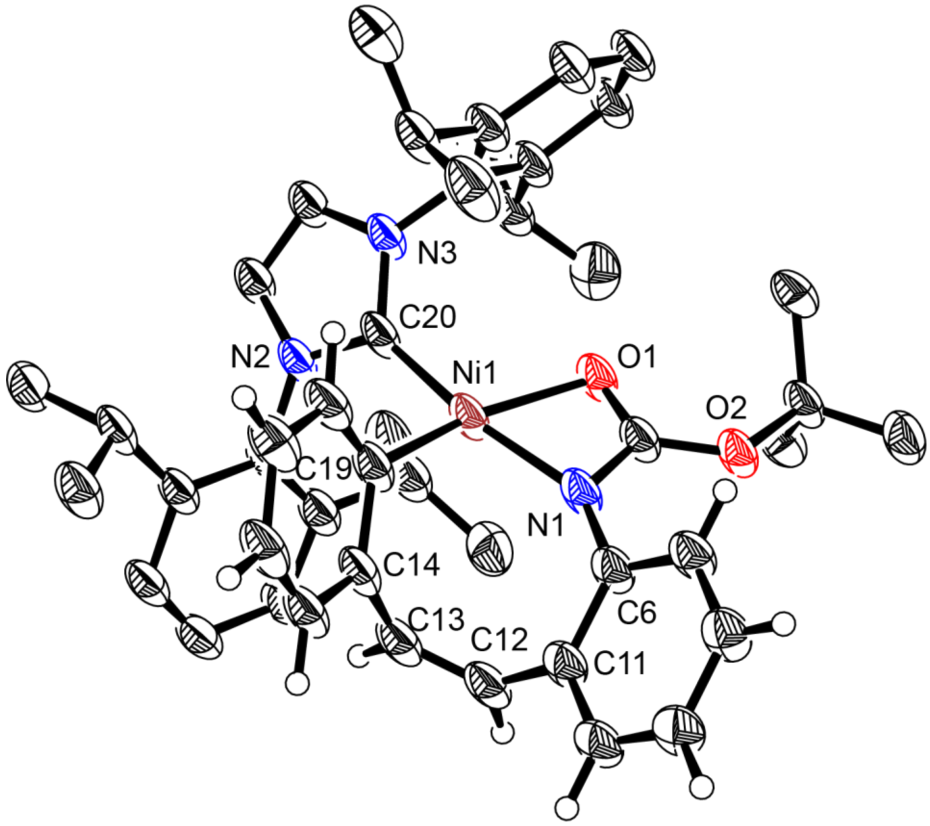

Supplementary Figure 51. ORTEP plot of **Ni-15** in 50% thermal ellipsoids, with H atoms omitted for clarity except for those in the metallacycle. The asymmetric unit additionally contains one disordered *n*-hexane molecule in total (not shown). One solvent position is shared between 0.5 *n*-hexane on a general position and 0.25 *n*-hexane disordered about a crystallographic inversion center. The remaining 0.25 *n*-hexane is disordered about a crystallographic 2-fold axis. Bond angles ∠C19–Ni1–N1: 97.2(1)°, ∠C19–Ni1–C20: 94.2(1)°. Bond distances (Å) Ni1–C19: 1.896(2), Ni1–N1: 1.929(2), Ni1–O1: 2.039(2), Ni1–C20: 1.867(2).

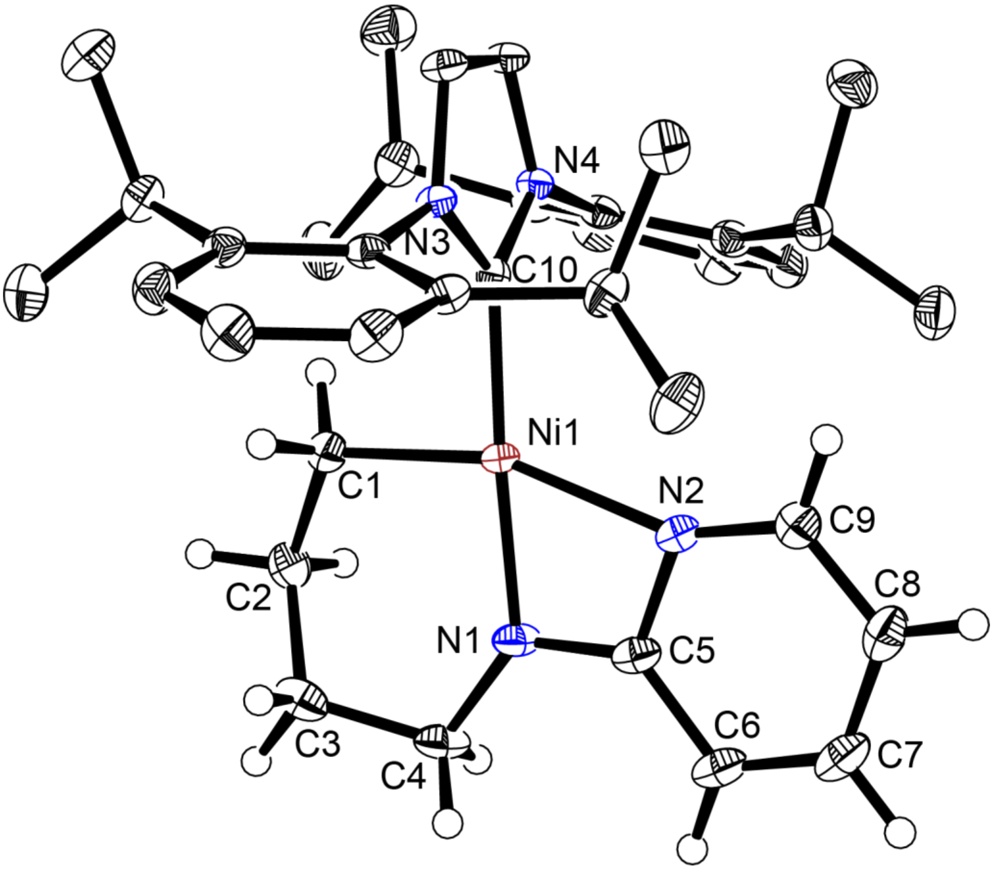

Supplementary Figure 52. ORTEP plot of **Ni-2-Py** in 50% thermal ellipsoids, with H atoms omitted for clarity except for those in the metallacycle. The asymmetric unit additionally contains a disordered toluene molecule (not shown). Bond angles ∠C1–Ni1–N1: 93.81(6)°, ∠C1–Ni1–C10: 89.00(6)°. Bond distances (Å): Ni1–C1: 1.929(1), Ni1–N1: 1.885(1), Ni1–N2: 2.082(1), Ni1–C10: 1.869(2).

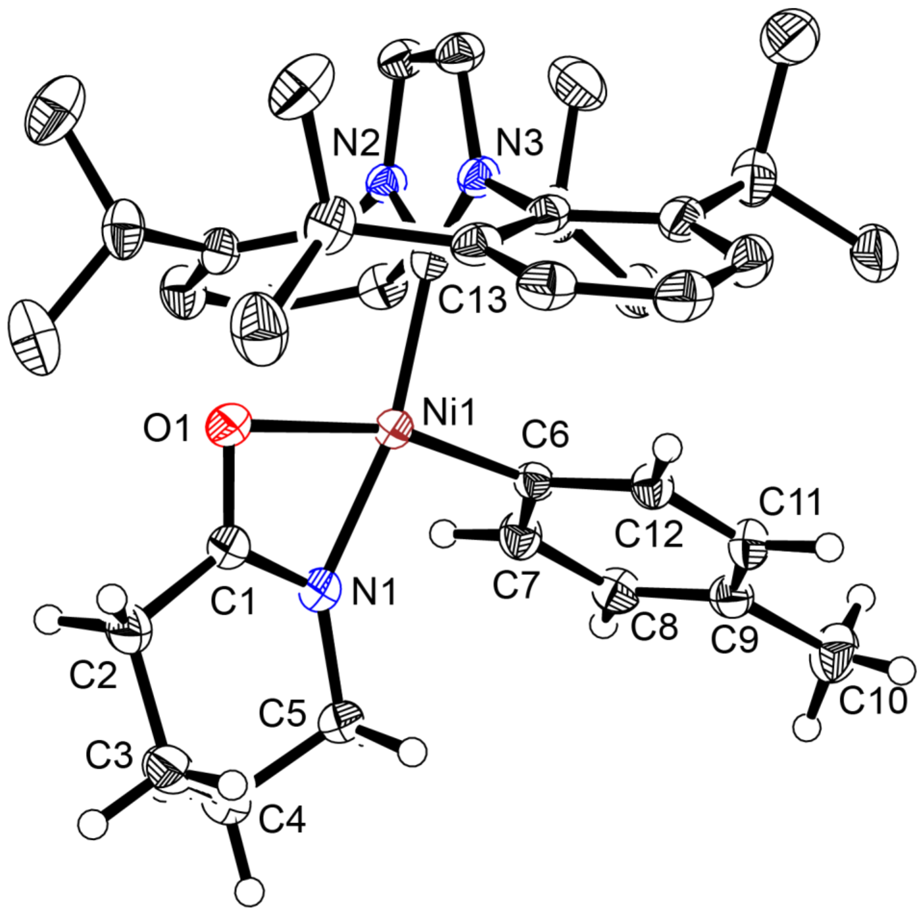

Supplementary Figure 53. ORTEP plot of **Ni-2-Ts** in 50% thermal ellipsoids, with H atoms omitted for clarity except for those that belong to **2-Ts**. The hydrogens at C10 are disordered by pseudorotation. Bond angles ∠C13–Ni1–C6: 96.66(7)°, ∠C13–Ni1–O1: 100.08(6)°. Bond distances (Å): Ni1–C6: 1.868(2), Ni1–C13: 1.873(2), Ni1–N1: 1.905(1), Ni1–O1: 2.035(1).

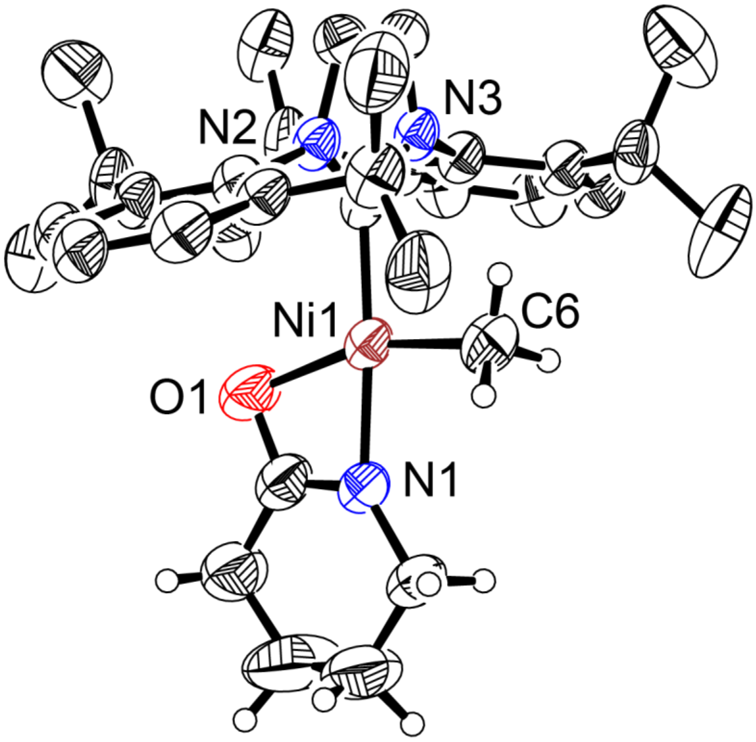

Supplementary Figure 54. ORTEP plot of preliminary structure for **Ni-2-Ac** in 50% thermal ellipsoids, with H atoms omitted for clarity except for those that belong to **2-Ac**. The crystals were systematically twinned. Overall data quality is considered too poor for a full publication, and the structure is not discussed in detail herein, nor uploaded to the CCDC. Nevertheless, at least the connectivity of nickel complex **Ni-2-Ac** could be established.

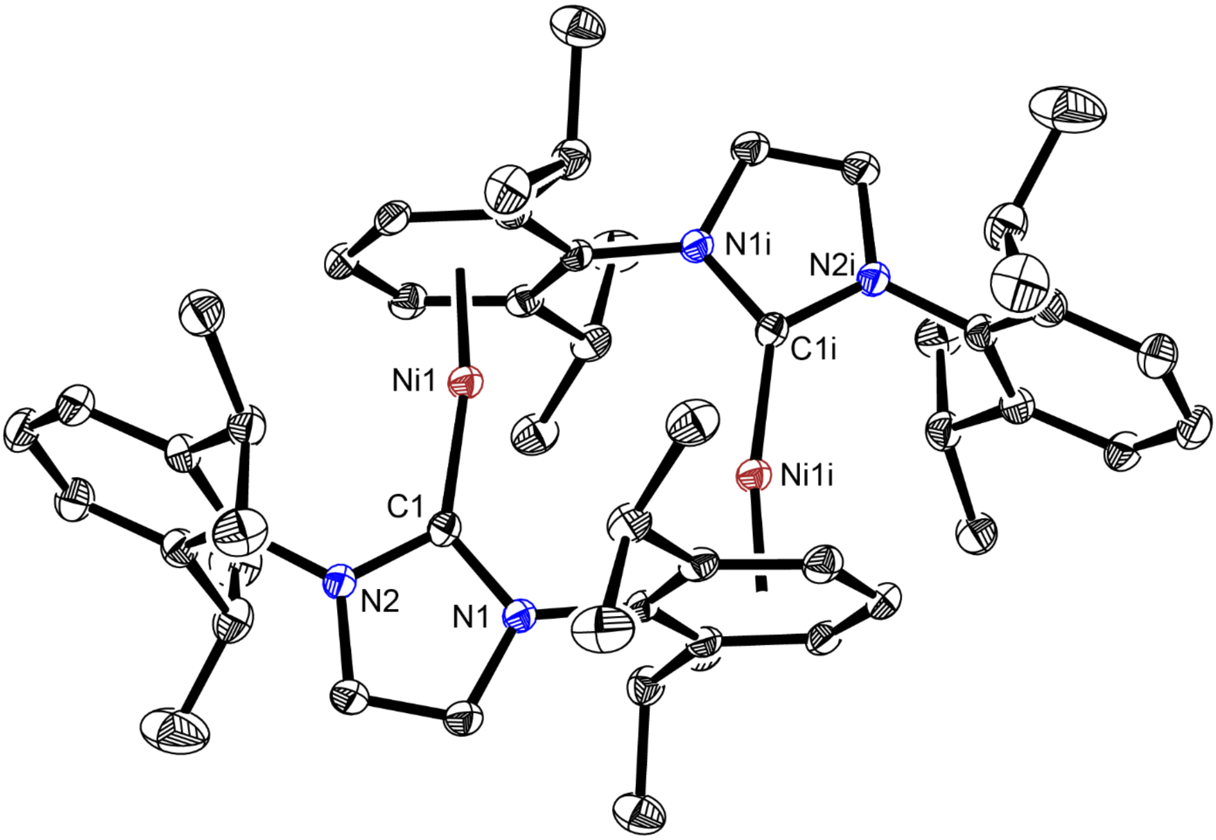

Supplementary Figure 55. ORTEP plot of **$(iPrNi)_2$** in 50% thermal ellipsoids, with H atoms omitted for clarity. The complex has been reported via a different synthesis.[8] The molecule is located on a crystallographic inversion center, symmetry-generated atoms are marked with suffix *i*.

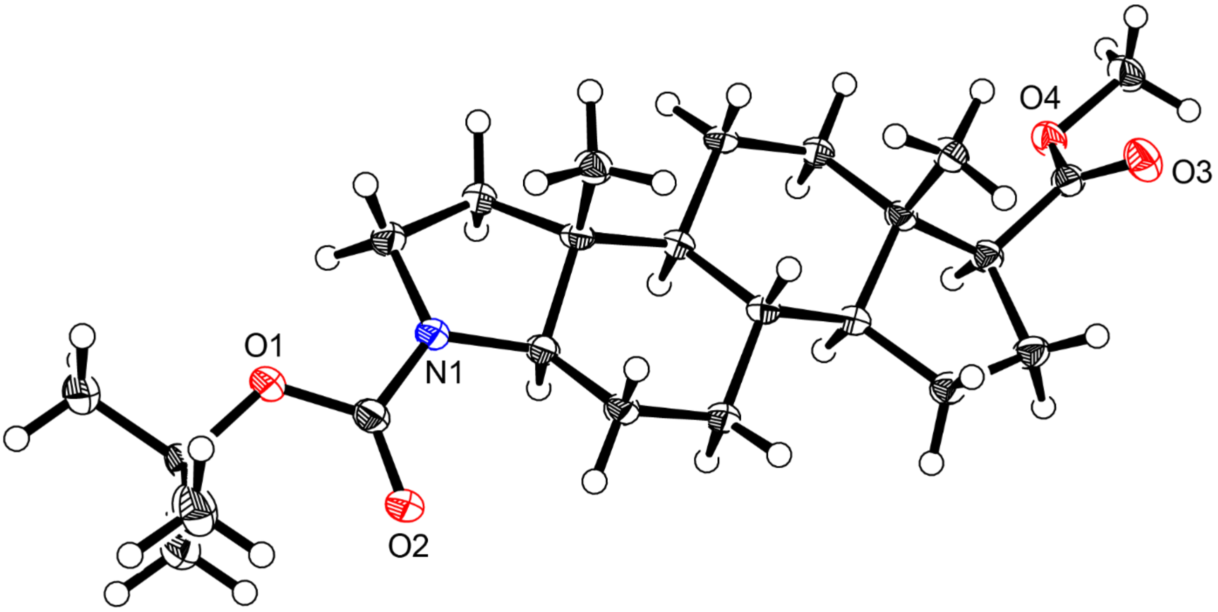

Supplementary Figure 56. ORTEP plot of **19a** in 50% thermal ellipsoids. Absolute structure was determined from anomalous signal in the structure analysis.

7. NMR spectra of organometallic compounds

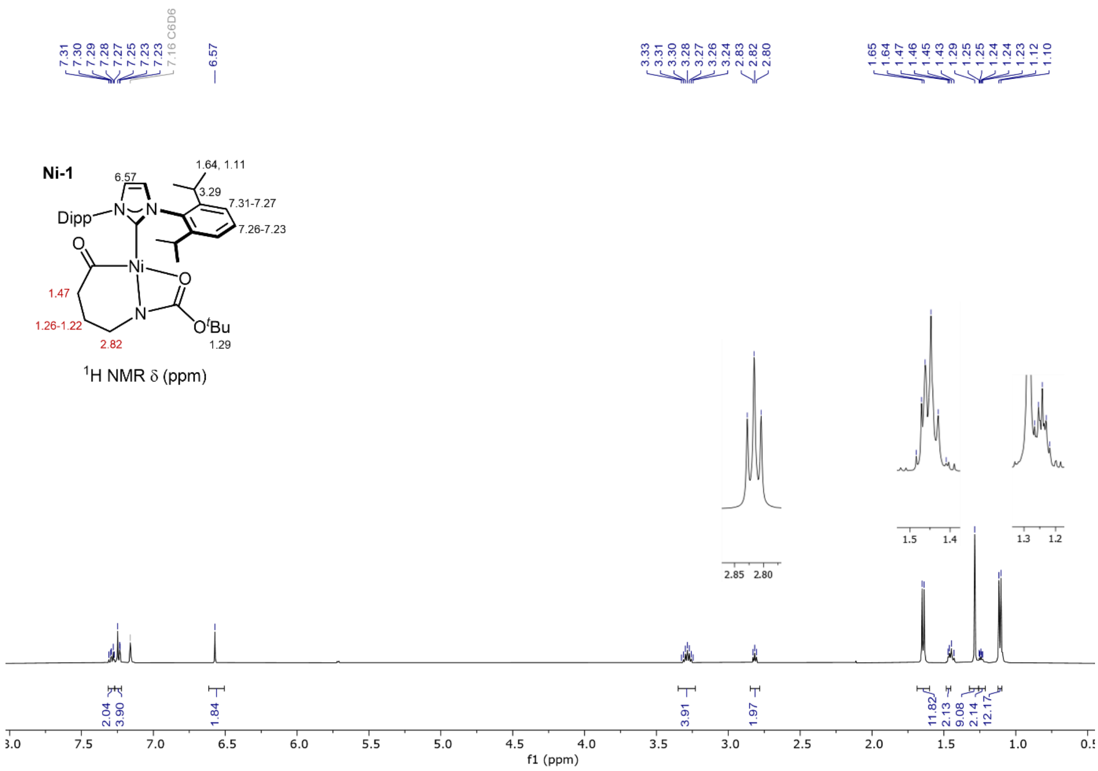

Supplementary Figure 57. ^{1}H NMR spectrum of **Ni-1** (500 MHz, benzene-d_6, 23 °C).

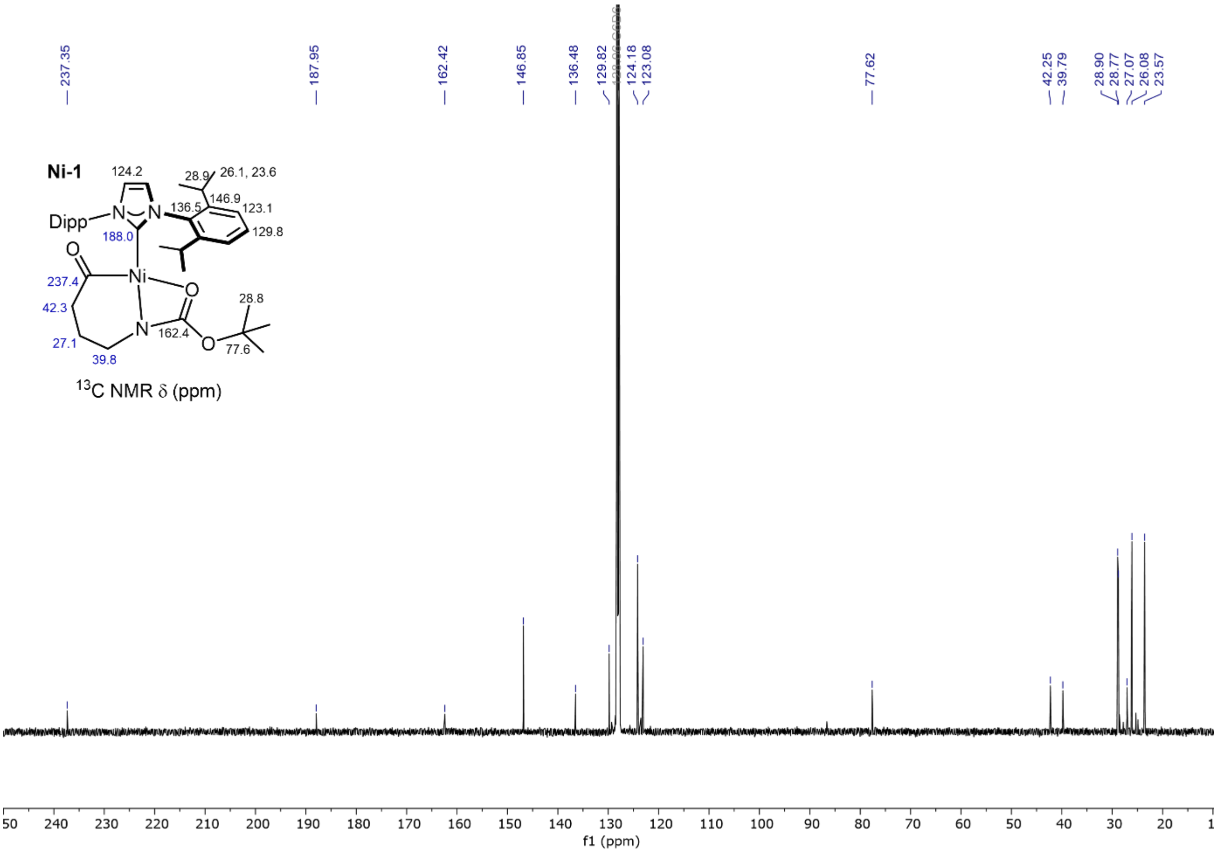

Supplementary Figure 58. ^{13}C{^{1}H} NMR spectrum of **Ni-1** (126 MHz, benzene-d_6, 23 °C).

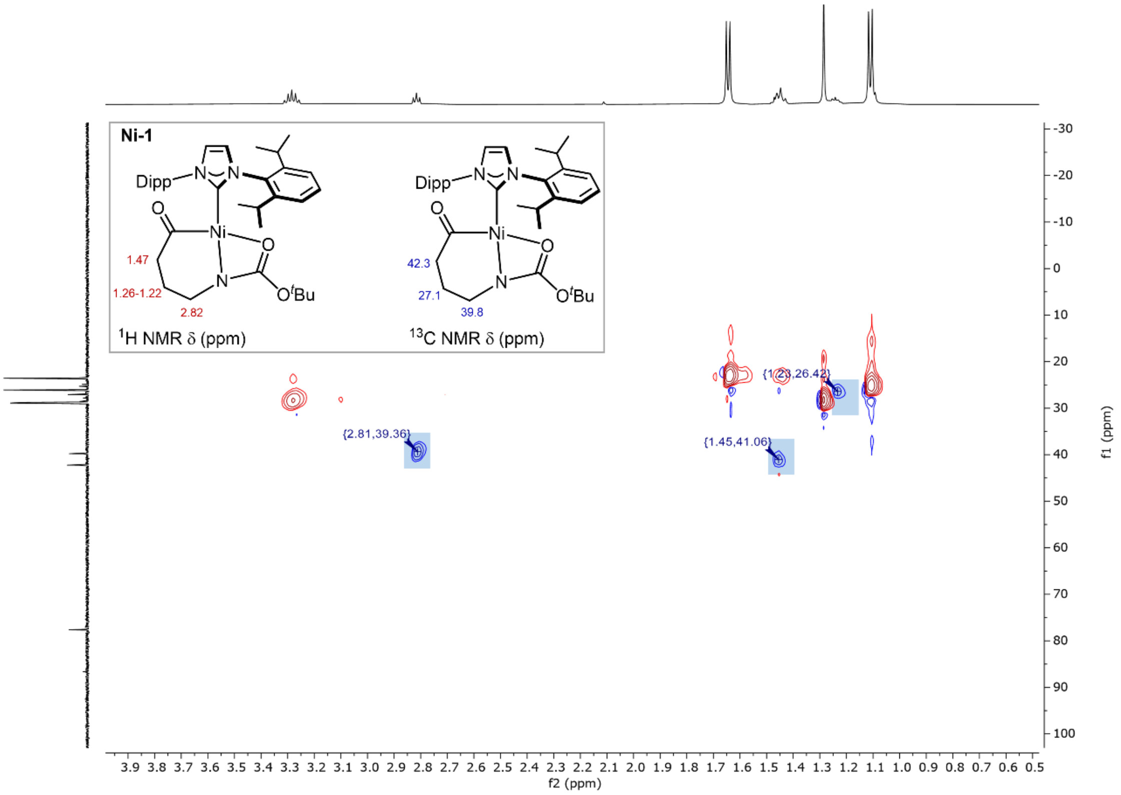

Supplementary Figure 59. Selected region of the HSQC spectrum of **Ni-1** displaying correlations between nickellacycle methylene protons and carbons (benzene-d_6, 23 °C).

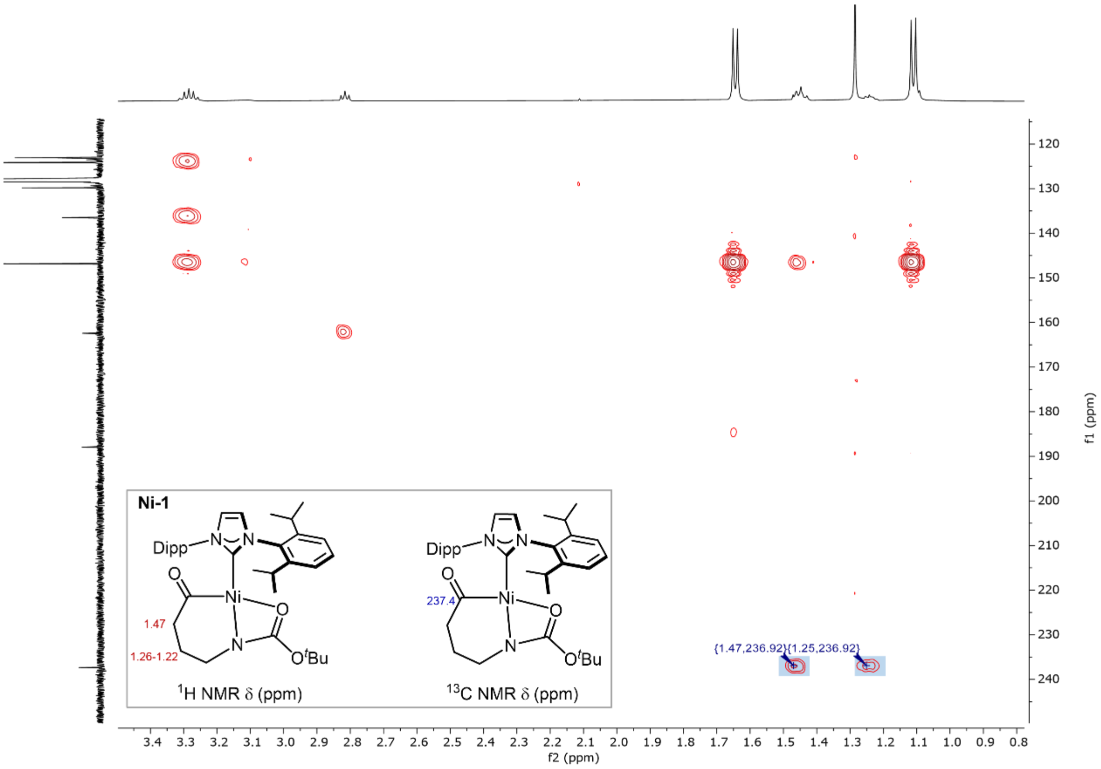

Supplementary Figure 60. Selected region of the HMBC spectrum of **Ni-1** displaying correlations between nickellacycle methylene protons and the acyl carbon (benzene-d_6, 23 °C).

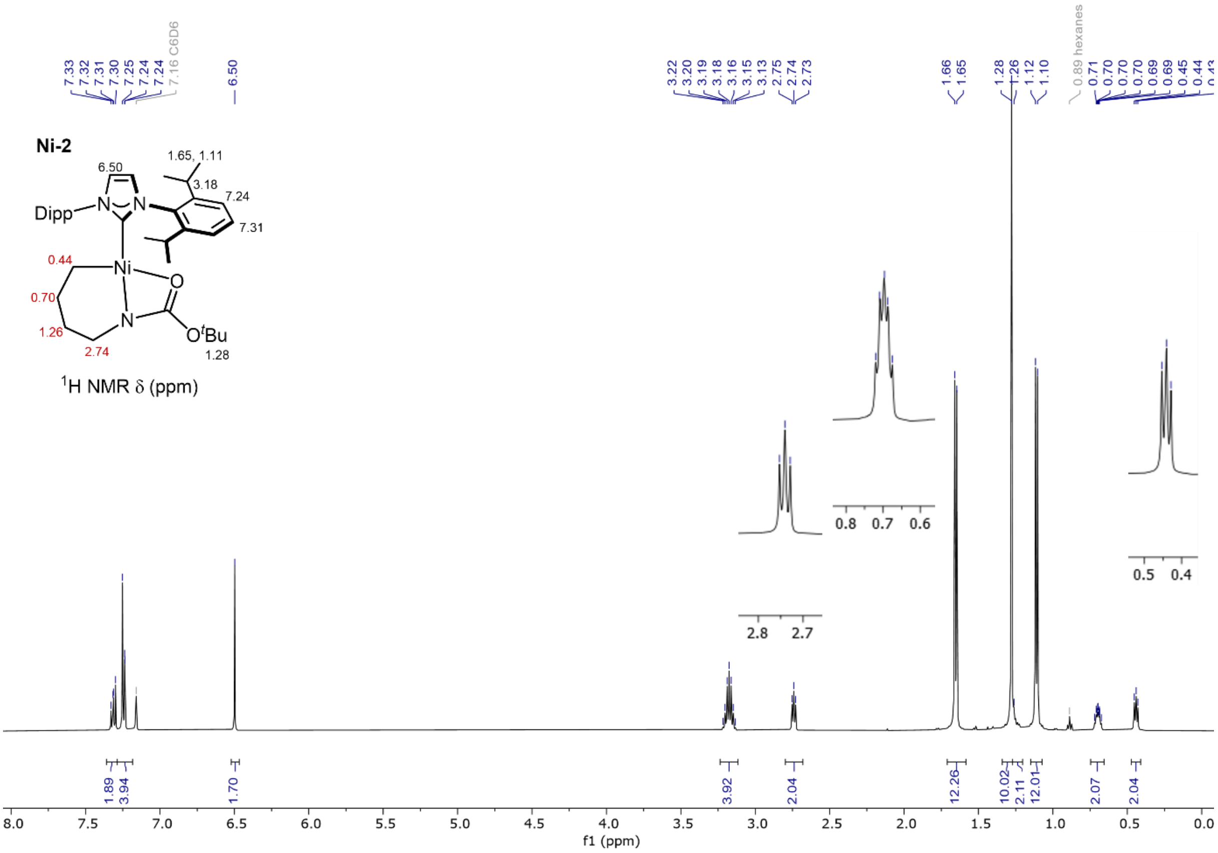

Supplementary Figure 61. ^{1}H NMR spectrum of **Ni-2** (500 MHz, benzene-d_6, 23 °C).

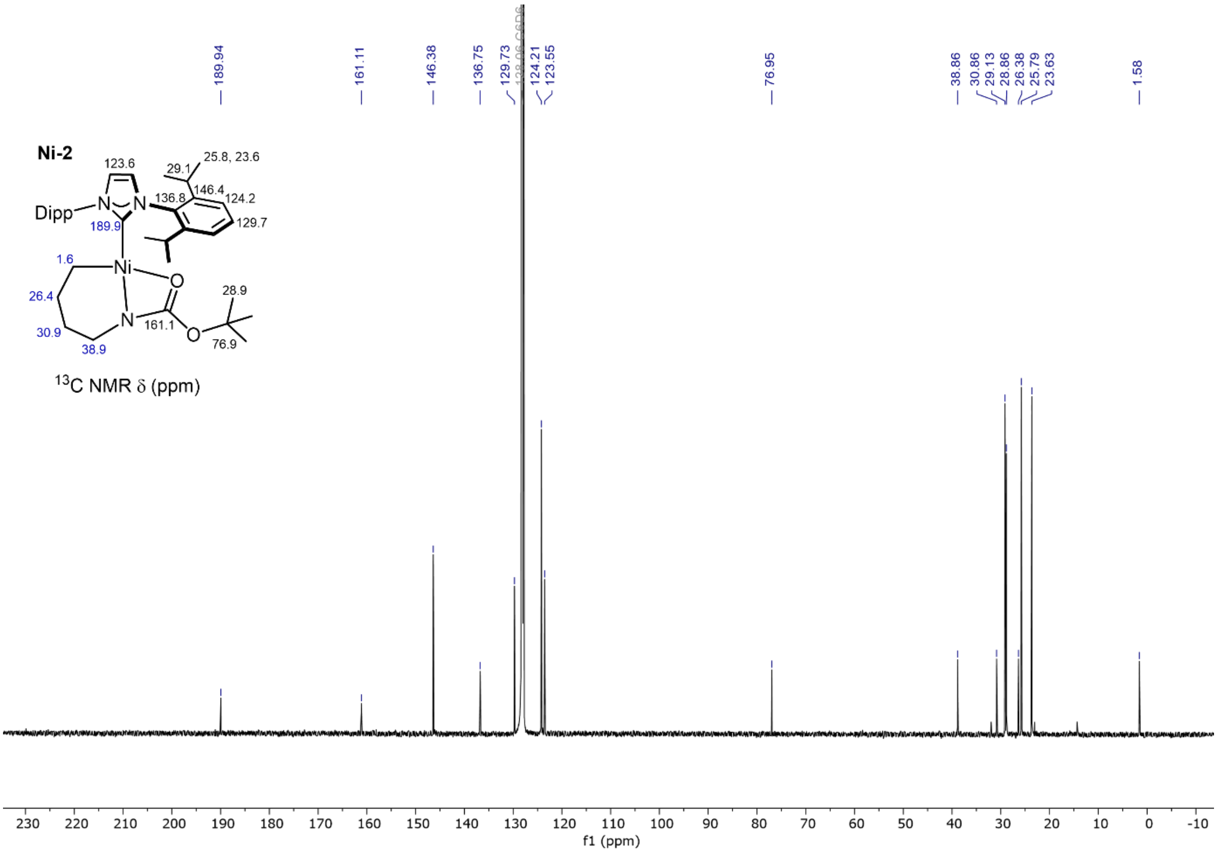

Supplementary Figure 62. ^{13}C{^{1}H} NMR spectrum of **Ni-2** (126 MHz, benzene-d_6, 23 °C).

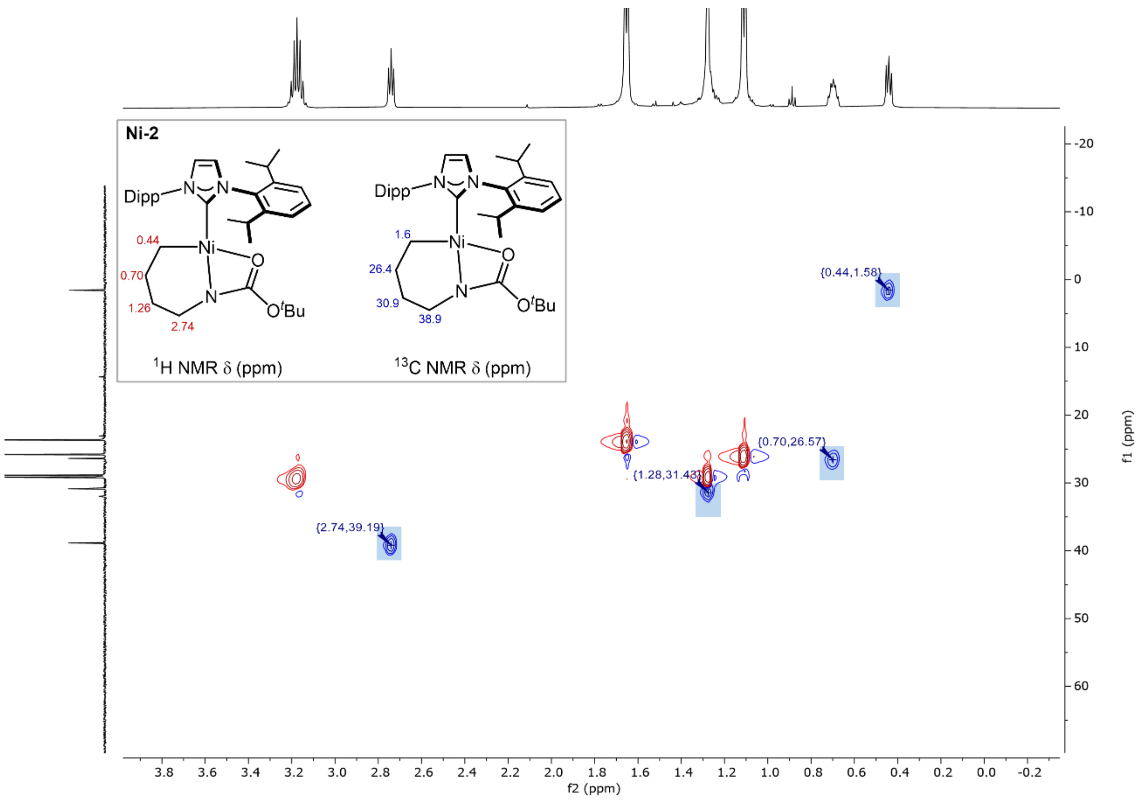

Supplementary Figure 63. Selected region of the HSQC spectrum of **Ni-2** displaying correlations between nickellacycle methylene protons and carbons (benzene-d_6, 23 °C).

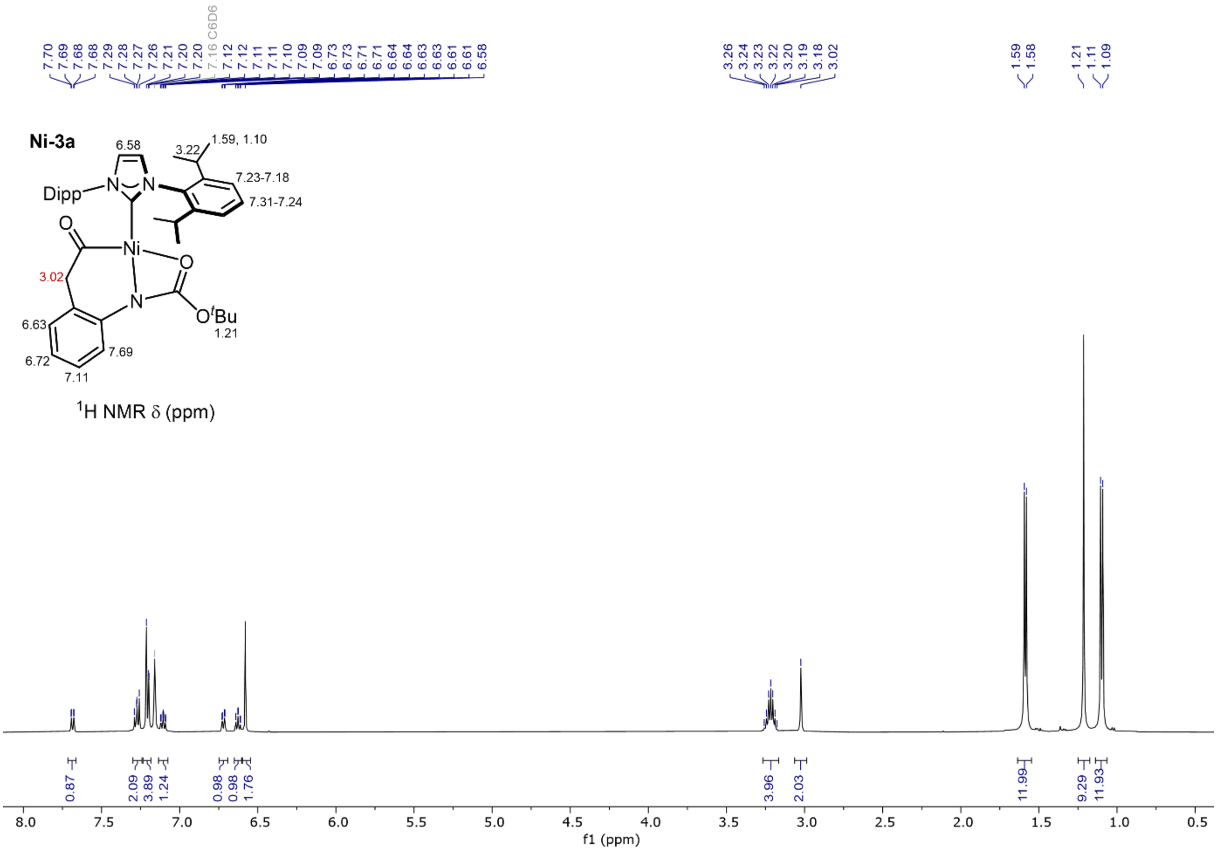

Supplementary Figure 64. ^{1}H NMR spectrum of **Ni-3a** (500 MHz, benzene-d_6, 23 °C).

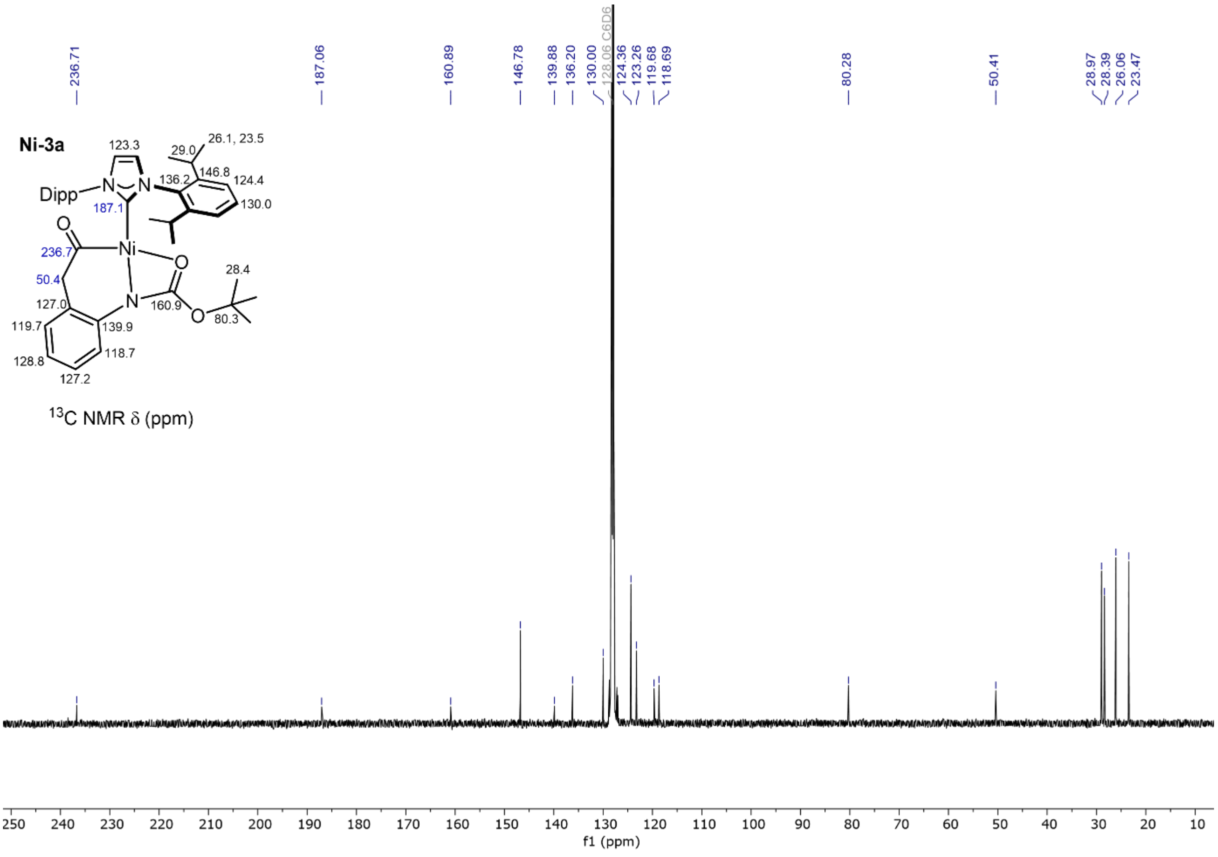

Supplementary Figure 65. ^{13}C{^{1}H} NMR spectrum of **Ni-3a** (126 MHz, benzene-d_6, 23 °C).

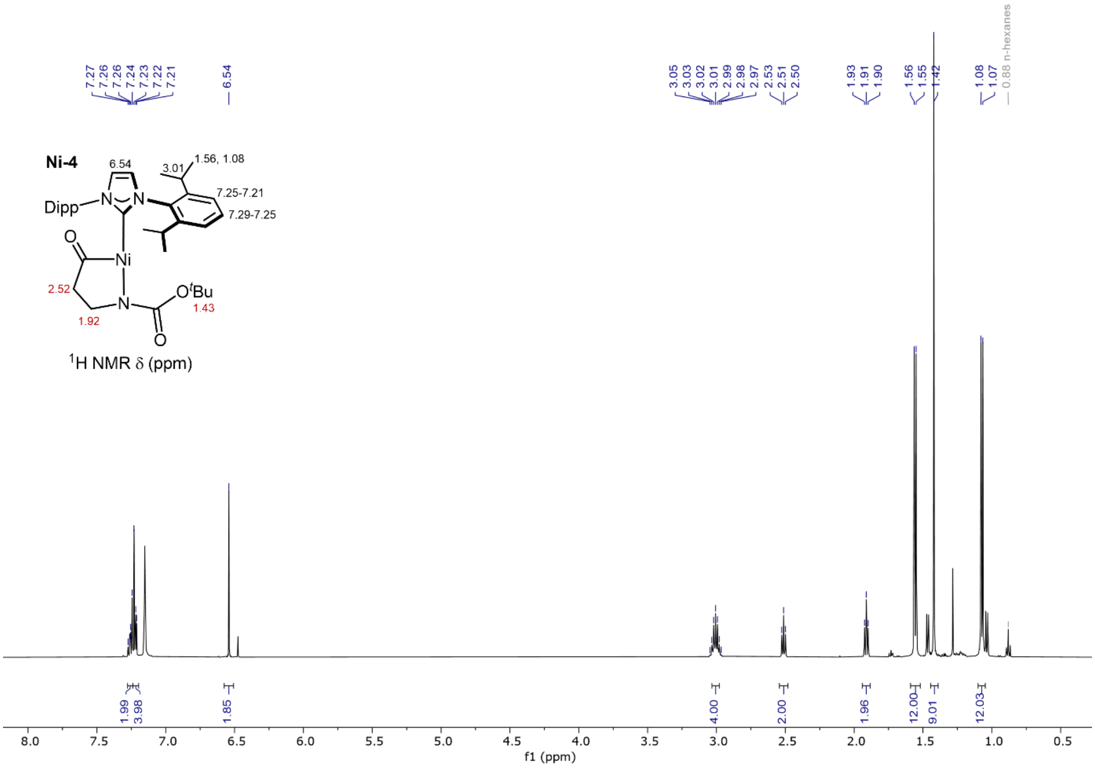

Supplementary Figure 66. ^{1}H NMR spectrum of **Ni-4** (126 MHz, benzene-d_6, 23 °C, a second nickel species(13%) is present and is assigned as the 4-coordinate complex indicated by the upfield-shifted ^{t}Bu group resonance at 1.28 ppm).

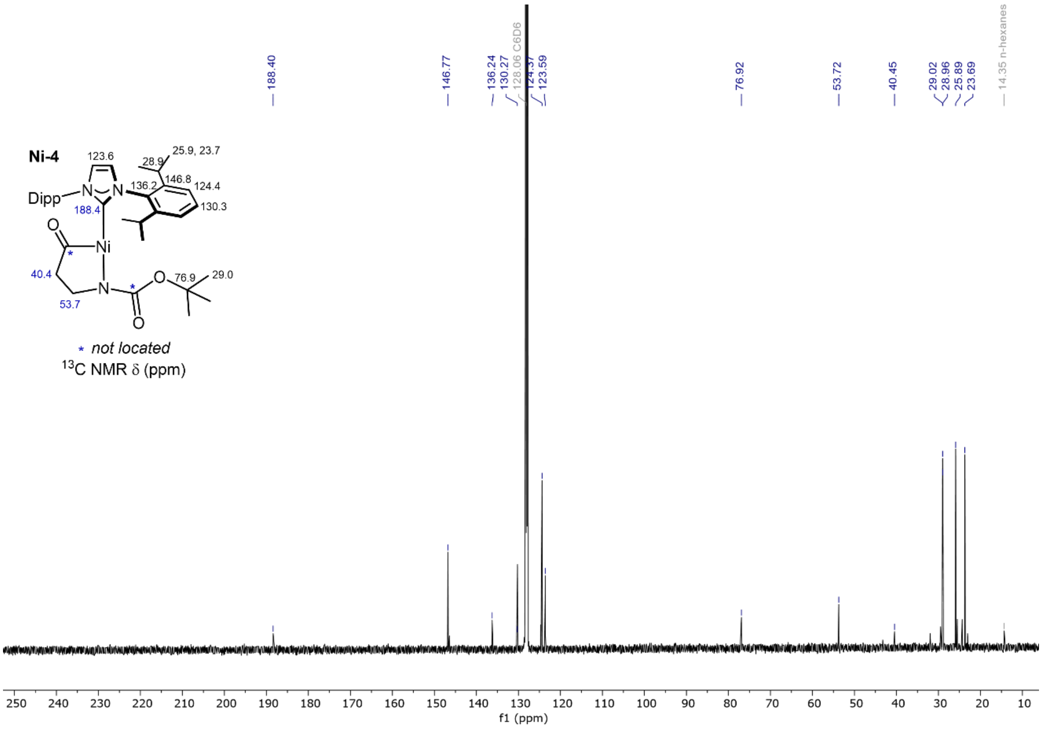

Supplementary Figure 67. ^{1}H NMR spectrum of **Ni-4** (126 MHz, benzene-d_6, 23 °C). The nickel acyl carbon signal and the Boc carbonyl carbon signal were not located.

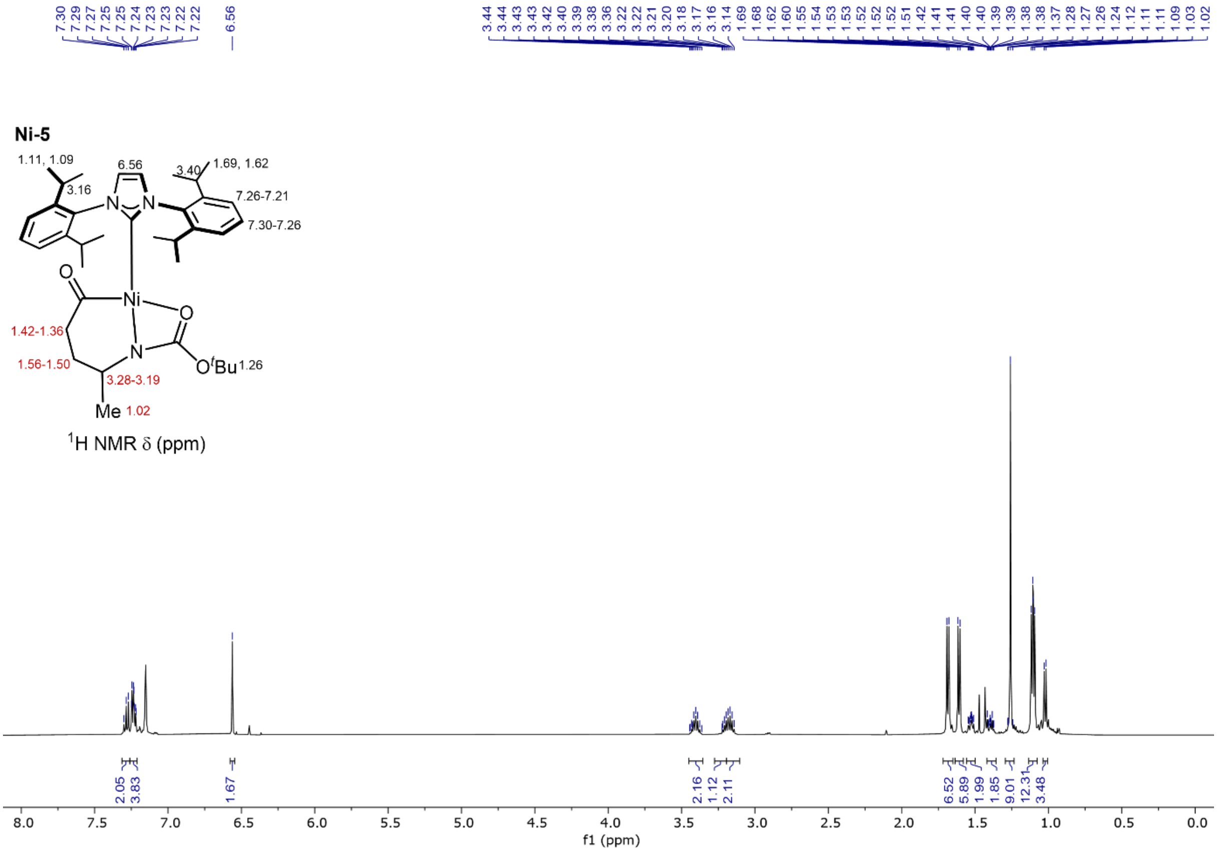

Supplementary Figure 68. ^{1}H NMR spectrum of **Ni-5** (500 MHz, benzene-d_6, 23 °C).

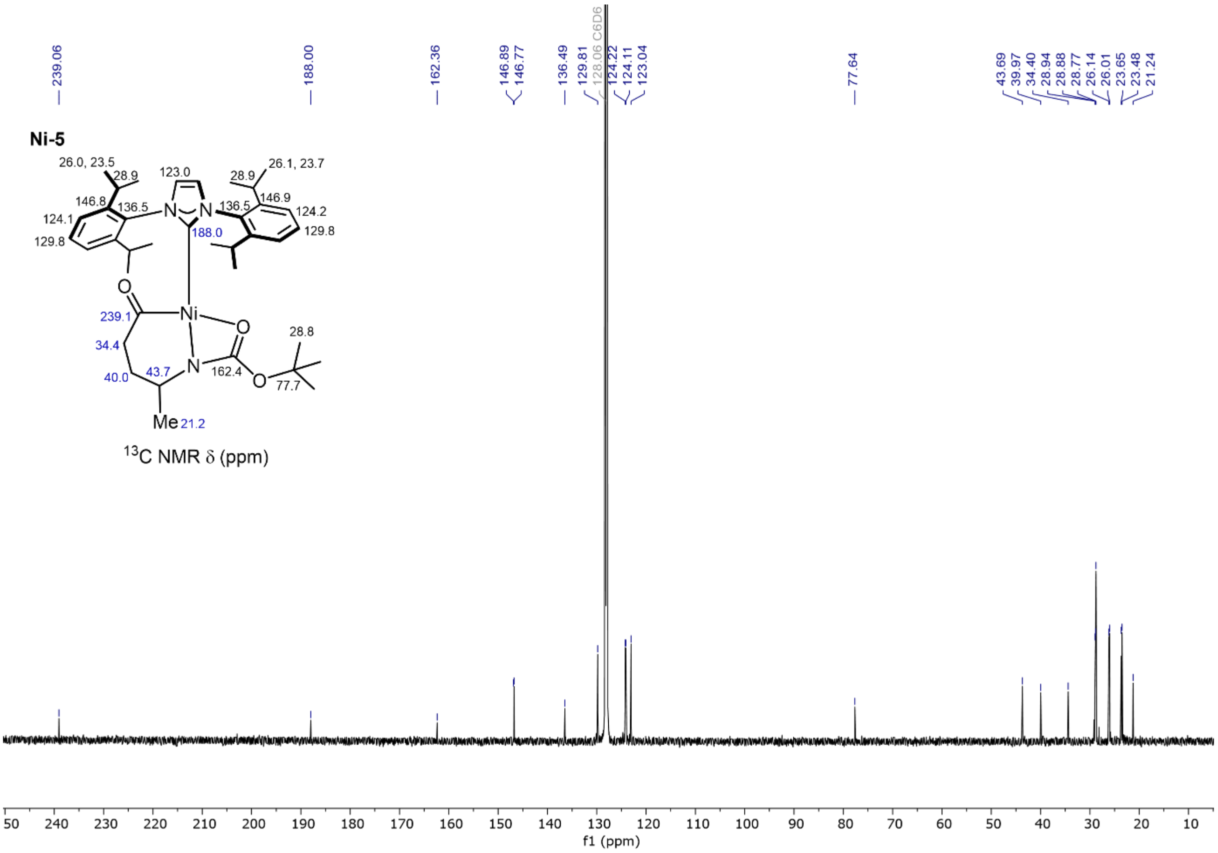

Supplementary Figure 69. ^{13}C{^{1}H} NMR spectrum of **Ni-5** (126 MHz, benzene-d_6, 23 °C).

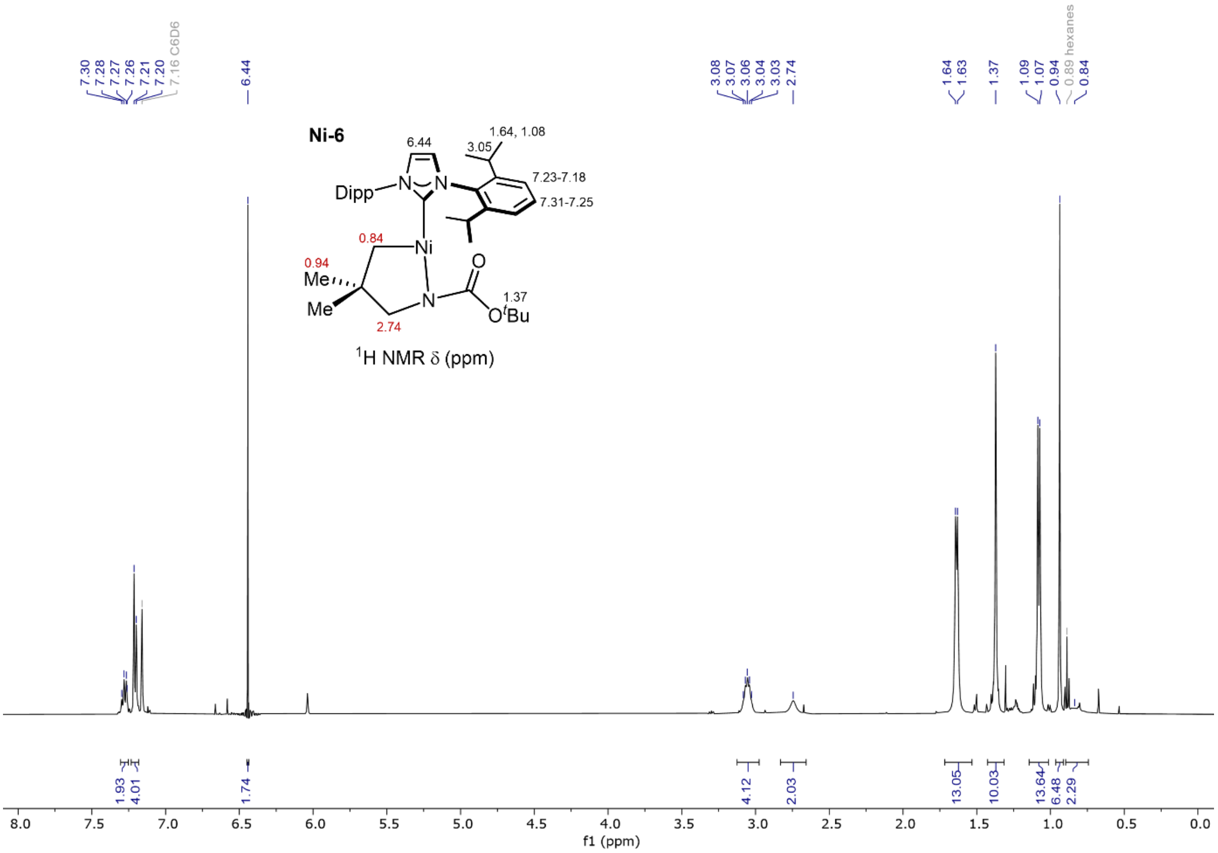

Supplementary Figure 70. ^{1}H NMR spectrum of **Ni-6** (500 MHz, benzene-d_6, 23 °C).

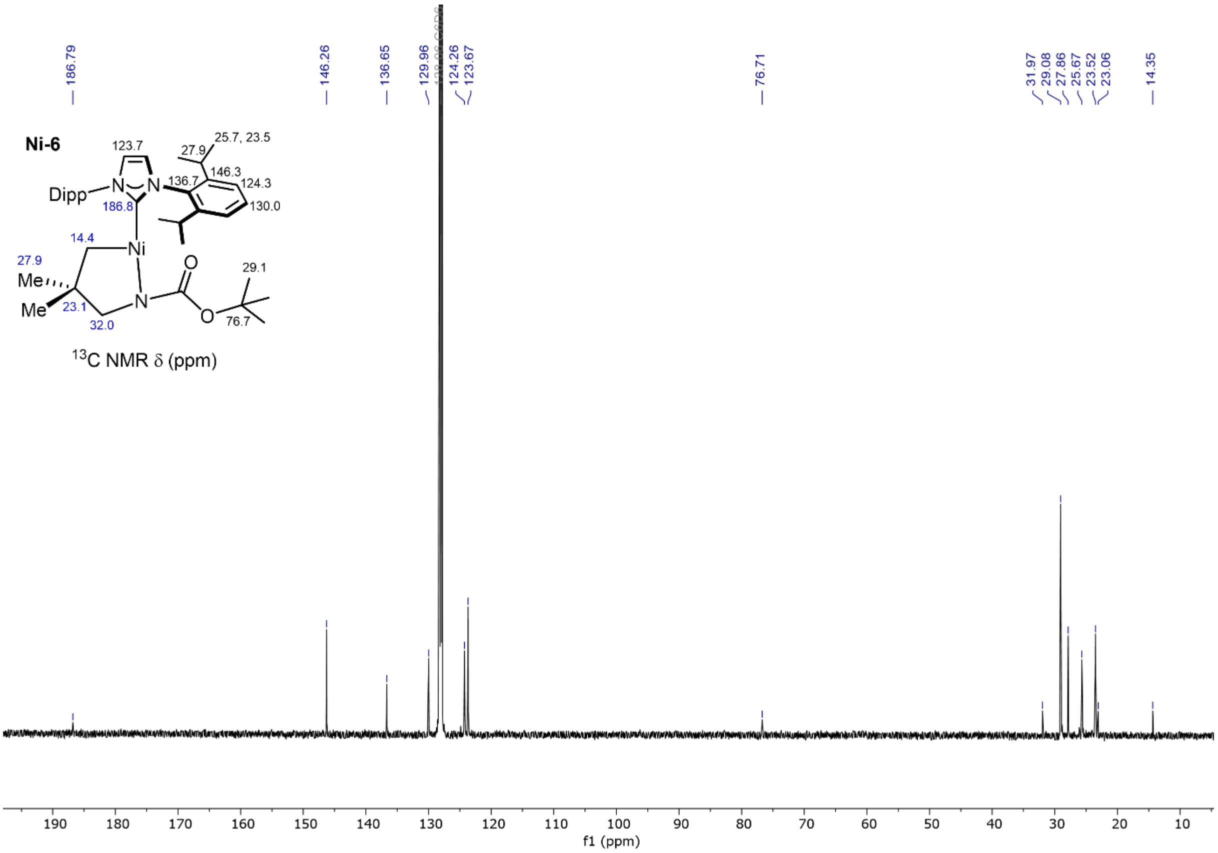

Supplementary Figure 71. ^{13}C{^{1}H} NMR spectrum of **Ni-6** (126 MHz, benzene-d_6, 23 °C).

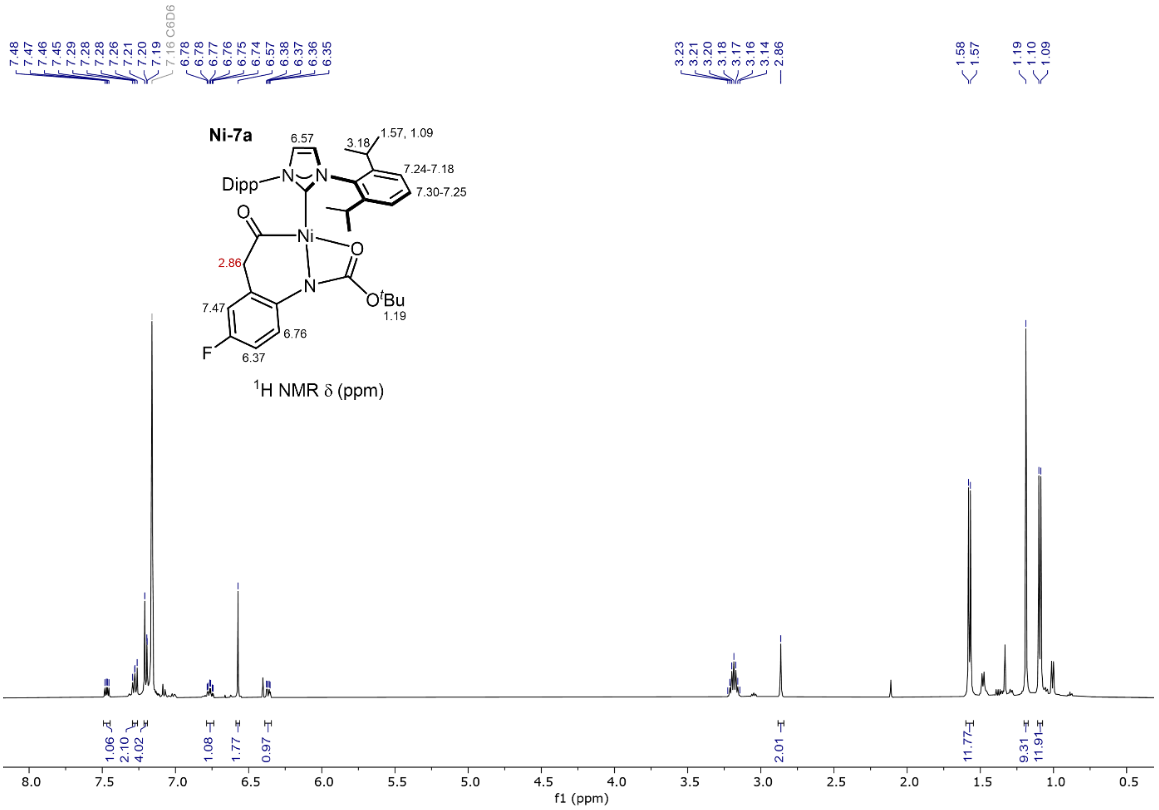

Supplementary Figure 72. ^{1}H NMR spectrum of **Ni-7a** (126 MHz, benzene-d_6, 23 °C, the nickel-alkyl complex **Ni-7b** (16%) is present).

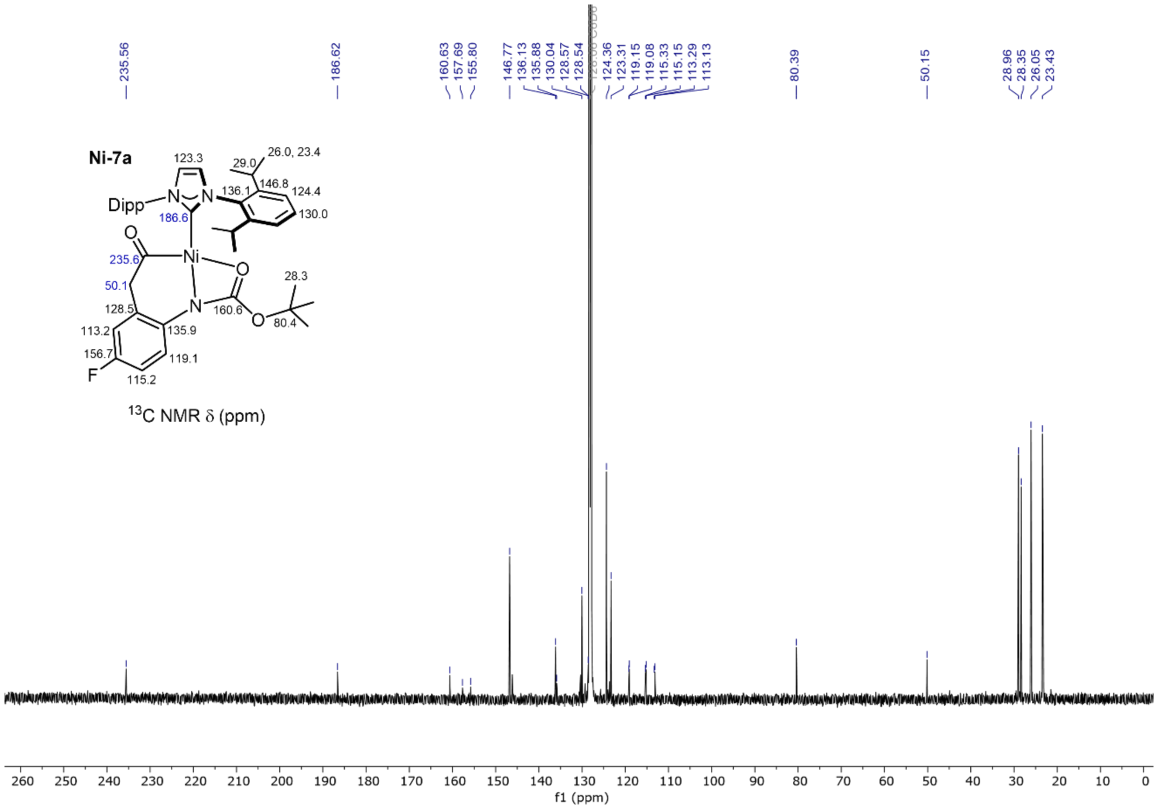

Supplementary Figure 73. ^{13}C{^{1}H} NMR spectrum of **Ni-7a** (126 MHz, benzene-d_6, 23 °C).

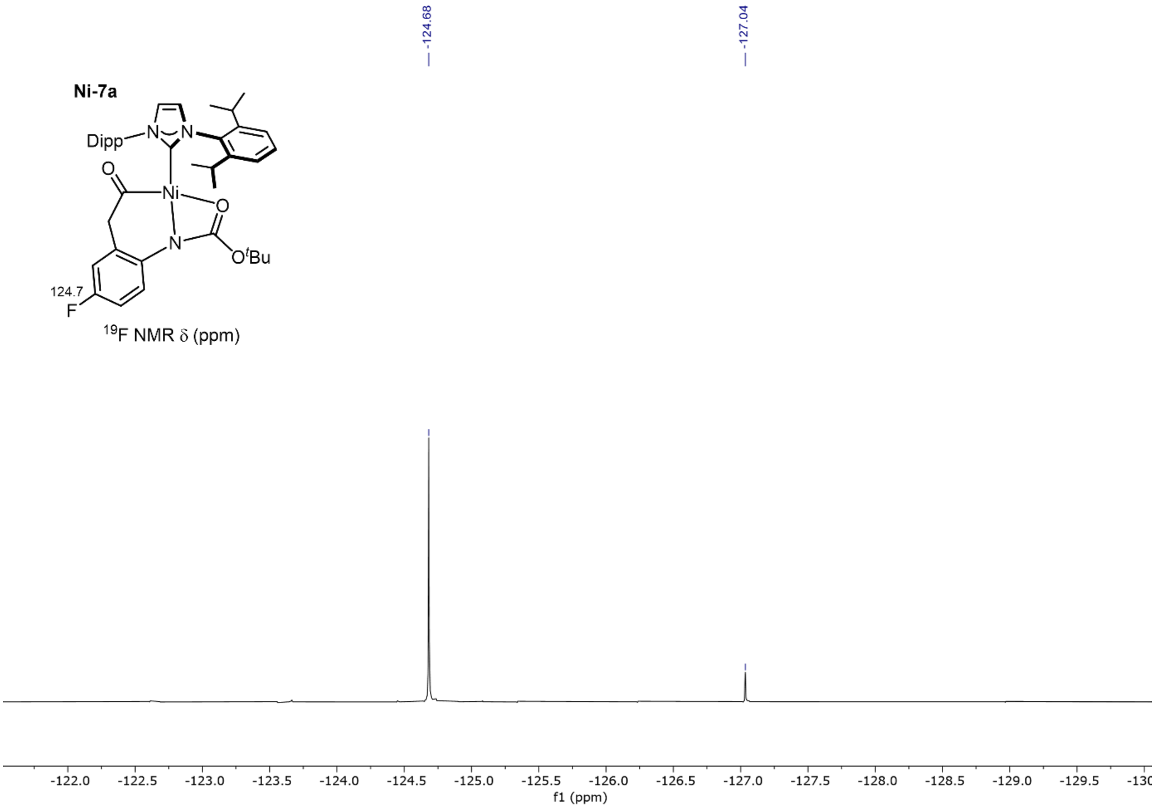

Supplementary Figure 74. $^{19}F\{^{1}H\}$ NMR spectrum of **Ni-7a** (376 MHz, benzene-d_6, 23 °C, which contains the minor nickel alkyl complex **Ni-7b** (11%)).

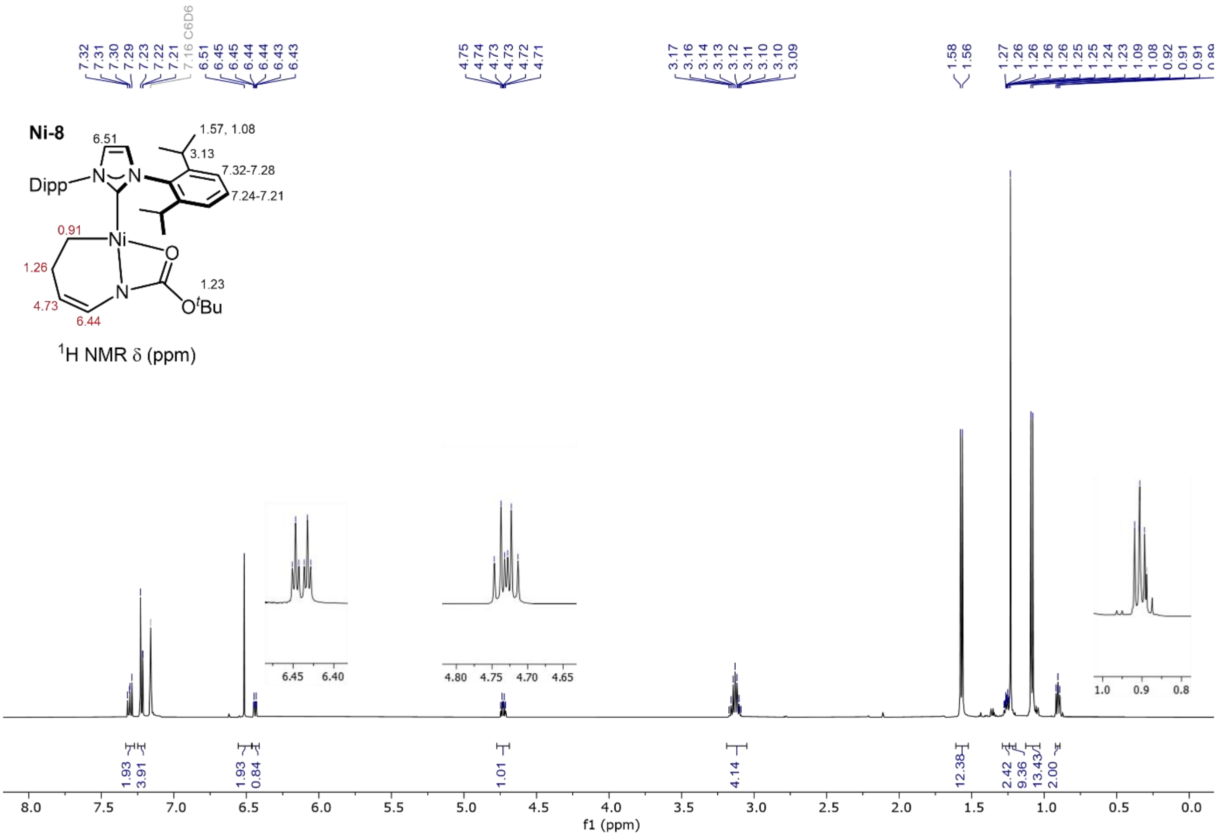

Supplementary Figure 75. ^{1}H NMR spectrum of **Ni-8** (500 MHz, benzene-d_6, 23 °C).

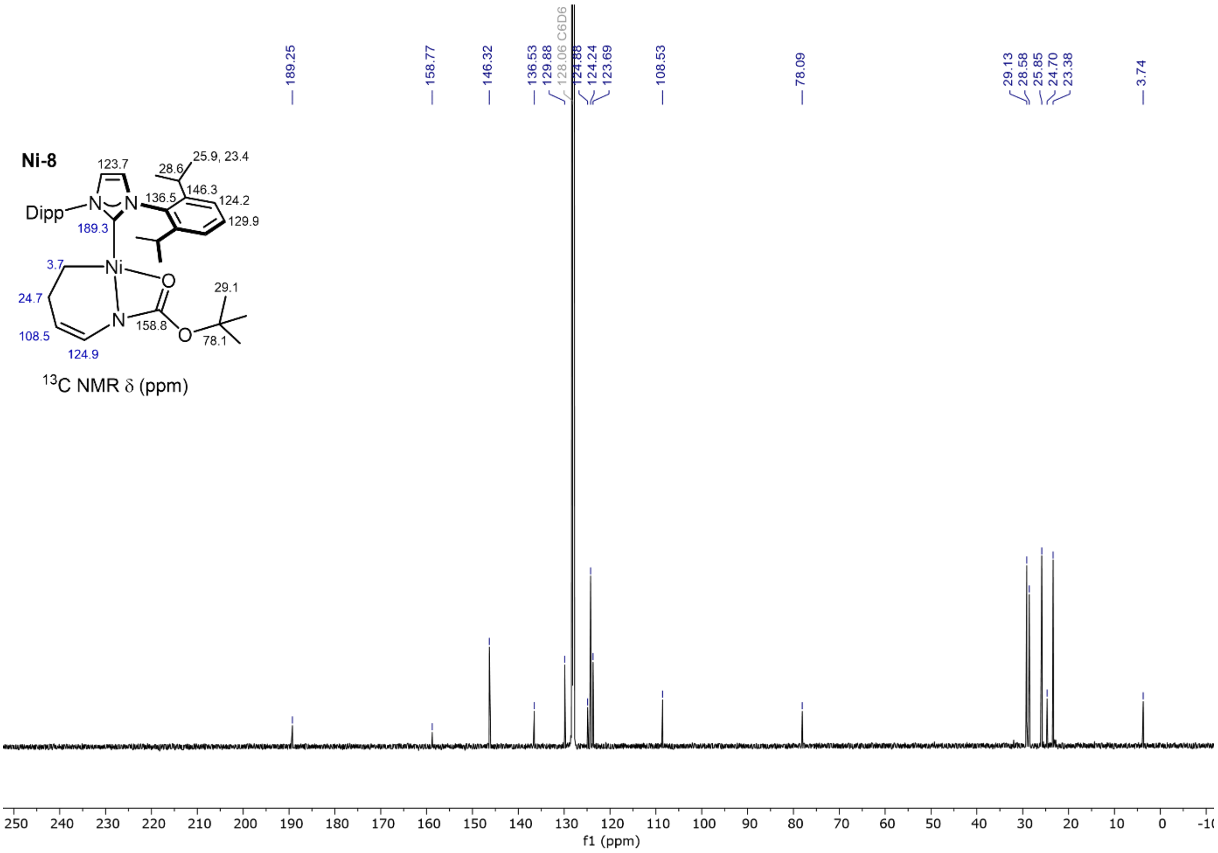

Supplementary Figure 76. ^{13}C{^{1}H} NMR spectrum of **Ni-8** (126 MHz, benzene-d_6, 23 °C).

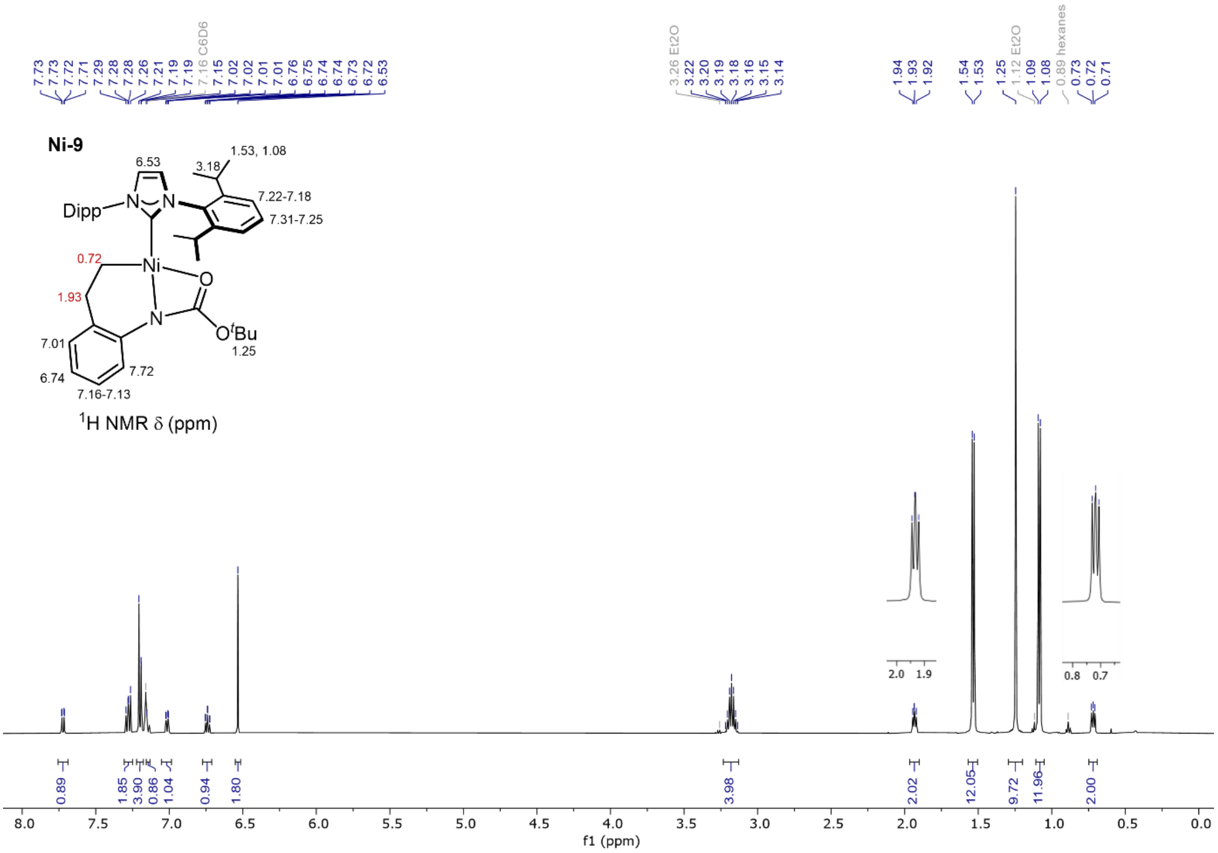

Supplementary Figure 77. ^{1}H NMR spectrum of **Ni-9** (500 MHz, benzene-d_6, 23 °C).

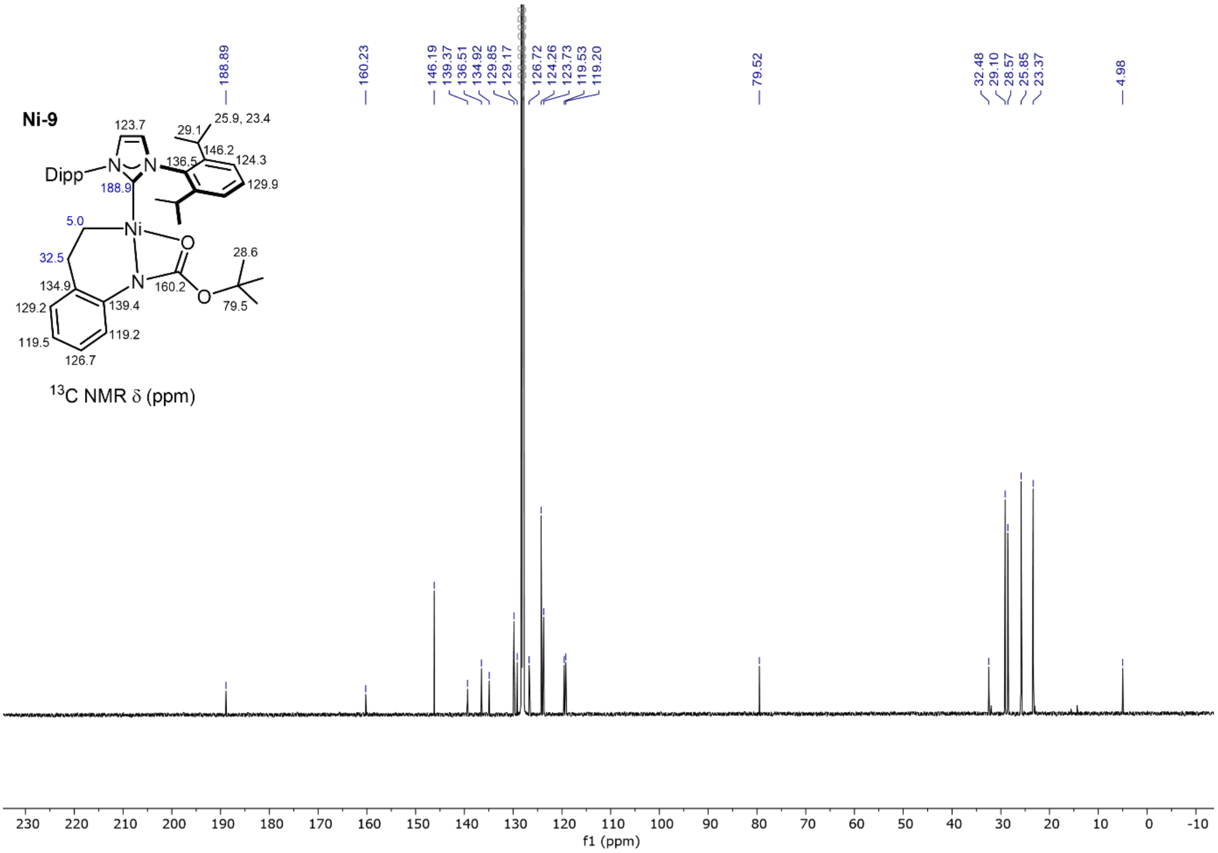

Supplementary Figure 78. ^{13}C{^{1}H} NMR spectrum of **Ni-9** (126 MHz, benzene-d_6, 23 °C).

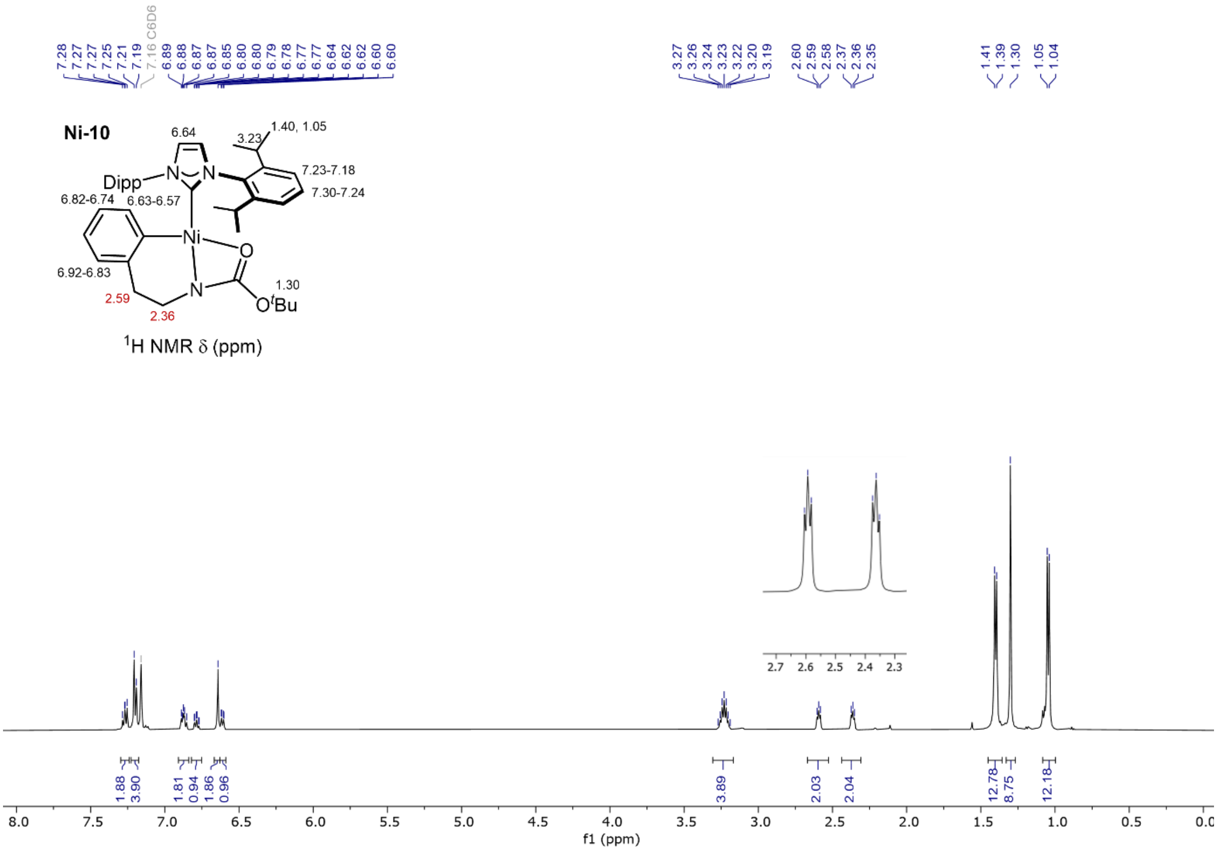

Supplementary Figure 79. ^{1}H NMR spectrum of **Ni-10** (500 MHz, benzene-d_6, 23 °C).

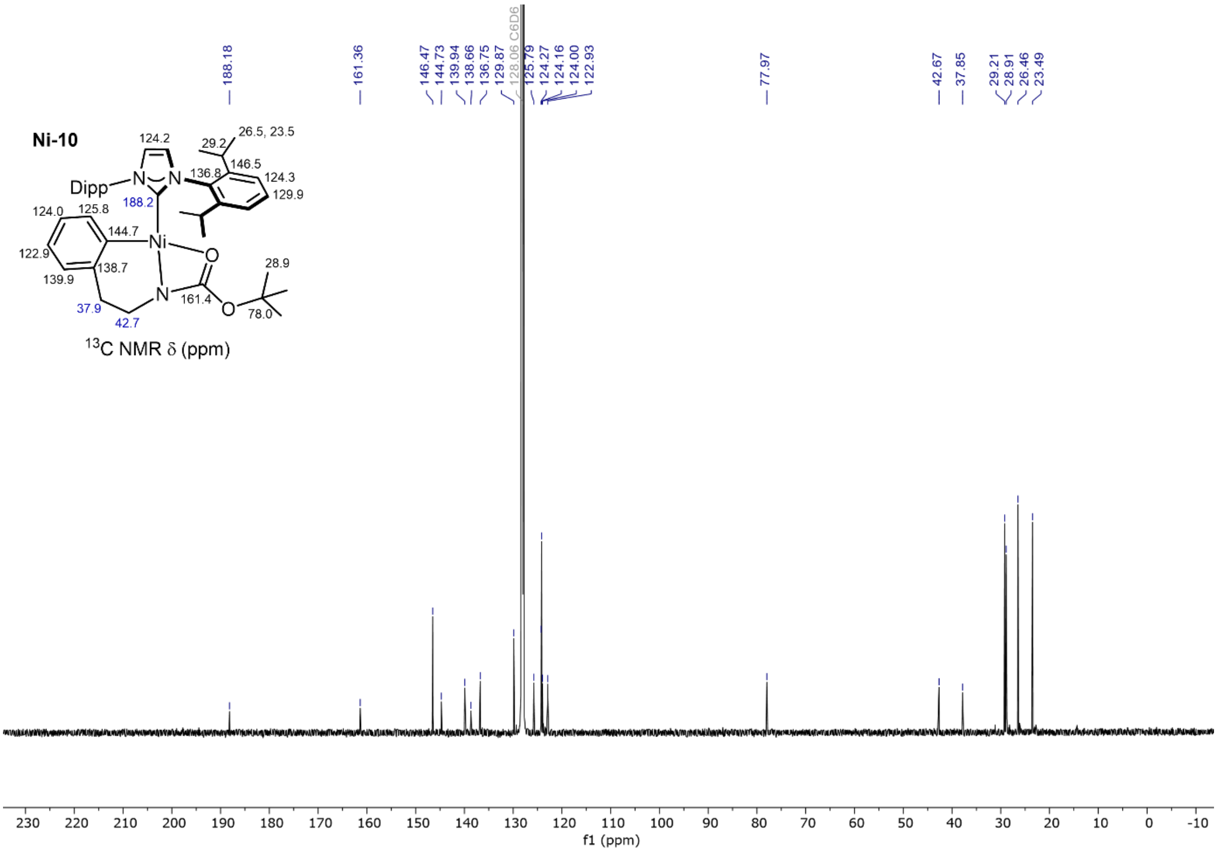

Supplementary Figure 80. ^{13}C{^{1}H} NMR spectrum of **Ni-10** (126 MHz, benzene-d_6, 23 °C).

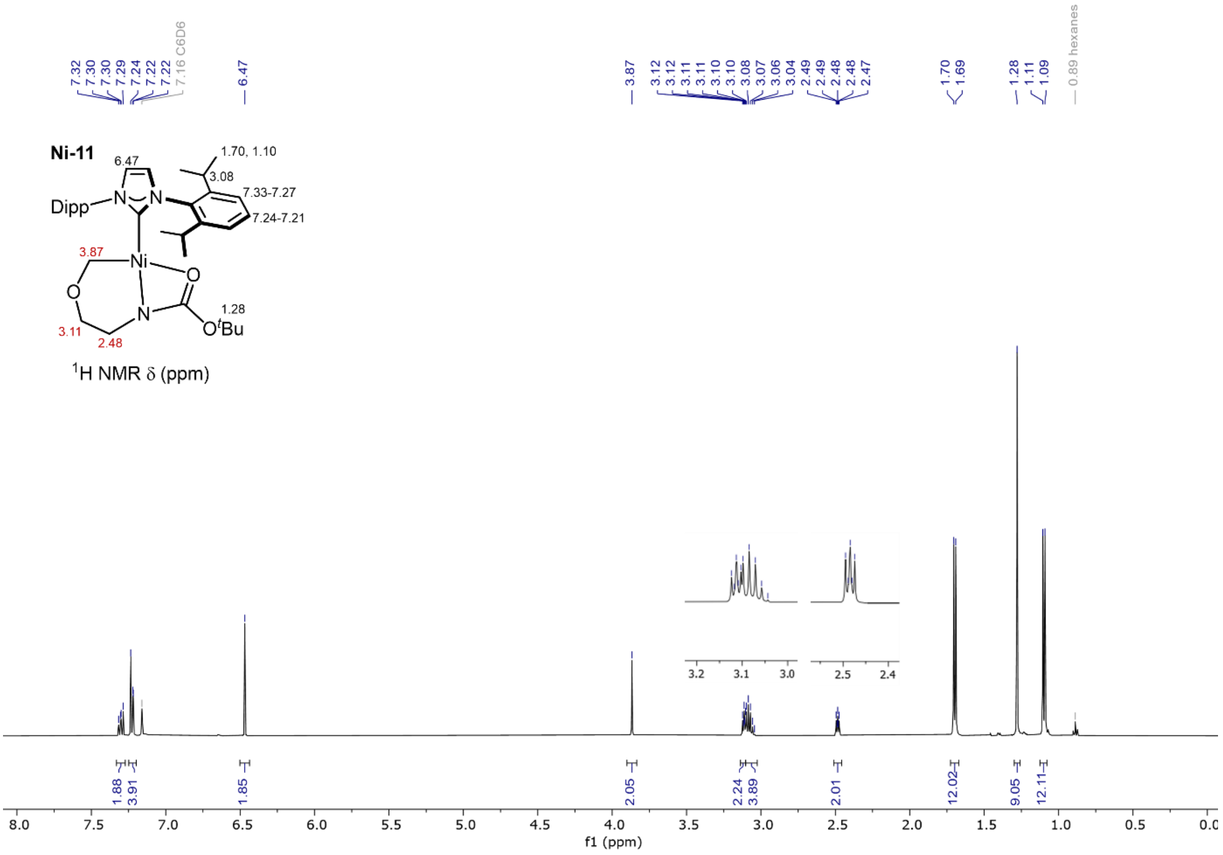

Supplementary Figure 81. ^{1}H NMR spectrum of **Ni-11** (500 MHz, benzene-d_6, 23 °C).

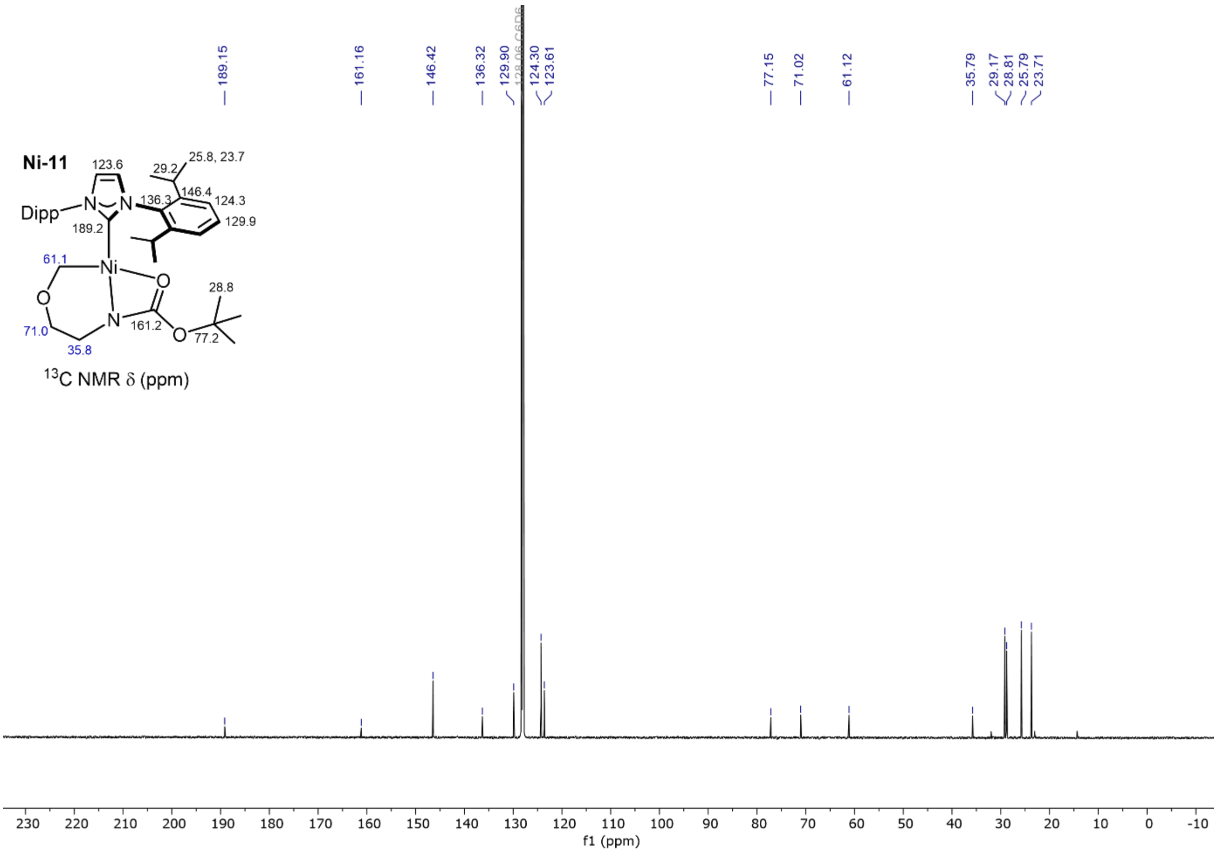

Supplementary Figure 82. ^{13}C{^{1}H} NMR spectrum of **Ni-11** (126 MHz, benzene-d_6, 23 °C).

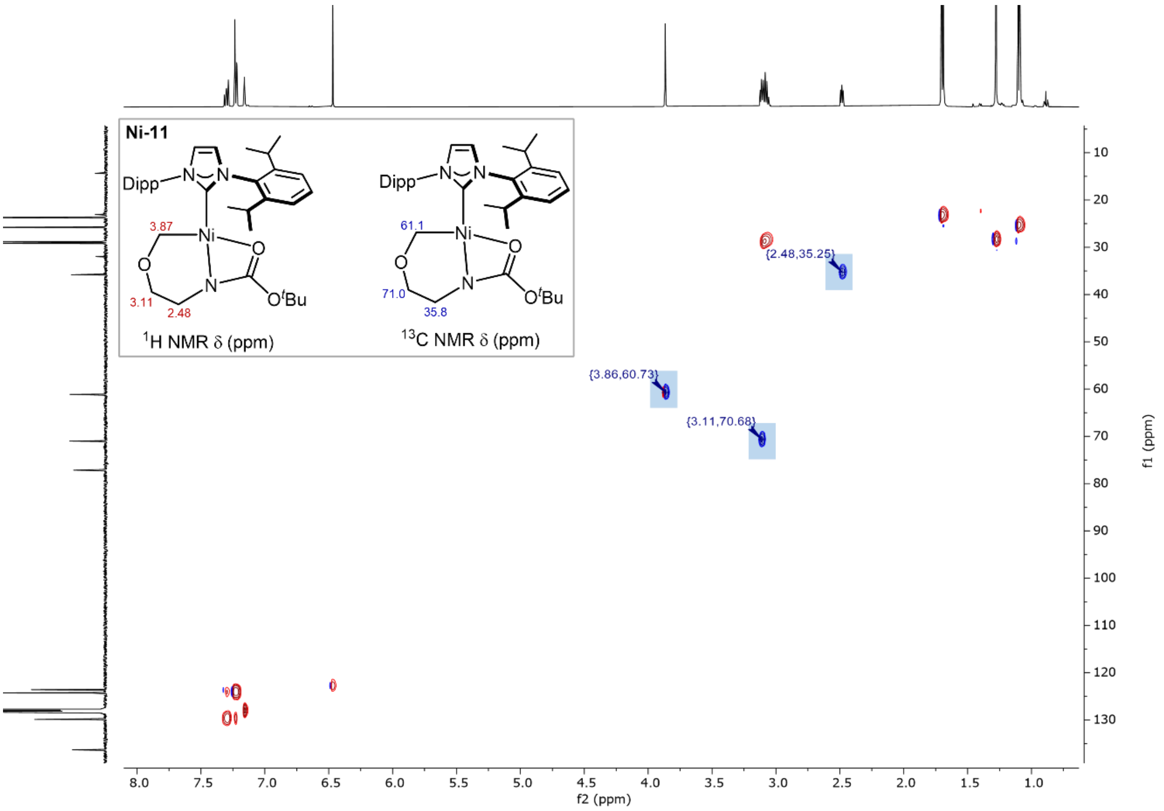

Supplementary Figure 83. Selected region of the HSQC spectrum of **Ni-11** displaying correlations between nickellacycle methylene protons and carbons (benzene-d_6, 23 °C).

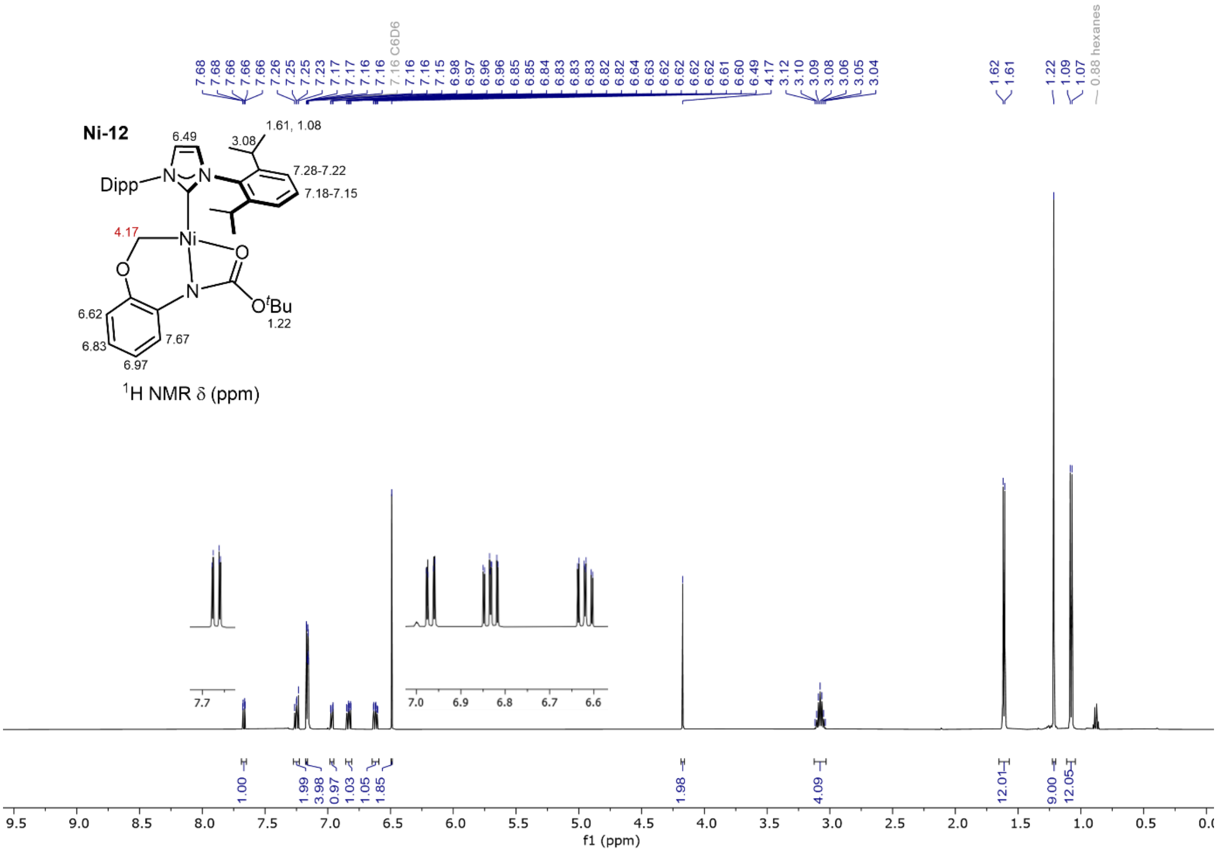

Supplementary Figure 84. ^{1}H NMR spectrum of **Ni-12** (500 MHz, benzene-d_6, 23 °C).

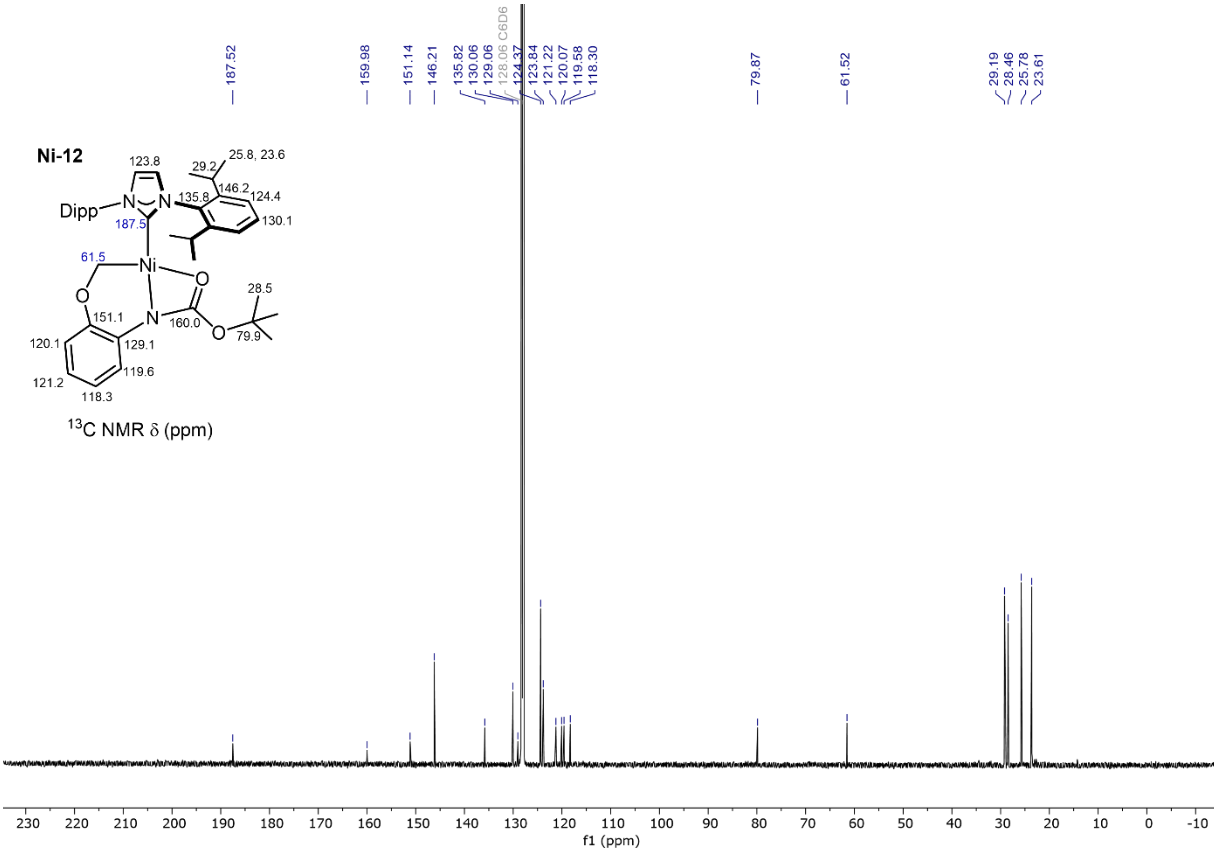

Supplementary Figure 85. ^{13}C{^{1}H} NMR spectrum of **Ni-12** (126 MHz, benzene-d_6, 23 °C).

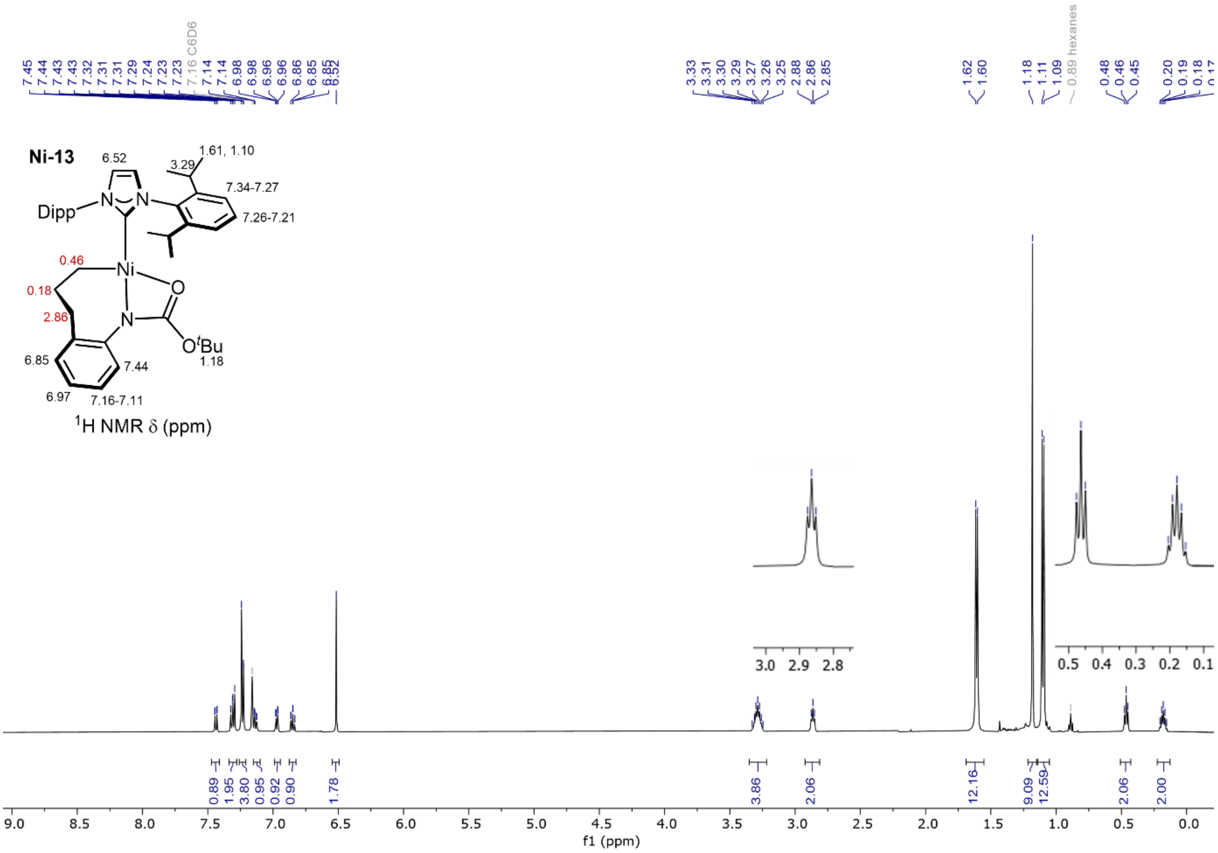

Supplementary Figure 86. ^{1}H NMR spectrum of **Ni-13** (500 MHz, benzene-d_6, 23 °C).

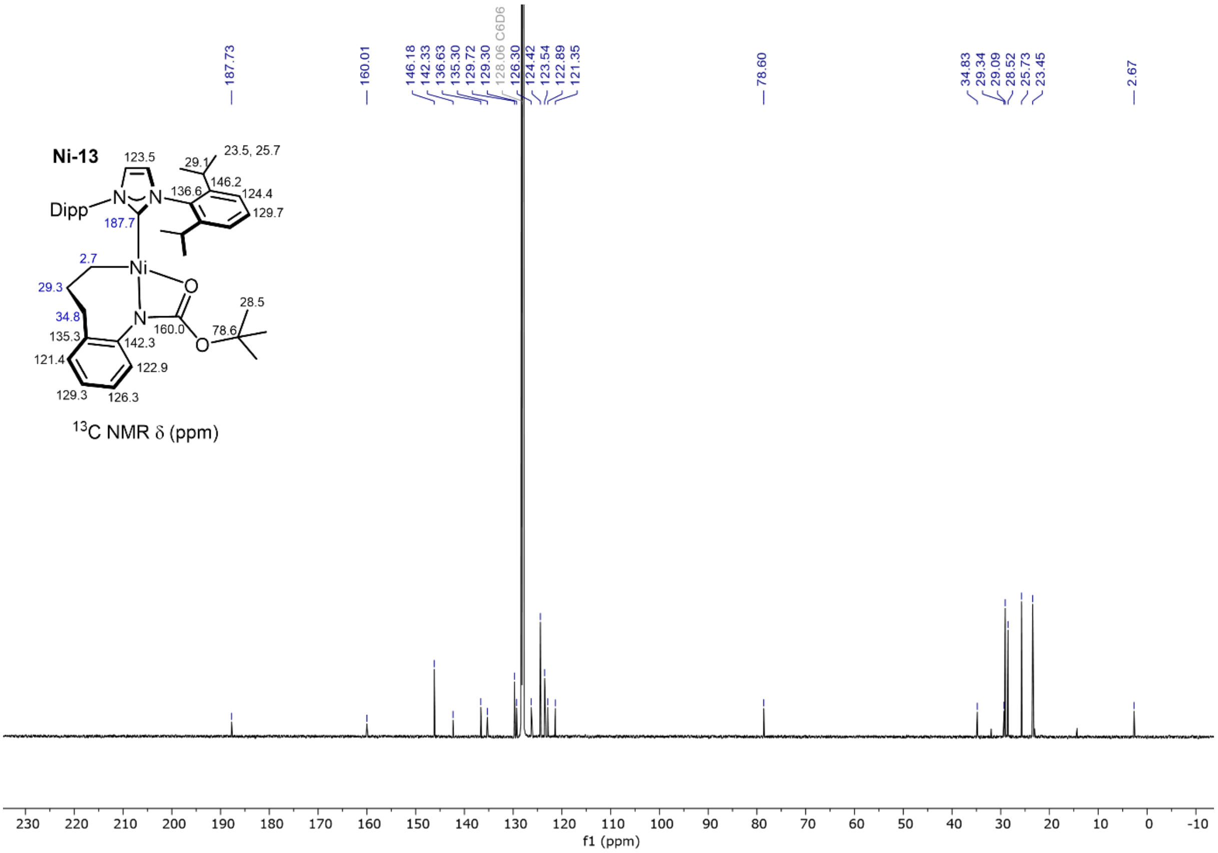

Supplementary Figure 87. ^{13}C{^{1}H} NMR spectrum of **Ni-13** (126 MHz, benzene-d_6, 23 °C).

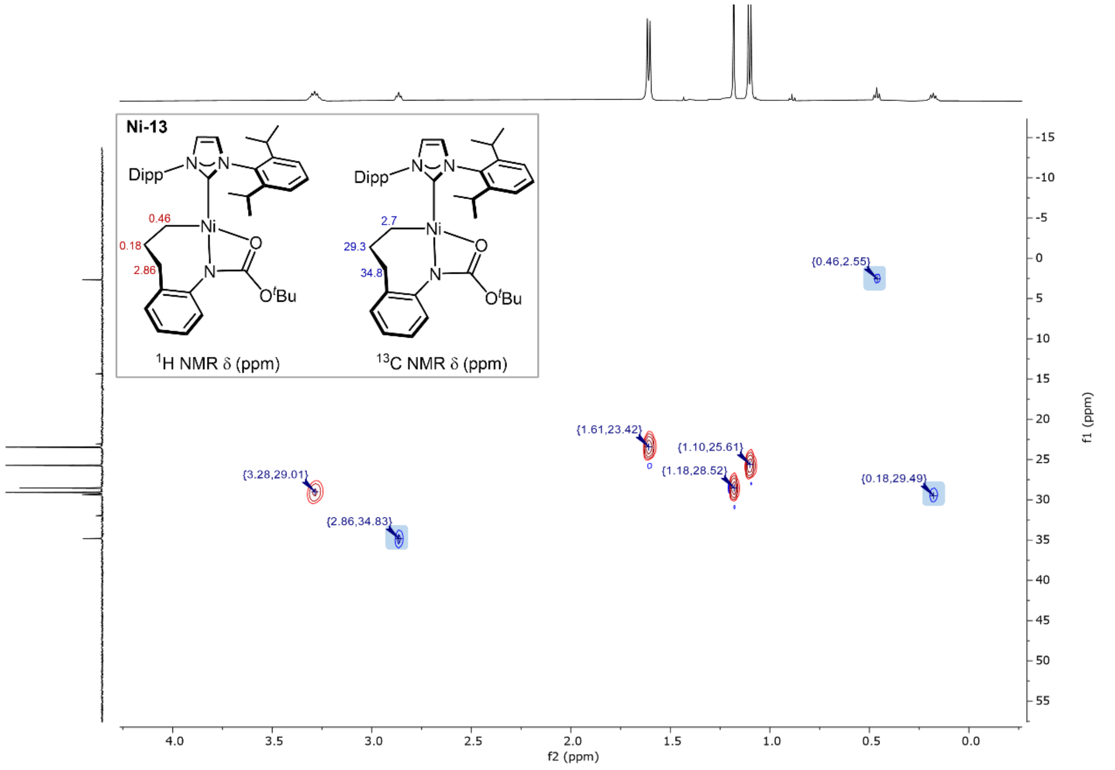

Supplementary Figure 88. Selected region of the HSQC spectrum of **Ni-13** displaying correlations between nickellacycle methylene protons and carbons (benzene-d_6, 23 °C).

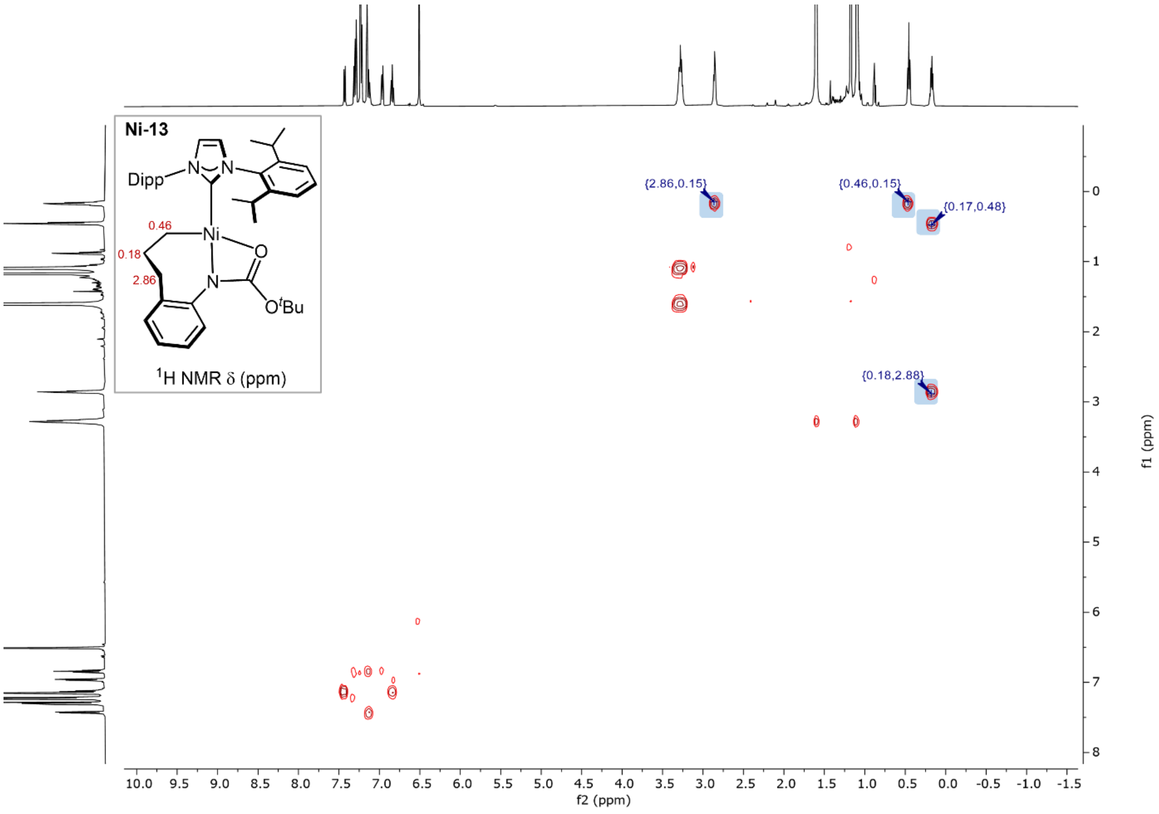

Supplementary Figure 89. Selected region of the COSY spectrum of **Ni-13** displaying correlations between nickellacycle methylene protons (benzene-d_6, 23 °C).

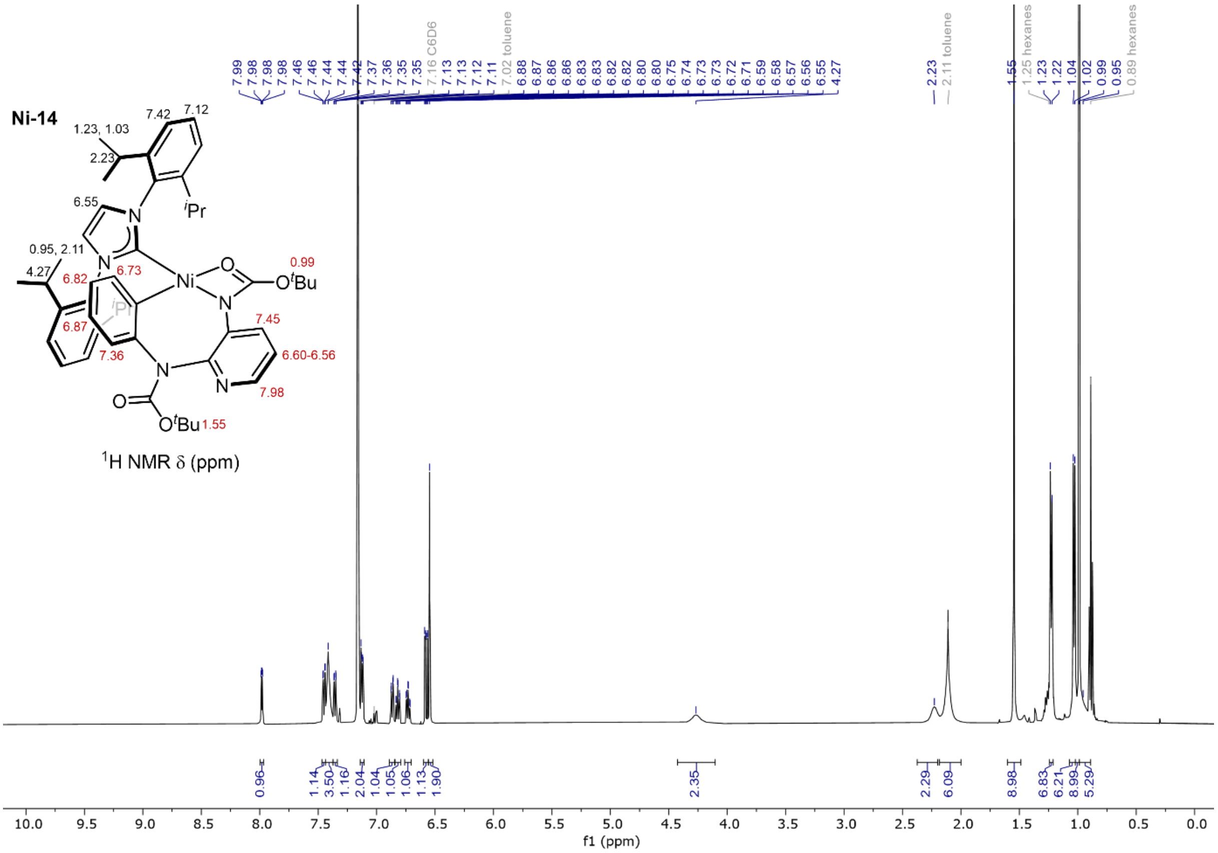

Supplementary Figure 90. ^{1}H NMR spectrum of **Ni-14** (500 MHz, benzene-d_6, 23 °C).

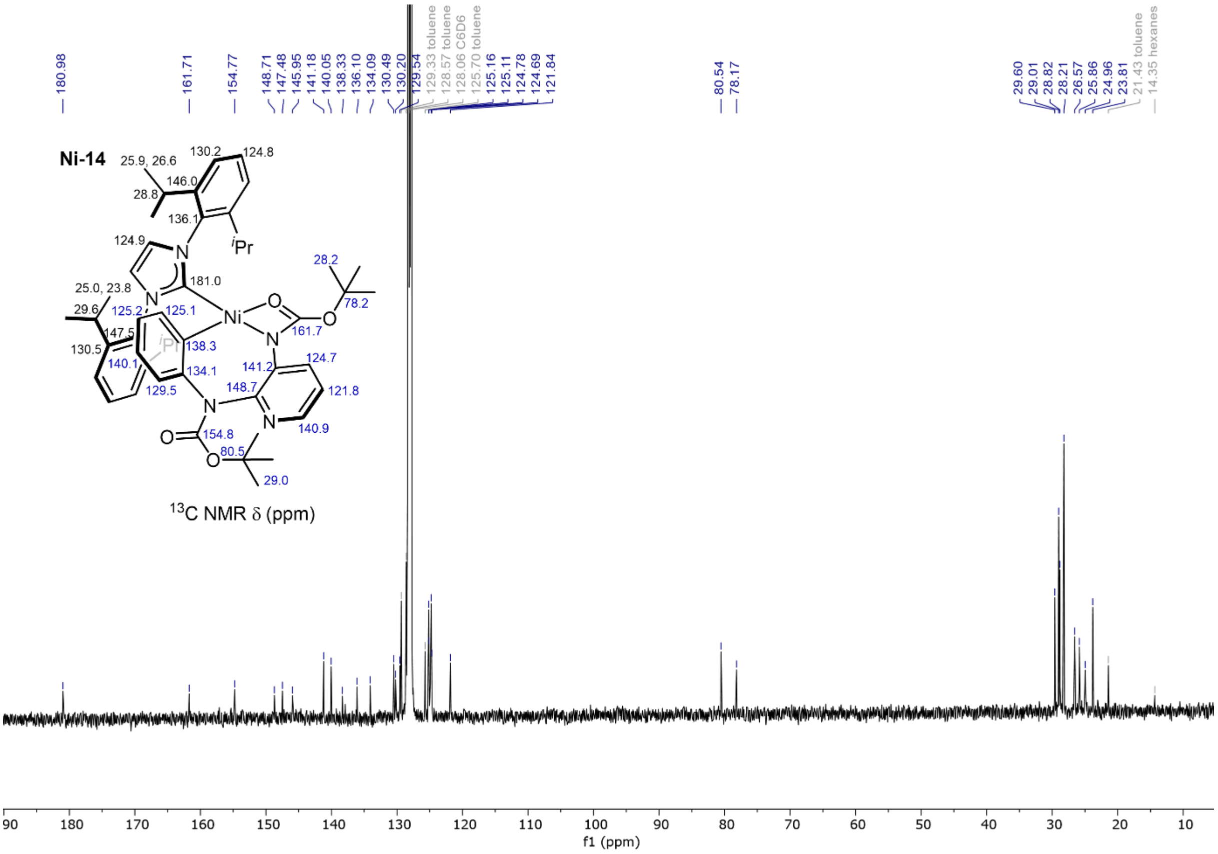

Supplementary Figure 91. ^{13}C{^{1}H} NMR spectrum of **Ni-14** (126 MHz, benzene-d_6, 23 °C).

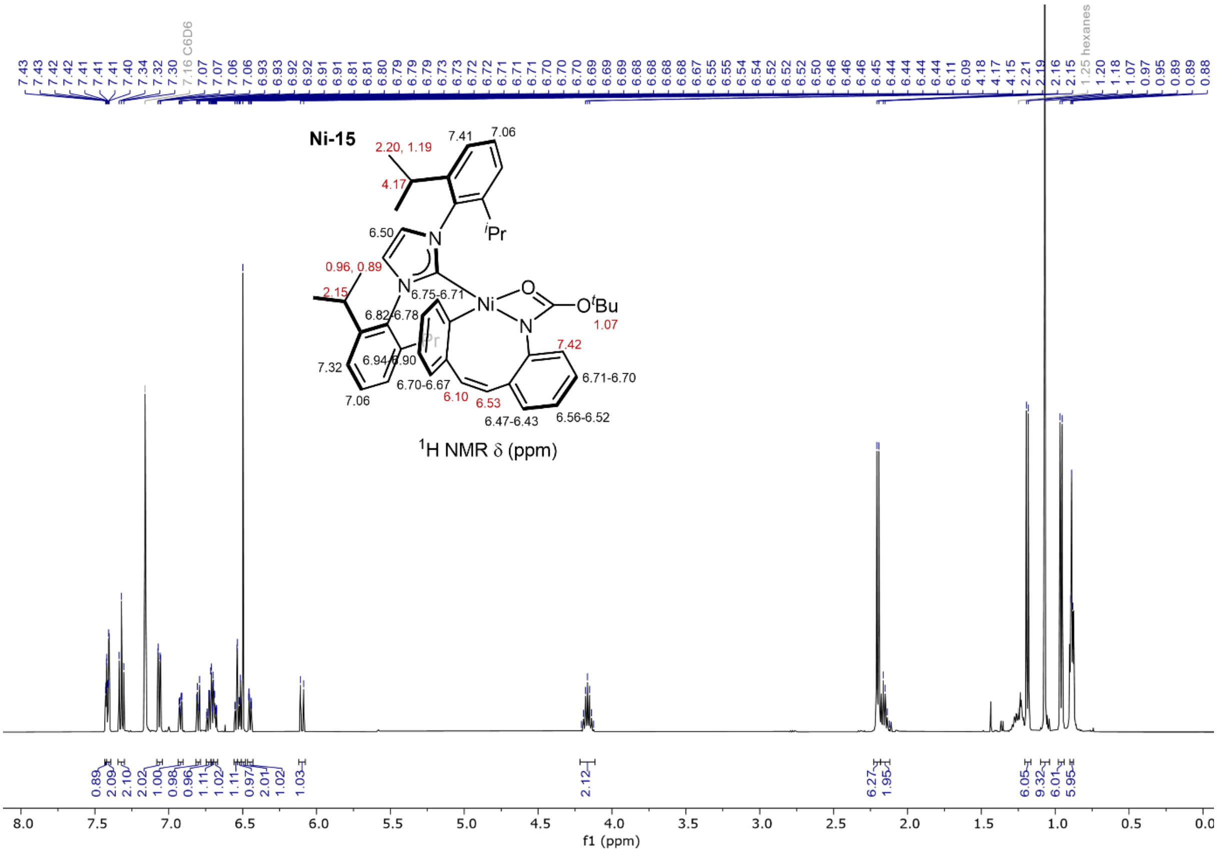

Supplementary Figure 92. ^{1}H NMR spectrum of **Ni-15** (500 MHz, benzene-d_6, 23 °C).

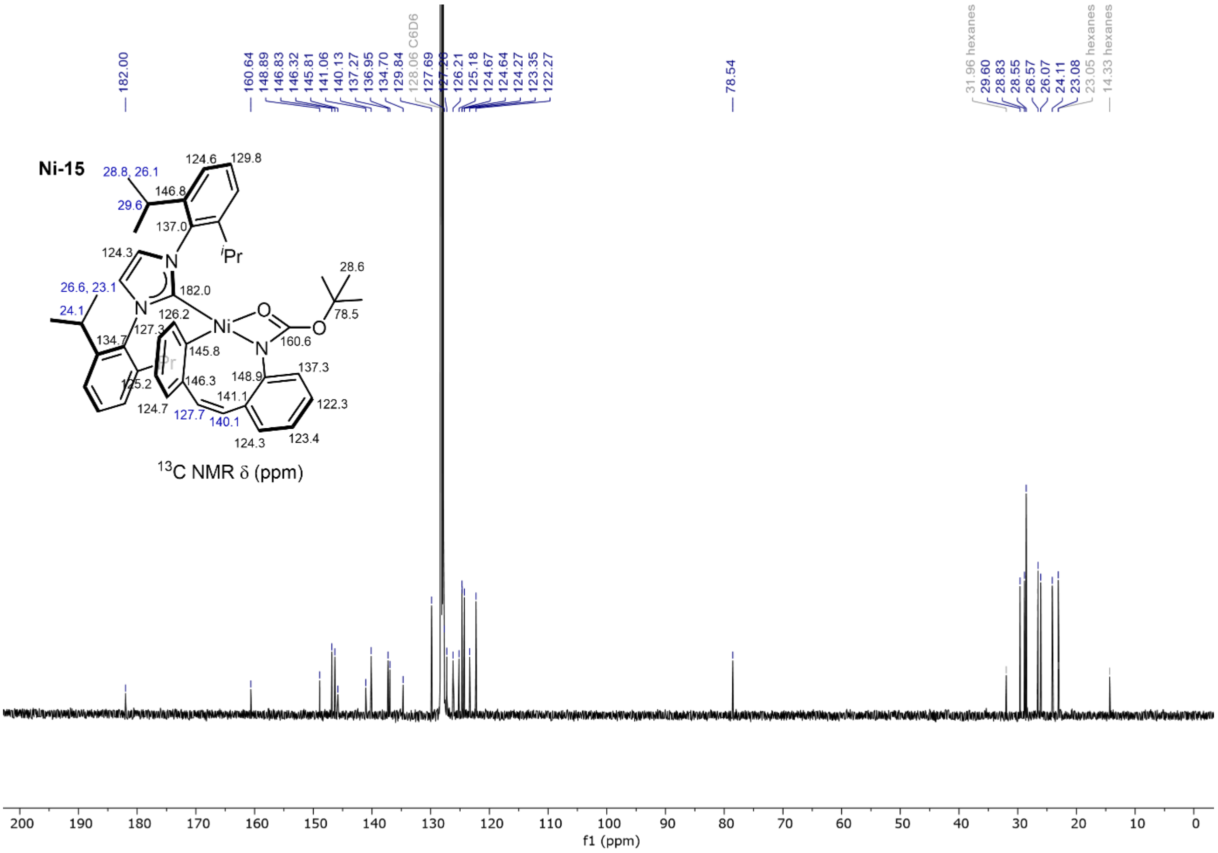

Supplementary Figure 93. ^{13}C{^{1}H} NMR spectrum of **Ni-15** (126 MHz, benzene-d_6, 23 °C).

8. NMR spectra of organic compounds

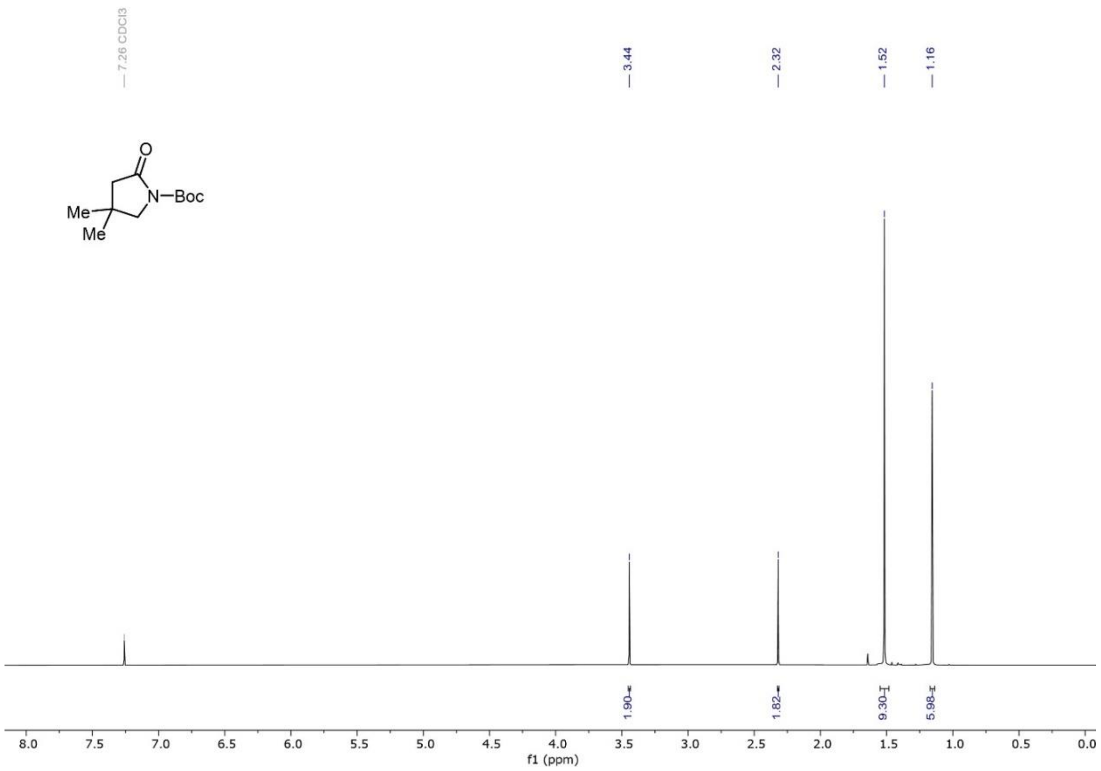

Supplementary Figure 94. ^{1}H NMR spectrum of **6** (500 MHz, chloroform-*d*, 23 °C).

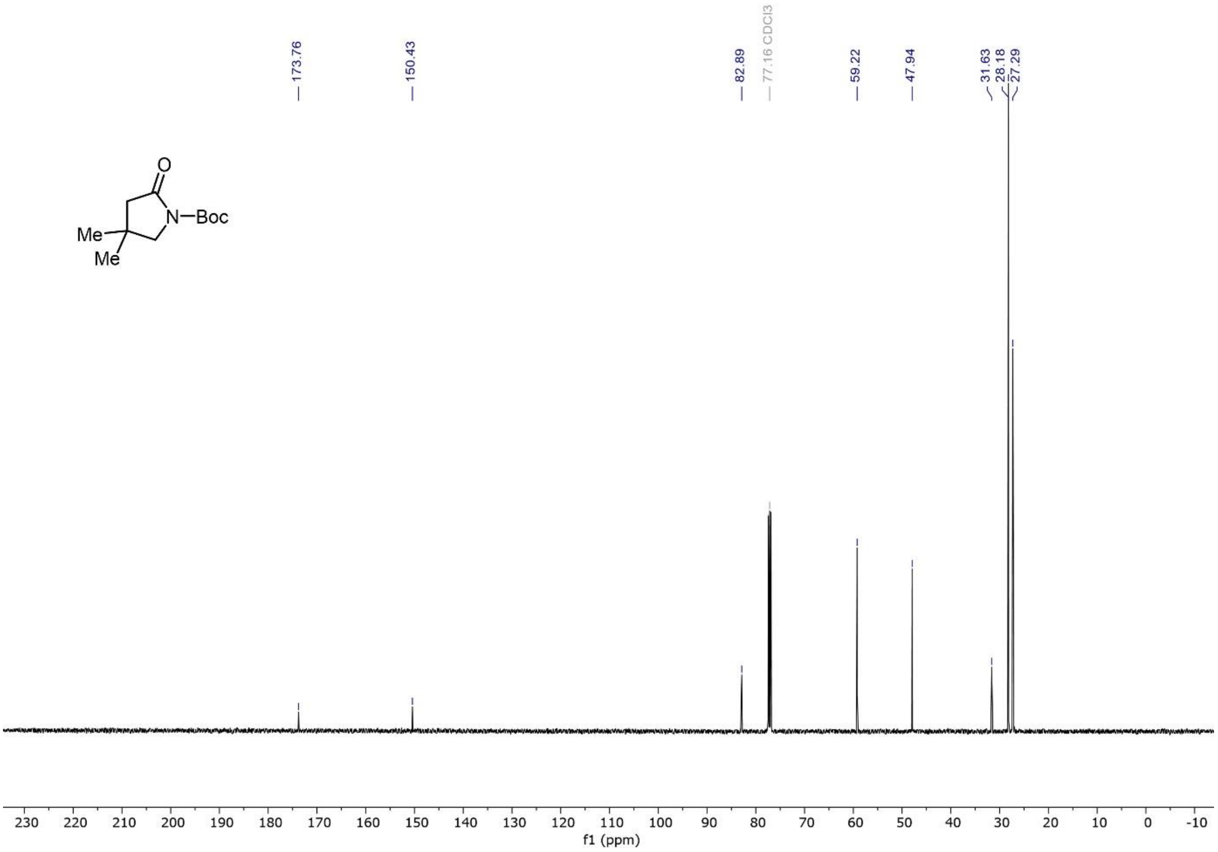

Supplementary Figure 95. $^{13}C\{^{1}H\}$ NMR spectrum of **6** (126 MHz, chloroform-*d*, 23 °C).

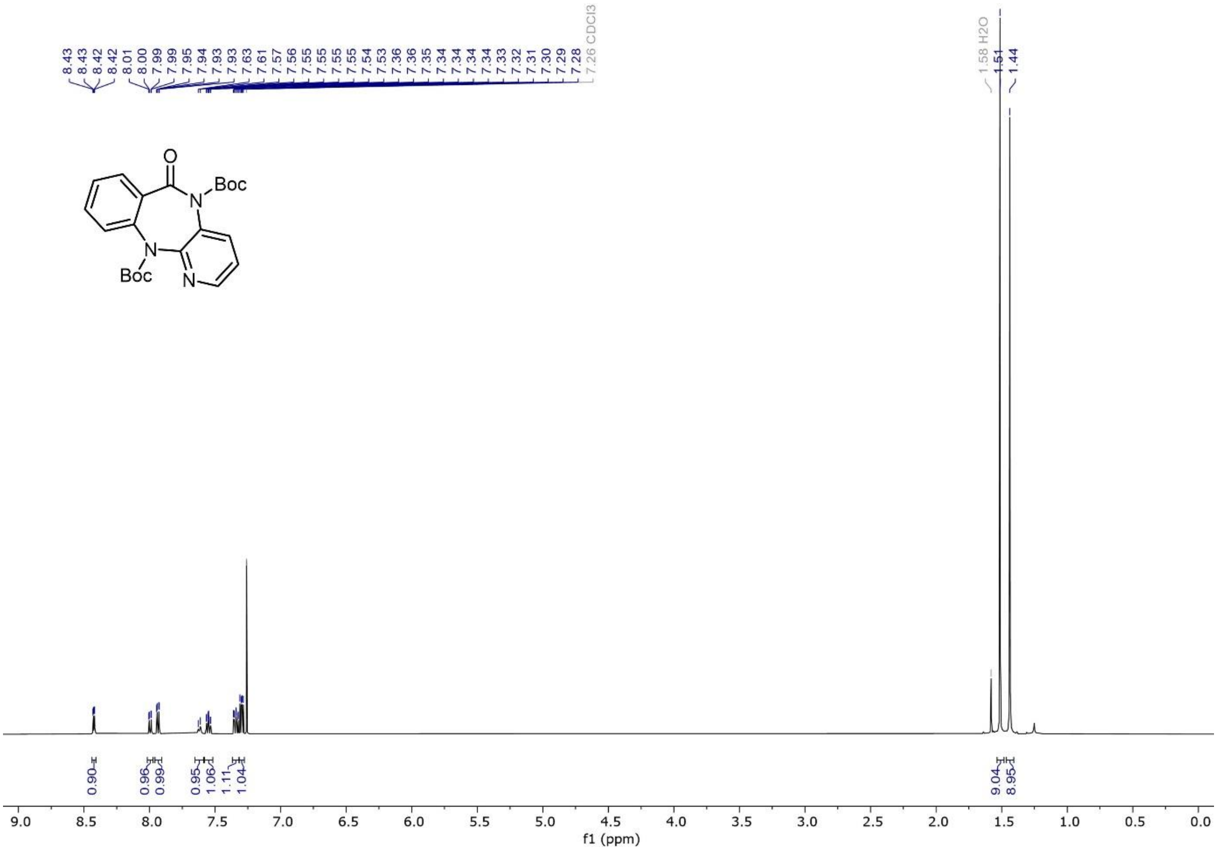

Supplementary Figure 96. ^{1}H NMR spectrum of **14** (500 MHz, chloroform-*d*, 23 °C).

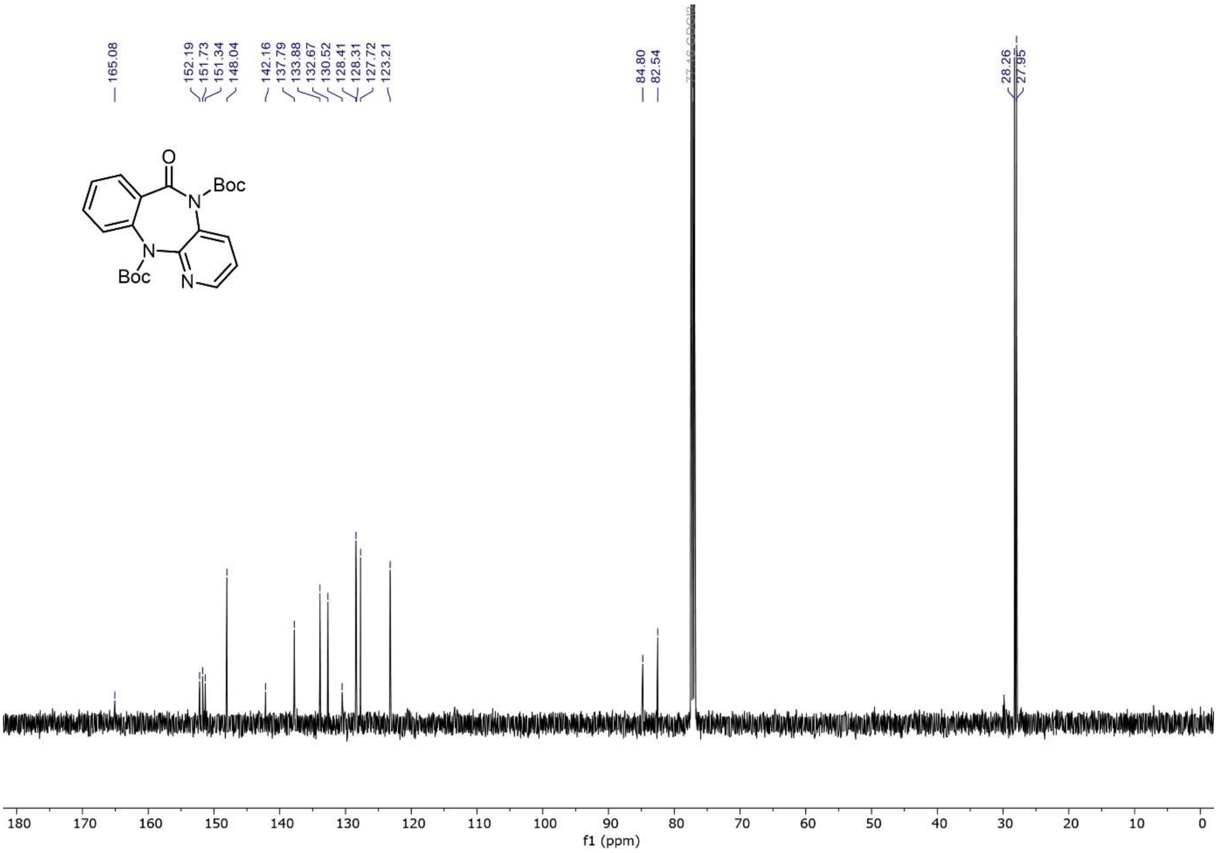

Supplementary Figure 97. $^{13}C\{^{1}H\}$ NMR spectrum of **14** (126 MHz, chloroform-*d*, 23 °C).

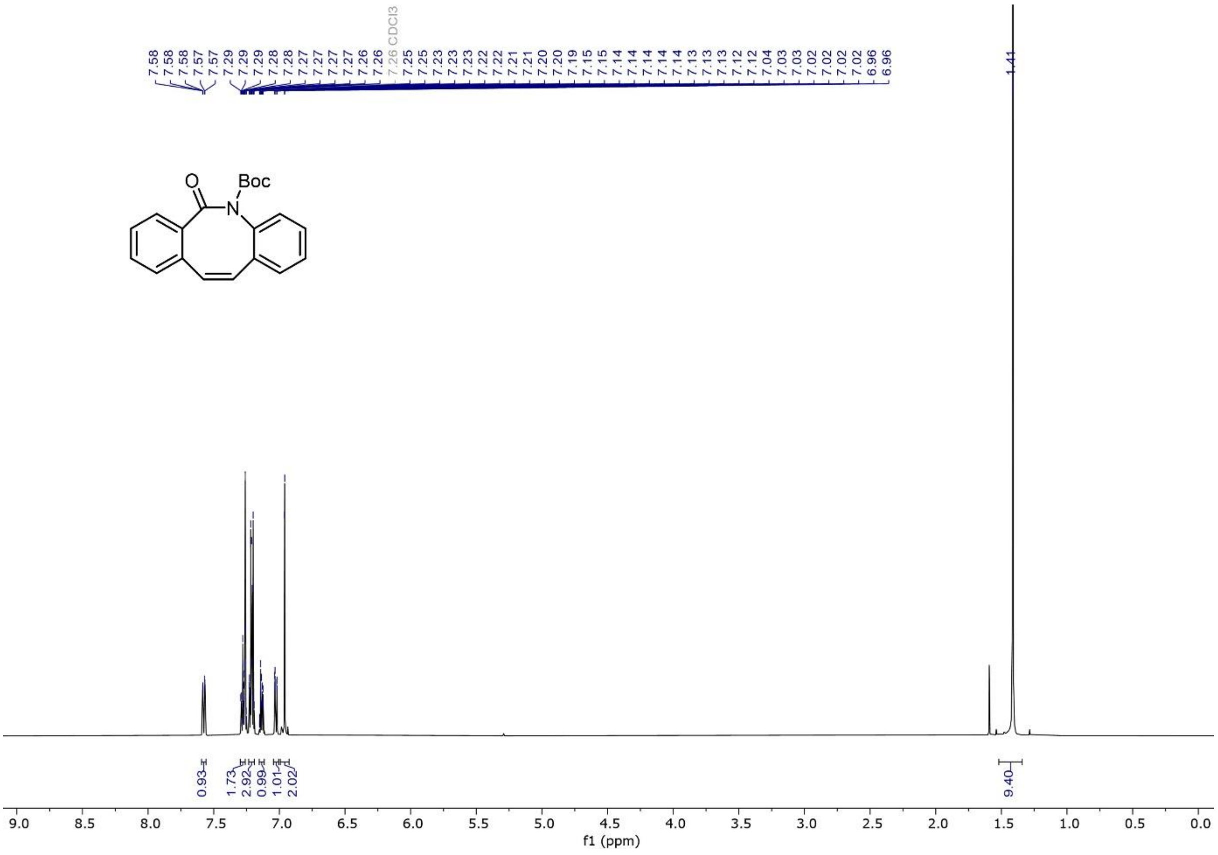

Supplementary Figure 98. ^{1}H NMR spectrum of **15** (500 MHz, chloroform-*d*, 23 °C).

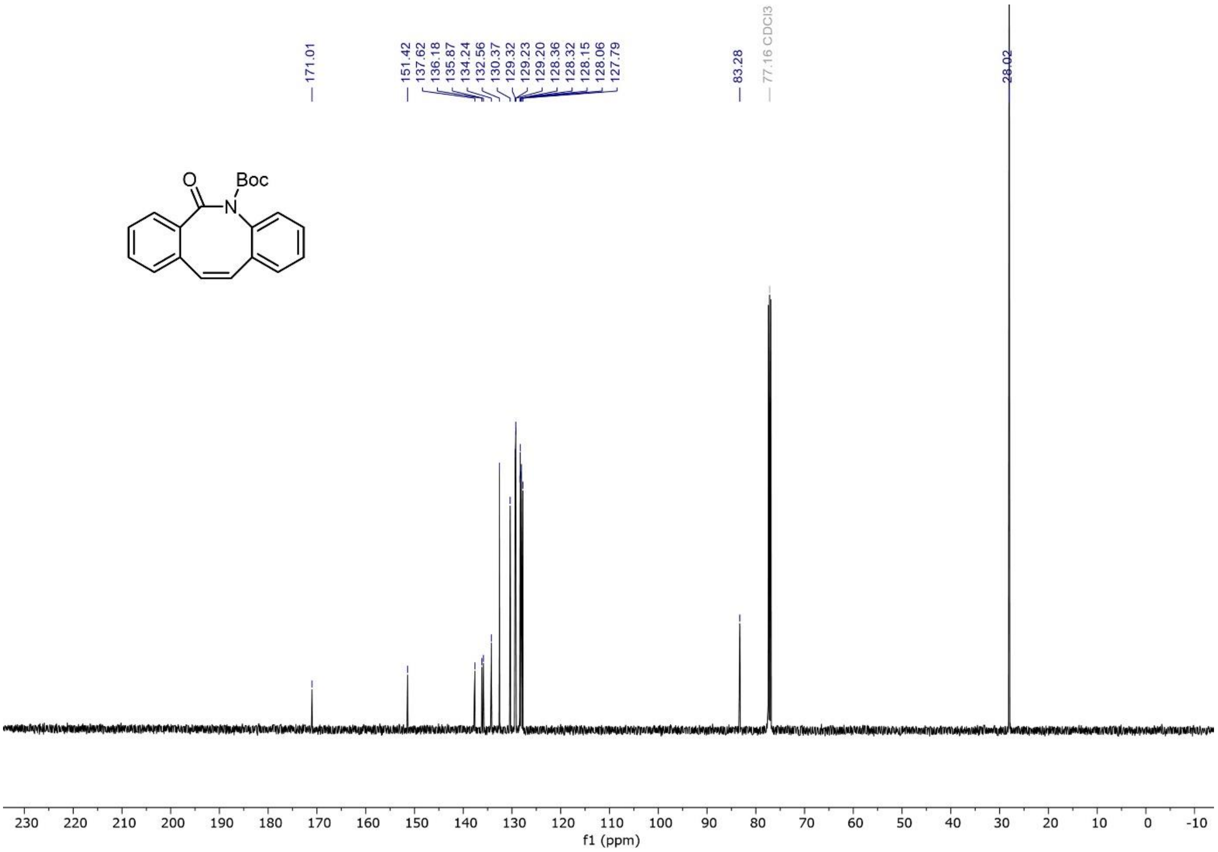

Supplementary Figure 99. ^{13}C{^{1}H} NMR spectrum of **15** (126 MHz, chloroform-*d*, 23 °C).

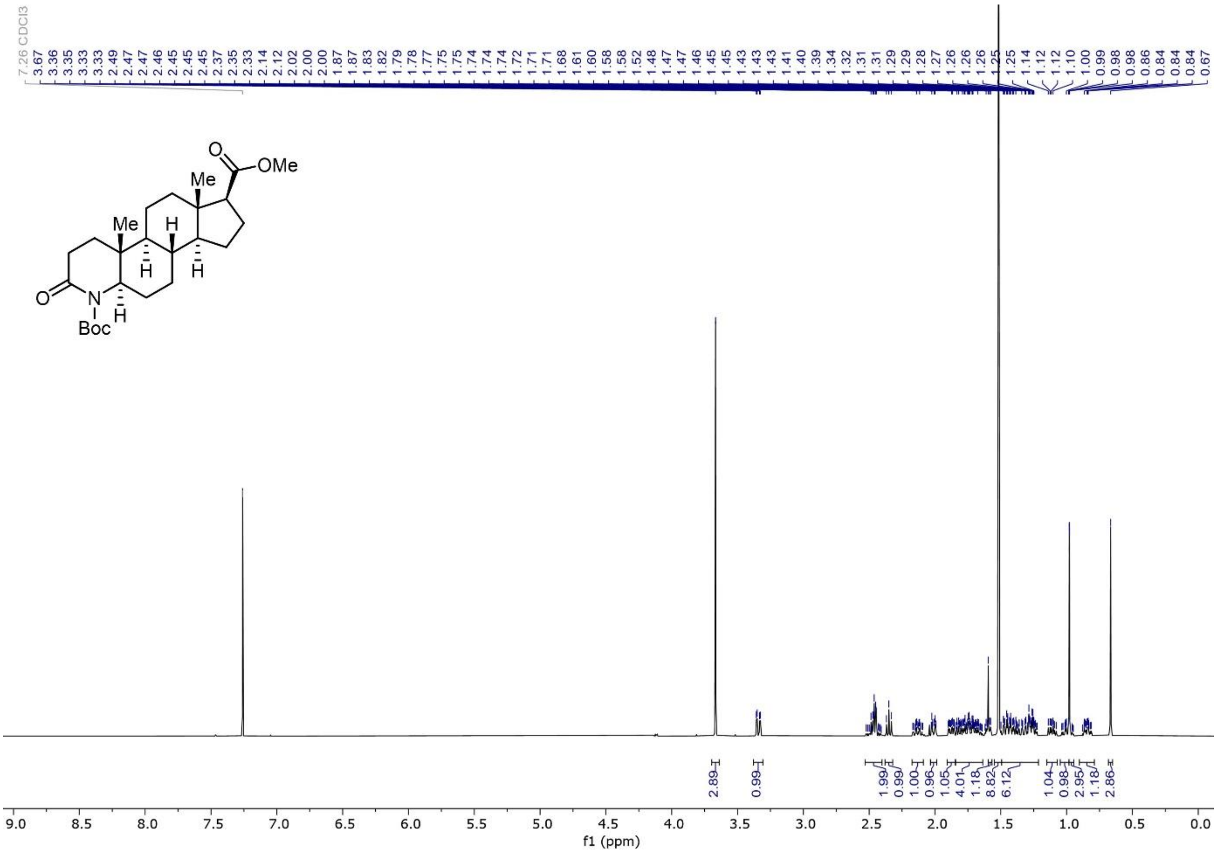

Supplementary Figure 100. ^{1}H NMR spectrum of **19** (500 MHz, chloroform-*d*, 23 °C).

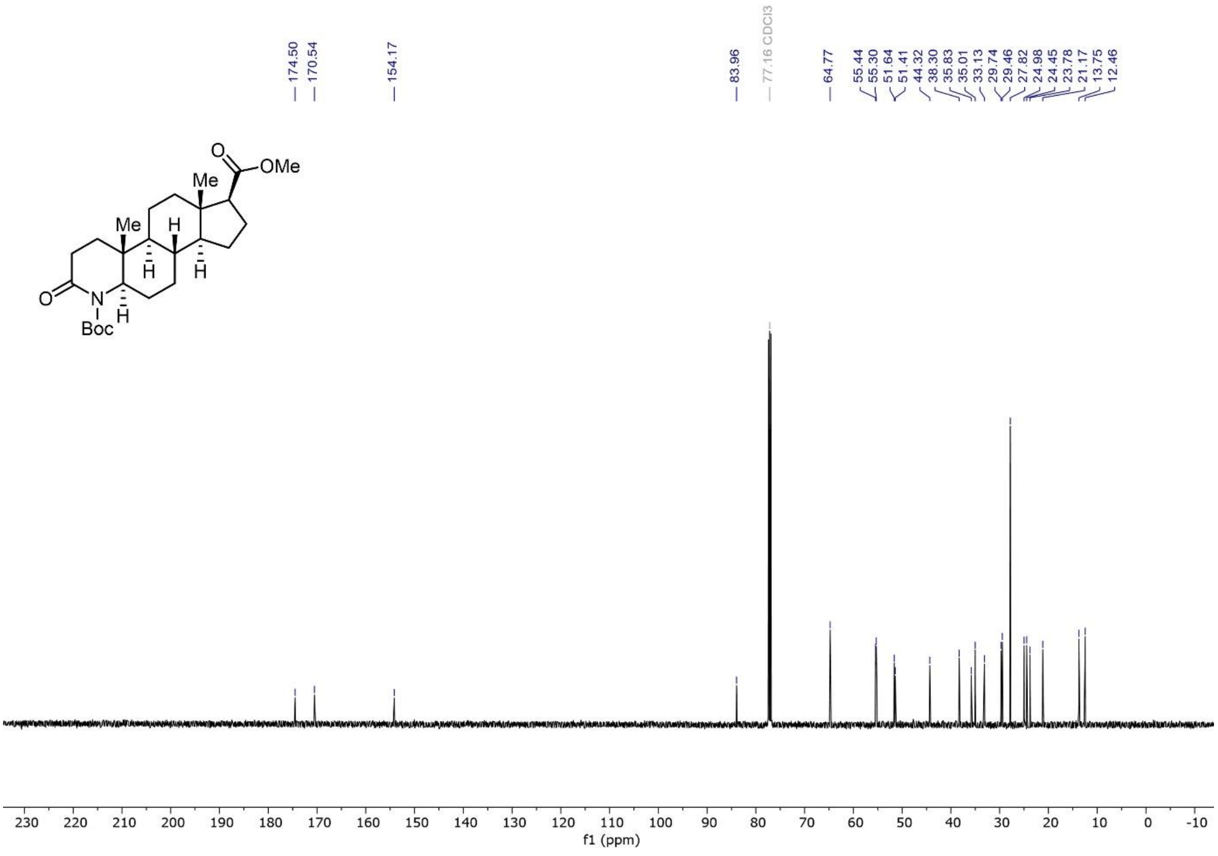

Supplementary Figure 101. ^{13}C{^{1}H} NMR spectrum of **19** (126 MHz, chloroform-*d*, 23 °C).

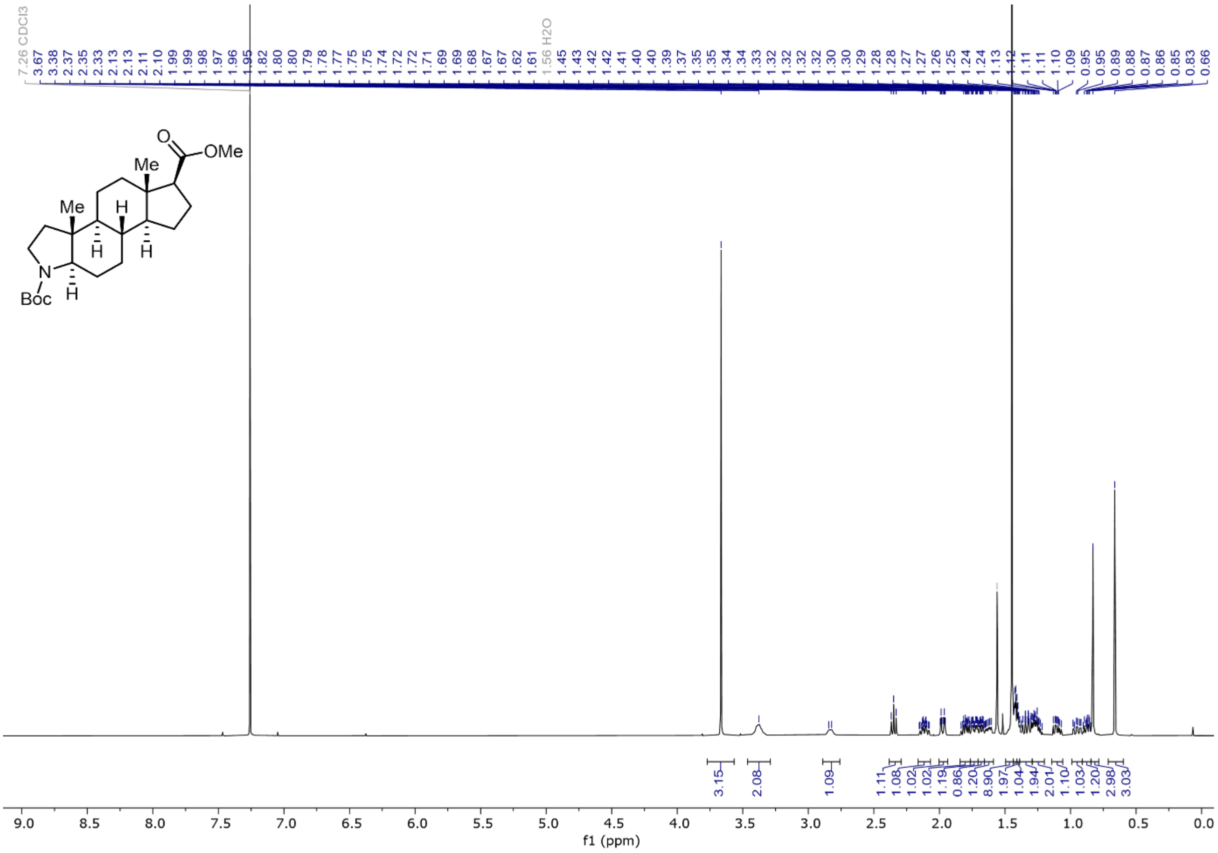

Supplementary Figure 102. ^{1}H NMR spectrum of **19a** (500 MHz, chloroform-*d*, 23 °C).

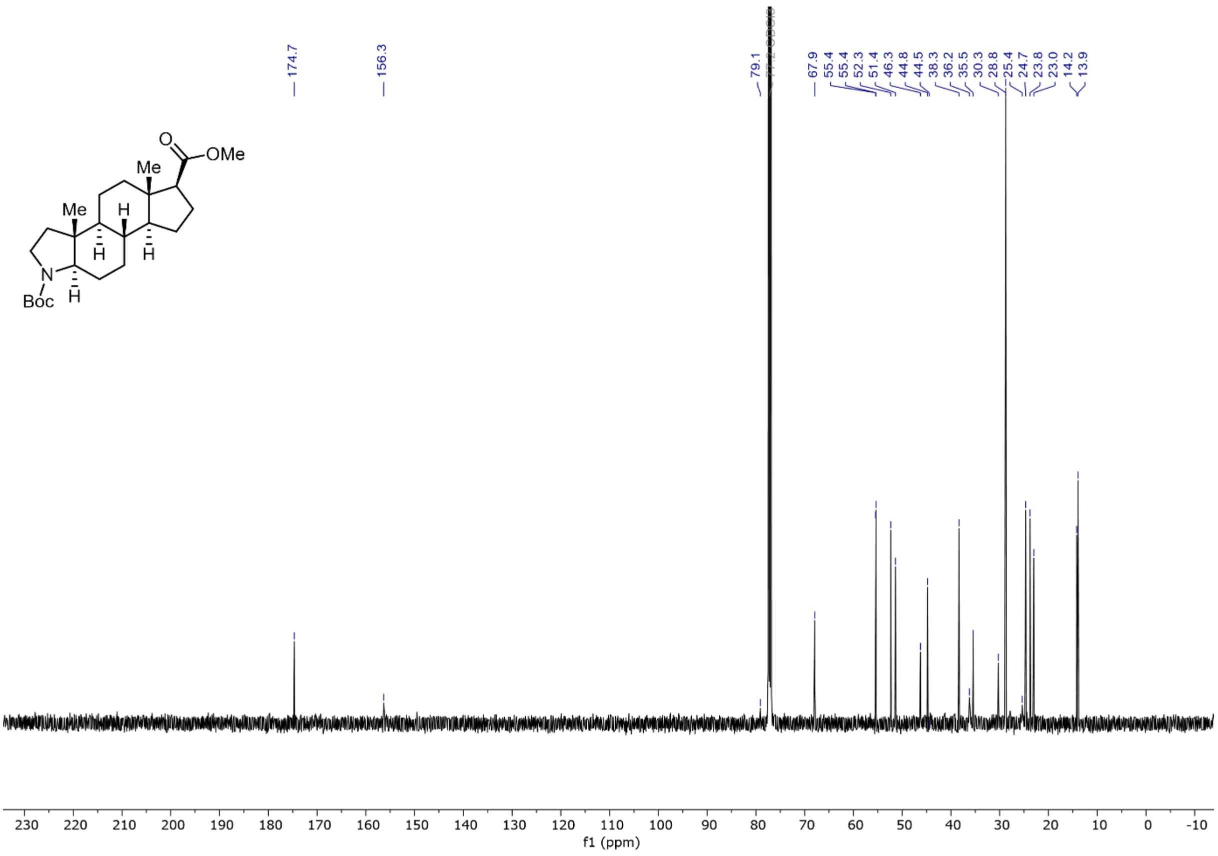

Supplementary Figure 103. ^{13}C{^{1}H} NMR spectrum of **19a** (126 MHz, chloroform-*d*, 23 °C).

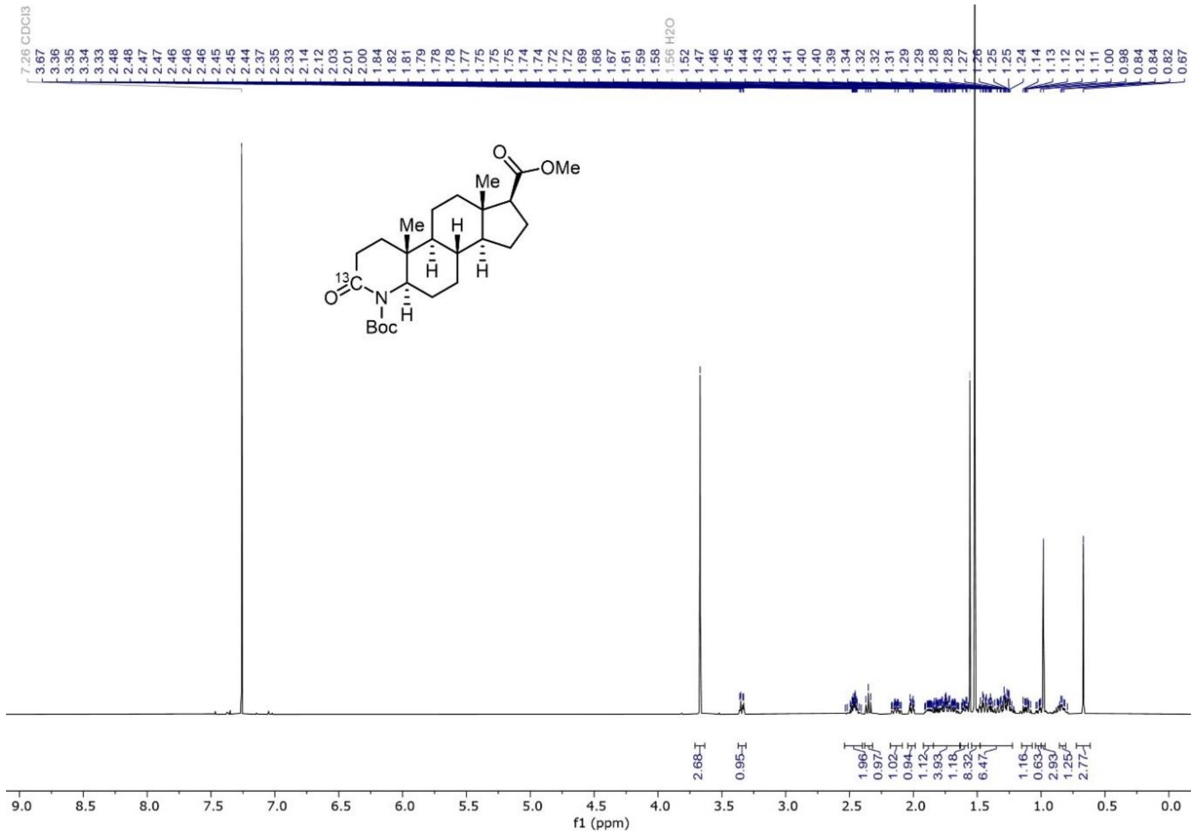

Supplementary Figure 104. ^{1}H NMR spectrum of **^{13}C-19** (500 MHz, chloroform-*d*, 23 °C).

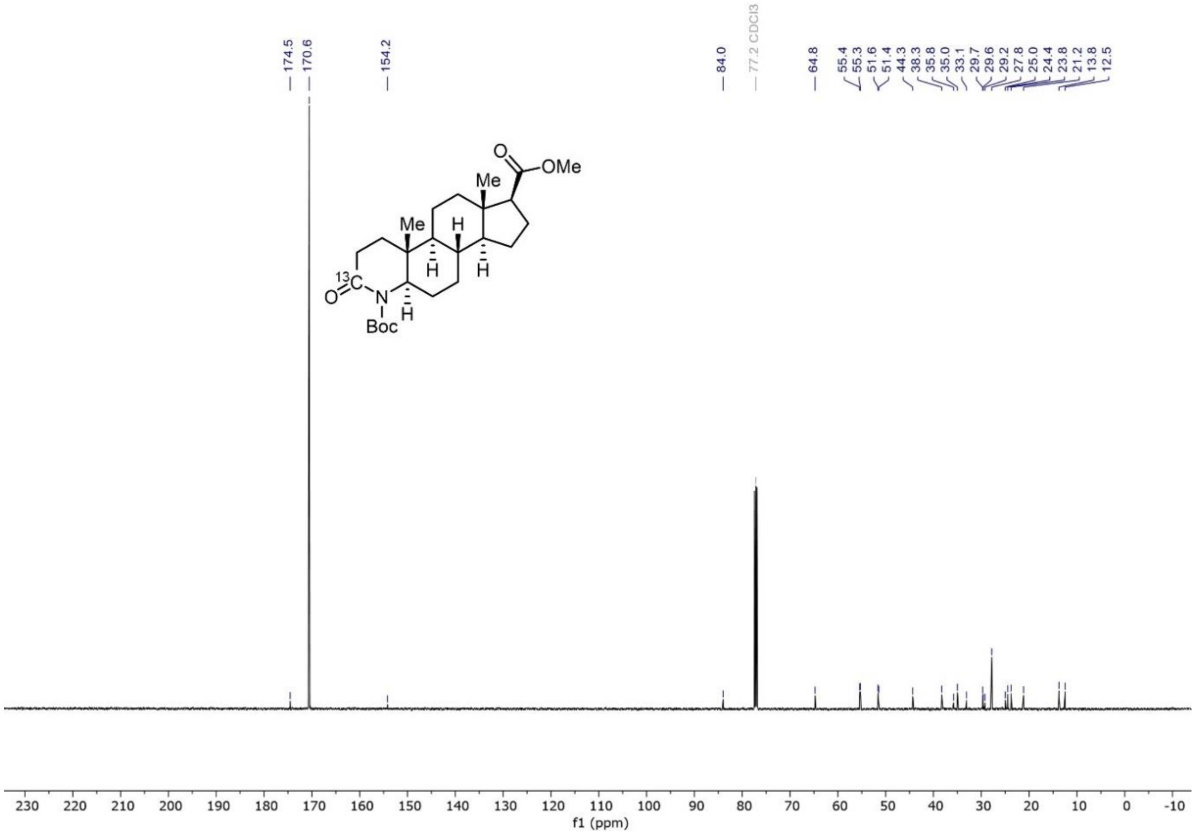

Supplementary Figure 105. ^{13}C{^{1}H} NMR spectrum of **^{13}C-19** (80±1% ^{13}C-labeled, 126 MHz, chloroform-*d*, 23 °C).

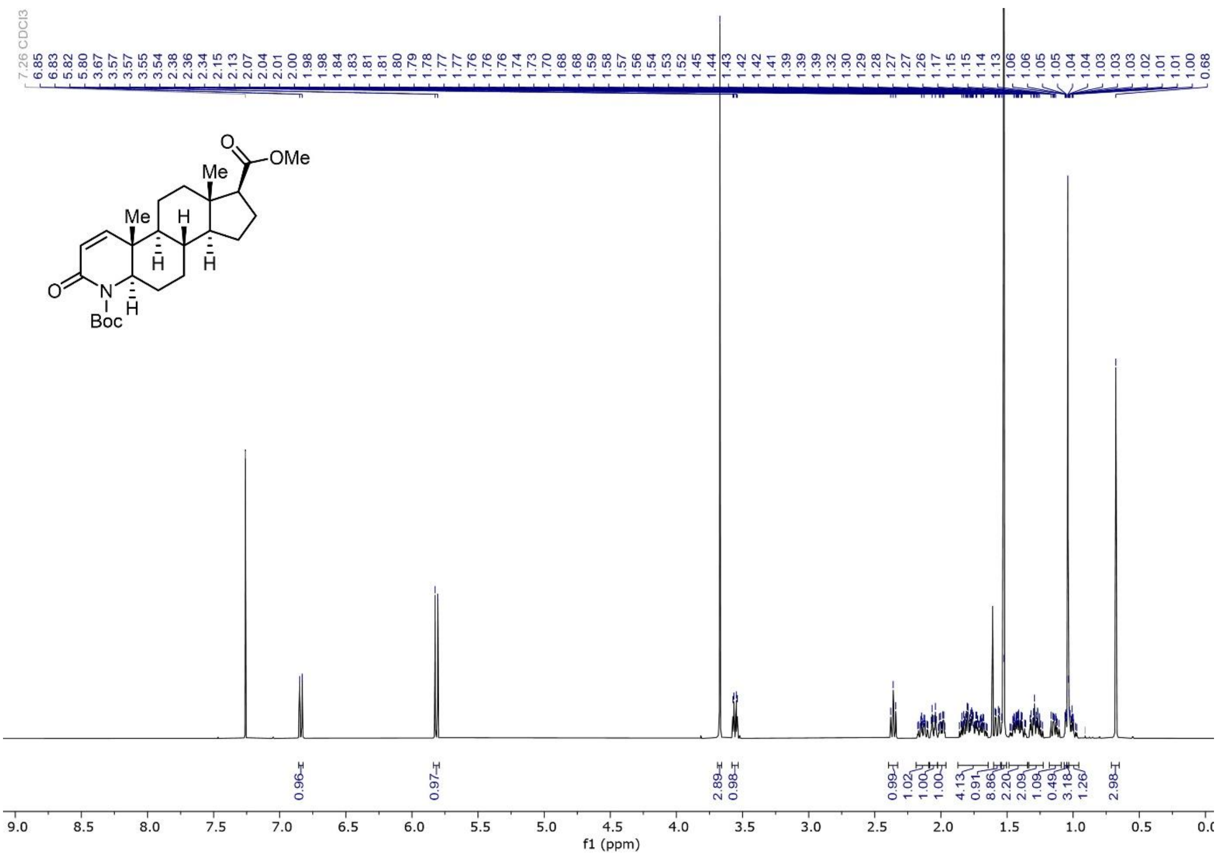

Supplementary Figure 106. ^{1}H NMR spectrum of **20** (500 MHz, chloroform-*d*, 23 °C).

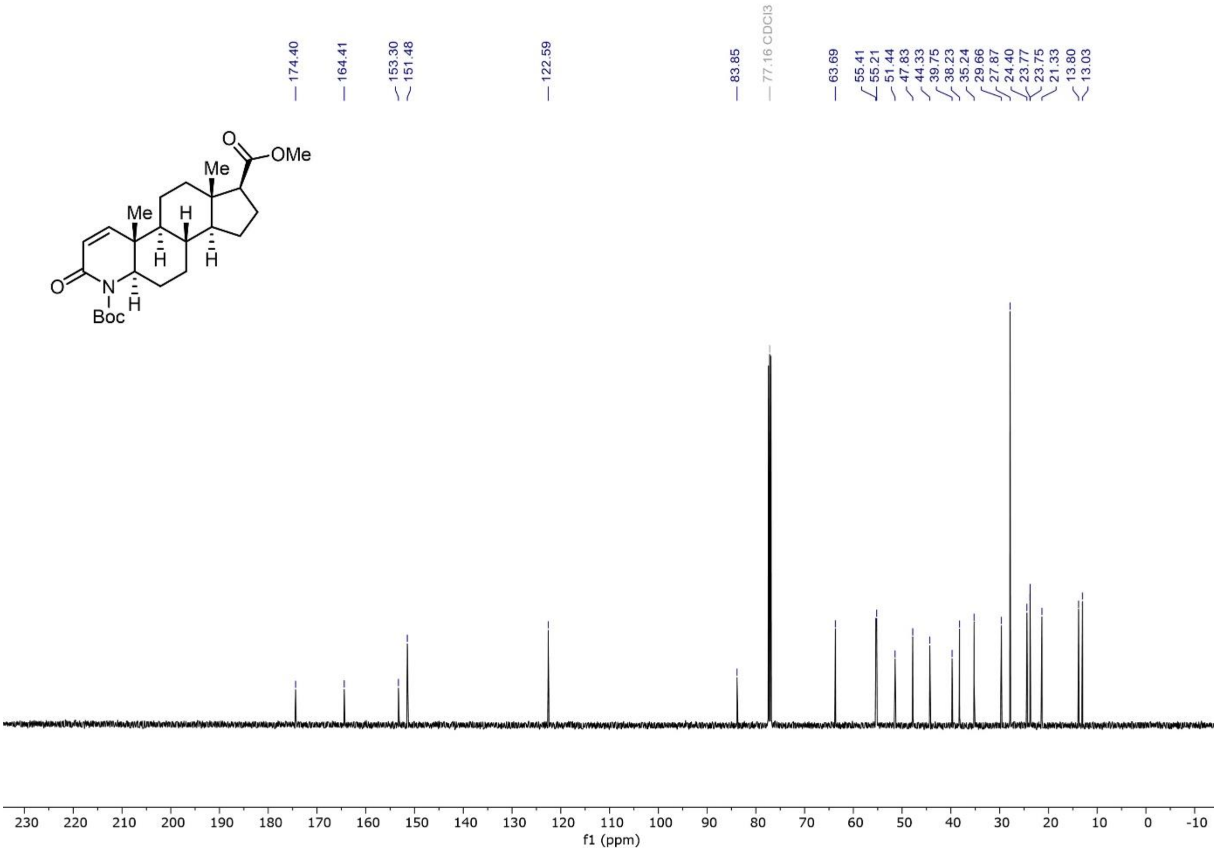

Supplementary Figure 107. ^{13}C{^{1}H} NMR spectrum of **20** (126 MHz, chloroform-*d*, 23 °C).

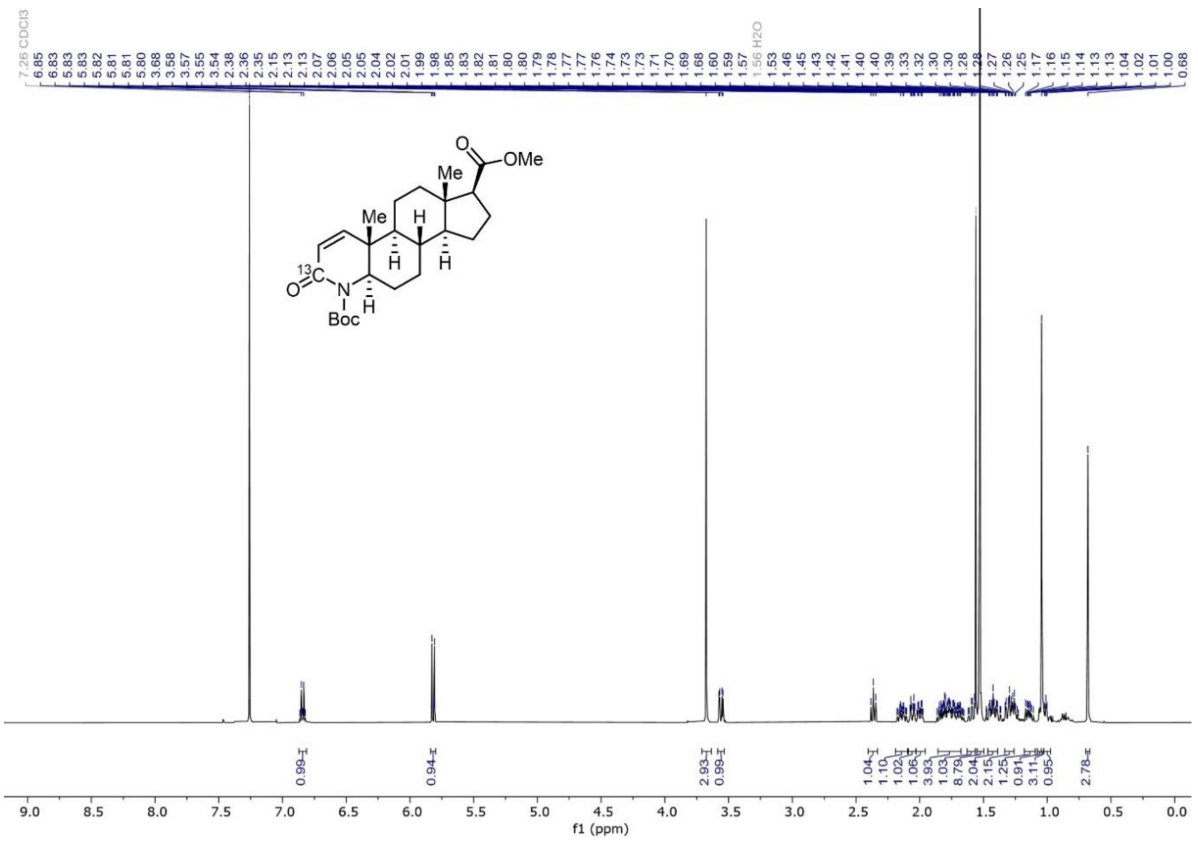

Supplementary Figure 108. ^{1}H NMR spectrum of **^{13}C-20** (500 MHz, chloroform-*d*, 23 °C).

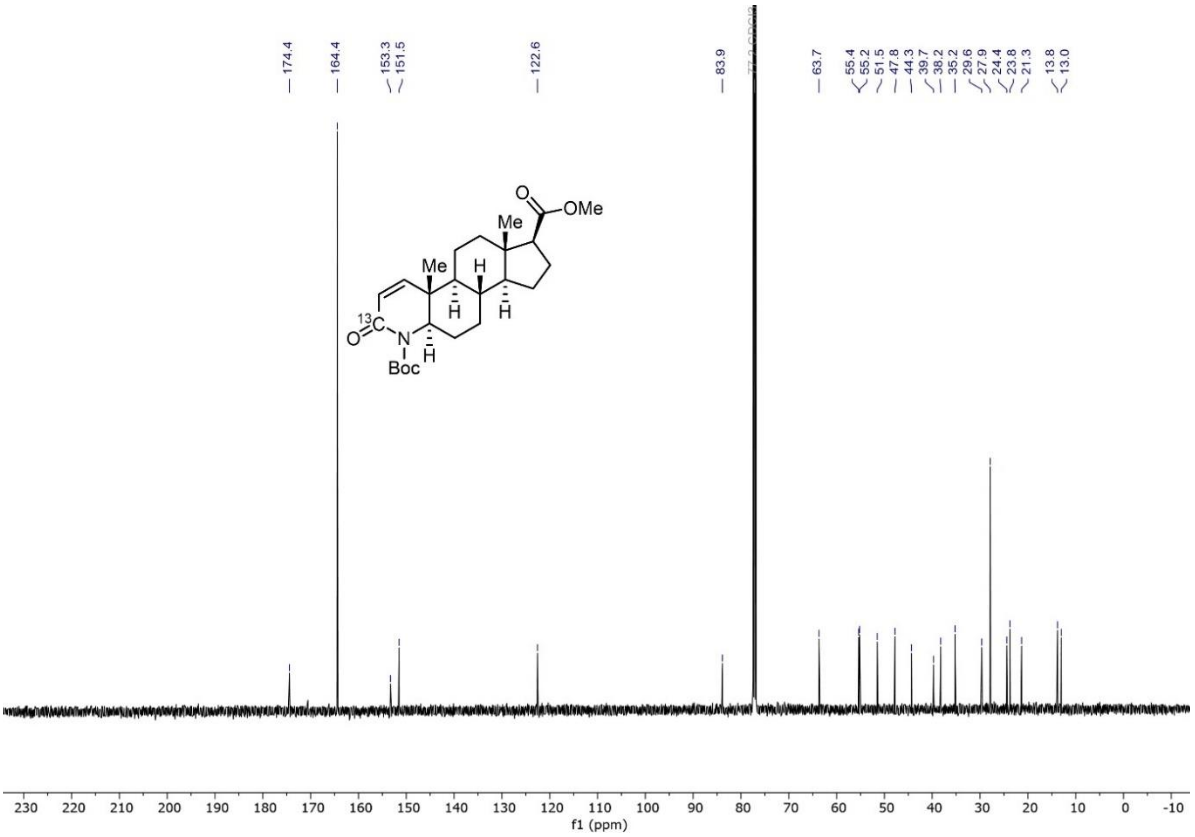

Supplementary Figure 109. ^{13}C{^{1}H} NMR spectrum of **^{13}C-20** (17±1% ^{13}C-labeled, 126 MHz, chloroform-*d*, 23 °C).

9. IR spectra of organometallic compounds

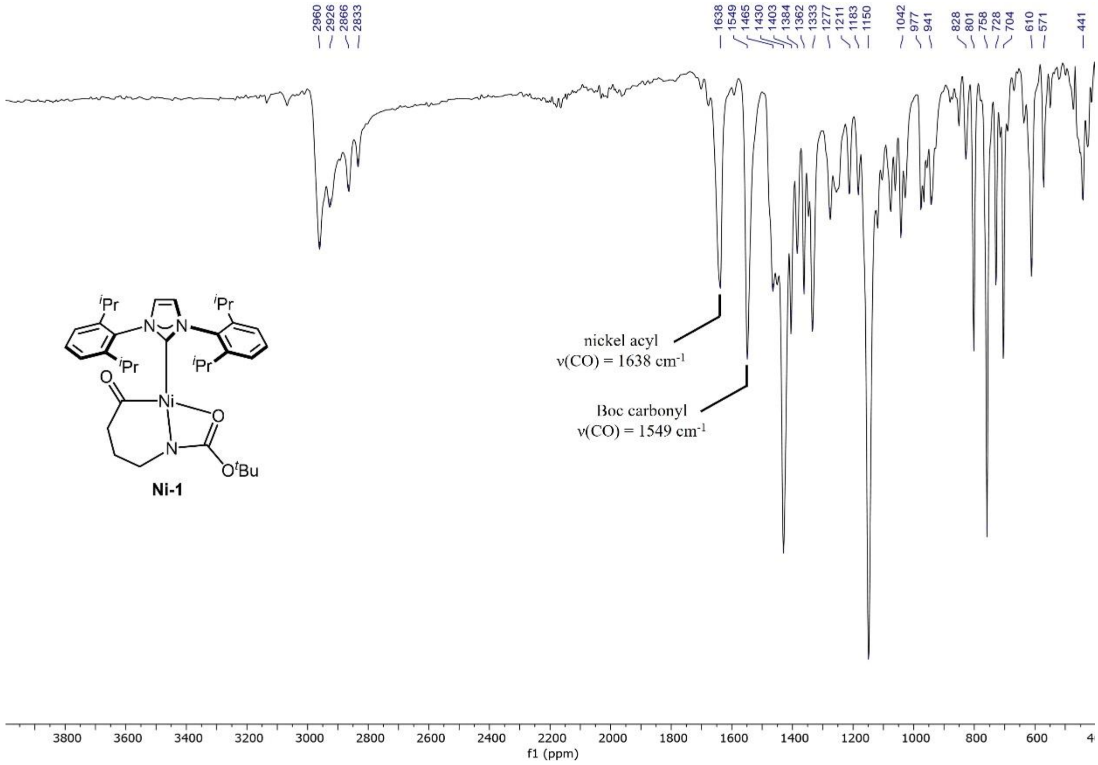

Supplementary Figure 110. ATR-IR spectrum of **Ni-1**.

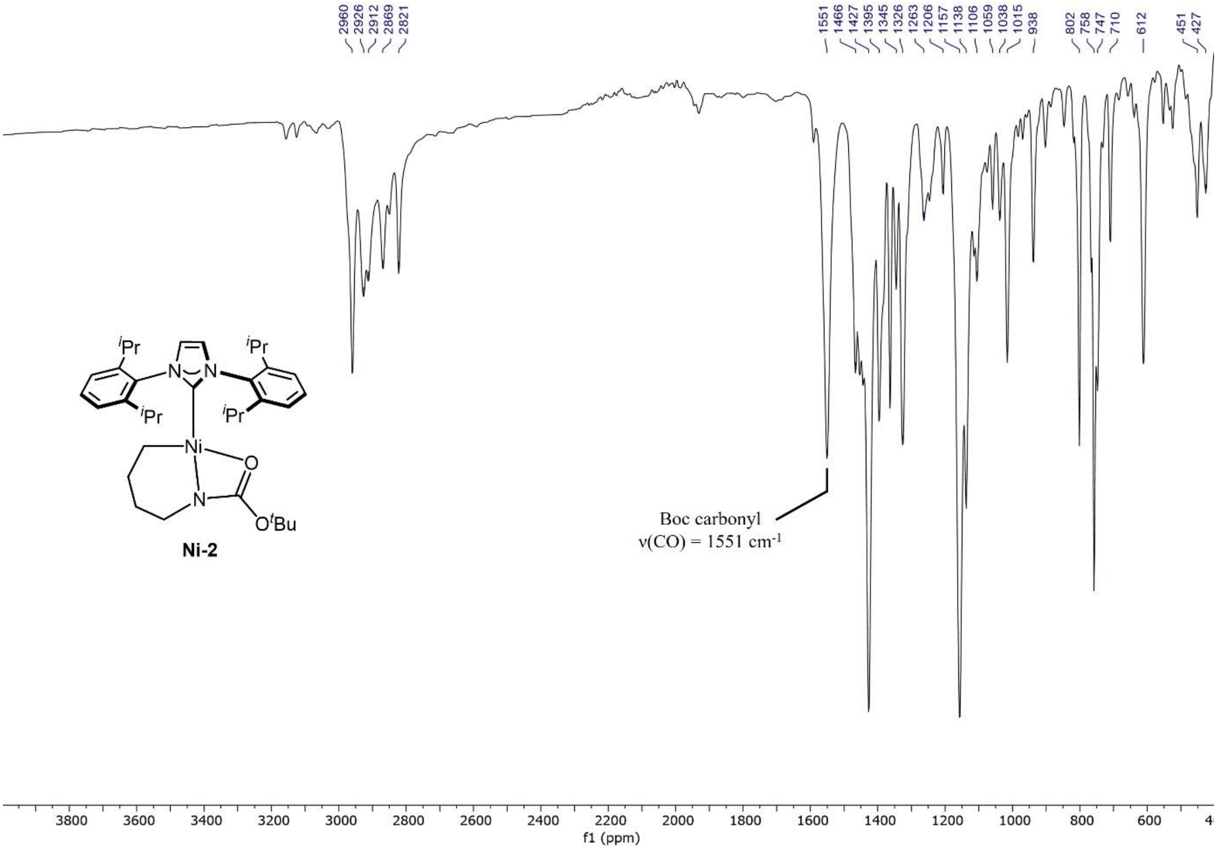

Supplementary Figure 111. ATR-IR spectrum of **Ni-2**.

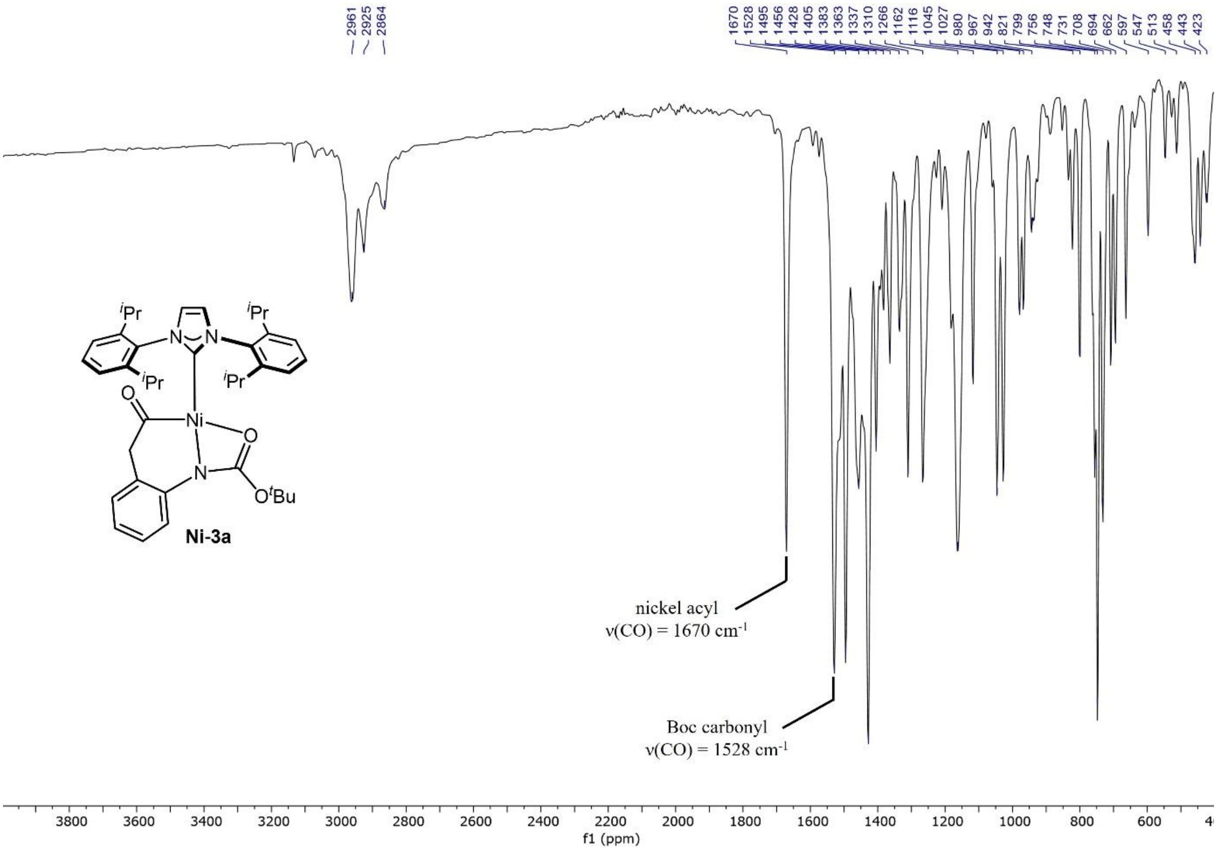

Supplementary Figure 112. ATR-IR spectrum of **Ni-3a**.

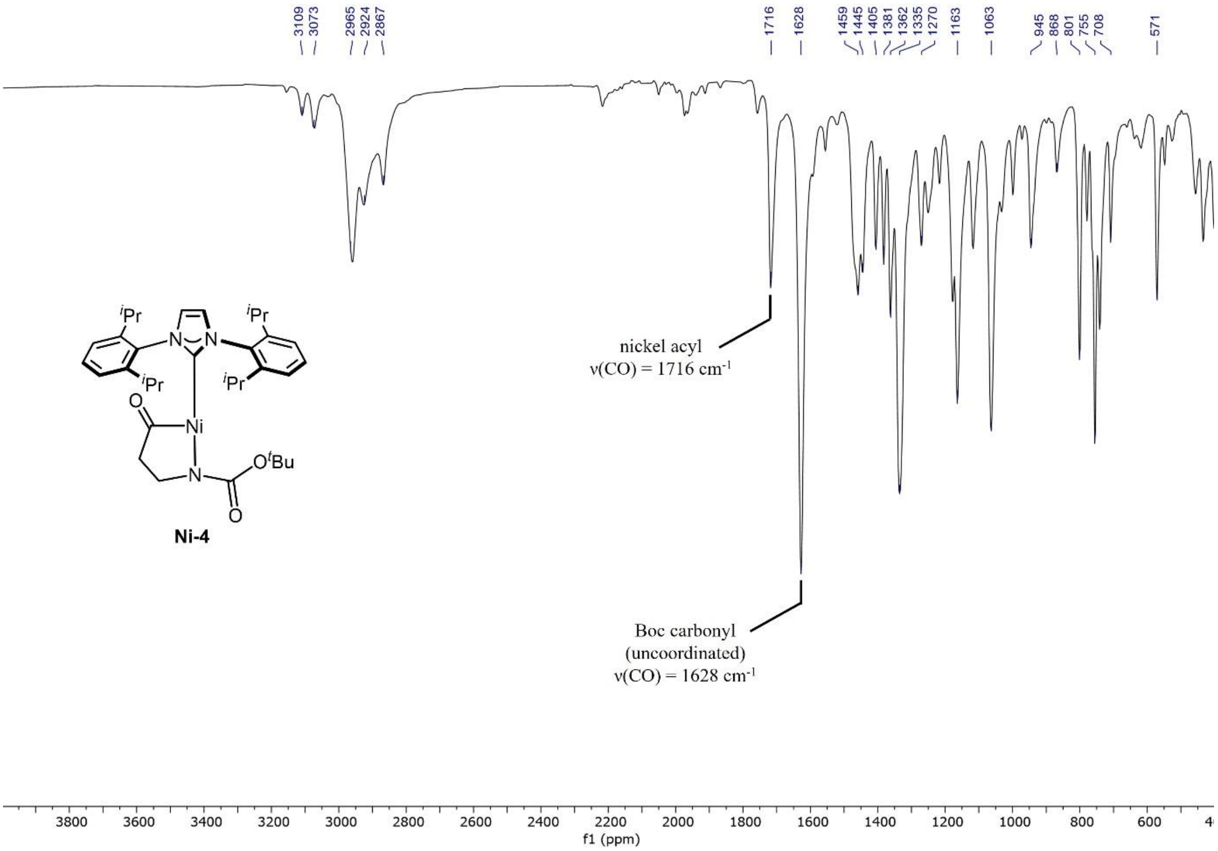

Supplementary Figure 113. ATR-IR spectrum of **Ni-4**.

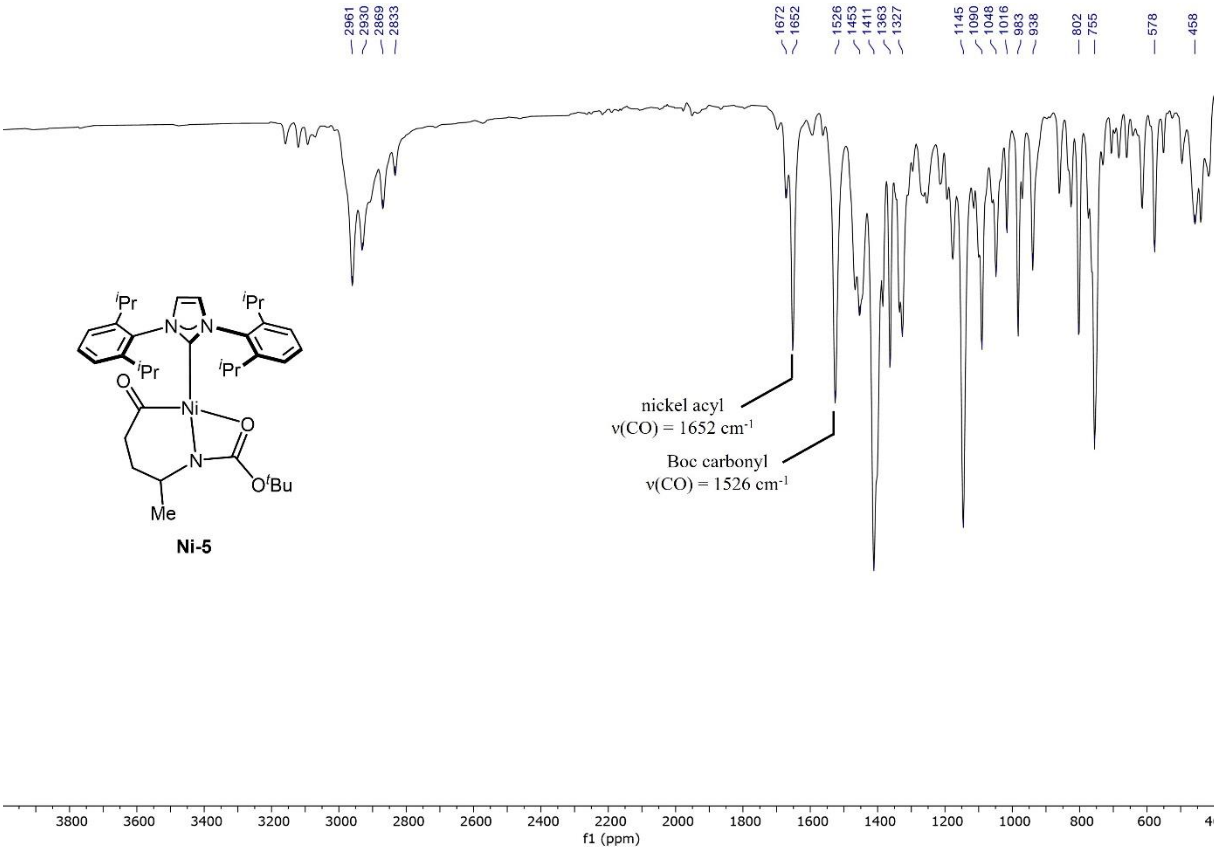

Supplementary Figure 114. ATR-IR spectrum of **Ni-5**.

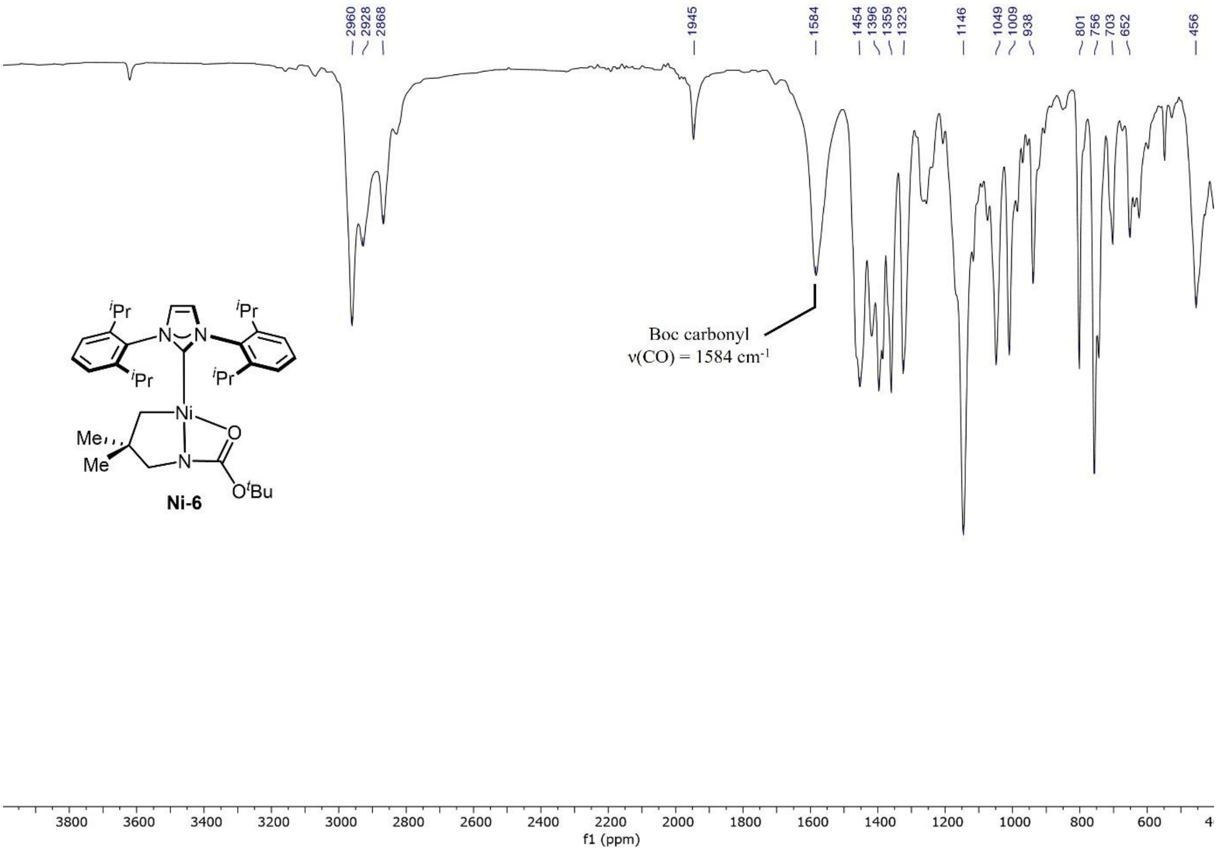

Supplementary Figure 115. ATR-IR spectrum of **Ni-6**.

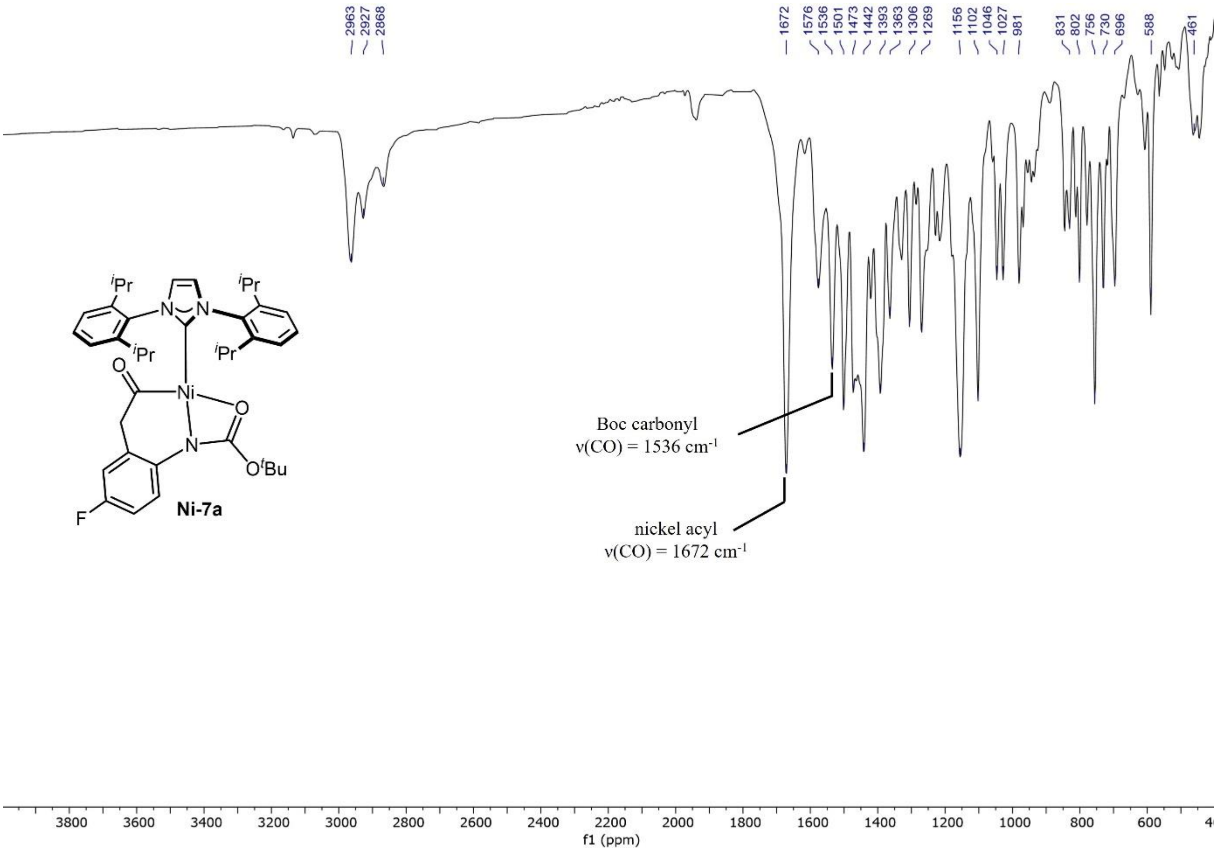

Supplementary Figure 116. ATR-IR spectrum of **Ni-7a**.

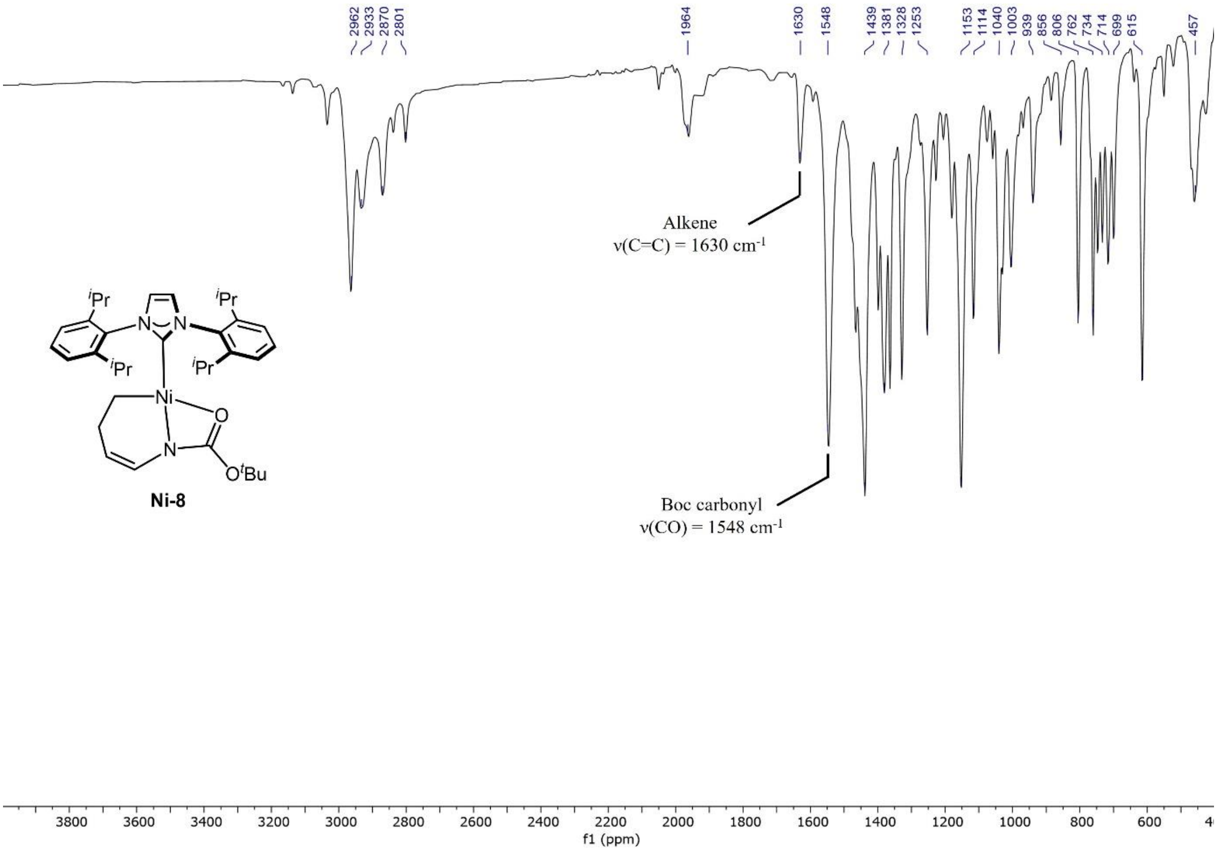

Supplementary Figure 117. ATR-IR spectrum of **Ni-8**.

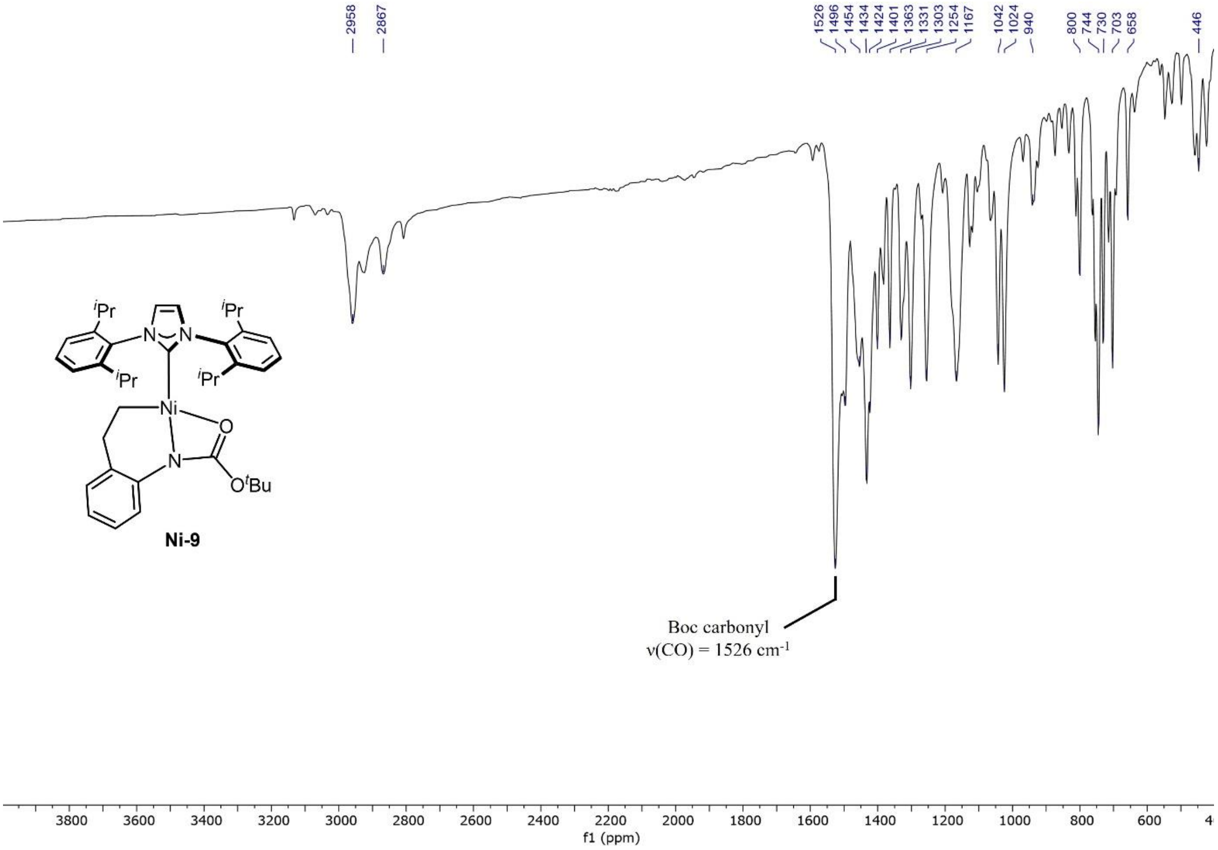

Supplementary Figure 118. ATR-IR spectrum of **Ni-9**.

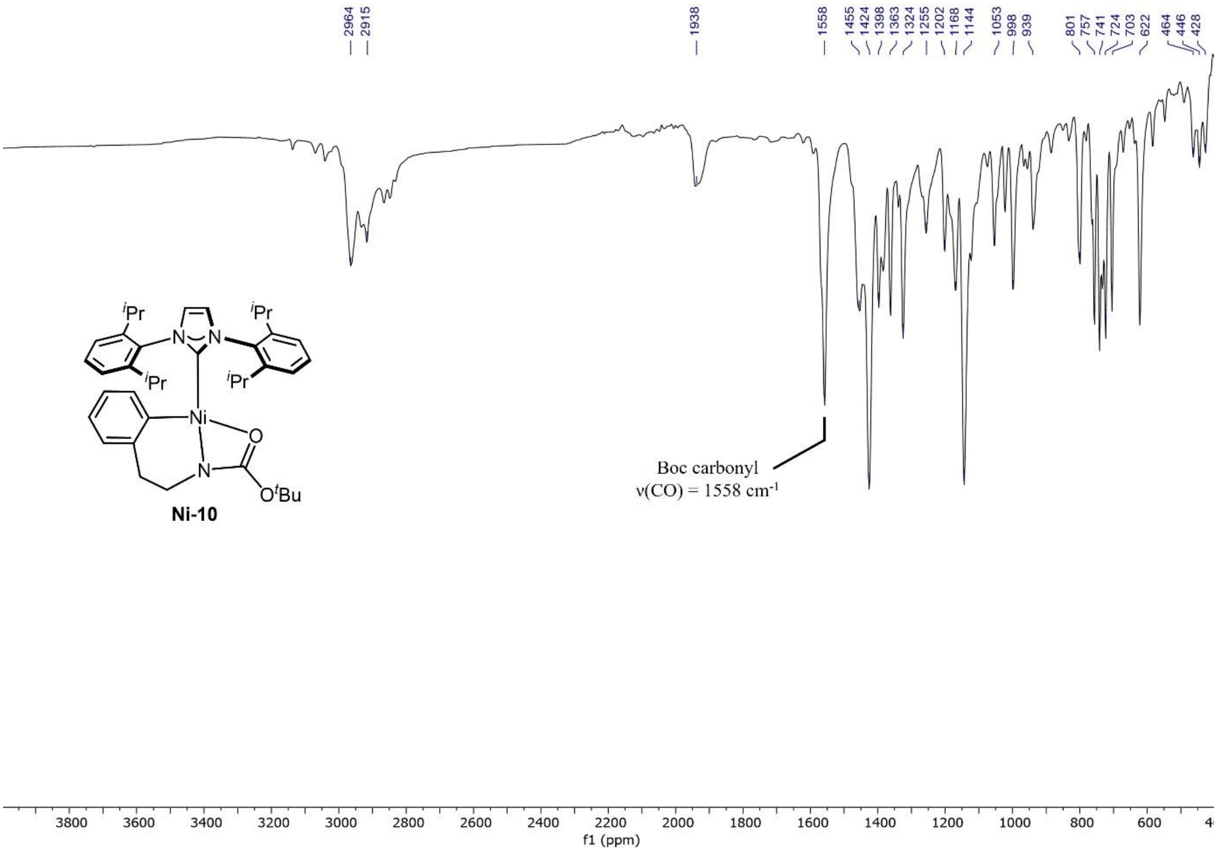

Supplementary Figure 119. ATR-IR spectrum of **Ni-10**.

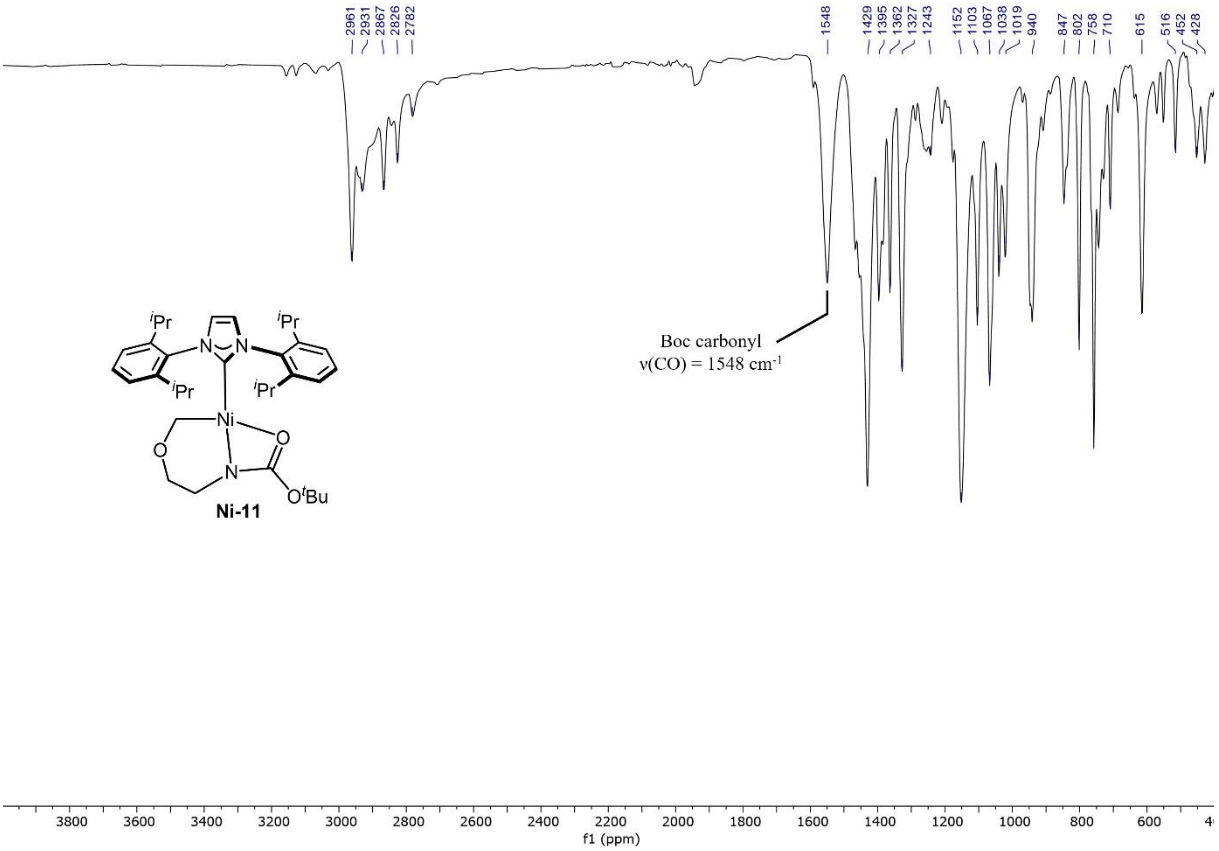

Supplementary Figure 120. ATR-IR spectrum of **Ni-11**.

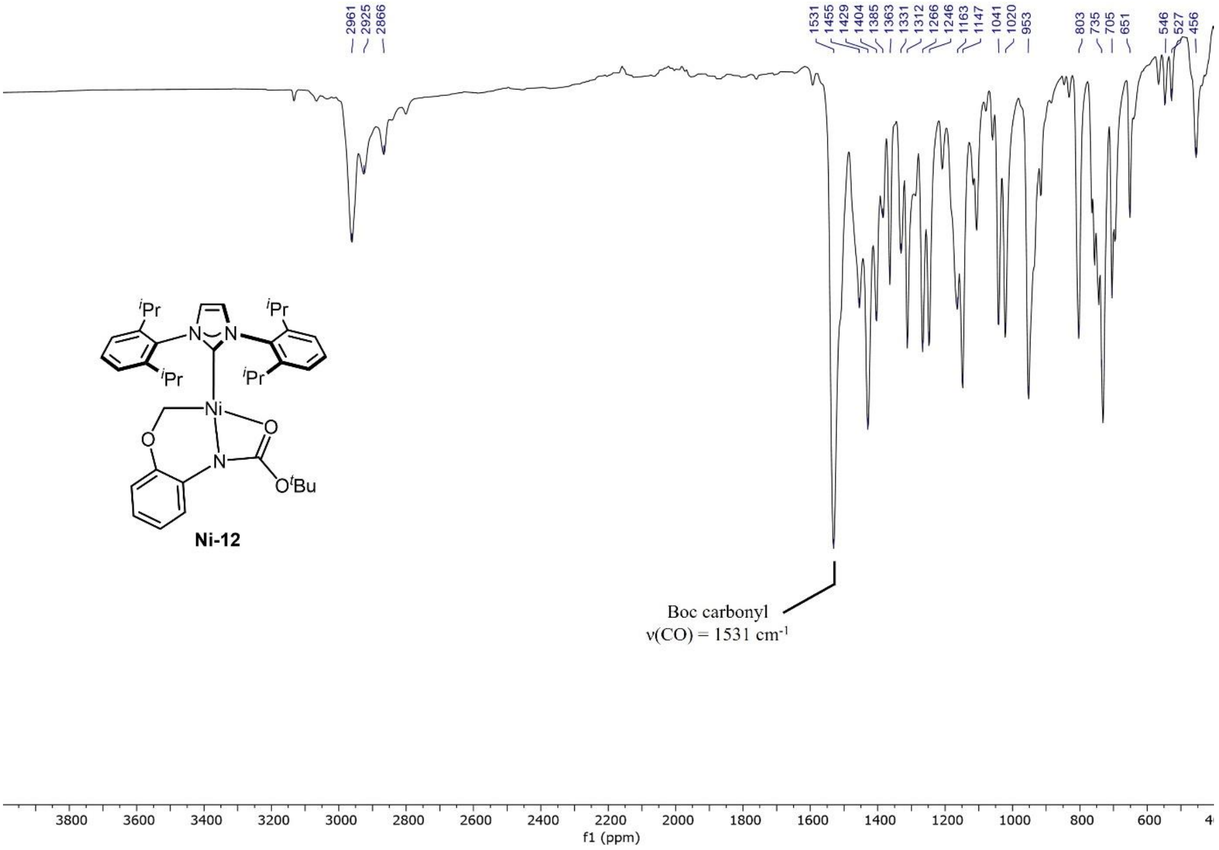

Supplementary Figure 121. ATR-IR spectrum of **Ni-12**.

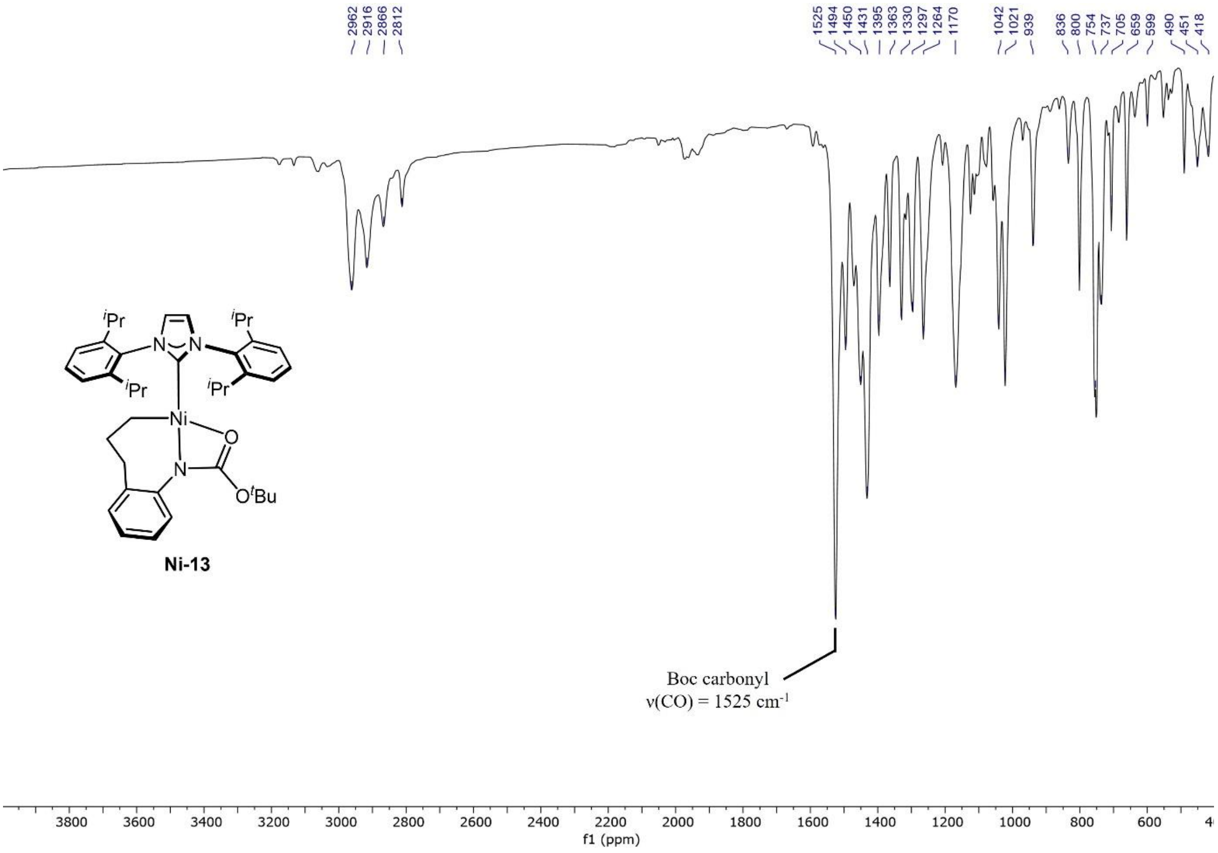

Supplementary Figure 122. ATR-IR spectrum of **Ni-13**.

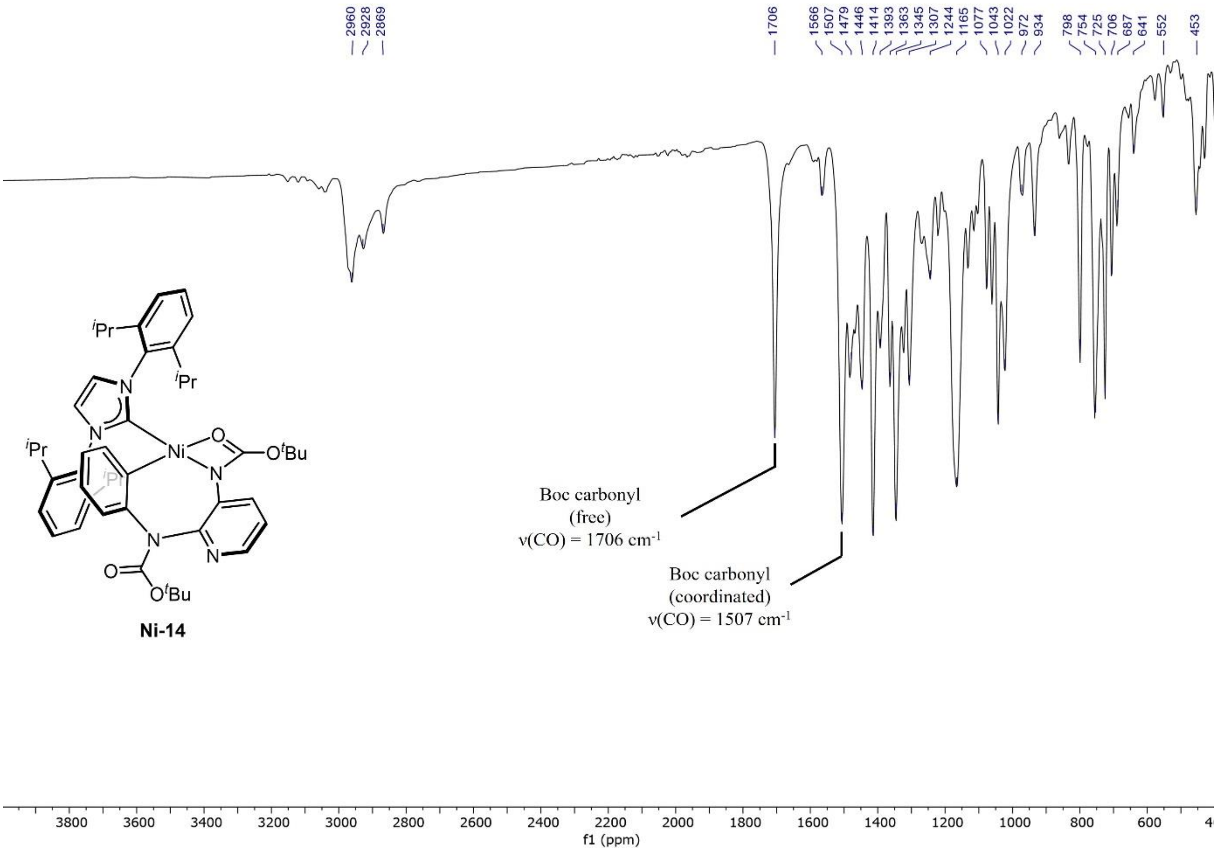

Supplementary Figure 123. ATR-IR spectrum of **Ni-14**.

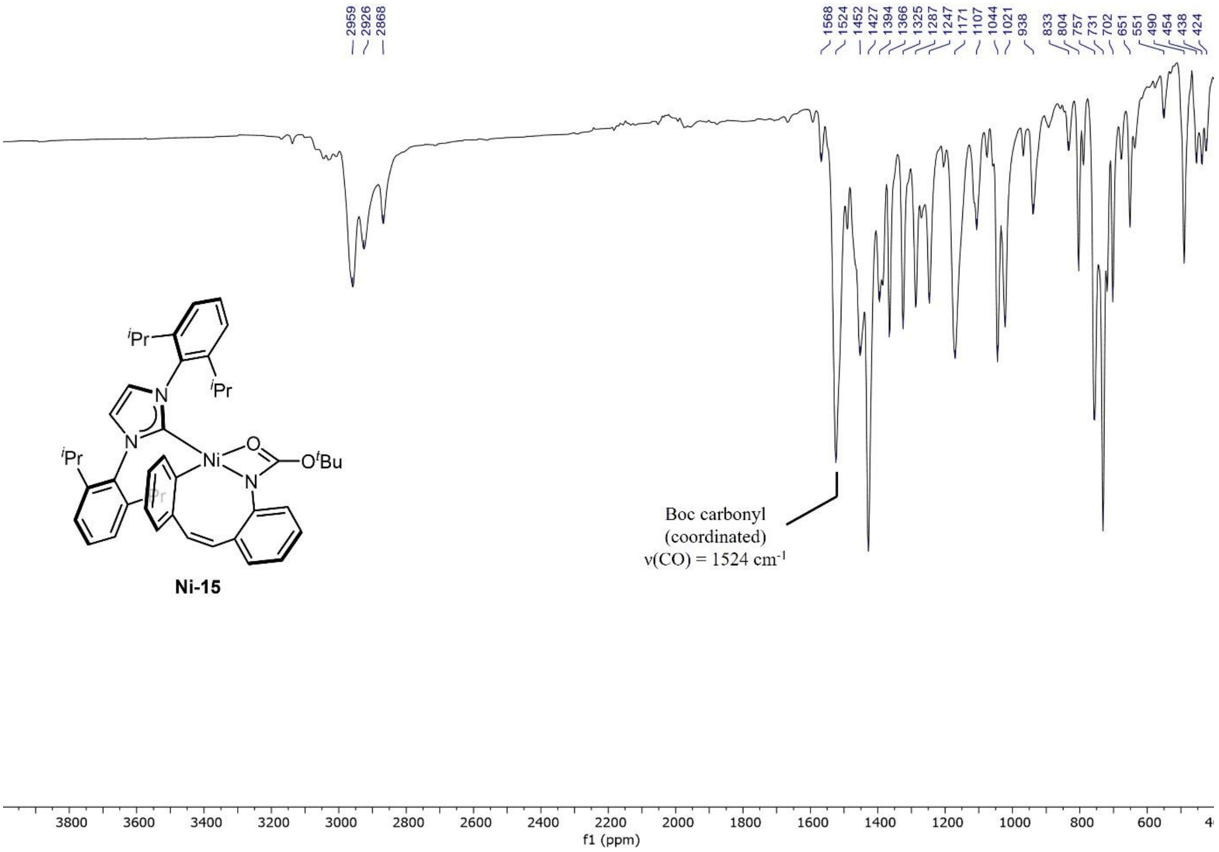

Supplementary Figure 124. ATR-IR spectrum of **Ni-15**.

References

1. Solar, M. & Trapp, N. µCHILL: a lightweight, modular system for handling crystalline samples at low temperatures under inert conditions. *J. Appl. Crystallogr.* **51**, 541–548 (2018).

2. CrysAlisPro and ABSPACK. Rigaku Oxford Diffraction, 2016-2023. https://www.rigaku.com/products/crystallography/crysalis.

3. Sheldrick, G. M. A short history of SHELX. *Acta Crystallogr. Sect. A Found. Crystallogr.* **64**, 112–122 (2008).

4. Sheldrick, G. M. SHELXT - Integrated space-group and crystal-structure determination. *Acta Crystallogr. Sect. A Found. Crystallogr.* **71**, 3–8 (2015).

5. Sheldrick, G. M. Crystal structure refinement with SHELXL. *Acta Crystallogr. Sect. C Struct. Chem.* **71**, 3–8 (2015).

6. Dolomanov, O. V., Bourhis, L. J., Gildea, R. J., Howard, J. A. K. & Puschmann, H. OLEX2: A complete structure solution, refinement and analysis program. *J. Appl. Crystallogr.* **42**, 339–341 (2009).

7. Hoshimoto, Y., Hayashi, Y., Suzuki, H., Ohashi, M. & Ogoshi, S. One-pot, single-step, and gram-scale synthesis of mononuclear [(η^6-arene)Ni(N-heterocyclic carbene)] complexes: Useful precursors of the Ni0-NHC unit. *Organometallics* **33**, 1276–1282 (2014).

8. Chang, H. L., Laitar, D. S., Mueller, P. & Sadighi, J. P. Generation of a doubly bridging CO_2 ligand and deoxygenation of CO_2 by an (NHC)Ni(0) complex. *J. Am. Chem. Soc.* **129**, 13802–13803 (2007).

9. Veryser, C., Van Mileghem, S., Egle, B., Gilles, P. & De Borggraeve, W. M. Low-cost instant CO generation at room temperature using formic acid, mesyl chloride and triethylamine. *React. Chem. Eng.* **1**, 142–146 (2016).

10. Dorta, R. *et al.* Steric and electronic properties of N-heterocyclic carbenes (NHC): A detailed study on their interaction with $Ni(CO)_4$. *J. Am. Chem. Soc.* **127**, 2485–2495 (2005).

11. S. Bair, J. *et al.* Linear-Selective Hydroarylation of Unactivated Terminal and Internal Olefins with Trifluoromethyl-Substituted Arenes. *J. Am. Chem. Soc.* **136**, 13098–13101 (2014).

12. Matsubara, K. *et al.* An unsaturated nickel(0) NHC catalyst: Facile preparation and structure of $Ni(0)(NHC)_2$, featuring a reduction process from $Ni(II)(NHC)(acac)_2$. *Organometallics* **27**, 6020–6024 (2008).

13. De, S., Das, M. K., Bhunia, S. & Bisai, A. Unified Approach to the Spiro(pyrrolidinyl-oxindole) and Hexahydropyrrolo[2,3-b]indole Alkaloids: Total Syntheses of Pseudophrynamines 270 and

272A. *Org. Lett.* **17**, 5922–5925 (2015).

14. Duchemin, N., Buccafusca, R., Daumas, M., Ferey, V. & Arseniyadis, S. A Unified Strategy for the Synthesis of Difluoromethyl-and Vinylfluoride-Containing Scaffolds. *Org. Lett.* **21**, 8205–8210 (2019).

15. Jung, H. Y., Chang, S. & Hong, S. Strategic approach to the metamorphosis of γ-Lactones to NH γ-lactams via reductive cleavage and C-H amidation. *Org. Lett.* **21**, 7099–7103 (2019).

16. Nicewicz, D. A., Pistritto, V. A. & Schutzbach-Horton, M. E. Nucleophilic aromatic substitution of unactivated fluoroarenes enabled by organic photoredox catalysis. *J. Am. Chem. Soc.* **142**, 17187–17194 (2020).

17. Jana, S. & Rainier, J. D. The synthesis of indoline and benzofuran scaffolds using a Suzuki-Miyaura coupling/oxidative cyclization strategy. *Org. Lett.* **15**, 4426–4429 (2013).

18. Keum, H., Jung, H., Jeong, J., Kim, D. & Chang, S. Visible-Light Induced C(sp^2)−H Amidation with an Aryl–Alkyl σ-Bond Relocation via Redox-Neutral Radical–Polar Crossover. *Angew. Chem. Int. Ed.* **60**, 25235–25240 (2021).

19. Patil, M. R., Dedhia, N. P., Kapdi, A. R. & Kumar, A. V. Cobalt(II)/ N -Hydroxyphthalimide-Catalyzed Cross-Dehydrogenative Coupling Reaction at Room Temperature under Aerobic Condition. *J. Org. Chem.* **83**, 4477–4490 (2018).

20. Chandrakumar, N. S. *et al.* Analogs of the .delta. opioid receptor selective cyclic peptide [cyclic][2-D-penicillamine,5-D-penicillamine]-enkephalin: 2’,6’-dimethyltyrosine and Gly3-Phe4 amide bond isostere substitutions. *J. Med. Chem.* **35**, 2928–2938 (1992).

21. Buon, C., Chacun-Lefèvre, L., Rabot, R., Bouyssou, P. & Coudert, G. Synthesis of 3-Substituted and 2,3-Disubstituted-4 H -1,4-Benzoxazines. *Tetrahedron* **56**, 605–614 (2000).

22. Aeyad, T., Jones, C. G. & Coldham, I. Preparation of Substituted Tetrahydro-1-benzazepines by Lithiation-Trapping. *Eur. J. Org. Chem.* **2018**, 5289–5296 (2018).

23. Chadwick, R., Van Gyzen, S., Liogier, S. & Adronov, A. Scalable Synthesis of Strained Cyclooctyne Derivatives. *Synthesis (Stuttg).* **46**, 669–677 (2014).

24. Evans, V., Mahon, M. F. & Webster, R. L. A mild, copper-catalysed amide deprotection strategy: Use of tert-butyl as a protecting group. *Tetrahedron* **70**, 7593–7597 (2014).

25. Kim, S. H. *et al.* Studies on the aza-Claisen rearrangement of 7 to 9-membered vinylazacycles. *Heterocycles* **92**, 886–899 (2016).

26. Peschiulli, A. *et al.* Ruthenium-catalyzed α-(hetero)arylation of saturated cyclic amines: Reaction scope and mechanism. *Chem. Eur. J.* **19**, 10378–10387 (2013).

27. Glaser, F. & Wenger, O. S. Red Light-Based Dual Photoredox Strategy Resembling the Z-Scheme of Natural Photosynthesis. *JACS Au* **2**, 1488–1503 (2022).

28. Subramani, M. & Rajendran, S. K. Mild, Metal-Free and Protection-Free Transamidation of N-Acyl-2-piperidones to Amino Acids, Amino Alcohols and Aliphatic Amines and Esterification of N-Acyl-2-piperidones. *Eur. J. Org. Chem.* **2019**, 3677–3686 (2019).

29. Law, J. A., Bartfield, N. M. & Frederich, J. H. Site-Specific Alkene Hydromethylation via Protonolysis of Titanacyclobutanes. *Angew. Chem. Int. Ed.* **60**, 14360–14364 (2021).

30. Blaszykowski, C., Dhimane, A. L., Fensterbank, L. & Malacria, M. N-silyl-tethered radical cyclizations: A new synthesis of γ-amino alcohols. *Org. Lett.* **5**, 1341–1344 (2003).

31. Jiao, L. & Oestreich, M. Oxidative Palladium(II)-Catalyzed C-7 Alkenylation of Indolines. *Org. Lett.* **15**, 5374–5377 (2013).

32. Bannwarth, C., Ehlert, S. & Grimme, S. GFN2-xTB - An Accurate and Broadly Parametrized Self-Consistent Tight-Binding Quantum Chemical Method with Multipole Electrostatics and Density-Dependent Dispersion Contributions. *J. Chem. Theory Comput.* **15**, 1652–1671 (2019).

33. Bannwarth, C. *et al.* Extended tight-binding quantum chemistry methods. *Wiley Interdiscip. Rev. Comput. Mol. Sci.* **11**, 1–49 (2021).

34. Grimme, S. Exploration of Chemical Compound, Conformer, and Reaction Space with Meta-Dynamics Simulations Based on Tight-Binding Quantum Chemical Calculations. *J. Chem. Theory Comput.* **15**, 2847–2862 (2019).

35. Pracht, P., Bohle, F. & Grimme, S. Automated exploration of the low-energy chemical space with fast quantum chemical methods. *Phys. Chem. Chem. Phys.* **22**, 7169–7192 (2020).

36. Grimme, S. *et al.* Efficient Quantum Chemical Calculation of Structure Ensembles and Free Energies for Nonrigid Molecules. *J. Phys. Chem. A* **125**, 4039–4054 (2021).

37. Neese, F. Software update: The ORCA program system—Version 5.0. *Wiley Interdiscip. Rev. Comput. Mol. Sci.* **12**, 1–15 (2022).

38. Becke, A. D. Density-functional exchange-energy approximation with correct asymptotic behavior. *Phys. Rev. A* **38**, 3098–3100 (1988).

39. Perdew, J. P. Density-functional approximation for the correlation energy of the inhomogeneous electron gas. *Phys. Rev. B* **33**, 8822–8824 (1986).

40. Weigend, F. & Ahlrichs, R. Balanced basis sets of split valence, triple zeta valence and quadruple zeta valence quality for H to Rn: Design and assessment of accuracy. *Phys. Chem. Chem. Phys.* **7**, 3297–3305 (2005).

41. Grimme, S., Ehrlich, S. & Goerigk, L. Effect of the damping function in dispersion corrected density functional theory. *J. Comput. Chem.* **32**, 1456–1465 (2011).

42. Grimme, S., Antony, J., Ehrlich, S. & Krieg, H. A consistent and accurate ab initio parametrization of density functional dispersion correction (DFT-D) for the 94 elements H-Pu. *J. Chem. Phys.* **132**, (2010).

43. Weigend, F. Accurate Coulomb-fitting basis sets for H to Rn. *Phys. Chem. Chem. Phys.* **8**, 1057–1065 (2006).

44. Barone, V. & Cossi, M. Quantum Calculation of Molecular Energies and Energy Gradients in Solution by a Conductor Solvent Model. *J. Phys. Chem. A* **102**, 1995–2001 (1998).

45. Adamo, C. & Barone, V. Toward reliable density functional methods without adjustable parameters: The PBE0 model. *J. Chem. Phys.* **110**, 6158–6170 (1999).

46. Legault, C. Y. CYLview20. http://www.cylview.org (2020).

47. Iglesias, M. J. *et al.* Synthesis, structural characterization, and catalytic activity of IPrNi(styrene)$_2$ in the amination of aryl tosylates. *Organometallics* **31**, 6312–6316 (2012).

48. Nett, A. J. *et al.* Stable, Well-Defined Nickel(0) Catalysts for Catalytic C-C and C-N Bond Formation. *ACS Catal.* **8**, 6606–6611 (2018).

49. Nattmann, L., Saeb, R., Nöthling, N. & Cornella, J. An air-stable binary Ni(0)–olefin catalyst. *Nat. Catal.* **3**, 6–13 (2020).

50. Adhikari, D., Nguyen, S. B. T. & Baik, M. H. A computational study of the mechanism of the [(salen)Cr + DMAP]-catalyzed formation of cyclic carbonates from CO_2 and epoxide. *Chem. Commun.* **50**, 2676–2678 (2014).

51. Ryu, H. *et al.* Pitfalls in Computational Modeling of Chemical Reactions and How to Avoid Them. *Organometallics* **37**, 3228–3239 (2018).

52. Wang, H., Zhang, S. Q. & Hong, X. Computational studies on Ni-catalyzed amide C-N bond activation. *Chem. Commun.* **55**, 11330–11341 (2019).

53. Xie, P. P., Qin, Z. X., Zhang, S. Q. & Hong, X. Understanding the Structure-Activity Relationship of Ni-Catalyzed Amide C−N Bond Activation using Distortion/Interaction Analysis. *ChemCatChem* **13**, 3536–3542 (2021).

54. Lu, Q., Yu, H. & Fu, Y. Mechanistic study of chemoselectivity in ni-catalyzed coupling reactions between azoles and aryl carboxylates. *J. Am. Chem. Soc.* **136**, 8252–8260 (2014).

55. Beattie, D. D., Bowes, E. G., Drover, M. W., Love, J. A. & Schafer, L. L. Oxidation State

Dependent Coordination Modes: Accessing an Amidate-Supported Nickel(I) δ-bis(C−H) Agostic Complex. *Angew. Chem. Int. Ed.* **55**, 13290–13295 (2016).
